# Supplementary material for: A phase 2 open-label study of the safety and efficacy of weekly dosing of ATL1102 in patients with non-ambulatory Duchenne muscular dystrophy and pharmacology in mdx mice
Source: PLoS One. 2024 Jan 25;19(1):e0294847. doi: 10.1371/journal.pone.0294847 (PMC10810432; doi:10.1371/journal.pone.0294847)
Supplement: S1 Fig — (DOCX) [file pone.0294847.s002.docx]

*Figure S1:* Inclusion and Exclusion criteria for the clinical trial

*Inclusion Criteria: Participants who:*

1. *Are adolescent males, aged 10 to 18 years inclusive, at the time of providing informed consent.*
2. *Have been diagnosed with Duchenne Muscular Dystrophy and have been non-ambulatory for at least 3 months. Non-ambulatory for this study is defined as having consistently required a wheelchair to mobilise more than a few metres for at least 3 months.*
3. *The diagnosis of DMD is to be confirmed by at least one of the following:*
   1. *Dystrophin immunofluorescence and/or immunoblot showing near complete dystrophin protein deficiency, and clinical picture consistent with typical DMD; or*
   2. *Gene deletion test positive (missing one or more exons) of the dystrophin gene, where the reading frame can be predicted as 'out-of frame', and clinical picture consistent with typical DMD; or*
   3. *Complete dystrophin gene sequencing showing an alteration (point mutation, duplication, or other mutation resulting in a stop codon) that can be definitely associated with DMD, with a typical clinical picture of DMD; or*
   4. *Positive family history of DMD confirmed by one of the criteria listed above in a sibling or maternal uncle, and clinical picture typical of DMD.*
4. *Have a body weight of more than 25 kg and less than or equal to 65 kg.*
5. *If currently receiving glucocorticoid therapy, have been on a stable dose of glucocorticoid therapy for at least 3 months prior to Day 1.*
6. *Are currently on stable doses of cardiac therapy (including angiotensin converting enzyme inhibitors, aldosterone receptor antagonists and/or beta blockers) for at least 3 months prior to Day 1.*
7. *Have a parent/guardian who is capable of understanding the purposes and risks of the study and able to provide written informed consent. If the participant is of sufficient maturity and has the ability to understand the nature and consequence of the study, involvement in study consent discussions are required.*
8. *Are able, and have a parent/guardian who are, willing and able to comply with scheduled visits, study drug administration plan, and study procedures.*

*Exclusion Criteria: Participants who:*

1. *Have been diagnosed with Duchenne Muscular Dystrophy and are still ambulatory. Ambulatory for this study is being able to complete at least 75 meters during the 6-minute walk test in the 4 weeks prior to Day 1.*
2. *Have the following abnormal haematology values during the Screening period or on Day 1, prior to first dose:*
   1. *Lymphocytes <1.2 x 109/L*
   2. *Neutrophils <1.8 x 109/L*
   3. *Platelets <150 x 109/L*
3. *Have a history of clinically significant bleeding or coagulation abnormalities.*
4. *Have hepatic dysfunction indicated by an abnormal total bilirubin and gamma glutamyl transferase (GGT) results at Screening.*
5. *Have renal impairment indicated by serum creatinine ≥ 1.5 mg/dL (132 umol/l) at Screening.*
6. *Have uncontrolled clinical symptoms and signs of congestive heart failure consistent with Stage C or Stage D criteria according to the American College of Cardiology/American Heart Association guidelines for cardiac dysfunction within 3 months of Day 1.*
7. *Have an inability to complete the cardiac, pulmonary or strength range of motion and mobility assessments at Screening.*
8. *Have taken nutritional, herbal, or antioxidant supplements that have a known demonstrated activity for maintaining or improving skeletal muscle strength or functional mobility within 4 weeks of Day 1. NOTE: daily multivitamin, Vitamin D or calcium supplements are permitted.*
9. *Are currently receiving antiplatelet or anticoagulant therapy, or have taken medication with an antiplatelet or anticoagulant effect within 4 weeks prior Day 1 (e.g., aspirin).*
10. *Have received any investigational product in the 2 months prior to Screening (4 months if the previous drug was a new chemical entity), whichever is longer.*
11. *Have severe behavioural disorder or inadequate cognitive development that would make them unable to comply with the study assessments, which, in the opinion of the investigator, makes the participant unsuitable for participation in the study.*
