# Supplementary figures and images for: A phase 2 open-label study of the safety and efficacy of weekly dosing of ATL1102 in patients with non-ambulatory Duchenne muscular dystrophy and pharmacology in mdx mice
Source: PLoS One. 2024 Jan 25;19(1):e0294847. doi: 10.1371/journal.pone.0294847 (PMC10810432; doi:10.1371/journal.pone.0294847)

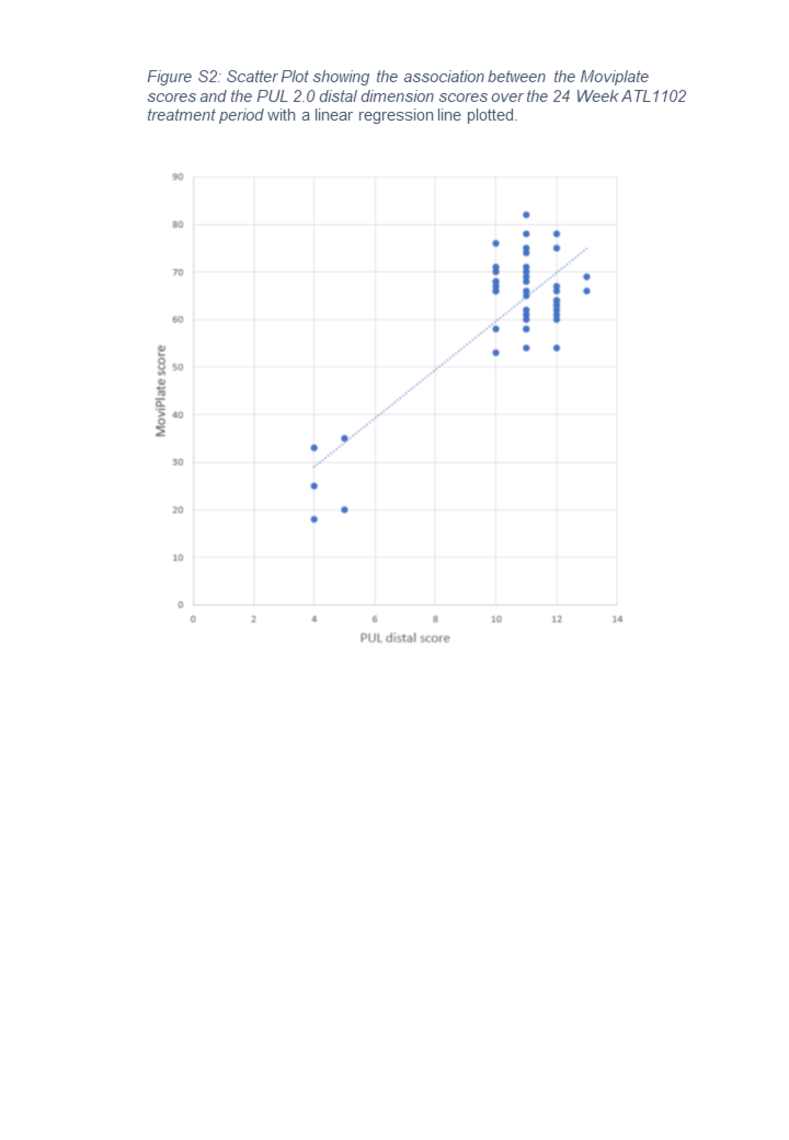

Supplement: S2 Fig — (TIF) [file pone.0294847.s003.tif]

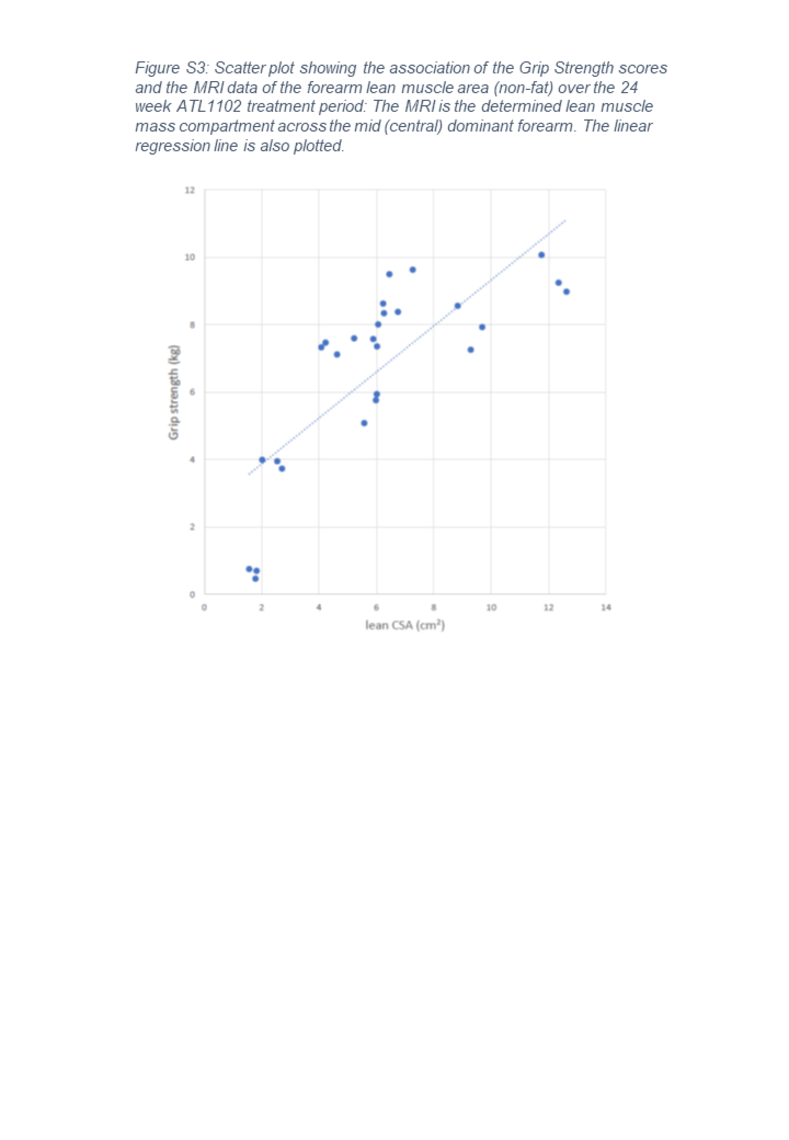

Supplement: S3 Fig — The linear regression line is also plotted. (TIF) [file pone.0294847.s004.tif]

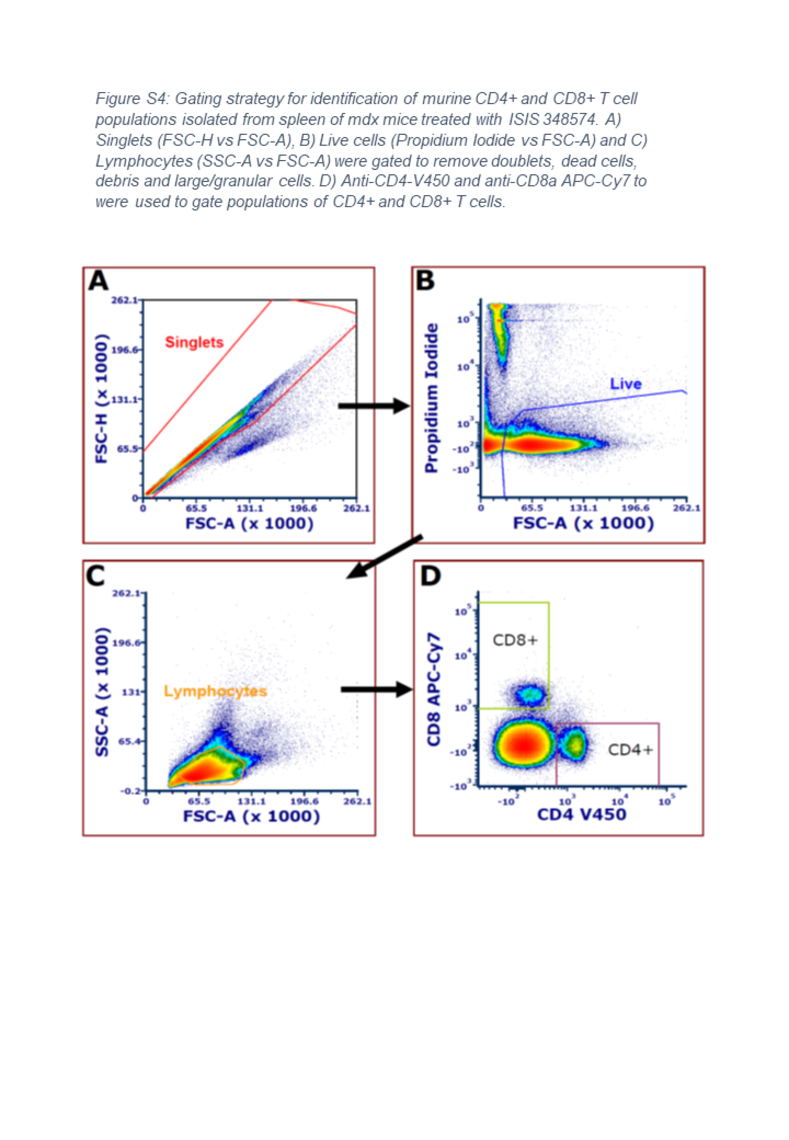

Supplement: S4 Fig — A) Singlets (FSC-H vs FSC-A), B) Live cells (Propidium Iodide vs FSC-A) and C) Lymphocytes (SSC-A vs FSC-A) were gated to remove doublets, dead cells, debris and large/granular cells. D) Anti-CD4-V450 and anti-CD8a APC-Cy7 to were used to gate populations of CD4+ and CD8+ T cells. (TIF) [file pone.0294847.s005.tif]

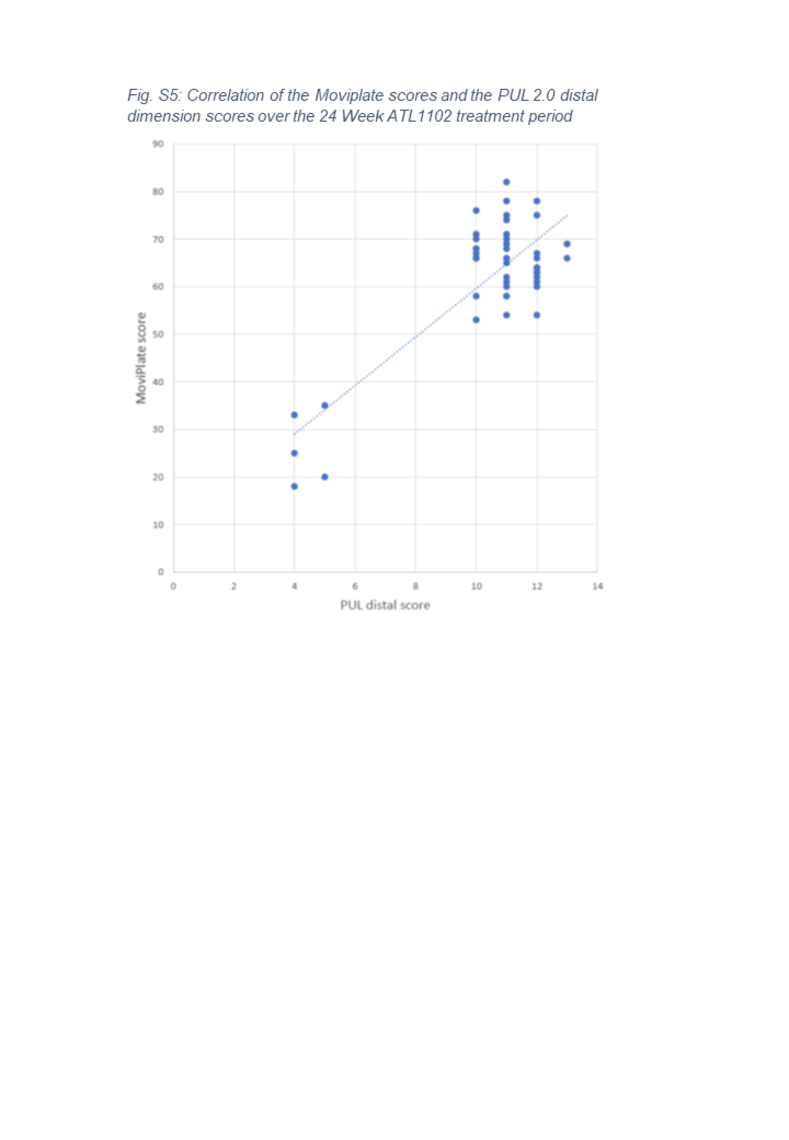

Supplement: S5 Fig — (TIF) [file pone.0294847.s006.tif]

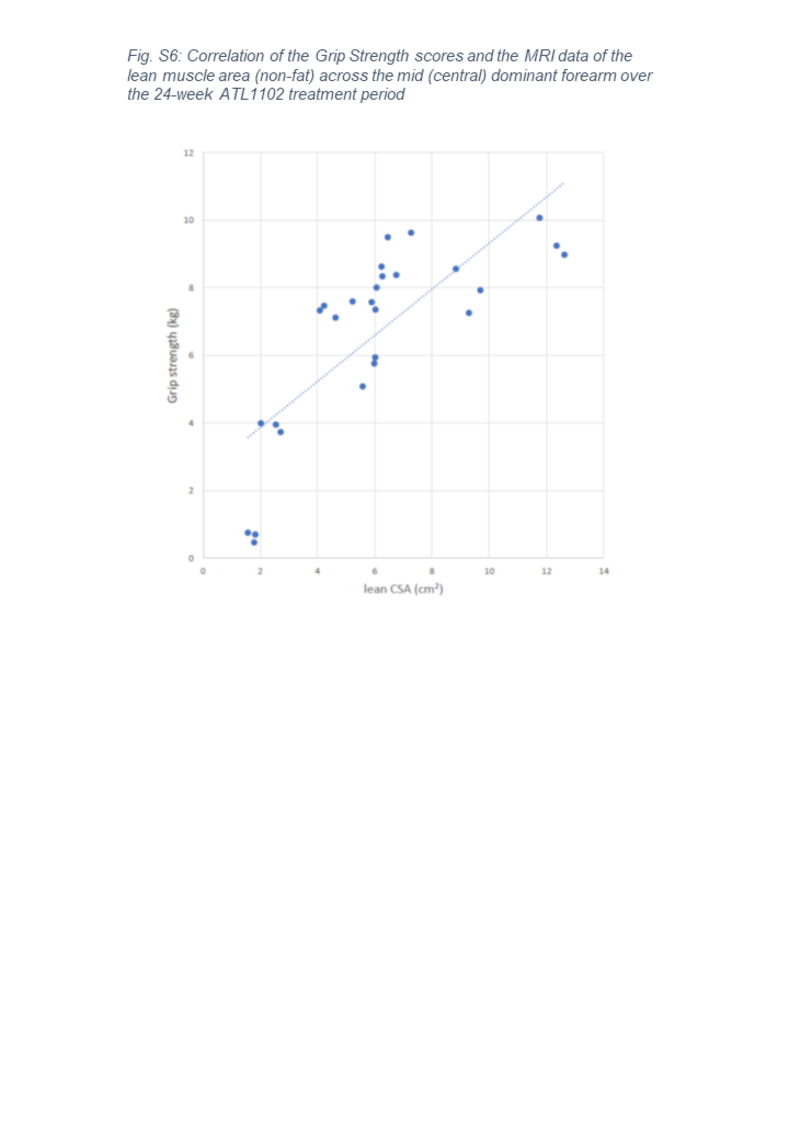

Supplement: S6 Fig — (TIF) [file pone.0294847.s007.tif]
