# Supplementary material for: A phase 2 open-label study of the safety and efficacy of weekly dosing of ATL1102 in patients with non-ambulatory Duchenne muscular dystrophy and pharmacology in mdx mice
Source: PLoS One. 2024 Jan 25;19(1):e0294847. doi: 10.1371/journal.pone.0294847 (PMC10810432; doi:10.1371/journal.pone.0294847)
Supplement: S7 Fig — (DOCX) [file pone.0294847.s008.docx]

Figure S7: Table of Participant specific genetic variant within Dystrophin Gene

| Participant Number | Specific Genetic Variant within Dystrophin Gene |
| --- | --- |
| 1001 | Deletion of exons 48-50 |
| 1002 | splice site mutation in intron 45 |
| 1003 | deletion of exon 50 |
| 1004 | duplication within exon 15 causing premature stop codon |
| 1006 | deletion of exon 50 |
| 1008 | deletion of exons 8-27 |
| 1009 | nonsense substitution mutation in exon 23 causing premature stop codon |
| 1010 | nonsense mutation in exon 52 causing a premature stop codon |
| 1011 | deletion of exons 8-25 |
