# Supplementary material for: A phase 2 open-label study of the safety and efficacy of weekly dosing of ATL1102 in patients with non-ambulatory Duchenne muscular dystrophy and pharmacology in mdx mice
Source: PLoS One. 2024 Jan 25;19(1):e0294847. doi: 10.1371/journal.pone.0294847 (PMC10810432; doi:10.1371/journal.pone.0294847)
Supplement: S1 File — (PDF) [file pone.0294847.s009.pdf]

Listing 16.4 Listing of MRI Cross Sectional Muscle Area (mm2)

Antisense Therapeutics Limited
Protocol: 1102-DMD-CT02
Population: All Enrolled Subjects (excludes Screen Failures)

Subject ID	Date	Visit	Measure	Location	Reading	Status	Arm Measured	Dorsal Muscles	Volar Muscle	ECRLB-Br	Total Area (mm2)	Change from Screening Total Area (mm2)
01001	27-AUG-2018	Visit 1 (Screening)	Cross Sectional Muscle Area (mm2)	Forearm	Central Reading	Dominant	Right	417	1000	108	1525	
				Forearm	Distal Reading	Dominant	Right	105	377	Not measurable	482	
				Forearm	Proximal Reading	Dominant	Right	371	705	260	1336	
	19-NOV-2018	Visit 8 (Week 12, Day 81)	Cross Sectional Muscle Area (mm2)	Forearm	Central Reading	Dominant	Right	410	1067	105	1582	57
				Forearm	Distal Reading	Dominant	Right	116	348	Not measurable	464	-18
				Forearm	Proximal Reading	Dominant	Right	385	698	248	1331	-5

Listing 16.4 Listing of MRI Cross Sectional Muscle Area (mm2)

Antisense Therapeutics Limited
Protocol: 1102-DMD-CT02
Population: All Enrolled Subjects (excludes Screen Failures)

Subject ID	Date	Visit	Measure	Location	Reading	Status	Arm Measured	Dorsal Muscles	Volar Muscle	ECRLB-Br	Total Area (mm2)	Change from Screening Total Area (mm2)
01001	18-FEB-2019	Visit 14 (Week 24, Day 165)	Cross Sectional Muscle Area (mm2)	Forearm	Central Reading	Dominant	Right	421	1095	100	1616	91
				Forearm	Distal Reading	Dominant	Right	123	322	Not measurable	445	-37
				Forearm	Proximal Reading	Dominant	Right	386	715	275	1376	40
01002	29-OCT-2018	Visit 1 (Screening)	Cross Sectional Muscle Area (mm2)	Forearm	Central Reading	Dominant	Right	482	769	207	1458	
				Forearm	Distal Reading	Dominant	Right	174	449	Not measurable	623	
				Forearm	Proximal Reading	Dominant	Right	409	729	166	1304	
	21-JAN-2019	Visit 8 (Week 12, Day 81)	Cross Sectional Muscle Area (mm2)	Forearm	Central Reading	Dominant	Right	468	751	213	1432	-26

Listing 16.4 Listing of MRI Cross Sectional Muscle Area (mm2)

Antisense Therapeutics Limited
Protocol: 1102-DMD-CT02
Population: All Enrolled Subjects (excludes Screen Failures)

Subject ID	Date	Visit	Measure	Location	Reading	Status	Arm Measured	Dorsal Muscles	Volar Muscle	ECRLB-Br	Total Area (mm2)	Change from Screening Total Area (mm2)
01002	21-JAN-2019	Visit 8 (Week 12, Day 81)	Cross Sectional Muscle Area (mm2)	Forearm	Distal Reading	Dominant	Right	170	426	Not measurable	596	-27
				Forearm	Proximal Reading	Dominant	Right	389	806	200	1395	91
	01-APR-2019	Visit 14 (Week 24, Day 165)	Cross Sectional Muscle Area (mm2)	Forearm	Central Reading	Dominant	Right	479	785	227	1491	33
				Forearm	Distal Reading	Dominant	Right	182	445	Not measurable	627	4
				Forearm	Proximal Reading	Dominant	Right	399	758	197	1354	50
01003	12-NOV-2018	Visit 1 (Screening)	Cross Sectional Muscle Area (mm2)	Forearm	Central Reading	Dominant	Right	489	671	59	1219	
				Forearm	Distal Reading	Dominant	Right	259	378	Not measurable	637	

Listing 16.4 Listing of MRI Cross Sectional Muscle Area (mm2)

Antisense Therapeutics Limited
Protocol: 1102-DMD-CT02
Population: All Enrolled Subjects (excludes Screen Failures)

Subject ID	Date	Visit	Measure	Location	Reading	Status	Arm Measured	Dorsal Muscles	Volar Muscle	ECRLB-Br	Total Area (mm2)	Change from Screening Total Area (mm2)
01003	12-NOV-2018	Visit 1 (Screening)	Cross Sectional Muscle Area (mm2)	Forearm	Proximal Reading	Dominant	Right	524	928	179	1631	
	11-FEB-2019	Visit 8 (Week 12, Day 81)	Cross Sectional Muscle Area (mm2)	Forearm	Central Reading	Dominant	Right	480	722	60	1262	43
				Forearm	Distal Reading	Dominant	Right	268	379	Not measurable	647	10
				Forearm	Proximal Reading	Dominant	Right	477	891	179	1547	-84
	29-APR-2019	Visit 14 (Week 24, Day 165)	Cross Sectional Muscle Area (mm2)	Forearm	Central Reading	Dominant	Right	530	740	58	1328	109
				Forearm	Distal Reading	Dominant	Right	296	346	Not measurable	642	5
				Forearm	Proximal Reading	Dominant	Right	789	453	176	1418	-213

Listing 16.4 Listing of MRI Cross Sectional Muscle Area (mm2)

Antisense Therapeutics Limited
Protocol: 1102-DMD-CT02
Population: All Enrolled Subjects (excludes Screen Failures)

Subject ID	Date	Visit	Measure	Location	Reading	Status	Arm Measured	Dorsal Muscles	Volar Muscle	ECRLB-Br	Total Area (mm2)	Change from Screening Total Area (mm2)
01004	14-JAN-2019	Visit 1 (Screening)	Cross Sectional Muscle Area (mm2)	Forearm	Central Reading	Dominant	Right	829	1548	173	2550	
				Forearm	Distal Reading	Dominant	Right	252	586	Not measurable	838	
				Forearm	Proximal Reading	Dominant	Right	1031	1106	315	2452	
	08-APR-2019	Visit 8 (Week 12, Day 81)	Cross Sectional Muscle Area (mm2)	Forearm	Central Reading	Dominant	Right	825	1498	174	2497	-53
				Forearm	Distal Reading	Dominant	Right	228	557	Not measurable	785	-53
				Forearm	Proximal Reading	Dominant	Right	926	1104	312	2342	-110
	01-JUL-2019	Visit 14 (Week 24, Day 165)	Cross Sectional Muscle Area (mm2)	Forearm	Central Reading	Dominant	Right	804	1487	168	2459	-91

Listing 16.4 Listing of MRI Cross Sectional Muscle Area (mm2)

Antisense Therapeutics Limited
Protocol: 1102-DMD-CT02
Population: All Enrolled Subjects (excludes Screen Failures)

Subject ID	Date	Visit	Measure	Location	Reading	Status	Arm Measured	Dorsal Muscles	Volar Muscle	ECRLB-Br	Total Area (mm2)	Change from Screening Total Area (mm2)
01004	01-JUL-2019	Visit 14 (Week 24, Day 165)	Cross Sectional Muscle Area (mm2)	Forearm	Distal Reading	Dominant	Right	Not measurable	Not measurable	Not measurable	Not measurable	
				Forearm	Proximal Reading	Dominant	Right	868	1127	315	2310	-142
01006	04-FEB-2019	Visit 1 (Screening)	Cross Sectional Muscle Area (mm2)	Forearm	Central Reading	Dominant	Right	625	1440	148	2213	
				Forearm	Distal Reading	Dominant	Right	313	686	Not measurable	999	
				Forearm	Proximal Reading	Dominant	Right	688	1181	208	2077	
	06-MAY-2019	Visit 8 (Week 12, Day 81)	Cross Sectional Muscle Area (mm2)	Forearm	Central Reading	Dominant	Right	581	1665	152	2398	185
				Forearm	Distal Reading	Dominant	Right	322	651	Not measurable	973	-26

Listing 16.4 Listing of MRI Cross Sectional Muscle Area (mm2)

Antisense Therapeutics Limited
Protocol: 1102-DMD-CT02
Population: All Enrolled Subjects (excludes Screen Failures)

Subject ID	Date	Visit	Measure	Location	Reading	Status	Arm Measured	Dorsal Muscles	Volar Muscle	ECRLB-Br	Total Area (mm2)	Change from Screening Total Area (mm2)
01006	06-MAY-2019	Visit 8 (Week 12, Day 81)	Cross Sectional Muscle Area (mm2)	Forearm	Proximal Reading	Dominant	Right	741	1233	319	2293	216
	08-JUL-2019	Visit 14 (Week 24, Day 165)	Cross Sectional Muscle Area (mm2)	Forearm	Central Reading	Dominant	Right	591	1508	146	2245	32
				Forearm	Distal Reading	Dominant	Right	327	696	Not measurable	1023	24
				Forearm	Proximal Reading	Dominant	Right	736	1266	282	2284	207
01008	25-FEB-2019	Visit 1 (Screening)	Cross Sectional Muscle Area (mm2)	Forearm	Central Reading	Dominant	Left	277	685	14	976	
				Forearm	Distal Reading	Dominant	Left	55	250	Not measurable	305	
				Forearm	Proximal Reading	Dominant	Left	407	891	140	1438	

Listing 16.4 Listing of MRI Cross Sectional Muscle Area (mm2)

Antisense Therapeutics Limited
Protocol: 1102-DMD-CT02
Population: All Enrolled Subjects (excludes Screen Failures)

Subject ID	Date	Visit	Measure	Location	Reading	Status	Arm Measured	Dorsal Muscles	Volar Muscle	ECRLB-Br	Total Area (mm2)	Change from Screening Total Area (mm2)
01008	20-MAY-2019	Visit 8 (Week 12, Day 81)	Cross Sectional Muscle Area (mm2)	Forearm	Central Reading	Dominant	Left	276	692	8	976	0
				Forearm	Distal Reading	Dominant	Left	Not measurable	Not measurable	Not measurable	Not measurable	
				Forearm	Proximal Reading	Dominant	Left	371	714	140	1225	-213
	12-AUG-2019	Visit 14 (Week 24, Day 165)	Cross Sectional Muscle Area (mm2)	Forearm	Central Reading	Dominant	Left	271	810	21	1102	126
				Forearm	Distal Reading	Dominant	Left	61	395	Not measurable	456	151
				Forearm	Proximal Reading	Dominant	Left	341	777	161	1279	-159
01009	15-APR-2019	Visit 1 (Screening)	Cross Sectional Muscle Area (mm2)	Forearm	Central Reading	Dominant	Left	486	817	58	1361	

Listing 16.4 Listing of MRI Cross Sectional Muscle Area (mm2)

Antisense Therapeutics Limited
Protocol: 1102-DMD-CT02
Population: All Enrolled Subjects (excludes Screen Failures)

Subject ID	Date	Visit	Measure	Location	Reading	Status	Arm Measured	Dorsal Muscles	Volar Muscle	ECRLB-Br	Total Area (mm2)	Change from Screening Total Area (mm2)
01009	15-APR-2019	Visit 1 (Screening)	Cross Sectional Muscle Area (mm2)	Forearm	Distal Reading	Dominant	Left	94	424	Not measurable	518	
				Forearm	Proximal Reading	Dominant	Left	510	1249	339	2098	
	15-JUL-2019	Visit 8 (Week 12, Day 81)	Cross Sectional Muscle Area (mm2)	Forearm	Central Reading	Dominant	Left	507	897	42	1446	85
				Forearm	Distal Reading	Dominant	Left	163	426	Not measurable	589	71
				Forearm	Proximal Reading	Dominant	Left	489	1327	316	2132	34
	30-SEP-2019	Visit 14 (Week 24, Day 165)	Cross Sectional Muscle Area (mm2)	Forearm	Central Reading	Dominant	Left	520	744	43	1307	-54
				Forearm	Distal Reading	Dominant	Left	Not measurable	Not measurable	Not measurable	Not measurable	

Listing 16.4 Listing of MRI Cross Sectional Muscle Area (mm2)

Antisense Therapeutics Limited
Protocol: 1102-DMD-CT02
Population: All Enrolled Subjects (excludes Screen Failures)

Subject ID	Date	Visit	Measure	Location	Reading	Status	Arm Measured	Dorsal Muscles	Volar Muscle	ECRLB-Br	Total Area (mm2)	Change from Screening Total Area (mm2)
01009	30-SEP-2019	Visit 14 (Week 24, Day 165)	Cross Sectional Muscle Area (mm2)	Forearm	Proximal Reading	Dominant	Left	493	1449	329	2271	173
01010	13-MAY-2019	Visit 1 (Screening)	Cross Sectional Muscle Area (mm2)	Forearm	Central Reading	Dominant	Right	237	424	95	756	
				Forearm	Distal Reading	Dominant	Right	265	326	Not measurable	591	
				Forearm	Proximal Reading	Dominant	Right	393	469	85	947	
	19-AUG-2019	Visit 8 (Week 12, Day 81)	Cross Sectional Muscle Area (mm2)	Forearm	Central Reading	Dominant	Right	245	419	94	758	2
				Forearm	Distal Reading	Dominant	Right	252	338	Not measurable	590	-1
				Forearm	Proximal Reading	Dominant	Right	402	472	82	956	9

Listing 16.4 Listing of MRI Cross Sectional Muscle Area (mm2)

Antisense Therapeutics Limited
Protocol: 1102-DMD-CT02
Population: All Enrolled Subjects (excludes Screen Failures)

Subject ID	Date	Visit	Measure	Location	Reading	Status	Arm Measured	Dorsal Muscles	Volar Muscle	ECRLB-Br	Total Area (mm2)	Change from Screening Total Area (mm2)
01010	04-NOV-2019	Visit 14 (Week 24, Day 165)	Cross Sectional Muscle Area (mm2)	Forearm	Central Reading	Dominant	Right	253	416	91	760	4
				Forearm	Distal Reading	Dominant	Right	262	345	Not measurable	607	16
				Forearm	Proximal Reading	Dominant	Right	396	491	84	971	24
01011	27-MAY-2019	Visit 1 (Screening)	Cross Sectional Muscle Area (mm2)	Forearm	Central Reading	Dominant	Right	357	789	32	1178	
				Forearm	Distal Reading	Dominant	Right	39	322	Not measurable	361	
				Forearm	Proximal Reading	Dominant	Right	503	923	205	1631	
	26-AUG-2019	Visit 8 (Week 12, Day 81)	Cross Sectional Muscle Area (mm2)	Forearm	Central Reading	Dominant	Right	325	754	25	1104	-74

Listing 16.4 Listing of MRI Cross Sectional Muscle Area (mm2)

Antisense Therapeutics Limited
Protocol: 1102-DMD-CT02
Population: All Enrolled Subjects (excludes Screen Failures)

Subject ID	Date	Visit	Measure	Location	Reading	Status	Arm Measured	Dorsal Muscles	Volar Muscle	ECRLB-Br	Total Area (mm2)	Change from Screening Total Area (mm2)
01011	26-AUG-2019	Visit 8 (Week 12, Day 81)	Cross Sectional Muscle Area (mm2)	Forearm	Distal Reading	Dominant	Right	32	286	Not measurable	318	-43
				Forearm	Proximal Reading	Dominant	Right	473	968	210	1651	20
	11-NOV-2019	Visit 14 (Week 24, Day 165)	Cross Sectional Muscle Area (mm2)	Forearm	Central Reading	Dominant	Right	338	763	28	1129	-49
				Forearm	Distal Reading	Dominant	Right	36	365	Not measurable	401	40
				Forearm	Proximal Reading	Dominant	Right	436	957	206	1599	-32

Listing 16.5 Listing of MRI Fat Fraction (%)

Antisense Therapeutics Limited
Protocol: 1102-DMD-CT02
Population: All Enrolled Subjects (excludes Screen Failures)

Subject ID	Date	Visit	Measure	Location	Reading	Status	Arm Measured	Dorsal Muscles	Volar Muscle	ECRLB-Br	Average Fat Fraction(%)	Change from Screening Average Fat Fraction(%)
01001	27-AUG-2018	Visit 1 (Screening)	Fat Fraction (%)	Forearm	Central Reading	Dominant	Right	14	11.6	10	11.9	
				Forearm	Distal Reading	Dominant	Right	14.1	16.4	Not measurable	15.3	
				Forearm	Proximal Reading	Dominant	Right	16.6	21.6	12.3	16.8	
	19-NOV-2018	Visit 8 (Week 12, Day 81)	Fat Fraction (%)	Forearm	Central Reading	Dominant	Right	13.7	12.9	10.4	12.3	0.4
				Forearm	Distal Reading	Dominant	Right	13	15.2	Not measurable	14.1	-1.2
				Forearm	Proximal Reading	Dominant	Right	15.1	23	12.5	16.9	0.1

Listing 16.5 Listing of MRI Fat Fraction (%)

Antisense Therapeutics Limited
Protocol: 1102-DMD-CT02
Population: All Enrolled Subjects (excludes Screen Failures)

Subject ID	Date	Visit	Measure	Location	Reading	Status	Arm Measured	Dorsal Muscles	Volar Muscle	ECRLB-Br	Average Fat Fraction(%)	Change from Screening Average Fat Fraction(%)
01001	18-FEB-2019	Visit 14 (Week 24, Day 165)	Fat Fraction (%)	Forearm	Central Reading	Dominant	Right	12.8	11.5	10.3	11.5	-0.4
				Forearm	Distal Reading	Dominant	Right	17	17.5	Not measurable	17.3	2
				Forearm	Proximal Reading	Dominant	Right	17.8	19.5	12	16.4	-0.4
01002	29-OCT-2018	Visit 1 (Screening)	Fat Fraction (%)	Forearm	Central Reading	Dominant	Right	41.1	64.7	41.5	49.1	
				Forearm	Distal Reading	Dominant	Right	48.5	56.4	Not measurable	52.5	
				Forearm	Proximal Reading	Dominant	Right	45.4	72.8	52.5	56.9	
	21-JAN-2019	Visit 8 (Week 12, Day 81)	Fat Fraction (%)	Forearm	Central Reading	Dominant	Right	50.5	73.1	47.7	57.1	8

Listing 16.5 Listing of MRI Fat Fraction (%)

Antisense Therapeutics Limited
Protocol: 1102-DMD-CT02
Population: All Enrolled Subjects (excludes Screen Failures)

Subject ID	Date	Visit	Measure	Location	Reading	Status	Arm Measured	Dorsal Muscles	Volar Muscle	ECRLB-Br	Average Fat Fraction(%)	Change from Screening Average Fat Fraction(%)
01002	21-JAN-2019	Visit 8 (Week 12, Day 81)	Fat Fraction (%)	Forearm	Distal Reading	Dominant	Right	53.2	62.1	Not measurable	57.7	5.2
				Forearm	Proximal Reading	Dominant	Right	56.6	73.7	57.6	62.6	5.7
	01-APR-2019	Visit 14 (Week 24, Day 165)	Fat Fraction (%)	Forearm	Central Reading	Dominant	Right	41.1	67.5	43.6	50.7	1.6
				Forearm	Distal Reading	Dominant	Right	51.7	61.7	Not measurable	56.7	4.2
				Forearm	Proximal Reading	Dominant	Right	50.5	70.6	48.3	56.5	-0.4
01003	12-NOV-2018	Visit 1 (Screening)	Fat Fraction (%)	Forearm	Central Reading	Dominant	Right	13.3	16.8	22.1	17.4	
				Forearm	Distal Reading	Dominant	Right	16.4	21.9	Not measurable	19.2	

Listing 16.5 Listing of MRI Fat Fraction (%)

Antisense Therapeutics Limited
Protocol: 1102-DMD-CT02
Population: All Enrolled Subjects (excludes Screen Failures)

Subject ID	Date	Visit	Measure	Location	Reading	Status	Arm Measured	Dorsal Muscles	Volar Muscle	ECRLB-Br	Average Fat Fraction(%)	Change from Screening Average Fat Fraction(%)
01003	12-NOV-2018	Visit 1 (Screening)	Fat Fraction (%)	Forearm	Proximal Reading	Dominant	Right	18.4	23.3	13.7	18.5	
	11-FEB-2019	Visit 8 (Week 12, Day 81)	Fat Fraction (%)	Forearm	Central Reading	Dominant	Right	13.8	16.6	20	16.8	-0.6
				Forearm	Distal Reading	Dominant	Right	13.4	17.7	Not measurable	15.6	-3.6
				Forearm	Proximal Reading	Dominant	Right	22	22.8	15	19.9	1.4
	29-APR-2019	Visit 14 (Week 24, Day 165)	Fat Fraction (%)	Forearm	Central Reading	Dominant	Right	13.4	19.1	25	19.2	1.8
				Forearm	Distal Reading	Dominant	Right	13.3	15.5	Not measurable	14.4	-4.8
				Forearm	Proximal Reading	Dominant	Right	26.4	18.3	19.9	21.5	3

Listing 16.5 Listing of MRI Fat Fraction (%)

Antisense Therapeutics Limited
Protocol: 1102-DMD-CT02
Population: All Enrolled Subjects (excludes Screen Failures)

Subject ID	Date	Visit	Measure	Location	Reading	Status	Arm Measured	Dorsal Muscles	Volar Muscle	ECRLB-Br	Average Fat Fraction(%)	Change from Screening Average Fat Fraction(%)
01004	14-JAN-2019	Visit 1 (Screening)	Fat Fraction (%)	Forearm	Central Reading	Dominant	Right	22.5	18.4	23	21.3	
				Forearm	Distal Reading	Dominant	Right	22	27.5	Not measurable	24.8	
				Forearm	Proximal Reading	Dominant	Right	25.9	24.6	21.3	23.9	
	08-APR-2019	Visit 8 (Week 12, Day 81)	Fat Fraction (%)	Forearm	Central Reading	Dominant	Right	23.6	17.5	32.9	24.7	3.4
				Forearm	Distal Reading	Dominant	Right	20.2	27.6	Not measurable	23.9	-0.9
				Forearm	Proximal Reading	Dominant	Right	19.6	30.7	19.9	23.4	-0.5
	01-JUL-2019	Visit 14 (Week 24, Day 165)	Fat Fraction (%)	Forearm	Central Reading	Dominant	Right	21.9	20.9	25.2	22.7	1.4

Listing 16.5 Listing of MRI Fat Fraction (%)

Antisense Therapeutics Limited
Protocol: 1102-DMD-CT02
Population: All Enrolled Subjects (excludes Screen Failures)

Subject ID	Date	Visit	Measure	Location	Reading	Status	Arm Measured	Dorsal Muscles	Volar Muscle	ECRLB-Br	Average Fat Fraction(%)	Change from Screening Average Fat Fraction(%)
01004	01-JUL-2019	Visit 14 (Week 24, Day 165)	Fat Fraction (%)	Forearm	Distal Reading	Dominant	Right	Not measurable	Not measurable	Not measurable	Not measurable	
				Forearm	Proximal Reading	Dominant	Right	18.9	24.5	17	20.1	-3.8
01006	04-FEB-2019	Visit 1 (Screening)	Fat Fraction (%)	Forearm	Central Reading	Dominant	Right	37.6	63.7	19.8	40.4	
				Forearm	Distal Reading	Dominant	Right	27.4	50.4	Not measurable	38.9	
				Forearm	Proximal Reading	Dominant	Right	34.3	58.8	57.5	50.2	
	06-MAY-2019	Visit 8 (Week 12, Day 81)	Fat Fraction (%)	Forearm	Central Reading	Dominant	Right	41.8	72.2	30.2	48.1	7.7
				Forearm	Distal Reading	Dominant	Right	37.9	54.1	Not measurable	46	7.1

Listing 16.5 Listing of MRI Fat Fraction (%)

Antisense Therapeutics Limited
Protocol: 1102-DMD-CT02
Population: All Enrolled Subjects (excludes Screen Failures)

Subject ID	Date	Visit	Measure	Location	Reading	Status	Arm Measured	Dorsal Muscles	Volar Muscle	ECRLB-Br	Average Fat Fraction(%)	Change from Screening Average Fat Fraction(%)
01006	06-MAY-2019	Visit 8 (Week 12, Day 81)	Fat Fraction (%)	Forearm	Proximal Reading	Dominant	Right	48.6	65.3	47.9	53.9	3.7
	08-JUL-2019	Visit 14 (Week 24, Day 165)	Fat Fraction (%)	Forearm	Central Reading	Dominant	Right	42.4	72.9	30.9	48.7	8.3
				Forearm	Distal Reading	Dominant	Right	41.4	56	Not measurable	48.7	9.8
				Forearm	Proximal Reading	Dominant	Right	44	69.2	65.2	59.5	9.3
01008	25-FEB-2019	Visit 1 (Screening)	Fat Fraction (%)	Forearm	Central Reading	Dominant	Left	12	8.9	14.4	11.8	
				Forearm	Distal Reading	Dominant	Left	49	33.9	Not measurable	41.5	
				Forearm	Proximal Reading	Dominant	Left	11.6	13.1	21	15.2	

Listing 16.5 Listing of MRI Fat Fraction (%)

Antisense Therapeutics Limited
Protocol: 1102-DMD-CT02
Population: All Enrolled Subjects (excludes Screen Failures)

Subject ID	Date	Visit	Measure	Location	Reading	Status	Arm Measured	Dorsal Muscles	Volar Muscle	ECRLB-Br	Average Fat Fraction(%)	Change from Screening Average Fat Fraction(%)
01008	20-MAY-2019	Visit 8 (Week 12, Day 81)	Fat Fraction (%)	Forearm	Central Reading	Dominant	Left	11.3	6.7	7.9	8.6	-3.2
				Forearm	Distal Reading	Dominant	Left	Not measurable	Not measurable	Not measurable	Not measurable	
				Forearm	Proximal Reading	Dominant	Left	5.9	8.6	4.6	6.4	-8.8
	12-AUG-2019	Visit 14 (Week 24, Day 165)	Fat Fraction (%)	Forearm	Central Reading	Dominant	Left	10.4	10.2	3.7	8.1	-3.7
				Forearm	Distal Reading	Dominant	Left	16.1	11.5	Not measurable	13.8	-27.7
				Forearm	Proximal Reading	Dominant	Left	7	16.7	6.8	10.2	-5
01009	15-APR-2019	Visit 1 (Screening)	Fat Fraction (%)	Forearm	Central Reading	Dominant	Left	15.6	23.3	10.1	16.3	

Listing 16.5 Listing of MRI Fat Fraction (%)

Antisense Therapeutics Limited
Protocol: 1102-DMD-CT02
Population: All Enrolled Subjects (excludes Screen Failures)

Subject ID	Date	Visit	Measure	Location	Reading	Status	Arm Measured	Dorsal Muscles	Volar Muscle	ECRLB-Br	Average Fat Fraction(%)	Change from Screening Average Fat Fraction(%)
01009	15-APR-2019	Visit 1 (Screening)	Fat Fraction (%)	Forearm	Distal Reading	Dominant	Left	19.1	16.9	Not measurable	18	
				Forearm	Proximal Reading	Dominant	Left	14.6	33.7	10.5	19.6	
	15-JUL-2019	Visit 8 (Week 12, Day 81)	Fat Fraction (%)	Forearm	Central Reading	Dominant	Left	15.8	24.7	9.9	16.8	0.5
				Forearm	Distal Reading	Dominant	Left	17.3	16.9	Not measurable	17.1	-0.9
				Forearm	Proximal Reading	Dominant	Left	17	33.4	13.1	21.2	1.6
	30-SEP-2019	Visit 14 (Week 24, Day 165)	Fat Fraction (%)	Forearm	Central Reading	Dominant	Left	13.7	18.5	7.8	13.3	-3
				Forearm	Distal Reading	Dominant	Left	Not measurable	Not measurable	Not measurable	Not measurable	

Listing 16.5 Listing of MRI Fat Fraction (%)

Antisense Therapeutics Limited
Protocol: 1102-DMD-CT02
Population: All Enrolled Subjects (excludes Screen Failures)

Subject ID	Date	Visit	Measure	Location	Reading	Status	Arm Measured	Dorsal Muscles	Volar Muscle	ECRLB-Br	Average Fat Fraction(%)	Change from Screening Average Fat Fraction(%)
01009	30-SEP-2019	Visit 14 (Week 24, Day 165)	Fat Fraction (%)	Forearm	Proximal Reading	Dominant	Left	14.3	32.3	11.3	19.3	-0.3
01010	13-MAY-2019	Visit 1 (Screening)	Fat Fraction (%)	Forearm	Central Reading	Dominant	Right	49.6	56.9	27.7	44.7	
				Forearm	Distal Reading	Dominant	Right	40.9	51.9	Not measurable	46.4	
				Forearm	Proximal Reading	Dominant	Right	45.1	68.1	57.4	56.9	
	19-AUG-2019	Visit 8 (Week 12, Day 81)	Fat Fraction (%)	Forearm	Central Reading	Dominant	Right	44.9	57.4	31.6	44.6	-0.1
				Forearm	Distal Reading	Dominant	Right	42.9	53.9	Not measurable	48.4	2
				Forearm	Proximal Reading	Dominant	Right	44.8	69.4	55.5	56.6	-0.3

Listing 16.5 Listing of MRI Fat Fraction (%)

Antisense Therapeutics Limited
Protocol: 1102-DMD-CT02
Population: All Enrolled Subjects (excludes Screen Failures)

Subject ID	Date	Visit	Measure	Location	Reading	Status	Arm Measured	Dorsal Muscles	Volar Muscle	ECRLB-Br	Average Fat Fraction(%)	Change from Screening Average Fat Fraction(%)
01010	04-NOV-2019	Visit 14 (Week 24, Day 165)	Fat Fraction (%)	Forearm	Central Reading	Dominant	Right	50	62.2	35.6	49.3	4.6
				Forearm	Distal Reading	Dominant	Right	46.4	54.1	Not measurable	50.3	3.9
				Forearm	Proximal Reading	Dominant	Right	45.5	55.4	55.4	52.1	-4.8
01011	27-MAY-2019	Visit 1 (Screening)	Fat Fraction (%)	Forearm	Central Reading	Dominant	Right	24.6	46.4	28.4	33.1	
				Forearm	Distal Reading	Dominant	Right	69	58.8	Not measurable	42.6	
				Forearm	Proximal Reading	Dominant	Right	38.8	57.6	16.9	37.8	
	26-AUG-2019	Visit 8 (Week 12, Day 81)	Fat Fraction (%)	Forearm	Central Reading	Dominant	Right	16.3	20.1	15.6	17.3	-15.8

Listing 16.5 Listing of MRI Fat Fraction (%)

Antisense Therapeutics Limited
Protocol: 1102-DMD-CT02
Population: All Enrolled Subjects (excludes Screen Failures)

Subject ID	Date	Visit	Measure	Location	Reading	Status	Arm Measured	Dorsal Muscles	Volar Muscle	ECRLB-Br	Average Fat Fraction(%)	Change from Screening Average Fat Fraction(%)
01011	26-AUG-2019	Visit 8 (Week 12, Day 81)	Fat Fraction (%)	Forearm	Distal Reading	Dominant	Right	31.7	26.3	Not measurable	19.3	-23.3
				Forearm	Proximal Reading	Dominant	Right	20.4	30.1	11.4	20.6	-17.2
	11-NOV-2019	Visit 14 (Week 24, Day 165)	Fat Fraction (%)	Forearm	Central Reading	Dominant	Right	16.7	22.8	13.8	17.8	-15.3
				Forearm	Distal Reading	Dominant	Right	24.8	32.8	Not measurable	19.2	-23.4
				Forearm	Proximal Reading	Dominant	Right	18.1	29.1	15.4	20.9	-16.9

Listing 16.7 Listing of Total Fat and Lean Muscle Area - By Muscle

Antisense Therapeutics Limited
Protocol: 1102-DMD-CT02
Population: All Enrolled Subjects (excludes Screen Failures)

				|-----Dorsal Muscle CSA (mm²)------|				|-----Volar Muscle CSA (mm²)-------|				|--ECRLB-Br Muscle CSA (mm²)--|				|Total Muscle CSA (mm²)|		
Subject ID	Date	Week	Reading	Fat	Lean Muscle	Total	Change** Lean Muscle	Fat	Lean Muscle	Total	Change** Lean Muscle	Fat	Lean Muscle	Total	Change** Lean Muscle	Fat	Lean Muscle	Total
01001	27-AUG-2018	0	Central	58.4	358.6	417		116.0	884.0	1000		10.8	97.2	108		185.2	1339.8	1525
			Distal	14.8	90.2	105		61.8	315.2	377						76.6	405.4	482
			Proximal	61.6	309.4	371		152.3	552.7	705		32.0	228.0	260		245.8	1090.2	1336
	19-NOV-2018	12	Central	56.2	353.8	410	-4.8	137.6	929.4	1067	45.4	10.9	94.1	105	-3.1	204.7	1377.3	1582
			Distal	15.1	100.9	116	10.7	52.9	295.1	348	-20.1					68.0	396.0	464
			Proximal	58.1	326.9	385	17.5	160.5	537.5	698	-15.3	31.0	217.0	248	-11.0	249.7	1081.3	1331
	18-FEB-2019	24	Central	53.9	367.1	421	8.5	125.9	969.1	1095	85.1	10.3	89.7	100	-7.5	190.1	1425.9	1616
			Distal	20.9	102.1	123	11.9	56.4	265.7	322	-49.5					77.3	367.7	445
			Proximal	68.7	317.3	386	7.9	139.4	575.6	715	22.9	33.0	242.0	275	14.0	241.1	1134.9	1376

CSA = Cross Sectional Area
Fat Area(mm²)=(Muscle Cross Sectional Area x Muscle Fat Fraction)
Reading: C=Central, D=Distal, P=Proximal
Lean Muscle Area(mm²)=Total Area - Fat Area
Change** Lean Muscle: Change from Baseline of Lean Muscle Area

Listing 16.7 Listing of Total Fat and Lean Muscle Area - By Muscle

Antisense Therapeutics Limited
Protocol: 1102-DMD-CT02
Population: All Enrolled Subjects (excludes Screen Failures)

				Dorsal Muscle CSA (mm²)				Volar Muscle CSA (mm²)				ECRLB-Br Muscle CSA (mm²)				Total Muscle CSA (mm²)		
Subject ID	Date	Week	Reading	Fat	Lean Muscle	Total	Change** Lean Muscle	Fat	Lean Muscle	Total	Change** Lean Muscle	Fat	Lean Muscle	Total	Change** Lean Muscle	Fat	Lean Muscle	Total
01002	29-OCT-2018	0	Central	198.1	283.9	482		497.5	271.5	769		85.9	121.1	207		781.6	676.5	1458
			Distal	84.4	89.6	174		253.2	195.8	449						337.6	285.4	623
			Proximal	185.7	223.3	409		530.7	198.3	729		87.2	78.9	166		803.5	500.5	1304
	21-JAN-2019	12	Central	236.3	231.7	468	-52.2	549.0	202.0	751	-69.4	101.6	111.4	213	-9.7	886.9	545.1	1432
			Distal	90.4	79.6	170	-10.0	264.5	161.5	426	-34.3					355.0	241.0	596
			Proximal	220.2	168.8	389	-54.5	594.0	212.0	806	13.7	115.2	84.8	200	5.9	929.4	465.6	1395
	01-APR-2019	24	Central	196.9	282.1	479	-1.8	529.9	255.1	785	-16.3	99.0	128.0	227	6.9	825.7	665.3	1491
			Distal	94.1	87.9	182	-1.7	274.6	170.4	445	-25.3					368.7	258.3	627
			Proximal	201.5	197.5	399	-25.8	535.1	222.9	758	24.6	95.2	101.8	197	23.0	831.8	522.2	1354

CSA = Cross Sectional Area
Fat Area(mm²)=(Muscle Cross Sectional Area x Muscle Fat Fraction)
Reading: C=Central, D=Distal, P=Proximal
Lean Muscle Area(mm²)=Total Area - Fat Area
Change** Lean Muscle: Change from Baseline of Lean Muscle Area

Listing 16.7 Listing of Total Fat and Lean Muscle Area - By Muscle

Antisense Therapeutics Limited
Protocol: 1102-DMD-CT02
Population: All Enrolled Subjects (excludes Screen Failures)

				|-----Dorsal Muscle CSA (mm²)------|				|-----Volar Muscle CSA (mm²)-------|				|--ECRLB-Br Muscle CSA (mm²)--|				|Total Muscle CSA (mm²)|		
Subject ID	Date	Week	Reading	Fat	Lean Muscle	Total	Change** Lean Muscle	Fat	Lean Muscle	Total	Change** Lean Muscle	Fat	Lean Muscle	Total	Change** Lean Muscle	Fat	Lean Muscle	Total
01003	12-NOV-2018	0	Central	65.0	424.0	489		112.7	558.3	671		13.0	46.0	59		190.8	1028.2	1219
			Distal	42.5	216.5	259		82.8	295.2	378						125.3	511.7	637
			Proximal	96.4	427.6	524		216.2	711.8	928		24.5	154.5	179		337.2	1293.8	1631
	11-FEB-2019	12	Central	66.2	413.8	480	-10.2	119.9	602.1	722	43.9	12.0	48.0	60	2.0	198.1	1063.9	1262
			Distal	35.9	232.1	268	15.6	67.1	311.9	379	16.7					103.0	544.0	647
			Proximal	104.9	372.1	477	-55.5	203.1	687.9	891	-23.9	26.9	152.2	179	-2.3	334.9	1212.1	1547
	29-APR-2019	24	Central	71.0	459.0	530	35.0	141.3	598.7	740	40.4	14.5	43.5	58	-2.5	226.9	1101.1	1328
			Distal	39.4	256.6	296	40.1	53.6	292.4	346	-2.8					93.0	549.0	642
			Proximal	208.3	580.7	789	153.1	82.9	370.1	453	-341.7	35.0	141.0	176	-13.5	326.2	1091.8	1418

CSA = Cross Sectional Area
Fat Area(mm²)=(Muscle Cross Sectional Area x Muscle Fat Fraction)
Reading: C=Central, D=Distal, P=Proximal
Lean Muscle Area(mm²)=Total Area - Fat Area
Change** Lean Muscle: Change from Baseline of Lean Muscle Area

Listing 16.7 Listing of Total Fat and Lean Muscle Area - By Muscle

Antisense Therapeutics Limited
Protocol: 1102-DMD-CT02
Population: All Enrolled Subjects (excludes Screen Failures)

				|-----Dorsal Muscle CSA (mm²)------|				|-----Volar Muscle CSA (mm²)-------|				|--ECRLB-Br Muscle CSA (mm²)--|				|Total Muscle CSA (mm²)|	
Subject ID	Date	Week	Reading	Fat	Lean Muscle	Total	Change** Lean Muscle	Fat	Lean Muscle	Total	Change** Lean Muscle	Fat	Lean Muscle	Total	Change** Lean Muscle	Fat	Lean Muscle	Total
01004	14-JAN-2019	0	Central	186.5	642.5	829		284.8	1263	1548		39.8	133.2	173		511.1	2038.9	2550
			Distal	55.4	196.6	252		161.2	424.9	586						216.6	621.4	838
			Proximal	267.0	764.0	1031		272.1	833.9	1106		67.1	247.9	315		606.2	1845.8	2452
	08-APR-2019	12	Central	194.7	630.3	825	-12.2	262.2	1236	1498	-27.3	57.2	116.8	174	-16.5	514.1	1982.9	2497
			Distal	46.1	181.9	228	-14.6	153.7	403.3	557	-21.6					199.8	585.2	785
			Proximal	181.5	744.5	926	-19.5	338.9	765.1	1104	-68.9	62.1	249.9	312	2.0	582.5	1759.5	2342
	01-JUL-2019	24	Central	176.1	627.9	804	-14.6	310.8	1176	1487	-87.0	42.3	125.7	168	-7.5	529.2	1929.8	2459
			Distal															
			Proximal	164.1	703.9	868	-60.0	276.1	850.9	1127	17.0	53.6	261.5	315	13.5	493.7	1816.3	2310

CSA = Cross Sectional Area
Fat Area(mm²)=(Muscle Cross Sectional Area x Muscle Fat Fraction)
Reading: C=Central, D=Distal, P=Proximal
Lean Muscle Area(mm²)=Total Area - Fat Area
Change** Lean Muscle: Change from Baseline of Lean Muscle Area

Listing 16.7 Listing of Total Fat and Lean Muscle Area - By Muscle

Antisense Therapeutics Limited
Protocol: 1102-DMD-CT02
Population: All Enrolled Subjects (excludes Screen Failures)

				Dorsal Muscle CSA (mm²)				Volar Muscle CSA (mm²)				ECRLB-Br Muscle CSA (mm²)				Total Muscle CSA (mm²)		
Subject ID	Date	Week	Reading	Fat	Lean Muscle	Total	Change** Lean Muscle	Fat	Lean Muscle	Total	Change** Lean Muscle	Fat	Lean Muscle	Total	Change** Lean Muscle	Fat	Lean Muscle	Total
01006	04-FEB-2019	0	Central	235.0	390.0	625		917.3	522.7	1440		29.3	118.7	148		1181.6	1031.4	2213
			Distal	85.8	227.2	313		345.7	340.3	686						431.5	567.5	999
			Proximal	236.0	452.0	688		694.4	486.6	1181		119.6	88.4	208		1050.0	1027.0	2077
	06-MAY-2019	12	Central	242.9	338.1	581	-51.9	1202	462.9	1665	-59.8	45.9	106.1	152	-12.6	1490.9	907.1	2398
			Distal	122.0	200.0	322	-27.3	352.2	298.8	651	-41.4					474.2	498.8	973
			Proximal	360.1	380.9	741	-71.1	805.1	427.9	1233	-58.7	152.8	166.2	319	77.8	1318.1	974.9	2293
	08-JUL-2019	24	Central	250.6	340.4	591	-49.6	1099	408.7	1508	-114.1	45.1	100.9	146	-17.8	1395.0	850.0	2245
			Distal	135.4	191.6	327	-35.6	389.8	306.2	696	-34.0					525.1	497.9	1023
			Proximal	323.8	412.2	736	-39.9	876.1	389.9	1266	-96.6	183.9	98.1	282	9.7	1383.8	900.2	2284

CSA = Cross Sectional Area
Fat Area(mm²)=(Muscle Cross Sectional Area x Muscle Fat Fraction)
Reading: C=Central, D=Distal, P=Proximal
Lean Muscle Area(mm²)=Total Area - Fat Area
Change** Lean Muscle: Change from Baseline of Lean Muscle Area

Listing 16.7 Listing of Total Fat and Lean Muscle Area - By Muscle

Antisense Therapeutics Limited
Protocol: 1102-DMD-CT02
Population: All Enrolled Subjects (excludes Screen Failures)

				|-----Dorsal Muscle CSA (mm²)------|				|-----Volar Muscle CSA (mm²)-------|				|--ECRLB-Br Muscle CSA (mm²)--|				|Total Muscle CSA (mm²)|	
Subject ID	Date	Week	Reading	Fat	Lean Muscle	Total	Change** Lean Muscle	Fat	Lean Muscle	Total	Change** Lean Muscle	Fat	Lean Muscle	Total	Change** Lean Muscle	Fat	Lean Muscle	Total
01008	25-FEB-2019	0	Central	33.2	243.8	277		61.0	624.0	685		2.0	12.0	14		96.2	879.8	976
			Distal	27.0	28.1	55		84.7	165.3	250						111.7	193.3	305
			Proximal	47.2	359.8	407		116.7	774.3	891		29.4	110.6	140		193.3	1244.7	1438
	20-MAY-2019	12	Central	31.2	244.8	276	1.1	46.4	645.6	692	21.6	0.6	7.4	8	-4.6	78.2	897.8	976
			Distal															
			Proximal	21.9	349.1	371	-10.7	61.4	652.6	714	-121.7	6.4	133.6	140	23.0	89.7	1135.3	1225
	12-AUG-2019	24	Central	28.2	242.8	271	-0.9	82.6	727.4	810	103.3	0.8	20.2	21	8.2	111.6	990.4	1102
			Distal	9.8	51.2	61	23.1	45.4	349.6	395	184.3					55.2	400.8	456
			Proximal	23.9	317.1	341	-42.7	129.8	647.2	777	-127.0	10.9	150.1	161	39.5	164.6	1114.4	1279

CSA = Cross Sectional Area
Fat Area(mm²)=(Muscle Cross Sectional Area x Muscle Fat Fraction)
Reading: C=Central, D=Distal, P=Proximal
Lean Muscle Area(mm²)=Total Area - Fat Area
Change** Lean Muscle: Change from Baseline of Lean Muscle Area

Listing 16.7 Listing of Total Fat and Lean Muscle Area - By Muscle

Antisense Therapeutics Limited
Protocol: 1102-DMD-CT02
Population: All Enrolled Subjects (excludes Screen Failures)

				Dorsal Muscle CSA (mm²)				Volar Muscle CSA (mm²)				ECRLB-Br Muscle CSA (mm²)				Total Muscle CSA (mm²)		
Subject ID	Date	Week	Reading	Fat	Lean Muscle	Total	Change** Lean Muscle	Fat	Lean Muscle	Total	Change** Lean Muscle	Fat	Lean Muscle	Total	Change** Lean Muscle	Fat	Lean Muscle	Total
01009	15-APR-2019	0	Central	75.8	410.2	486		190.4	626.6	817		5.9	52.1	58		272.0	1089.0	1361
			Distal	18.0	76.0	94		71.7	352.3	424						89.6	428.4	518
			Proximal	74.5	435.5	510		420.9	828.1	1249		35.6	303.4	339		531.0	1567.0	2098
	15-JUL-2019	12	Central	80.1	426.9	507	16.7	221.6	675.4	897	48.8	4.2	37.8	42	-14.3	305.8	1140.2	1446
			Distal	28.2	134.8	163	58.8	72.0	354.0	426	1.7					100.2	488.8	589
			Proximal	83.1	405.9	489	-29.7	443.2	883.8	1327	55.7	41.4	274.6	316	-28.8	567.7	1564.3	2132
	30-SEP-2019	24	Central	71.2	448.8	520	38.6	137.6	606.4	744	-20.3	3.4	39.6	43	-12.5	212.2	1094.8	1307
			Distal															
			Proximal	70.5	422.5	493	-13.0	468.0	981.0	1449	152.9	37.2	291.8	329	-11.6	575.7	1695.3	2271

CSA = Cross Sectional Area
Fat Area(mm²)=(Muscle Cross Sectional Area x Muscle Fat Fraction)
Reading: C=Central, D=Distal, P=Proximal
Lean Muscle Area(mm²)=Total Area - Fat Area
Change** Lean Muscle: Change from Baseline of Lean Muscle Area

Listing 16.7 Listing of Total Fat and Lean Muscle Area - By Muscle

Antisense Therapeutics Limited
Protocol: 1102-DMD-CT02
Population: All Enrolled Subjects (excludes Screen Failures)

				Dorsal Muscle CSA (mm²)				Volar Muscle CSA (mm²)				ECRLB-Br Muscle CSA (mm²)				Total Muscle CSA (mm²)		
Subject ID	Date	Week	Reading	Fat	Lean Muscle	Total	Change** Lean Muscle	Fat	Lean Muscle	Total	Change** Lean Muscle	Fat	Lean Muscle	Total	Change** Lean Muscle	Fat	Lean Muscle	Total
01010	13-MAY-2019	0	Central	117.6	119.4	237		241.3	182.7	424		26.3	68.7	95		385.1	370.9	756
			Distal	108.4	156.6	265		169.2	156.8	326						277.6	313.4	591
			Proximal	177.2	215.8	393		319.4	149.6	469		48.8	36.2	85		545.4	401.6	947
	19-AUG-2019	12	Central	110.0	135.0	245	15.5	240.5	178.5	419	-4.3	29.7	64.3	94	-4.4	380.2	377.8	758
			Distal	108.1	143.9	252	-12.7	182.2	155.8	338	-1.0					290.3	299.7	590
			Proximal	180.1	221.9	402	6.1	327.6	144.4	472	-5.2	45.5	36.5	82	0.3	553.2	402.8	956
	04-NOV-2019	24	Central	126.5	126.5	253	7.1	258.8	157.2	416	-25.5	32.4	58.6	91	-10.1	417.6	342.4	760
			Distal	121.6	140.4	262	-16.2	186.6	158.4	345	1.5					308.2	298.8	607
			Proximal	180.2	215.8	396	0.1	272.0	219.0	491	69.4	46.5	37.5	84	1.3	498.7	472.3	971

CSA = Cross Sectional Area
Fat Area(mm²)=(Muscle Cross Sectional Area x Muscle Fat Fraction)
Reading: C=Central, D=Distal, P=Proximal
Lean Muscle Area(mm²)=Total Area - Fat Area
Change** Lean Muscle: Change from Baseline of Lean Muscle Area

Listing 16.7 Listing of Total Fat and Lean Muscle Area - By Muscle

Antisense Therapeutics Limited
Protocol: 1102-DMD-CT02
Population: All Enrolled Subjects (excludes Screen Failures)

				|-----Dorsal Muscle CSA (mm²)------|				|-----Volar Muscle CSA (mm²)-------|				|--ECRLB-Br Muscle CSA (mm²)--|				|Total Muscle CSA (mm²)|	
Subject ID	Date	Week	Reading	Fat	Lean Muscle	Total	Change** Lean Muscle	Fat	Lean Muscle	Total	Change** Lean Muscle	Fat	Lean Muscle	Total	Change** Lean Muscle	Fat	Lean Muscle	Total
01011	27-MAY-2019	0	Central	87.8	269.2	357		366.1	422.9	789		9.1	22.9	32		463.0	715.0	1178
			Distal	26.9	12.1	39		189.3	132.7	322						216.2	144.8	361
			Proximal	195.2	307.8	503		531.6	391.4	923		34.6	170.4	205		761.5	869.5	1631
	26-AUG-2019	12	Central	53.0	272.0	325	2.8	151.6	602.4	754	179.5	3.9	21.1	25	-1.8	208.4	895.6	1104
			Distal	10.1	21.9	32	9.8	75.2	210.8	286	78.1					85.4	232.6	318
			Proximal	96.5	376.5	473	68.7	291.4	676.6	968	285.3	23.9	186.1	210	15.7	411.8	1239.2	1651
	11-NOV-2019	24	Central	56.4	281.6	338	12.4	174.0	589.0	763	166.1	3.9	24.1	28	1.2	234.3	894.7	1129
			Distal	8.9	27.1	36	15.0	119.7	245.3	365	112.6					128.6	272.4	401
			Proximal	78.9	357.1	436	49.2	278.5	678.5	957	287.2	31.7	174.3	206	3.9	389.1	1209.9	1599

CSA = Cross Sectional Area
Fat Area(mm²)=(Muscle Cross Sectional Area x Muscle Fat Fraction)
Reading: C=Central, D=Distal, P=Proximal
Lean Muscle Area(mm²)=Total Area - Fat Area
Change** Lean Muscle: Change from Baseline of Lean Muscle Area

Table 1.1 Summary of Demographics - Gender, Race and Ethnicity

Antisense Therapeutics Limited
Protocol: 1102-DMD-CT02
Population: All Enrolled Subjects (excludes Screen Failures)(N=9)

Characteristic	Category	n(%)
Gender	Male	9 (100%)
Race	Native Hawaiian or Other Pacific Islander	1 (11.1%)
	White	8 (88.9%)
Ethnic	Non-Hispanic and Non-Latino	9 (100%)

Table 1.1.2 Summary of Demographics - Age, Weight, Height and BMI

Antisense Therapeutics Limited
Protocol: 1102-DMD-CT02
Population: All Enrolled Subjects (excludes Screen Failures)(N=9)

	Parameter			
Statistic	Age (years)	Weight (kg)	Height (cm)	BMI
n	9	9	9	9
Missing	0	0	0	0
Mean	14.9	52.7	141.1	27.1
STD	2.1	9.8	10.0	7.4
Minimum	12	39	129	15
Median	14.0	52.0	144.0	28.5
Maximum	18	64	160	37

Table 3.1 Adverse Events - Overall Summary

Antisense Therapeutics Limited
Protocol: 1102-DMD-CT02
Population: All Enrolled Subjects (excludes Screen Failures)

	All Subjects N=9
Number of Subjects with TEAEs	9 (100.0%)
Number of Subjects with Related TEAEs	9 (100.0%)
Number of Subjects with at least Moderate TEAEs	2 (22.2%)
Number of Subjects with Related and at least Moderate TEAEs	2 (22.2%)
Number of Subjects with SAEs	0 (0%)
Number of Subjects with AEs leading to withdrawal	0 (0%)
Number of Subjects Who Died	0 (0%)
Number of TEAEs	136
Number of Related TEAEs	114
Number of at least Moderate TEAEs	5
Number of Related and at least Moderate TEAEs	3
Number of SAEs	0
Number of AEs leading to withdrawal	0
Number of Deaths	0

Related*: includes those recorded as Possible, Probably, Definitely, or Unlikely
Not Related*: includes those recorded as Not Related

Table 3.2 Summary of Adverse Events

Antisense Therapeutics Limited
Protocol: 1102-DMD-CT02
Population: All Enrolled Subjects (excludes Screen Failures)

SYSTEM ORGAN CLASS/ Preferred Term	All N=9 Subjects (%) [Events]
Subjects reporting any TEAE in this table	9 (100.0%) [136]
GENERAL DISORDERS AND ADMINISTRATION SITE CONDITIONS	9 (100.0%) [86]
Injection site erythema	8 (88.9%) [59]
Injection site pain	5 (55.6%) [7]
Injection site swelling	3 (33.3%) [6]
Injection site bruising	4 (44.4%) [4]
Pyrexia	2 (22.2%) [4]
Injection site reaction	2 (22.2%) [3]
Injection site pruritus	1 (11.1%) [1]
Malaise	1 (11.1%) [1]
Peripheral swelling	1 (11.1%) [1]
GASTROINTESTINAL DISORDERS	6 (66.7%) [9]
Vomiting	2 (22.2%) [4]
Constipation	2 (22.2%) [2]
Diarrhoea	1 (11.1%) [1]
Gastrooesophageal reflux disease	1 (11.1%) [1]

Investigator text for Adverse Events encoded using MedDRA (Version 21.0). N = number of subjects; % = percentage of subjects in the analysis set.
A subject can have one or more preferred term reported under a given system organ class. A subject is only counted once per system organ class.

Table 3.2 Summary of Adverse Events

Antisense Therapeutics Limited
Protocol: 1102-DMD-CT02
Population: All Enrolled Subjects (excludes Screen Failures)

SYSTEM ORGAN CLASS/<br>Preferred Term	All<br>N=9<br>Subjects (%) [Events]
Malpositioned teeth	1 (11.1%) [1]
SKIN AND SUBCUTANEOUS TISSUE DISORDERS	6 (66.7%) [9]
Skin discolouration	6 (66.7%) [7]
Erythema	1 (11.1%) [1]
Pruritus	1 (11.1%) [1]
RESPIRATORY, THORACIC AND MEDIASTINAL DISORDERS	5 (55.6%) [8]
Cough	2 (22.2%) [2]
Nasal congestion	2 (22.2%) [2]
Oropharyngeal pain	2 (22.2%) [2]
Hyperventilation	1 (11.1%) [1]
Rhinorrhoea	1 (11.1%) [1]
INFECTIONS AND INFESTATIONS	5 (55.6%) [6]
Lower respiratory tract infection	2 (22.2%) [2]
Nasopharyngitis	2 (22.2%) [2]
Ear infection	1 (11.1%) [1]
Viral upper respiratory tract infection	1 (11.1%) [1]

Investigator text for Adverse Events encoded using MedDRA (Version 21.0). N = number of subjects; % = percentage of subjects in the analysis set.
A subject can have one or more preferred term reported under a given system organ class. A subject is only counted once per system organ class.

Table 3.2 Summary of Adverse Events

Antisense Therapeutics Limited
Protocol: 1102-DMD-CT02
Population: All Enrolled Subjects (excludes Screen Failures)

SYSTEM ORGAN CLASS/ Preferred Term	All N=9 Subjects (%) [Events]
NERVOUS SYSTEM DISORDERS	4 (44.4%) [5]
Migraine	2 (22.2%) [2]
Dizziness	1 (11.1%) [1]
Headache	1 (11.1%) [1]
Lethargy	1 (11.1%) [1]
INVESTIGATIONS	3 (33.3%) [4]
C-reactive protein increased	1 (11.1%) [1]
Colonoscopy	1 (11.1%) [1]
Electrocardiogram QT prolonged	1 (11.1%) [1]
Haptoglobin abnormal	1 (11.1%) [1]
PSYCHIATRIC DISORDERS	2 (22.2%) [3]
Anxiety	1 (11.1%) [1]
Insomnia	1 (11.1%) [1]
Restlessness	1 (11.1%) [1]

Investigator text for Adverse Events encoded using MedDRA (Version 21.0). N = number of subjects; % = percentage of subjects in the analysis set.
A subject can have one or more preferred term reported under a given system organ class. A subject is only counted once per system organ class.

Table 3.2 Summary of Adverse Events

Antisense Therapeutics Limited
Protocol: 1102-DMD-CT02
Population: All Enrolled Subjects (excludes Screen Failures)

SYSTEM ORGAN CLASS/ Preferred Term	All N=9 Subjects (%) [Events]
MUSCULOSKELETAL AND CONNECTIVE TISSUE DISORDERS	1 (11.1%) [2]
Muscle spasms	1 (11.1%) [1]
Pain in extremity	1 (11.1%) [1]
CARDIAC DISORDERS	1 (11.1%) [1]
Palpitations	1 (11.1%) [1]
METABOLISM AND NUTRITION DISORDERS	1 (11.1%) [1]
Decreased appetite	1 (11.1%) [1]
SURGICAL AND MEDICAL PROCEDURES	1 (11.1%) [1]
Tenotomy	1 (11.1%) [1]
VASCULAR DISORDERS	1 (11.1%) [1]
Hypotension	1 (11.1%) [1]

Investigator text for Adverse Events encoded using MedDRA (Version 21.0). N = number of subjects; % = percentage of subjects in the analysis set.
A subject can have one or more preferred term reported under a given system organ class. A subject is only counted once per system organ class.

Table 7.1 Summary of PUL2.0 Total Score Entry Score 0 to 6

Antisense Therapeutics Limited
Protocol: 1102-DMD-CT02
Population: All Enrolled Subjects (excludes Screen Failures) (N=9)

		Actual Values							Change From Baseline (Week 1, Day 1)							
Measure	Visit	n	Miss.	Mean	Std.	Min.	Median	Max.	n	Miss.	Mean	95% CI (for Mean)	Std.	Min.	Median	Max.
Total PUL 2.0 Score	Visit 2 (Week 1, Day 1)	9	0	24.78	9.64	5	27.0	37								
	Visit 4 (Week 5, Day 29)	9	0	25.56	10.30	4	28.0	35	9	0	0.78	(-0.80, 2.35)	2.05	-2.0	0.0	4.0
	Visit 6 (Week 8, Day 53)	9	0	25.89	10.06	4	29.0	38	9	0	1.11	(-0.13, 2.35)	1.62	-1.0	1.0	4.0
	Visit 8 (Week 12, Day 81)	9	0	26.22	9.90	4	28.0	35	9	0	1.44	(-0.32, 3.21)	2.30	-2.0	2.0	4.0
	Visit 14 (Week 24, Day 165)	9	0	25.67	9.66	5	29.0	37	9	0	0.89	(-1.33, 3.11)	2.89	-3.0	0.0	7.0
High Level Shoulder Dimension Score	Visit 2 (Week 1, Day 1)	7	2	4.71	4.46	0	5.0	11								
	Visit 4 (Week 5, Day 29)	7	2	6.00	3.37	1	5.0	10	7	2	1.29	(-0.29, 2.86)	1.70	-1.0	1.0	4.0
	Visit 6 (Week 8, Day 53)	7	2	5.71	3.04	1	6.0	10	7	2	1.00	(-0.77, 2.77)	1.91	-1.0	1.0	4.0
	Visit 8 (Week 12, Day 81)	7	2	5.57	2.76	2	5.0	9	7	2	0.86	(-1.10, 2.81)	2.12	-3.0	1.0	3.0
	Visit 14 (Week 24, Day 165)	7	2	5.57	3.05	1	5.0	10	7	2	0.86	(-1.61, 3.33)	2.67	-2.0	0.0	5.0

Miss. = Missing (subject attends a visit but measure not recorded)

Table 7.1 Summary of PUL2.0 Total Score Entry Score 0 to 6

Antisense Therapeutics Limited
Protocol: 1102-DMD-CT02
Population: All Enrolled Subjects (excludes Screen Failures) (N=9)

		Actual Values							Change From Baseline (Week 1, Day 1)							
Measure	Visit	n	Miss.	Mean	Std.	Min.	Median	Max.	n	Miss.	Mean	95% CI (for Mean)	Std.	Min.	Median	Max.
Mid Level Elbow Dimension Score	Visit 2 (Week 1, Day 1)	9	0	10.78	5.02	0	12.0	16								
	Visit 4 (Week 5, Day 29)	9	0	10.44	4.93	0	13.0	14	9	0	-0.33	(-1.00, 0.33)	0.87	-2.0	0.0	1.0
	Visit 6 (Week 8, Day 53)	9	0	11.22	4.94	0	13.0	17	9	0	0.44	(-0.42, 1.31)	1.13	-2.0	1.0	2.0
	Visit 8 (Week 12, Day 81)	9	0	11.33	4.97	0	13.0	16	9	0	0.56	(-0.31, 1.42)	1.13	-1.0	1.0	2.0
	Visit 14 (Week 24, Day 165)	9	0	10.89	4.81	0	12.0	16	9	0	0.11	(-0.86, 1.09)	1.27	-1.0	0.0	2.0
Distal Wrist and Hand Dimension Score	Visit 2 (Week 1, Day 1)	9	0	10.33	2.18	5	11.0	12								
	Visit 4 (Week 5, Day 29)	9	0	10.44	2.60	4	11.0	13	9	0	0.11	(-0.49, 0.71)	0.78	-1.0	0.0	1.0
	Visit 6 (Week 8, Day 53)	9	0	10.22	2.49	4	11.0	12	9	0	-0.11	(-0.71, 0.49)	0.78	-1.0	0.0	1.0
	Visit 8 (Week 12, Day 81)	9	0	10.56	2.60	4	11.0	13	9	0	0.22	(-0.52, 0.97)	0.97	-1.0	0.0	2.0
	Visit 14 (Week 24, Day 165)	9	0	10.44	2.13	5	11.0	12	9	0	0.11	(-0.35, 0.57)	0.60	-1.0	0.0	1.0

Miss. = Missing (subject attends a visit but measure not recorded)

Table 7.1 Summary of PUL2.0 Total Score Entry Score 0 to 6

Antisense Therapeutics Limited
Protocol: 1102-DMD-CT02
Population: All Enrolled Subjects (excludes Screen Failures) (N=9)

		Actual Values							Change From Baseline (Week 1, Day 1)							
Measure	Visit	n	Miss.	Mean	Std.	Min.	Median	Max.	n	Miss.	Mean	95% CI (for Mean)	Std.	Min.	Median	Max.
Entry Item A	Visit 2 (Week 1, Day 1)	9	0	3.56	1.81	1	3.0	6								
	Visit 4 (Week 5, Day 29)	9	0	3.78	1.92	1	4.0	6	9	0	0.22	(-0.29, 0.73)	0.67	-1.0	0.0	1.0
	Visit 6 (Week 8, Day 53)	9	0	3.89	1.76	1	4.0	6	9	0	0.33	(-0.21, 0.88)	0.71	-1.0	0.0	1.0
	Visit 8 (Week 12, Day 81)	9	0	3.78	1.79	1	3.0	6	9	0	0.22	(-0.29, 0.73)	0.67	-1.0	0.0	1.0
	Visit 14 (Week 24, Day 165)	9	0	3.67	1.66	1	3.0	6	9	0	0.11	(-0.35, 0.57)	0.60	-1.0	0.0	1.0

Miss. = Missing (subject attends a visit but measure not recorded)

Table 8 Summary of Egen Klassification Scale Version 2 (EK2)

Antisense Therapeutics Limited
Protocol: 1102-DMD-CT02
Population: All Enrolled Subjects (excludes Screen Failures) (N=9)

	Actual Values							Change From Baseline (Week 1, Day 1)								
Visit	n	Miss.	Mean	Std.	Min.	Median	Max.	n	Miss.	Mean	95% CI (for Mean)	Std.	Min.	Median	Max.
Visit 2 (Week 1, Day 1)	9	0	11.4	8.6	1	10.0	31								
Visit 4 (Week 5, Day 29)	9	0	11.3	8.7	1	11.0	32	9	0	-0.1	(-2.09, 1.87)	2.6	-6.0	0.0	2.0
Visit 6 (Week 8, Day 53)	9	0	11.7	11.0	0	8.0	39	9	0	0.2	(-3.03, 3.48)	4.2	-7.0	1.0	8.0
Visit 8 (Week 12, Day 81)	9	0	12.4	9.8	0	12.0	36	9	0	1.0	(-1.14, 3.14)	2.8	-3.0	2.0	5.0
Visit 14 (Week 24, Day 165)	9	0	11.7	7.5	3	11.0	30	9	0	0.2	(-1.80, 2.25)	2.6	-6.0	1.0	2.0

Miss. = Missing (subject attends a visit but measure not recorded)

Table 9 Summary of Respiratory Measures

Antisense Therapeutics Limited
Protocol: 1102-DMD-CT02
Population: All Enrolled Subjects (excludes Screen Failures) (N=9)

		Actual Values							Change From Baseline (Week 1, Day 1)							
Measure	Visit	n	Miss.	Mean	Std.	Min.	Median	Max.	n	Miss.	Mean	95% CI (for Mean)	Std.	Min.	Median	Max.
FEV1(L)	Visit 2 (Week 1, Day 1)	9	0	1.72	0.40	0.9	1.66	2.2								
	Visit 4 (Week 5, Day 29)	9	0	1.67	0.40	1.1	1.56	2.4	9	0	-0.05	(-0.19, 0.09)	0.18	-0.4	-0.02	0.2
	Visit 6 (Week 8, Day 53)	9	0	1.71	0.36	1.0	1.68	2.2	9	0	-0.01	(-0.09, 0.08)	0.11	-0.2	-0.02	0.1
	Visit 8 (Week 12, Day 81)	8	1	1.68	0.37	1.0	1.61	2.1	8	1	-0.06	(-0.18, 0.07)	0.15	-0.3	-0.04	0.1
	Visit 14 (Week 24, Day 165)	9	0	1.64	0.37	1.1	1.67	2.3	9	0	-0.07	(-0.20, 0.06)	0.17	-0.3	-0.12	0.2
	Visit 15 (Week 28, Day 190)	8	1	1.73	0.28	1.3	1.73	2.1	8	1	-0.09	(-0.22, 0.04)	0.15	-0.3	-0.06	0.2
FEV1 % Predicted	Visit 2 (Week 1, Day 1)	9	0	69.84	22.99	21.2	72.80	95.4								
	Visit 4 (Week 5, Day 29)	9	0	66.70	20.52	25.7	71.40	91.3	9	0	-3.14	(-9.17, 2.88)	7.84	-16.6	-0.90	5.5
	Visit 6 (Week 8, Day 53)	9	0	68.92	21.78	24.1	76.30	96.2	9	0	-0.92	(-4.69, 2.84)	4.90	-10.7	-0.90	6.7
	Visit 8 (Week 12, Day 81)	8	1	64.05	20.72	23.4	65.70	90.5	8	1	-2.93	(-7.62, 1.77)	5.61	-14.5	-2.00	2.2
	Visit 14 (Week 24, Day 165)	9	0	65.78	23.17	25.1	68.40	103.0	9	0	-4.07	(-9.46, 1.33)	7.02	-14.7	-4.60	7.6
	Visit 15 (Week 28, Day 190)	8	1	69.53	12.47	50.9	72.20	86.6	8	1	-6.40	(-12.3,-0.49)	7.07	-16.4	-8.25	4.7

Miss. = Missing (subject attends a visit but measure not recorded)

Table 9 Summary of Respiratory Measures

Antisense Therapeutics Limited
Protocol: 1102-DMD-CT02
Population: All Enrolled Subjects (excludes Screen Failures) (N=9)

		Actual Values							Change From Baseline (Week 1, Day 1)							
Measure	Visit	n	Miss.	Mean	Std.	Min.	Median	Max.	n	Miss.	Mean	95% CI (for Mean)	Std.	Min.	Median	Max.
FVC(L)	Visit 2 (Week 1, Day 1)	9	0	1.96	0.45	1.1	1.85	2.5								
	Visit 4 (Week 5, Day 29)	9	0	1.91	0.43	1.2	1.80	2.5	9	0	-0.05	(-0.17, 0.08)	0.16	-0.4	-0.05	0.2
	Visit 6 (Week 8, Day 53)	9	0	1.89	0.41	1.2	1.86	2.4	9	0	-0.06	(-0.20, 0.07)	0.17	-0.4	-0.03	0.1
	Visit 8 (Week 12, Day 81)	8	1	1.91	0.41	1.1	2.01	2.3	8	1	-0.08	(-0.17, 0.02)	0.11	-0.3	-0.05	0.0
	Visit 14 (Week 24, Day 165)	9	0	1.82	0.41	1.1	1.81	2.4	9	0	-0.14	(-0.25,-0.04)	0.14	-0.3	-0.18	0.0
	Visit 15 (Week 28, Day 190)	8	1	1.96	0.34	1.4	1.99	2.4	8	1	-0.10	(-0.24, 0.04)	0.16	-0.3	-0.10	0.1
FVC % Predicted	Visit 2 (Week 1, Day 1)	9	0	69.77	22.37	22.6	74.00	95.3								
	Visit 4 (Week 5, Day 29)	9	0	67.81	21.02	23.4	71.40	92.5	9	0	-1.96	(-6.26, 2.35)	5.60	-12.1	-1.80	6.3
	Visit 6 (Week 8, Day 53)	9	0	67.40	21.55	23.5	73.40	98.9	9	0	-2.37	(-7.52, 2.79)	6.71	-13.5	-1.50	5.9
	Visit 8 (Week 12, Day 81)	8	1	64.34	20.30	22.1	71.75	87.5	8	1	-3.03	(-6.41, 0.36)	4.05	-9.0	-1.85	1.0
	Visit 14 (Week 24, Day 165)	9	0	64.09	22.26	22.2	72.90	96.1	9	0	-5.68	(-9.60,-1.76)	5.10	-14.8	-6.50	0.8
	Visit 15 (Week 28, Day 190)	8	1	69.56	12.49	50.5	71.15	84.5	8	1	-6.10	(-12.1,-0.06)	7.23	-16.3	-7.15	3.6

Miss. = Missing (subject attends a visit but measure not recorded)

Table 9 Summary of Respiratory Measures

Antisense Therapeutics Limited
Protocol: 1102-DMD-CT02
Population: All Enrolled Subjects (excludes Screen Failures) (N=9)

		Actual Values							Change From Baseline (Week 1, Day 1)							
Measure	Visit	n	Miss.	Mean	Std.	Min.	Median	Max.	n	Miss.	Mean	95% CI (for Mean)	Std.	Min.	Median	Max.
PEF(L/s)	Visit 2 (Week 1, Day 1)	9	0	3.82	0.91	2.2	3.98	4.9								
	Visit 4 (Week 5, Day 29)	9	0	3.64	0.77	2.3	3.95	4.4	9	0	-0.18	(-0.70, 0.34)	0.68	-1.0	-0.46	1.1
	Visit 6 (Week 8, Day 53)	9	0	3.87	0.72	2.9	4.11	4.7	9	0	0.05	(-0.57, 0.67)	0.81	-1.1	-0.10	1.1
	Visit 8 (Week 12, Day 81)	8	1	3.98	0.85	2.3	4.34	4.8	8	1	0.18	(-0.40, 0.75)	0.69	-0.6	0.09	1.4
	Visit 14 (Week 24, Day 165)	9	0	3.95	0.59	3.1	3.81	5.1	9	0	0.13	(-0.31, 0.58)	0.58	-0.8	0.29	0.9
	Visit 15 (Week 28, Day 190)	8	1	3.93	0.96	1.9	4.25	4.8	8	1	-0.09	(-0.85, 0.67)	0.91	-1.5	-0.14	1.4
PEF % Predicted	Visit 2 (Week 1, Day 1)	9	0	71.86	27.12	25.8	84.10	103.0								
	Visit 4 (Week 5, Day 29)	9	0	66.47	20.78	30.6	73.80	86.5	9	0	-5.39	(-14.1, 3.34)	11.35	-20.9	-8.80	13.7
	Visit 6 (Week 8, Day 53)	9	0	70.84	22.16	32.5	74.30	101.8	9	0	-1.01	(-12.7,10.71)	15.25	-28.7	-2.10	17.7
	Visit 8 (Week 12, Day 81)	8	1	68.64	23.93	34.6	71.00	93.9	8	1	0.68	(-8.27, 9.62)	10.70	-11.2	1.75	17.0
	Visit 14 (Week 24, Day 165)	9	0	71.91	24.18	35.0	71.40	98.7	9	0	0.06	(-8.33, 8.44)	10.91	-18.2	6.30	9.2
	Visit 15 (Week 28, Day 190)	8	1	72.78	22.47	35.1	77.80	100.0	8	1	-4.84	(-17.5, 7.84)	15.17	-29.8	-4.70	15.9

Miss. = Missing (subject attends a visit but measure not recorded)

Table 9 Summary of Respiratory Measures

Antisense Therapeutics Limited
Protocol: 1102-DMD-CT02
Population: All Enrolled Subjects (excludes Screen Failures) (N=9)

		Actual Values							Change From Baseline (Week 1, Day 1)							
Measure	Visit	n	Miss.	Mean	Std.	Min.	Median	Max.	n	Miss.	Mean	95% CI (for Mean)	Std.	Min.	Median	Max.
CPF(L/s)	Visit 2 (Week 1, Day 1)	9	0	4.64	0.71	3.6	4.31	5.7								
	Visit 4 (Week 5, Day 29)	9	0	4.48	0.77	3.0	4.54	5.5	9	0	-0.15	(-0.53, 0.22)	0.49	-1.1	0.02	0.3
	Visit 6 (Week 8, Day 53)	9	0	4.46	0.83	3.2	4.66	5.7	9	0	-0.18	(-0.50, 0.14)	0.42	-0.8	-0.15	0.5
	Visit 8 (Week 12, Day 81)	8	1	4.73	0.55	3.8	4.74	5.5	8	1	0.04	(-0.17, 0.24)	0.25	-0.4	0.09	0.3
	Visit 14 (Week 24, Day 165)	9	0	4.43	0.72	3.4	4.29	5.8	9	0	-0.20	(-0.72, 0.31)	0.67	-1.2	-0.02	0.7
	Visit 15 (Week 28, Day 190)	8	1	4.43	0.82	3.0	4.82	5.1	8	1	-0.25	(-0.75, 0.24)	0.59	-1.1	-0.43	0.6
MIP(cmH2O)	Visit 2 (Week 1, Day 1)	9	0	34.75	26.83	2.2	28.35	79.4								
	Visit 4 (Week 5, Day 29)	9	0	35.24	28.82	3.9	32.22	77.4	9	0	0.49	(-16.1,17.04)	21.53	-45.5	1.20	34.5
	Visit 6 (Week 8, Day 53)	9	0	30.54	29.49	1.3	12.75	74.2	9	0	-4.21	(-37.6,29.17)	43.42	-71.0	0.00	63.8
	Visit 8 (Week 12, Day 81)	8	1	30.87	34.33	1.2	11.17	82.8	8	1	-7.26	(-38.8,24.34)	37.79	-60.9	-4.03	42.7
	Visit 14 (Week 24, Day 165)	9	0	11.86	21.06	0.6	5.66	67.6	9	0	-22.9	(-49.3, 3.47)	34.29	-71.1	-19.5	39.3
	Visit 15 (Week 28, Day 190)	8	1	17.71	23.24	0.5	6.73	63.6	8	1	-21.1	(-50.3, 8.11)	34.94	-71.3	-16.8	38.5
MEP(cmH2O)	Visit 2 (Week 1, Day 1)	9	0	35.59	26.27	1.5	30.32	79.6								
	Visit 4 (Week 5, Day 29)	9	0	33.90	23.55	4.5	26.38	65.1	9	0	-1.69	(-14.4,11.02)	16.54	-39.1	3.71	17.6
	Visit 6 (Week 8, Day 53)	9	0	32.48	32.10	1.5	28.96	86.4	9	0	-3.10	(-32.5,26.29)	38.24	-57.7	-0.02	78.6
	Visit 8 (Week 12, Day 81)	8	1	25.77	28.59	2.2	11.86	76.8	8	1	-13.3	(-36.4, 9.84)	27.66	-58.4	-11.7	19.3
	Visit 14 (Week 24, Day 165)	9	0	12.78	24.33	1.4	4.88	77.4	9	0	-22.8	(-38.9,-6.76)	20.88	-58.2	-20.7	-0.1
	Visit 15 (Week 28, Day 190)	8	1	14.42	17.59	1.6	7.14	51.2	8	1	-25.4	(-53.2, 2.35)	33.23	-71.9	-23.8	32.3

Miss. = Missing (subject attends a visit but measure not recorded)

Table 10.1 Summary of MYOSET Measures

Antisense Therapeutics Limited
Protocol: 1102-DMD-CT02
Population: All Enrolled Subjects (excludes Screen Failures) (N=9)

		Actual Values							Change From Baseline (Week 1, Day 1)							
Measure	Visit	n	Miss.	Mean	Std.	Min.	Median	Max.	n	Miss.	Mean	95% CI (for Mean)	Std.	Min.	Median	Max.
Grip(kg) - Dominant	Visit 2 (Week 1, Day 1)	9	0	6.6	2.8	1	7.6	9								
	Visit 4 (Week 5, Day 29)	9	0	6.8	3.0	1	7.6	10	9	0	0.2	(-0.45, 0.82)	0.8	-0.8	0.1	1.7
	Visit 6 (Week 8, Day 53)	9	0	7.3	3.2	1	8.3	10	9	0	0.7	(0.07, 1.34)	0.8	-0.2	0.4	1.9
	Visit 8 (Week 12, Day 81)	9	0	6.6	2.8	0	7.3	10	9	0	0.0	(-0.50, 0.54)	0.7	-1.3	0.0	0.9
	Visit 14 (Week 24, Day 165)	9	0	6.8	2.9	1	7.6	10	9	0	0.2	(-0.25, 0.67)	0.6	-0.6	0.1	1.1
Grip(kg) - Non-Dominant	Visit 2 (Week 1, Day 1)	9	0	6.5	2.9	1	7.1	10								
	Visit 4 (Week 5, Day 29)	9	0	6.9	3.3	1	7.6	12	9	0	0.4	(-0.13, 0.97)	0.7	-0.2	0.1	1.7
	Visit 6 (Week 8, Day 53)	9	0	7.0	3.2	1	7.9	12	9	0	0.5	(-0.32, 1.33)	1.1	-1.5	0.7	1.9
	Visit 8 (Week 12, Day 81)	9	0	6.8	3.3	1	7.2	12	9	0	0.3	(-0.44, 1.09)	1.0	-1.4	0.1	2.1
	Visit 14 (Week 24, Day 165)	9	0	6.9	3.4	1	7.5	12	9	0	0.4	(-0.16, 0.93)	0.7	-0.4	0.4	1.9

Miss. = Missing (subject attends a visit but measure not recorded)
Average* = Average of Left and Right Hands

Table 10.1 Summary of MYOSET Measures

Antisense Therapeutics Limited
Protocol: 1102-DMD-CT02
Population: All Enrolled Subjects (excludes Screen Failures) (N=9)

		Actual Values							Change From Baseline (Week 1, Day 1)							
Measure	Visit	n	Miss.	Mean	Std.	Min.	Median	Max.	n	Miss.	Mean	95% CI (for Mean)	Std.	Min.	Median	Max.
Pinch(kg) - Dominant	Visit 2 (Week 1, Day 1)	9	0	1.8	0.8	0	2.0	3								
	Visit 4 (Week 5, Day 29)	9	0	1.9	0.9	0	2.0	3	9	0	0.2	(0.02, 0.30)	0.2	-0.0	0.1	0.5
	Visit 6 (Week 8, Day 53)	9	0	2.0	0.9	0	2.1	3	9	0	0.2	(0.03, 0.37)	0.2	-0.0	0.1	0.7
	Visit 8 (Week 12, Day 81)	9	0	1.9	0.9	0	2.0	3	9	0	0.1	(-0.04, 0.29)	0.2	-0.0	0.0	0.7
	Visit 14 (Week 24, Day 165)	9	0	1.8	0.9	0	1.9	3	9	0	0.0	(-0.18, 0.19)	0.2	-0.4	0.0	0.4
Pinch(kg) - Non-Dominant	Visit 2 (Week 1, Day 1)	9	0	1.8	0.9	0	1.8	3								
	Visit 4 (Week 5, Day 29)	9	0	1.9	0.9	0	2.0	3	9	0	0.1	(-0.19, 0.44)	0.4	-0.6	0.1	0.7
	Visit 6 (Week 8, Day 53)	9	0	2.0	0.9	0	2.1	3	9	0	0.2	(0.02, 0.37)	0.2	-0.1	0.3	0.6
	Visit 8 (Week 12, Day 81)	9	0	2.0	1.0	0	2.0	3	9	0	0.2	(-0.14, 0.49)	0.4	-0.3	0.0	1.0
	Visit 14 (Week 24, Day 165)	9	0	1.8	0.9	0	1.9	3	9	0	0.0	(-0.21, 0.28)	0.3	-0.4	0.0	0.6

Miss. = Missing (subject attends a visit but measure not recorded)
Average* = Average of Left and Right Hands

Table 10.1 Summary of MYOSET Measures

Antisense Therapeutics Limited
Protocol: 1102-DMD-CT02
Population: All Enrolled Subjects (excludes Screen Failures) (N=9)

		Actual Values							Change From Baseline (Week 1, Day 1)							
Measure	Visit	n	Miss.	Mean	Std.	Min.	Median	Max.	n	Miss.	Mean	95% CI (for Mean)	Std.	Min.	Median	Max.
Moviplate(Taps in 30 secs) - Dominant	Visit 2 (Week 1, Day 1)	9	0	60.1	11.1	35	63.0	74								
	Visit 4 (Week 5, Day 29)	9	0	60.6	15.0	25	66.0	78	9	0	0.4	(-4.02, 4.91)	5.8	-10.0	2.0	8.0
	Visit 6 (Week 8, Day 53)	9	0	64.6	13.1	33	66.0	78	9	0	4.4	(0.16, 8.73)	5.6	-2.0	2.0	14.0
	Visit 8 (Week 12, Day 81)	9	0	61.3	17.1	18	67.0	75	9	0	1.2	(-4.79, 7.24)	7.8	-17.0	1.0	10.0
	Visit 14 (Week 24, Day 165)	9	0	62.0	17.9	20	64.0	82	9	0	1.9	(-6.08, 9.85)	10.4	-15.0	7.0	13.0
Moviplate(Taps in 30 secs) - Non-Dominant	Visit 2 (Week 1, Day 1)	9	0	56.0	10.4	30	59.0	63								
	Visit 4 (Week 5, Day 29)	9	0	57.8	12.6	32	62.0	73	9	0	1.8	(-2.46, 6.02)	5.5	-9.0	2.0	11.0
	Visit 6 (Week 8, Day 53)	9	0	58.7	16.5	16	63.0	70	9	0	2.7	(-2.45, 7.78)	6.7	-14.0	4.0	8.0
	Visit 8 (Week 12, Day 81)	9	0	57.2	18.9	15	65.0	70	9	0	1.2	(-6.45, 8.90)	10.0	-17.0	5.0	9.0
	Visit 14 (Week 24, Day 165)	9	0	57.8	16.3	16	63.0	68	9	0	1.8	(-4.32, 7.88)	7.9	-14.0	5.0	10.0

Miss. = Missing (subject attends a visit but measure not recorded)
Average* = Average of Left and Right Hands

Table 10.1 Summary of MYOSET Measures

Antisense Therapeutics Limited
Protocol: 1102-DMD-CT02
Population: All Enrolled Subjects (excludes Screen Failures) (N=9)

		Actual Values							Change From Baseline (Week 1, Day 1)							
Measure	Visit	n	Miss.	Mean	Std.	Min.	Median	Max.	n	Miss.	Mean	95% CI (for Mean)	Std.	Min.	Median	Max.
Grip(kg) - Average*	Visit 2 (Week 1, Day 1)	9	0	6.5	2.9	1	7.3	10								
	Visit 4 (Week 5, Day 29)	9	0	6.8	3.1	1	7.6	11	9	0	0.3	(-0.15, 0.76)	0.6	-0.4	0.2	1.2
	Visit 6 (Week 8, Day 53)	9	0	7.1	3.1	1	8.1	10	9	0	0.6	(0.10, 1.10)	0.7	-0.2	0.5	1.9
	Visit 8 (Week 12, Day 81)	9	0	6.7	3.0	1	7.3	11	9	0	0.2	(-0.31, 0.65)	0.6	-0.7	0.2	1.2
	Visit 14 (Week 24, Day 165)	9	0	6.8	3.1	1	7.6	11	9	0	0.3	(-0.17, 0.77)	0.6	-0.5	0.1	1.5
Pinch(kg) - Average*	Visit 2 (Week 1, Day 1)	9	0	1.8	0.8	0	1.9	3								
	Visit 4 (Week 5, Day 29)	9	0	1.9	0.9	0	2.0	3	9	0	0.1	(-0.05, 0.33)	0.2	-0.3	0.1	0.5
	Visit 6 (Week 8, Day 53)	9	0	2.0	0.9	0	2.1	3	9	0	0.2	(0.06, 0.33)	0.2	0.0	0.2	0.5
	Visit 8 (Week 12, Day 81)	9	0	1.9	0.9	0	2.0	3	9	0	0.1	(-0.07, 0.36)	0.3	-0.0	0.0	0.8
	Visit 14 (Week 24, Day 165)	9	0	1.8	0.9	0	2.0	3	9	0	0.0	(-0.17, 0.21)	0.2	-0.4	0.1	0.4

Miss. = Missing (subject attends a visit but measure not recorded)
Average* = Average of Left and Right Hands

Table 10.2 Summary of MYOSET Measures - Percent Predicted

Antisense Therapeutics Limited
Protocol: 1102-DMD-CT02
Population: All Enrolled Subjects (excludes Screen Failures) (N=9)

			|--- Actual Percent Predicted Values-------|						|-------Change From Baseline (Week 1, Day 1)--------|						
Side	Measure	Visit	n	Mean	Std.	Min.	Median	Max.	n	Mean	95% CI (for Mean)	Std.	Min.	Median	Max.
AVERAGE	PINCH	Visit 2 (Week 1, Day 1)	9	25.2	12.0	4.4	25.1	40.2							
		Visit 4 (Week 5, Day 29)	9	27.0	12.9	4.8	28.1	43.3	9	1.8	(-0.75, 4.43)	3.4	-4.2	1.5	6.6
		Visit 6 (Week 8, Day 53)	9	27.7	13.2	5.1	29.2	45.9	9	2.5	(0.71, 4.31)	2.3	-0.1	2.1	6.0
		Visit 8 (Week 12, Day 81)	9	26.7	12.8	4.4	30.4	40.2	9	1.5	(-1.14, 4.16)	3.4	-1.1	-0.0	9.1
		Visit 14 (Week 24, Day 165)	9	24.4	11.5	5.1	28.6	36.2	9	-0.7	(-3.56, 2.09)	3.7	-6.8	-0.1	3.5
AVERAGE	GRIP	Visit 2 (Week 1, Day 1)	9	20.5	10.8	1.8	23.0	34.7							
		Visit 4 (Week 5, Day 29)	9	21.0	10.6	1.6	24.4	32.7	9	0.5	(-0.80, 1.87)	1.7	-2.0	0.4	2.5
		Visit 6 (Week 8, Day 53)	9	22.1	12.0	1.3	24.5	42.0	9	1.6	(-0.32, 3.55)	2.5	-0.8	1.1	7.4
		Visit 8 (Week 12, Day 81)	9	20.2	9.8	1.5	23.4	30.5	9	-0.3	(-1.97, 1.39)	2.2	-4.1	0.0	2.6
		Visit 14 (Week 24, Day 165)	9	20.0	9.9	1.8	23.8	31.5	9	-0.5	(-1.91, 1.01)	1.9	-4.0	-0.0	1.8

Side* : Average is the average of the Left and Right hand Pinch and Grip results as input to the Percent Predicted calculation (which uses the Subjects's AGE)

Table 10.2 Summary of MYOSET Measures - Percent Predicted

Antisense Therapeutics Limited
Protocol: 1102-DMD-CT02
Population: All Enrolled Subjects (excludes Screen Failures) (N=9)

			|--- Actual Percent Predicted Values-------|						|-------Change From Baseline (Week 1, Day 1)--------|						
Side	Measure	Visit	n	Mean	Std.	Min.	Median	Max.	n	Mean	95% CI (for Mean)	Std.	Min.	Median	Max.
DOMINANT	PINCH	Visit 2 (Week 1, Day 1)	9	24.9	11.0	5.1	28.9	37.2							
		Visit 4 (Week 5, Day 29)	9	27.0	12.6	5.8	28.8	41.5	9	2.1	(0.30, 3.86)	2.3	-0.1	0.7	5.6
		Visit 6 (Week 8, Day 53)	9	27.4	12.6	6.1	28.8	43.2	9	2.4	(0.33, 4.56)	2.8	-0.5	1.3	7.9
		Visit 8 (Week 12, Day 81)	9	26.1	12.2	5.3	29.3	39.4	9	1.2	(-0.67, 3.08)	2.4	-0.6	0.2	7.3
		Visit 14 (Week 24, Day 165)	9	24.0	11.1	5.8	26.2	36.3	9	-1.0	(-3.56, 1.63)	3.4	-6.6	-0.3	3.0
DOMINANT	GRIP	Visit 2 (Week 1, Day 1)	9	20.8	11.2	1.6	21.5	36.4							
		Visit 4 (Week 5, Day 29)	9	20.9	10.7	1.4	22.8	32.6	9	0.2	(-1.97, 2.35)	2.8	-3.7	-0.1	4.8
		Visit 6 (Week 8, Day 53)	9	22.8	12.8	1.2	22.2	43.7	9	2.0	(-0.24, 4.26)	2.9	-1.3	0.7	7.4
		Visit 8 (Week 12, Day 81)	9	20.2	10.4	1.0	21.4	35.3	9	-0.6	(-2.41, 1.24)	2.4	-6.0	-0.5	2.3
		Visit 14 (Week 24, Day 165)	9	20.0	9.9	1.7	22.5	32.6	9	-0.7	(-2.33, 0.90)	2.1	-4.5	0.1	1.1

Side* : Average is the average of the Left and Right hand Pinch and Grip results as input to the Percent Predicted calculation (which uses the Subjects's AGE)

Table 10.2 Summary of MYOSET Measures - Percent Predicted

Antisense Therapeutics Limited
Protocol: 1102-DMD-CT02
Population: All Enrolled Subjects (excludes Screen Failures) (N=9)

			|--- Actual Percent Predicted Values-------|						|-------Change From Baseline (Week 1, Day 1)--------|						
Side	Measure	Visit	n	Mean	Std.	Min.	Median	Max.	n	Mean	95% CI (for Mean)	Std.	Min.	Median	Max.
NON-DOMINANT	PINCH	Visit 2 (Week 1, Day 1)	9	25.4	13.5	3.7	25.6	43.2							
		Visit 4 (Week 5, Day 29)	9	27.0	13.3	3.7	31.1	45.0	9	1.6	(-2.85, 6.06)	5.8	-8.4	1.8	10.3
		Visit 6 (Week 8, Day 53)	9	28.0	14.0	4.0	29.6	48.6	9	2.6	(-0.09, 5.24)	3.5	-2.5	3.4	8.8
		Visit 8 (Week 12, Day 81)	9	27.2	13.4	3.4	31.6	41.1	9	1.8	(-2.41, 6.04)	5.5	-4.4	-0.3	10.8
		Visit 14 (Week 24, Day 165)	9	24.9	12.1	4.4	28.4	36.1	9	-0.5	(-4.23, 3.20)	4.8	-8.6	-0.7	7.6
NON-DOMINANT	GRIP	Visit 2 (Week 1, Day 1)	9	20.2	10.5	2.0	23.2	33.0							
		Visit 4 (Week 5, Day 29)	9	21.1	10.7	1.8	24.0	32.7	9	0.9	(-0.39, 2.14)	1.6	-0.5	0.1	3.7
		Visit 6 (Week 8, Day 53)	9	21.4	11.4	1.4	23.7	40.4	9	1.2	(-1.56, 3.99)	3.6	-5.3	2.1	7.4
		Visit 8 (Week 12, Day 81)	9	20.2	9.6	1.9	23.1	29.5	9	0.0	(-2.51, 2.52)	3.3	-7.2	-0.0	4.0
		Visit 14 (Week 24, Day 165)	9	20.0	10.0	1.8	24.0	30.4	9	-0.2	(-1.67, 1.29)	1.9	-3.5	-0.2	2.6

Side* : Average is the average of the Left and Right hand Pinch and Grip results as input to the Percent Predicted calculation (which uses the Subjects's AGE)

Baseline*=Visit 2 (Week 1, Day 1); Change from Baseline*=(Visit Value-Baseline Value); Percent Change*:((Visit Value-Baseline Value)/(Baseline Value)) x 100
Percent Change only calculated if the Baseline Value was not missing and not zero

Table 5.3.5.1 Summary of Haematology - Lymphocytes (absolute)

Antisense Therapeutics Limited
Protocol: 1102-DMD-CT02
Population: All Enrolled Subjects (excludes Screen Failures)(N=9)

	Haematology Parameter : Lymphocytes (absolute) (x 10^9/L)													
	Actual Values							Change From Baseline (Week 1, Day 1)						
Visit	n	Miss.	Mean	Std.	Min.	Median	Max.	n	Miss.	Mean	Std.	Min.	Median	Max.
Week 1, (Day 1)	9	0	3.68	1.47	1.03	4.00	6.16							
Week 5, (Day 29)	9	0	3.88	1.38	1.54	4.01	6.73	9	0	0.19	0.78	-1.18	0.51	1.00
Week 8, (Day 53)	9	0	3.12	1.38	1.12	3.33	5.53	9	0	-0.56	1.25	-2.88	-0.20	0.83
Week 12, (Day 81)	9	0	3.15	1.44	0.96	3.25	5.72	9	0	-0.53	1.45	-3.04	-0.44	1.23
Week 24, (Day 165)	9	0	3.41	1.29	1.63	3.52	5.90	9	0	-0.28	1.07	-2.60	-0.23	1.18
Week 28, (Day 190)	9	0	3.87	1.66	1.51	4.00	7.20	9	0	0.19	0.81	-1.55	0.48	1.04

Baseline*=Visit 2 (Week 1, Day 1); Change from Baseline*=(Visit Value-Baseline Value); Percent Change*:((Visit Value-Baseline Value)/(Baseline Value)) x 100
Percent Change only calculated if the Baseline Value was not missing and not zero

Table 5.3.5.2 Summary of Haematology - Lymphocytes (absolute)

Antisense Therapeutics Limited
Protocol: 1102-DMD-CT02
Population: All Enrolled Subjects (excludes Screen Failures)(N=9)

	Haematology Parameter : Lymphocytes (absolute) (x 10^9/L)													
	Actual Values							Pct Chg From Baseline (Week 1, Day 1)						
Visit	n	Miss.	Mean	Std.	Min.	Median	Max.	n	Miss.	Mean	Std.	Min.	Median	Max.
Week 1, (Day 1)	9	0	3.68	1.47	1.03	4.00	6.16							
Week 5, (Day 29)	9	0	3.88	1.38	1.54	4.01	6.73	9	0	11.81	25.58	-24.9	9.25	49.51
Week 8, (Day 53)	9	0	3.12	1.38	1.12	3.33	5.53	9	0	-7.62	36.29	-72.0	-4.63	36.73
Week 12, (Day 81)	9	0	3.15	1.44	0.96	3.25	5.72	9	0	-3.45	46.67	-76.0	-7.14	66.02
Week 24, (Day 165)	9	0	3.41	1.29	1.63	3.52	5.90	9	0	0.70	32.94	-55.0	-4.22	58.25
Week 28, (Day 190)	9	0	3.87	1.66	1.51	4.00	7.20	9	0	8.44	24.09	-32.8	11.81	46.60

Table 11.1.1.1 Summary of FACS Tube 1

Antisense Therapeutics Limited
Protocol: 1102-DMD-CT02
Population: All Enrolled Subjects (excludes Screen Failures) (N=9)

		Actual Values							Change From Baseline (Week 1, Day 1)							
Measure	Visit	n	Miss.	Mean	Std.	Min.	Median	Max.	n	Miss.	Mean	95% CI (for Mean)	Std.	Min.	Median	Max.
CD3	Visit 2 (Week 1, Day 1)	9	0	2.93	1.20	0.76	3.21	5.11								
	Visit 4 (Week 5, Day 29)	9	0	3.15	1.11	1.10	3.12	5.39	9	0	0.22	(-0.17, 0.61)	0.51	-0.59	0.28	0.90
	Visit 6 (Week 8, Day 53)	9	0	2.40	1.23	0.63	2.64	4.56	9	0	-0.53	(-1.34, 0.27)	1.05	-2.58	-0.56	0.63
	Visit 8 (Week 12, Day 81)	9	0	2.60	1.22	0.62	2.89	4.70	9	0	-0.33	(-1.24, 0.58)	1.19	-2.59	-0.18	0.95
	Visit 14 (Week 24, Day 165)	9	0	2.75	1.08	1.13	2.86	4.78	9	0	-0.18	(-0.79, 0.43)	0.80	-1.90	0.03	0.91
	Visit 15 (Week 28, Day 190)	9	0	3.18	1.40	1.08	3.27	5.99	9	0	0.25	(-0.17, 0.66)	0.54	-0.69	0.33	0.87
CD3 CD49d+	Visit 2 (Week 1, Day 1)	9	0	2.44	1.02	0.69	2.63	4.34								
	Visit 4 (Week 5, Day 29)	9	0	2.60	0.97	0.97	2.39	4.71	9	0	0.16	(-0.17, 0.48)	0.42	-0.56	0.29	0.67
	Visit 6 (Week 8, Day 53)	9	0	1.94	1.06	0.50	2.10	4.01	9	0	-0.50	(-1.17, 0.17)	0.87	-2.13	-0.33	0.55
	Visit 8 (Week 12, Day 81)	9	0	2.05	0.99	0.53	2.22	3.97	9	0	-0.39	(-1.12, 0.33)	0.94	-2.11	-0.27	0.69
	Visit 14 (Week 24, Day 165)	9	0	2.16	0.76	1.00	2.28	3.66	9	0	-0.28	(-0.78, 0.21)	0.64	-1.58	-0.21	0.61
	Visit 15 (Week 28, Day 190)	9	0	2.55	1.07	0.93	2.57	4.77	9	0	0.11	(-0.26, 0.48)	0.48	-0.74	0.24	0.82

Miss. = Missing (subject attends a visit but measure not recorded)
All units = x10^9/L

Table 11.1.1.1 Summary of FACS Tube 1

Antisense Therapeutics Limited
Protocol: 1102-DMD-CT02
Population: All Enrolled Subjects (excludes Screen Failures) (N=9)

		Actual Values							Change From Baseline (Week 1, Day 1)							
Measure	Visit	n	Miss.	Mean	Std.	Min.	Median	Max.	n	Miss.	Mean	95% CI (for Mean)	Std.	Min.	Median	Max.
CD3 CD49d-	Visit 2 (Week 1, Day 1)	9	0	0.48	0.21	0.08	0.53	0.77								
	Visit 4 (Week 5, Day 29)	9	0	0.55	0.28	0.13	0.62	0.88	9	0	0.07	(-0.08, 0.22)	0.19	-0.21	0.05	0.35
	Visit 6 (Week 8, Day 53)	9	0	0.46	0.32	0.10	0.40	1.14	9	0	-0.02	(-0.22, 0.18)	0.26	-0.45	0.02	0.47
	Visit 8 (Week 12, Day 81)	9	0	0.54	0.35	0.09	0.46	1.04	9	0	0.05	(-0.19, 0.30)	0.32	-0.49	0.01	0.53
	Visit 14 (Week 24, Day 165)	9	0	0.57	0.37	0.12	0.56	1.06	9	0	0.09	(-0.09, 0.27)	0.24	-0.33	0.04	0.36
	Visit 15 (Week 28, Day 190)	9	0	0.62	0.35	0.12	0.67	1.14	9	0	0.13	(-0.03, 0.30)	0.21	-0.21	0.14	0.49
CD3-	Visit 2 (Week 1, Day 1)	9	0	0.74	0.32	0.24	0.77	1.21								
	Visit 4 (Week 5, Day 29)	9	0	0.71	0.33	0.34	0.78	1.32	9	0	-0.02	(-0.28, 0.23)	0.33	-0.79	0.07	0.29
	Visit 6 (Week 8, Day 53)	9	0	0.63	0.35	0.22	0.49	1.35	9	0	-0.11	(-0.41, 0.19)	0.39	-0.76	-0.08	0.57
	Visit 8 (Week 12, Day 81)	9	0	0.54	0.29	0.27	0.37	1.02	9	0	-0.20	(-0.49, 0.09)	0.38	-0.91	-0.28	0.28
	Visit 14 (Week 24, Day 165)	9	0	0.65	0.26	0.34	0.62	1.10	9	0	-0.09	(-0.32, 0.14)	0.30	-0.68	-0.11	0.28
	Visit 15 (Week 28, Day 190)	9	0	0.68	0.32	0.28	0.76	1.19	9	0	-0.06	(-0.31, 0.19)	0.33	-0.84	0.04	0.28

Miss. = Missing (subject attends a visit but measure not recorded)
All units = x10^9/L

Table 11.1.1.1 Summary of FACS Tube 1

Antisense Therapeutics Limited
Protocol: 1102-DMD-CT02
Population: All Enrolled Subjects (excludes Screen Failures) (N=9)

		|------------------- Actual Values-----------------------|							|---------- Change From Baseline (Week 1, Day 1)-----------|							
Measure	Visit	n	Miss.	Mean	Std.	Min.	Median	Max.	n	Miss.	Mean	95% CI (for Mean)	Std.	Min.	Median	Max.
CD4	Visit 2 (Week 1, Day 1)	9	0	1.57	0.67	0.34	1.53	2.48								
	Visit 4 (Week 5, Day 29)	9	0	1.72	0.62	0.42	1.87	2.53	9	0	0.15	(-0.05, 0.35)	0.26	-0.18	0.08	0.54
	Visit 6 (Week 8, Day 53)	9	0	1.27	0.63	0.31	1.37	2.17	9	0	-0.30	(-0.76, 0.15)	0.59	-1.57	-0.30	0.30
	Visit 8 (Week 12, Day 81)	9	0	1.36	0.66	0.32	1.39	2.22	9	0	-0.20	(-0.72, 0.32)	0.68	-1.56	-0.06	0.57
	Visit 14 (Week 24, Day 165)	9	0	1.42	0.58	0.46	1.59	2.25	9	0	-0.15	(-0.52, 0.23)	0.48	-1.23	-0.02	0.52
	Visit 15 (Week 28, Day 190)	9	0	1.68	0.75	0.47	1.88	2.89	9	0	0.11	(-0.12, 0.34)	0.30	-0.37	0.16	0.41
CD4 CD49d High	Visit 2 (Week 1, Day 1)	9	0	0.24	0.10	0.08	0.24	0.39								
	Visit 4 (Week 5, Day 29)	9	0	0.28	0.13	0.07	0.30	0.48	9	0	0.04	(-0.01, 0.09)	0.06	-0.02	0.05	0.16
	Visit 6 (Week 8, Day 53)	9	0	0.19	0.12	0.06	0.19	0.41	9	0	-0.05	(-0.12, 0.03)	0.10	-0.18	-0.03	0.09
	Visit 8 (Week 12, Day 81)	9	0	0.22	0.12	0.06	0.21	0.42	9	0	-0.02	(-0.11, 0.07)	0.12	-0.26	0.03	0.10
	Visit 14 (Week 24, Day 165)	9	0	0.23	0.10	0.09	0.27	0.34	9	0	-0.01	(-0.06, 0.04)	0.06	-0.09	-0.02	0.08
	Visit 15 (Week 28, Day 190)	9	0	0.25	0.10	0.09	0.27	0.37	9	0	0.01	(-0.02, 0.05)	0.04	-0.04	0.01	0.08

Miss. = Missing (subject attends a visit but measure not recorded)
All units = x10^9/L

Table 11.1.1.1 Summary of FACS Tube 1

Antisense Therapeutics Limited
Protocol: 1102-DMD-CT02
Population: All Enrolled Subjects (excludes Screen Failures) (N=9)

		Actual Values							Change From Baseline (Week 1, Day 1)							
Measure	Visit	n	Miss.	Mean	Std.	Min.	Median	Max.	n	Miss.	Mean	95% CI (for Mean)	Std.	Min.	Median	Max.
CD4 CD49d+	Visit 2 (Week 1, Day 1)	9	0	1.20	0.55	0.29	1.13	1.88								
	Visit 4 (Week 5, Day 29)	9	0	1.28	0.53	0.33	1.41	1.92	9	0	0.08	(-0.06, 0.22)	0.18	-0.19	0.09	0.35
	Visit 6 (Week 8, Day 53)	9	0	0.92	0.52	0.22	1.03	1.70	9	0	-0.28	(-0.64, 0.08)	0.47	-1.28	-0.12	0.18
	Visit 8 (Week 12, Day 81)	9	0	0.97	0.49	0.26	1.03	1.67	9	0	-0.22	(-0.63, 0.18)	0.53	-1.25	-0.13	0.32
	Visit 14 (Week 24, Day 165)	9	0	1.01	0.40	0.37	1.12	1.47	9	0	-0.19	(-0.48, 0.10)	0.38	-1.06	-0.15	0.26
	Visit 15 (Week 28, Day 190)	9	0	1.21	0.53	0.37	1.39	1.96	9	0	0.01	(-0.20, 0.21)	0.27	-0.36	0.03	0.53
CD4 CD49d Negative	Visit 2 (Week 1, Day 1)	9	0	0.36	0.19	0.05	0.36	0.68								
	Visit 4 (Week 5, Day 29)	9	0	0.43	0.24	0.09	0.42	0.78	9	0	0.07	(-0.03, 0.17)	0.13	-0.08	0.04	0.29
	Visit 6 (Week 8, Day 53)	9	0	0.34	0.24	0.07	0.30	0.76	9	0	-0.02	(-0.14, 0.11)	0.16	-0.27	-0.02	0.28
	Visit 8 (Week 12, Day 81)	9	0	0.38	0.27	0.06	0.35	0.89	9	0	0.02	(-0.13, 0.17)	0.19	-0.30	0.02	0.39
	Visit 14 (Week 24, Day 165)	9	0	0.40	0.29	0.09	0.37	0.81	9	0	0.04	(-0.06, 0.15)	0.14	-0.16	0.01	0.26
	Visit 15 (Week 28, Day 190)	9	0	0.46	0.29	0.06	0.46	0.89	9	0	0.10	(-0.04, 0.24)	0.18	-0.21	0.11	0.45

Miss. = Missing (subject attends a visit but measure not recorded)
All units = x10^9/L

Table 11.1.1.1 Summary of FACS Tube 1

Antisense Therapeutics Limited
Protocol: 1102-DMD-CT02
Population: All Enrolled Subjects (excludes Screen Failures) (N=9)

		Actual Values							Change From Baseline (Week 1, Day 1)							
Measure	Visit	n	Miss.	Mean	Std.	Min.	Median	Max.	n	Miss.	Mean	95% CI (for Mean)	Std.	Min.	Median	Max.
CD4 CD49d Low	Visit 2 (Week 1, Day 1)	9	0	0.96	0.50	0.21	0.94	1.69								
	Visit 4 (Week 5, Day 29)	9	0	1.01	0.48	0.26	1.15	1.69	9	0	0.05	(-0.05, 0.14)	0.12	-0.14	0.05	0.21
	Visit 6 (Week 8, Day 53)	9	0	0.73	0.47	0.17	0.62	1.47	9	0	-0.23	(-0.53, 0.07)	0.39	-1.12	-0.09	0.13
	Visit 8 (Week 12, Day 81)	9	0	0.76	0.42	0.20	0.71	1.40	9	0	-0.20	(-0.53, 0.13)	0.43	-1.09	-0.11	0.21
	Visit 14 (Week 24, Day 165)	9	0	0.79	0.34	0.27	0.77	1.21	9	0	-0.17	(-0.43, 0.08)	0.33	-0.95	-0.12	0.18
	Visit 15 (Week 28, Day 190)	9	0	0.96	0.46	0.28	1.08	1.61	9	0	-0.01	(-0.20, 0.19)	0.25	-0.38	-0.05	0.49
CD4 HLA-DR Positive	Visit 2 (Week 1, Day 1)	9	0	0.02	0.01	0.01	0.02	0.03								
	Visit 4 (Week 5, Day 29)	9	0	0.02	0.01	0.01	0.02	0.03	9	0	0.00	(-0.00, 0.01)	0.01	-0.01	0.00	0.01
	Visit 6 (Week 8, Day 53)	9	0	0.02	0.01	0.01	0.02	0.05	9	0	0.00	(-0.01, 0.01)	0.01	-0.01	0.00	0.02
	Visit 8 (Week 12, Day 81)	9	0	0.01	0.01	0.01	0.01	0.02	9	0	-0.00	(-0.01, 0.00)	0.01	-0.01	-0.00	0.00
	Visit 14 (Week 24, Day 165)	9	0	0.01	0.01	0.00	0.01	0.02	9	0	-0.00	(-0.01, 0.00)	0.01	-0.02	-0.00	0.01
	Visit 15 (Week 28, Day 190)	9	0	0.02	0.01	0.01	0.02	0.03	9	0	-0.00	(-0.00, 0.00)	0.00	-0.00	-0.00	0.01

Miss. = Missing (subject attends a visit but measure not recorded)
All units = x10^9/L

Table 11.1.1.1 Summary of FACS Tube 1

Antisense Therapeutics Limited
Protocol: 1102-DMD-CT02
Population: All Enrolled Subjects (excludes Screen Failures) (N=9)

		Actual Values							Change From Baseline (Week 1, Day 1)							
Measure	Visit	n	Miss.	Mean	Std.	Min.	Median	Max.	n	Miss.	Mean	95% CI (for Mean)	Std.	Min.	Median	Max.
CD8	Visit 2 (Week 1, Day 1)	9	0	1.22	0.61	0.39	1.10	2.46								
	Visit 4 (Week 5, Day 29)	9	0	1.30	0.60	0.65	1.13	2.67	9	0	0.08	(-0.09, 0.26)	0.23	-0.31	0.21	0.32
	Visit 6 (Week 8, Day 53)	9	0	1.02	0.65	0.29	0.83	2.23	9	0	-0.20	(-0.53, 0.13)	0.43	-0.91	-0.23	0.42
	Visit 8 (Week 12, Day 81)	9	0	1.13	0.63	0.27	1.17	2.34	9	0	-0.09	(-0.45, 0.26)	0.46	-0.93	-0.10	0.46
	Visit 14 (Week 24, Day 165)	9	0	1.20	0.58	0.51	1.11	2.39	9	0	-0.02	(-0.24, 0.20)	0.29	-0.59	-0.05	0.37
	Visit 15 (Week 28, Day 190)	9	0	1.36	0.74	0.58	1.17	2.92	9	0	0.14	(-0.04, 0.31)	0.23	-0.27	0.19	0.45
CD8 CD49d High	Visit 2 (Week 1, Day 1)	9	0	0.23	0.11	0.08	0.19	0.39								
	Visit 4 (Week 5, Day 29)	9	0	0.25	0.15	0.08	0.23	0.57	9	0	0.03	(-0.05, 0.10)	0.10	-0.10	0.02	0.17
	Visit 6 (Week 8, Day 53)	9	0	0.17	0.17	0.05	0.12	0.59	9	0	-0.05	(-0.15, 0.04)	0.13	-0.22	-0.09	0.19
	Visit 8 (Week 12, Day 81)	9	0	0.22	0.18	0.05	0.18	0.63	9	0	-0.00	(-0.11, 0.10)	0.13	-0.22	-0.00	0.23
	Visit 14 (Week 24, Day 165)	9	0	0.25	0.18	0.08	0.21	0.67	9	0	0.02	(-0.07, 0.11)	0.12	-0.11	-0.01	0.28
	Visit 15 (Week 28, Day 190)	9	0	0.25	0.14	0.10	0.23	0.51	9	0	0.02	(-0.02, 0.07)	0.06	-0.09	0.03	0.12

Miss. = Missing (subject attends a visit but measure not recorded)
All units = x10^9/L

Table 11.1.1.1 Summary of FACS Tube 1

Antisense Therapeutics Limited
Protocol: 1102-DMD-CT02
Population: All Enrolled Subjects (excludes Screen Failures) (N=9)

		Actual Values							Change From Baseline (Week 1, Day 1)							
Measure	Visit	n	Miss.	Mean	Std.	Min.	Median	Max.	n	Miss.	Mean	95% CI (for Mean)	Std.	Min.	Median	Max.
CD8 CD49d+	Visit 2 (Week 1, Day 1)	9	0	1.17	0.58	0.38	1.06	2.40								
	Visit 4 (Week 5, Day 29)	9	0	1.24	0.58	0.64	1.06	2.62	9	0	0.07	(-0.10, 0.25)	0.23	-0.32	0.21	0.30
	Visit 6 (Week 8, Day 53)	9	0	0.94	0.61	0.27	0.80	2.18	9	0	-0.22	(-0.54, 0.09)	0.41	-0.85	-0.22	0.40
	Visit 8 (Week 12, Day 81)	9	0	1.05	0.59	0.26	1.05	2.23	9	0	-0.12	(-0.45, 0.21)	0.43	-0.85	-0.13	0.43
	Visit 14 (Week 24, Day 165)	9	0	1.11	0.50	0.50	1.05	2.18	9	0	-0.05	(-0.27, 0.16)	0.28	-0.57	-0.06	0.33
	Visit 15 (Week 28, Day 190)	9	0	1.28	0.68	0.54	1.15	2.72	9	0	0.11	(-0.06, 0.28)	0.22	-0.30	0.16	0.33
CD8 CD49d Negative	Visit 2 (Week 1, Day 1)	9	0	0.06	0.04	0.01	0.05	0.16								
	Visit 4 (Week 5, Day 29)	9	0	0.07	0.06	0.01	0.05	0.22	9	0	0.01	(-0.01, 0.03)	0.03	-0.03	0.01	0.06
	Visit 6 (Week 8, Day 53)	9	0	0.07	0.10	0.01	0.04	0.34	9	0	0.02	(-0.03, 0.07)	0.07	-0.06	-0.00	0.18
	Visit 8 (Week 12, Day 81)	9	0	0.08	0.06	0.01	0.06	0.18	9	0	0.02	(-0.01, 0.05)	0.04	-0.07	0.03	0.09
	Visit 14 (Week 24, Day 165)	9	0	0.08	0.09	0.01	0.06	0.25	9	0	0.03	(-0.01, 0.07)	0.05	-0.03	0.01	0.13
	Visit 15 (Week 28, Day 190)	9	0	0.08	0.06	0.01	0.05	0.18	9	0	0.02	(-0.01, 0.06)	0.04	-0.04	0.02	0.11

Miss. = Missing (subject attends a visit but measure not recorded)
All units = x10^9/L

Table 11.1.1.1 Summary of FACS Tube 1

Antisense Therapeutics Limited
Protocol: 1102-DMD-CT02
Population: All Enrolled Subjects (excludes Screen Failures) (N=9)

		|------------------- Actual Values-----------------------|							|---------- Change From Baseline (Week 1, Day 1)-----------|							
Measure	Visit	n	Miss.	Mean	Std.	Min.	Median	Max.	n	Miss.	Mean	95% CI (for Mean)	Std.	Min.	Median	Max.
CD8 CD49d Low	Visit 2 (Week 1, Day 1)	9	0	0.94	0.54	0.28	0.85	2.09								
	Visit 4 (Week 5, Day 29)	9	0	0.99	0.55	0.41	0.94	2.24	9	0	0.05	(-0.07, 0.17)	0.15	-0.24	0.11	0.22
	Visit 6 (Week 8, Day 53)	9	0	0.77	0.55	0.22	0.61	1.93	9	0	-0.17	(-0.41, 0.07)	0.31	-0.74	-0.16	0.20
	Visit 8 (Week 12, Day 81)	9	0	0.83	0.52	0.21	0.69	1.89	9	0	-0.11	(-0.36, 0.14)	0.32	-0.75	-0.10	0.24
	Visit 14 (Week 24, Day 165)	9	0	0.86	0.52	0.30	0.85	1.90	9	0	-0.07	(-0.24, 0.09)	0.22	-0.46	-0.05	0.30
	Visit 15 (Week 28, Day 190)	9	0	1.03	0.65	0.30	0.96	2.39	9	0	0.09	(-0.06, 0.24)	0.19	-0.22	0.12	0.31
CD8 HLA-DR Positive	Visit 2 (Week 1, Day 1)	9	0	0.05	0.02	0.02	0.04	0.08								
	Visit 4 (Week 5, Day 29)	9	0	0.05	0.02	0.02	0.05	0.08	9	0	0.00	(-0.02, 0.02)	0.02	-0.04	0.01	0.03
	Visit 6 (Week 8, Day 53)	9	0	0.04	0.02	0.01	0.03	0.08	9	0	-0.01	(-0.03, 0.01)	0.03	-0.05	0.00	0.03
	Visit 8 (Week 12, Day 81)	9	0	0.04	0.02	0.01	0.05	0.08	9	0	-0.00	(-0.03, 0.02)	0.03	-0.04	-0.02	0.04
	Visit 14 (Week 24, Day 165)	9	0	0.04	0.02	0.01	0.04	0.08	9	0	-0.00	(-0.02, 0.01)	0.02	-0.03	0.00	0.03
	Visit 15 (Week 28, Day 190)	9	0	0.04	0.02	0.01	0.04	0.09	9	0	-0.01	(-0.02, 0.01)	0.02	-0.03	-0.00	0.01

Miss. = Missing (subject attends a visit but measure not recorded)
All units = x10^9/L

Table 11.1.1.1 Summary of FACS Tube 1

Antisense Therapeutics Limited
Protocol: 1102-DMD-CT02
Population: All Enrolled Subjects (excludes Screen Failures) (N=9)

		Actual Values							Change From Baseline (Week 1, Day 1)							
Measure	Visit	n	Miss.	Mean	Std.	Min.	Median	Max.	n	Miss.	Mean	95% CI (for Mean)	Std.	Min.	Median	Max.
NK	Visit 2 (Week 1, Day 1)	9	0	0.47	0.30	0.07	0.43	1.03								
	Visit 4 (Week 5, Day 29)	9	0	0.39	0.26	0.04	0.33	0.85	9	0	-0.07	(-0.31, 0.16)	0.30	-0.80	-0.03	0.23
	Visit 6 (Week 8, Day 53)	9	0	0.26	0.17	0.09	0.26	0.60	9	0	-0.21	(-0.42,-0.01)	0.27	-0.77	-0.13	0.03
	Visit 8 (Week 12, Day 81)	9	0	0.28	0.21	0.07	0.19	0.64	9	0	-0.18	(-0.44, 0.07)	0.33	-0.92	-0.17	0.17
	Visit 14 (Week 24, Day 165)	9	0	0.36	0.17	0.14	0.37	0.63	9	0	-0.11	(-0.29, 0.08)	0.24	-0.63	-0.11	0.11
	Visit 15 (Week 28, Day 190)	9	0	0.36	0.22	0.09	0.37	0.65	9	0	-0.10	(-0.32, 0.11)	0.28	-0.82	-0.05	0.13
NK CD49d+	Visit 2 (Week 1, Day 1)	9	0	0.45	0.29	0.07	0.42	0.99								
	Visit 4 (Week 5, Day 29)	9	0	0.38	0.25	0.04	0.33	0.82	9	0	-0.07	(-0.29, 0.16)	0.29	-0.77	-0.03	0.24
	Visit 6 (Week 8, Day 53)	9	0	0.24	0.15	0.09	0.25	0.52	9	0	-0.21	(-0.41,-0.01)	0.26	-0.75	-0.13	0.03
	Visit 8 (Week 12, Day 81)	9	0	0.27	0.20	0.07	0.19	0.62	9	0	-0.18	(-0.42, 0.07)	0.32	-0.88	-0.17	0.15
	Visit 14 (Week 24, Day 165)	9	0	0.35	0.16	0.14	0.36	0.61	9	0	-0.10	(-0.28, 0.08)	0.23	-0.61	-0.11	0.11
	Visit 15 (Week 28, Day 190)	9	0	0.35	0.21	0.09	0.37	0.63	9	0	-0.10	(-0.31, 0.12)	0.28	-0.80	-0.04	0.13

Miss. = Missing (subject attends a visit but measure not recorded)
All units = x10^9/L

Table 11.1.1.1 Summary of FACS Tube 1

Antisense Therapeutics Limited
Protocol: 1102-DMD-CT02
Population: All Enrolled Subjects (excludes Screen Failures) (N=9)

		Actual Values							Change From Baseline (Week 1, Day 1)							
Measure	Visit	n	Miss.	Mean	Std.	Min.	Median	Max.	n	Miss.	Mean	95% CI (for Mean)	Std.	Min.	Median	Max.
NK CD49d-	Visit 2 (Week 1, Day 1)	9	0	0.02	0.01	0.00	0.01	0.04								
	Visit 4 (Week 5, Day 29)	9	0	0.01	0.01	0.00	0.01	0.02	9	0	-0.01	(-0.01, 0.00)	0.01	-0.03	-0.01	0.01
	Visit 6 (Week 8, Day 53)	9	0	0.02	0.03	0.00	0.01	0.08	9	0	0.00	(-0.01, 0.02)	0.02	-0.02	-0.00	0.05
	Visit 8 (Week 12, Day 81)	9	0	0.01	0.01	0.00	0.00	0.03	9	0	-0.01	(-0.02, 0.00)	0.01	-0.04	-0.01	0.01
	Visit 14 (Week 24, Day 165)	9	0	0.01	0.01	0.00	0.01	0.02	9	0	-0.01	(-0.01, 0.00)	0.01	-0.02	-0.01	0.00
	Visit 15 (Week 28, Day 190)	9	0	0.01	0.01	0.00	0.01	0.02	9	0	-0.01	(-0.02,-0.00)	0.01	-0.03	-0.01	0.01
Lymphocytes	Visit 2 (Week 1, Day 1)	9	0	3.68	1.47	1.03	4.00	6.16								
	Visit 4 (Week 5, Day 29)	9	0	3.88	1.38	1.54	4.01	6.73	9	0	0.19	(-0.41, 0.79)	0.78	-1.18	0.51	1.00
	Visit 6 (Week 8, Day 53)	9	0	3.12	1.38	1.12	3.33	5.53	9	0	-0.56	(-1.52, 0.40)	1.25	-2.88	-0.20	0.83
	Visit 8 (Week 12, Day 81)	9	0	3.15	1.44	0.96	3.25	5.72	9	0	-0.53	(-1.65, 0.58)	1.45	-3.04	-0.44	1.23
	Visit 14 (Week 24, Day 165)	9	0	3.41	1.29	1.63	3.52	5.90	9	0	-0.28	(-1.10, 0.55)	1.07	-2.60	-0.23	1.18
	Visit 15 (Week 28, Day 190)	9	0	3.87	1.66	1.51	4.00	7.20	9	0	0.19	(-0.44, 0.81)	0.81	-1.55	0.48	1.04

Miss. = Missing (subject attends a visit but measure not recorded)
All units = x10^9/L

Table 11.1.1.1 Summary of FACS Tube 1

Antisense Therapeutics Limited
Protocol: 1102-DMD-CT02
Population: All Enrolled Subjects (excludes Screen Failures) (N=9)

		Actual Values							Change From Baseline (Week 1, Day 1)							
Measure	Visit	n	Miss.	Mean	Std.	Min.	Median	Max.	n	Miss.	Mean	95% CI (for Mean)	Std.	Min.	Median	Max.
Percent Parent Lymphocytes	Visit 2 (Week 1, Day 1)	9	0	34.84	8.45	18.38	36.63	46.40								
	Visit 4 (Week 5, Day 29)	9	0	34.47	7.79	23.81	34.06	47.65	9	0	-0.37	(-5.87, 5.13)	7.15	-12.8	1.25	8.02
	Visit 6 (Week 8, Day 53)	9	0	28.11	11.16	10.78	32.04	45.31	9	0	-6.73	(-12.5,-0.91)	7.57	-22.2	-3.87	-0.01
	Visit 8 (Week 12, Day 81)	9	0	31.89	10.83	10.59	32.10	44.90	9	0	-2.95	(-9.46, 3.56)	8.47	-22.3	-1.50	6.06
	Visit 14 (Week 24, Day 165)	9	0	31.17	12.31	14.30	34.12	51.34	9	0	-3.67	(-10.5, 3.13)	8.84	-22.3	-0.06	4.94
	Visit 15 (Week 28, Day 190)	9	0	32.59	12.14	14.04	35.29	52.00	9	0	-2.25	(-7.73, 3.23)	7.13	-18.4	-0.89	5.60
Percent Parent CD4+	Visit 2 (Week 1, Day 1)	9	0	53.13	8.63	36.91	56.79	64.07								
	Visit 4 (Week 5, Day 29)	9	0	53.96	10.92	38.11	57.68	67.50	9	0	0.83	(-1.90, 3.55)	3.55	-6.85	1.20	6.15
	Visit 6 (Week 8, Day 53)	9	0	53.21	10.83	39.88	49.54	68.45	9	0	0.08	(-4.10, 4.25)	5.43	-9.06	0.81	9.06
	Visit 8 (Week 12, Day 81)	9	0	52.55	10.38	37.00	51.69	68.27	9	0	-0.59	(-3.33, 2.16)	3.58	-6.91	0.09	4.20
	Visit 14 (Week 24, Day 165)	9	0	51.69	10.13	38.75	55.56	63.05	9	0	-1.45	(-4.43, 1.53)	3.88	-9.95	-1.02	2.74
	Visit 15 (Week 28, Day 190)	9	0	52.33	10.88	37.80	57.18	67.07	9	0	-0.80	(-4.33, 2.73)	4.60	-11.8	-0.25	4.33

Miss. = Missing (subject attends a visit but measure not recorded)
All units = x10^9/L

Table 11.1.1.1 Summary of FACS Tube 1

Antisense Therapeutics Limited
Protocol: 1102-DMD-CT02
Population: All Enrolled Subjects (excludes Screen Failures) (N=9)

		Actual Values							Change From Baseline (Week 1, Day 1)							
Measure	Visit	n	Miss.	Mean	Std.	Min.	Median	Max.	n	Miss.	Mean	95% CI (for Mean)	Std.	Min.	Median	Max.
Percent Parent CD8+	Visit 2 (Week 1, Day 1)	9	0	41.96	8.15	31.36	39.79	55.86								
	Visit 4 (Week 5, Day 29)	9	0	41.94	10.28	29.84	38.66	59.23	9	0	-0.02	(-2.43, 2.39)	3.13	-3.45	-1.11	7.38
	Visit 6 (Week 8, Day 53)	9	0	42.16	9.77	28.75	46.06	54.18	9	0	0.20	(-3.59, 3.99)	4.93	-5.82	-0.84	8.71
	Visit 8 (Week 12, Day 81)	9	0	43.34	9.40	28.79	43.56	56.52	9	0	1.38	(-1.00, 3.75)	3.09	-2.57	-0.03	6.86
	Visit 14 (Week 24, Day 165)	9	0	43.44	8.89	31.64	40.93	56.69	9	0	1.47	(-0.87, 3.82)	3.05	-1.93	0.67	7.68
	Visit 15 (Week 28, Day 190)	9	0	42.92	9.10	29.43	39.53	55.10	9	0	0.96	(-1.67, 3.59)	3.42	-1.93	-0.46	9.45

Miss. = Missing (subject attends a visit but measure not recorded)
All units = x10^9/L

Table 11.1.1.1.2 Summary of FACS Tube 1

Antisense Therapeutics Limited
Protocol: 1102-DMD-CT02
Population: All Enrolled Subjects (excludes Screen Failures) (N=9)

		-------- Change From Baseline** (Week 1, Day 1)---------									
Measure	Visit	n	Miss.	Mean	95% CI (for Mean)	Std.	Min.	Median	Max.	P-value (Paired T-Test)	P-Value (WSRT)
CD3	Visit 2 (Week 1, Day 1)										
	Visit 4 (Week 5, Day 29)	9	0	0.22	(-0.17, 0.61)	0.51	-0.59	0.28	0.90	0.227	0.250
	Visit 6 (Week 8, Day 53)	9	0	-0.53	(-1.34, 0.27)	1.05	-2.58	-0.56	0.63	0.166	0.250
	Visit 8 (Week 12, Day 81)	9	0	-0.33	(-1.24, 0.58)	1.19	-2.59	-0.18	0.95	0.430	0.734
	Visit 14 (Week 24, Day 165)	9	0	-0.18	(-0.79, 0.43)	0.80	-1.90	0.03	0.91	0.516	0.910
	Visit 15 (Week 28, Day 190)	9	0	0.25	(-0.17, 0.66)	0.54	-0.69	0.33	0.87	0.205	0.301
	Change Week 24 to Week 28	9	0	0.43	(-0.01, 0.87)	0.58	-0.40	0.48	1.21	0.056	0.055
CD3 CD49d+	Visit 2 (Week 1, Day 1)										
	Visit 4 (Week 5, Day 29)	9	0	0.16	(-0.17, 0.48)	0.42	-0.56	0.29	0.67	0.296	0.301
	Visit 6 (Week 8, Day 53)	9	0	-0.50	(-1.17, 0.17)	0.87	-2.13	-0.33	0.55	0.123	0.203
	Visit 8 (Week 12, Day 81)	9	0	-0.39	(-1.12, 0.33)	0.94	-2.11	-0.27	0.69	0.245	0.496
	Visit 14 (Week 24, Day 165)	9	0	-0.28	(-0.78, 0.21)	0.64	-1.58	-0.21	0.61	0.221	0.250
	Visit 15 (Week 28, Day 190)	9	0	0.11	(-0.26, 0.48)	0.48	-0.74	0.24	0.82	0.502	0.496
	Change Week 24 to Week 28	9	0	0.40	(0.05, 0.74)	0.45	-0.32	0.32	1.11	0.030	0.055

Miss. = Missing (subject attends a visit but measure not recorded)
All units = x10^9/L; WSRT=Wilcoxon Sign-Rank Test
** Change from Baseline other than the Row 'Change Week 24 to Week 28'

Table 11.1.1.1.2 Summary of FACS Tube 1

Antisense Therapeutics Limited
Protocol: 1102-DMD-CT02
Population: All Enrolled Subjects (excludes Screen Failures) (N=9)

		-------- Change From Baseline** (Week 1, Day 1)---------									
Measure	Visit	n	Miss.	Mean	95% CI (for Mean)	Std.	Min.	Median	Max.	P-value (Paired T-Test)	P-Value (WSRT)
CD3 CD49d-	Visit 2 (Week 1, Day 1)										
	Visit 4 (Week 5, Day 29)	9	0	0.07	(-0.08, 0.22)	0.19	-0.21	0.05	0.35	0.309	0.496
	Visit 6 (Week 8, Day 53)	9	0	-0.02	(-0.22, 0.18)	0.26	-0.45	0.02	0.47	0.812	0.820
	Visit 8 (Week 12, Day 81)	9	0	0.05	(-0.19, 0.30)	0.32	-0.49	0.01	0.53	0.631	0.820
	Visit 14 (Week 24, Day 165)	9	0	0.09	(-0.09, 0.27)	0.24	-0.33	0.04	0.36	0.294	0.426
	Visit 15 (Week 28, Day 190)	9	0	0.13	(-0.03, 0.30)	0.21	-0.21	0.14	0.49	0.096	0.129
	Change Week 24 to Week 28	9	0	0.05	(-0.15, 0.24)	0.26	-0.50	0.08	0.38	0.615	0.426
CD3-	Visit 2 (Week 1, Day 1)										
	Visit 4 (Week 5, Day 29)	9	0	-0.02	(-0.28, 0.23)	0.33	-0.79	0.07	0.29	0.847	0.820
	Visit 6 (Week 8, Day 53)	9	0	-0.11	(-0.41, 0.19)	0.39	-0.76	-0.08	0.57	0.434	0.496
	Visit 8 (Week 12, Day 81)	9	0	-0.20	(-0.49, 0.09)	0.38	-0.91	-0.28	0.28	0.156	0.164
	Visit 14 (Week 24, Day 165)	9	0	-0.09	(-0.32, 0.14)	0.30	-0.68	-0.11	0.28	0.404	0.496
	Visit 15 (Week 28, Day 190)	9	0	-0.06	(-0.31, 0.19)	0.33	-0.84	0.04	0.28	0.609	0.910
	Change Week 24 to Week 28	9	0	0.03	(-0.06, 0.12)	0.12	-0.16	0.03	0.20	0.454	0.426

Miss. = Missing (subject attends a visit but measure not recorded)
All units = x10^9/L; WSRT=Wilcoxon Sign-Rank Test
** Change from Baseline other than the Row 'Change Week 24 to Week 28'

Table 11.1.1.1.2 Summary of FACS Tube 1

Antisense Therapeutics Limited
Protocol: 1102-DMD-CT02
Population: All Enrolled Subjects (excludes Screen Failures) (N=9)

		-------- Change From Baseline** (Week 1, Day 1)---------									
Measure	Visit	n	Miss.	Mean	95% CI (for Mean)	Std.	Min.	Median	Max.	P-value (Paired T-Test)	P-Value (WSRT)
CD4	Visit 2 (Week 1, Day 1)										
	Visit 4 (Week 5, Day 29)	9	0	0.15	(-0.05, 0.35)	0.26	-0.18	0.08	0.54	0.119	0.203
	Visit 6 (Week 8, Day 53)	9	0	-0.30	(-0.76, 0.15)	0.59	-1.57	-0.30	0.30	0.160	0.203
	Visit 8 (Week 12, Day 81)	9	0	-0.20	(-0.72, 0.32)	0.68	-1.56	-0.06	0.57	0.393	0.652
	Visit 14 (Week 24, Day 165)	9	0	-0.15	(-0.52, 0.23)	0.48	-1.23	-0.02	0.52	0.394	0.570
	Visit 15 (Week 28, Day 190)	9	0	0.11	(-0.12, 0.34)	0.30	-0.37	0.16	0.41	0.294	0.301
	Change Week 24 to Week 28	9	0	0.26	(-0.02, 0.53)	0.36	-0.19	0.20	0.87	0.063	0.055
CD4 CD49d High	Visit 2 (Week 1, Day 1)										
	Visit 4 (Week 5, Day 29)	9	0	0.04	(-0.01, 0.09)	0.06	-0.02	0.05	0.16	0.088	0.164
	Visit 6 (Week 8, Day 53)	9	0	-0.05	(-0.12, 0.03)	0.10	-0.18	-0.03	0.09	0.177	0.301
	Visit 8 (Week 12, Day 81)	9	0	-0.02	(-0.11, 0.07)	0.12	-0.26	0.03	0.10	0.595	0.820
	Visit 14 (Week 24, Day 165)	9	0	-0.01	(-0.06, 0.04)	0.06	-0.09	-0.02	0.08	0.659	0.734
	Visit 15 (Week 28, Day 190)	9	0	0.01	(-0.02, 0.05)	0.04	-0.04	0.01	0.08	0.303	0.250
	Change Week 24 to Week 28	9	0	0.02	(-0.01, 0.06)	0.05	-0.05	0.03	0.10	0.183	0.301

Miss. = Missing (subject attends a visit but measure not recorded)
All units = x10^9/L; WSRT=Wilcoxon Sign-Rank Test
** Change from Baseline other than the Row 'Change Week 24 to Week 28'

Table 11.1.1.1.2 Summary of FACS Tube 1

Antisense Therapeutics Limited
Protocol: 1102-DMD-CT02
Population: All Enrolled Subjects (excludes Screen Failures) (N=9)

		-------- Change From Baseline** (Week 1, Day 1)---------									
Measure	Visit	n	Miss.	Mean	95% CI (for Mean)	Std.	Min.	Median	Max.	P-value (Paired T-Test)	P-Value (WSRT)
CD4 CD49d+	Visit 2 (Week 1, Day 1)										
	Visit 4 (Week 5, Day 29)	9	0	0.08	(-0.06, 0.22)	0.18	-0.19	0.09	0.35	0.230	0.359
	Visit 6 (Week 8, Day 53)	9	0	-0.28	(-0.64, 0.08)	0.47	-1.28	-0.12	0.18	0.114	0.164
	Visit 8 (Week 12, Day 81)	9	0	-0.22	(-0.63, 0.18)	0.53	-1.25	-0.13	0.32	0.236	0.570
	Visit 14 (Week 24, Day 165)	9	0	-0.19	(-0.48, 0.10)	0.38	-1.06	-0.15	0.26	0.174	0.203
	Visit 15 (Week 28, Day 190)	9	0	0.01	(-0.20, 0.21)	0.27	-0.36	0.03	0.53	0.924	0.820
	Change Week 24 to Week 28	9	0	0.20	(-0.02, 0.42)	0.29	-0.16	0.22	0.70	0.073	0.074
CD4 CD49d Negative	Visit 2 (Week 1, Day 1)										
	Visit 4 (Week 5, Day 29)	9	0	0.07	(-0.03, 0.17)	0.13	-0.08	0.04	0.29	0.126	0.203
	Visit 6 (Week 8, Day 53)	9	0	-0.02	(-0.14, 0.11)	0.16	-0.27	-0.02	0.28	0.751	0.652
	Visit 8 (Week 12, Day 81)	9	0	0.02	(-0.13, 0.17)	0.19	-0.30	0.02	0.39	0.747	0.652
	Visit 14 (Week 24, Day 165)	9	0	0.04	(-0.06, 0.15)	0.14	-0.16	0.01	0.26	0.385	0.359
	Visit 15 (Week 28, Day 190)	9	0	0.10	(-0.04, 0.24)	0.18	-0.21	0.11	0.45	0.126	0.129
	Change Week 24 to Week 28	9	0	0.06	(-0.13, 0.25)	0.25	-0.47	0.08	0.45	0.485	0.359

Miss. = Missing (subject attends a visit but measure not recorded)
All units = x10^9/L; WSRT=Wilcoxon Sign-Rank Test
** Change from Baseline other than the Row 'Change Week 24 to Week 28'

Table 11.1.1.1.2 Summary of FACS Tube 1

Antisense Therapeutics Limited
Protocol: 1102-DMD-CT02
Population: All Enrolled Subjects (excludes Screen Failures) (N=9)

		-------- Change From Baseline** (Week 1, Day 1)---------									
Measure	Visit	n	Miss.	Mean	95% CI (for Mean)	Std.	Min.	Median	Max.	P-value (Paired T-Test)	P-Value (WSRT)
CD4 CD49d Low	Visit 2 (Week 1, Day 1)										
	Visit 4 (Week 5, Day 29)	9	0	0.05	(-0.05, 0.14)	0.12	-0.14	0.05	0.21	0.289	0.301
	Visit 6 (Week 8, Day 53)	9	0	-0.23	(-0.53, 0.07)	0.39	-1.12	-0.09	0.13	0.116	0.164
	Visit 8 (Week 12, Day 81)	9	0	-0.20	(-0.53, 0.13)	0.43	-1.09	-0.11	0.21	0.202	0.301
	Visit 14 (Week 24, Day 165)	9	0	-0.17	(-0.43, 0.08)	0.33	-0.95	-0.12	0.18	0.150	0.129
	Visit 15 (Week 28, Day 190)	9	0	-0.01	(-0.20, 0.19)	0.25	-0.38	-0.05	0.49	0.939	0.910
	Change Week 24 to Week 28	9	0	0.17	(-0.02, 0.36)	0.25	-0.15	0.17	0.57	0.075	0.074
CD4 HLA-DR Positive	Visit 2 (Week 1, Day 1)										
	Visit 4 (Week 5, Day 29)	9	0	0.00	(-0.00, 0.01)	0.01	-0.01	0.00	0.01	0.787	0.910
	Visit 6 (Week 8, Day 53)	9	0	0.00	(-0.01, 0.01)	0.01	-0.01	0.00	0.02	0.808	0.910
	Visit 8 (Week 12, Day 81)	9	0	-0.00	(-0.01, 0.00)	0.01	-0.01	-0.00	0.00	0.149	0.301
	Visit 14 (Week 24, Day 165)	9	0	-0.00	(-0.01, 0.00)	0.01	-0.02	-0.00	0.01	0.184	0.203
	Visit 15 (Week 28, Day 190)	9	0	-0.00	(-0.00, 0.00)	0.00	-0.00	-0.00	0.01	0.562	0.570
	Change Week 24 to Week 28	9	0	0.00	(-0.00, 0.01)	0.01	-0.01	0.00	0.01	0.204	0.301

Miss. = Missing (subject attends a visit but measure not recorded)
All units = x10^9/L; WSRT=Wilcoxon Sign-Rank Test
** Change from Baseline other than the Row 'Change Week 24 to Week 28'

Table 11.1.1.1.2 Summary of FACS Tube 1

Antisense Therapeutics Limited
Protocol: 1102-DMD-CT02
Population: All Enrolled Subjects (excludes Screen Failures) (N=9)

		-------- Change From Baseline** (Week 1, Day 1)---------									
Measure	Visit	n	Miss.	Mean	95% CI (for Mean)	Std.	Min.	Median	Max.	P-value (Paired T-Test)	P-Value (WSRT)
CD8	Visit 2 (Week 1, Day 1)										
	Visit 4 (Week 5, Day 29)	9	0	0.08	(-0.09, 0.26)	0.23	-0.31	0.21	0.32	0.309	0.301
	Visit 6 (Week 8, Day 53)	9	0	-0.20	(-0.53, 0.13)	0.43	-0.91	-0.23	0.42	0.194	0.203
	Visit 8 (Week 12, Day 81)	9	0	-0.09	(-0.45, 0.26)	0.46	-0.93	-0.10	0.46	0.558	0.652
	Visit 14 (Week 24, Day 165)	9	0	-0.02	(-0.24, 0.20)	0.29	-0.59	-0.05	0.37	0.834	1.000
	Visit 15 (Week 28, Day 190)	9	0	0.14	(-0.04, 0.31)	0.23	-0.27	0.19	0.45	0.105	0.129
	Change Week 24 to Week 28	9	0	0.16	(-0.01, 0.33)	0.23	-0.16	0.17	0.52	0.068	0.074
CD8 CD49d High	Visit 2 (Week 1, Day 1)										
	Visit 4 (Week 5, Day 29)	9	0	0.03	(-0.05, 0.10)	0.10	-0.10	0.02	0.17	0.453	0.496
	Visit 6 (Week 8, Day 53)	9	0	-0.05	(-0.15, 0.04)	0.13	-0.22	-0.09	0.19	0.251	0.250
	Visit 8 (Week 12, Day 81)	9	0	-0.00	(-0.11, 0.10)	0.13	-0.22	-0.00	0.23	0.930	0.910
	Visit 14 (Week 24, Day 165)	9	0	0.02	(-0.07, 0.11)	0.12	-0.11	-0.01	0.28	0.628	0.734
	Visit 15 (Week 28, Day 190)	9	0	0.02	(-0.02, 0.07)	0.06	-0.09	0.03	0.12	0.244	0.164
	Change Week 24 to Week 28	9	0	0.00	(-0.06, 0.07)	0.09	-0.16	0.02	0.14	0.905	0.652

Miss. = Missing (subject attends a visit but measure not recorded)
All units = x10^9/L; WSRT=Wilcoxon Sign-Rank Test
** Change from Baseline other than the Row 'Change Week 24 to Week 28'

Table 11.1.1.1.2 Summary of FACS Tube 1

Antisense Therapeutics Limited
Protocol: 1102-DMD-CT02
Population: All Enrolled Subjects (excludes Screen Failures) (N=9)

		-------- Change From Baseline** (Week 1, Day 1)---------									
Measure	Visit	n	Miss.	Mean	95% CI (for Mean)	Std.	Min.	Median	Max.	P-value (Paired T-Test)	P-Value (WSRT)
CD8 CD49d+	Visit 2 (Week 1, Day 1)										
	Visit 4 (Week 5, Day 29)	9	0	0.07	(-0.10, 0.25)	0.23	-0.32	0.21	0.30	0.368	0.359
	Visit 6 (Week 8, Day 53)	9	0	-0.22	(-0.54, 0.09)	0.41	-0.85	-0.22	0.40	0.144	0.129
	Visit 8 (Week 12, Day 81)	9	0	-0.12	(-0.45, 0.21)	0.43	-0.85	-0.13	0.43	0.439	0.652
	Visit 14 (Week 24, Day 165)	9	0	-0.05	(-0.27, 0.16)	0.28	-0.57	-0.06	0.33	0.577	0.734
	Visit 15 (Week 28, Day 190)	9	0	0.11	(-0.06, 0.28)	0.22	-0.30	0.16	0.33	0.164	0.129
	Change Week 24 to Week 28	9	0	0.17	(-0.01, 0.34)	0.23	-0.16	0.15	0.54	0.064	0.074
CD8 CD49d Negative	Visit 2 (Week 1, Day 1)										
	Visit 4 (Week 5, Day 29)	9	0	0.01	(-0.01, 0.03)	0.03	-0.03	0.01	0.06	0.337	0.496
	Visit 6 (Week 8, Day 53)	9	0	0.02	(-0.03, 0.07)	0.07	-0.06	-0.00	0.18	0.451	1.000
	Visit 8 (Week 12, Day 81)	9	0	0.02	(-0.01, 0.05)	0.04	-0.07	0.03	0.09	0.224	0.164
	Visit 14 (Week 24, Day 165)	9	0	0.03	(-0.01, 0.07)	0.05	-0.03	0.01	0.13	0.145	0.203
	Visit 15 (Week 28, Day 190)	9	0	0.02	(-0.01, 0.06)	0.04	-0.04	0.02	0.11	0.125	0.098
	Change Week 24 to Week 28	9	0	-0.01	(-0.04, 0.03)	0.04	-0.08	0.01	0.04	0.728	1.000

Miss. = Missing (subject attends a visit but measure not recorded)
All units = x10^9/L; WSRT=Wilcoxon Sign-Rank Test
** Change from Baseline other than the Row 'Change Week 24 to Week 28'

Table 11.1.1.1.2 Summary of FACS Tube 1

Antisense Therapeutics Limited
Protocol: 1102-DMD-CT02
Population: All Enrolled Subjects (excludes Screen Failures) (N=9)

		-------- Change From Baseline** (Week 1, Day 1)---------									
Measure	Visit	n	Miss.	Mean	95% CI (for Mean)	Std.	Min.	Median	Max.	P-value (Paired T-Test)	P-Value (WSRT)
CD8 CD49d Low	Visit 2 (Week 1, Day 1)										
	Visit 4 (Week 5, Day 29)	9	0	0.05	(-0.07, 0.17)	0.15	-0.24	0.11	0.22	0.372	0.359
	Visit 6 (Week 8, Day 53)	9	0	-0.17	(-0.41, 0.07)	0.31	-0.74	-0.16	0.20	0.137	0.203
	Visit 8 (Week 12, Day 81)	9	0	-0.11	(-0.36, 0.14)	0.32	-0.75	-0.10	0.24	0.331	0.570
	Visit 14 (Week 24, Day 165)	9	0	-0.07	(-0.24, 0.09)	0.22	-0.46	-0.05	0.30	0.336	0.359
	Visit 15 (Week 28, Day 190)	9	0	0.09	(-0.06, 0.24)	0.19	-0.22	0.12	0.31	0.209	0.203
	Change Week 24 to Week 28	9	0	0.16	(0.04, 0.29)	0.16	0.00	0.13	0.49	0.018	0.004
CD8 HLA-DR Positive	Visit 2 (Week 1, Day 1)										
	Visit 4 (Week 5, Day 29)	9	0	0.00	(-0.02, 0.02)	0.02	-0.04	0.01	0.03	0.778	0.734
	Visit 6 (Week 8, Day 53)	9	0	-0.01	(-0.03, 0.01)	0.03	-0.05	0.00	0.03	0.349	0.496
	Visit 8 (Week 12, Day 81)	9	0	-0.00	(-0.03, 0.02)	0.03	-0.04	-0.02	0.04	0.628	0.496
	Visit 14 (Week 24, Day 165)	9	0	-0.00	(-0.02, 0.01)	0.02	-0.03	0.00	0.03	0.665	0.734
	Visit 15 (Week 28, Day 190)	9	0	-0.01	(-0.02, 0.01)	0.02	-0.03	-0.00	0.01	0.248	0.359
	Change Week 24 to Week 28	9	0	-0.00	(-0.02, 0.01)	0.01	-0.03	0.00	0.01	0.417	0.820

Miss. = Missing (subject attends a visit but measure not recorded)
All units = x10^9/L; WSRT=Wilcoxon Sign-Rank Test
** Change from Baseline other than the Row 'Change Week 24 to Week 28'

Table 11.1.1.1.2 Summary of FACS Tube 1

Antisense Therapeutics Limited
Protocol: 1102-DMD-CT02
Population: All Enrolled Subjects (excludes Screen Failures) (N=9)

		-------- Change From Baseline** (Week 1, Day 1)---------									
Measure	Visit	n	Miss.	Mean	95% CI (for Mean)	Std.	Min.	Median	Max.	P-value (Paired T-Test)	P-Value (WSRT)
NK	Visit 2 (Week 1, Day 1)										
	Visit 4 (Week 5, Day 29)	9	0	-0.07	(-0.31, 0.16)	0.30	-0.80	-0.03	0.23	0.476	0.652
	Visit 6 (Week 8, Day 53)	9	0	-0.21	(-0.42,-0.01)	0.27	-0.77	-0.13	0.03	0.045	0.027
	Visit 8 (Week 12, Day 81)	9	0	-0.18	(-0.44, 0.07)	0.33	-0.92	-0.17	0.17	0.133	0.129
	Visit 14 (Week 24, Day 165)	9	0	-0.11	(-0.29, 0.08)	0.24	-0.63	-0.11	0.11	0.213	0.164
	Visit 15 (Week 28, Day 190)	9	0	-0.10	(-0.32, 0.11)	0.28	-0.82	-0.05	0.13	0.299	0.426
	Change Week 24 to Week 28	9	0	0.00	(-0.08, 0.09)	0.11	-0.19	0.02	0.16	0.910	0.734
NK CD49d+	Visit 2 (Week 1, Day 1)										
	Visit 4 (Week 5, Day 29)	9	0	-0.07	(-0.29, 0.16)	0.29	-0.77	-0.03	0.24	0.498	0.820
	Visit 6 (Week 8, Day 53)	9	0	-0.21	(-0.41,-0.01)	0.26	-0.75	-0.13	0.03	0.039	0.020
	Visit 8 (Week 12, Day 81)	9	0	-0.18	(-0.42, 0.07)	0.32	-0.88	-0.17	0.15	0.135	0.129
	Visit 14 (Week 24, Day 165)	9	0	-0.10	(-0.28, 0.08)	0.23	-0.61	-0.11	0.11	0.221	0.203
	Visit 15 (Week 28, Day 190)	9	0	-0.10	(-0.31, 0.12)	0.28	-0.80	-0.04	0.13	0.322	0.570
	Change Week 24 to Week 28	9	0	0.01	(-0.08, 0.09)	0.11	-0.19	0.02	0.17	0.877	0.820

Miss. = Missing (subject attends a visit but measure not recorded)
All units = x10^9/L; WSRT=Wilcoxon Sign-Rank Test
** Change from Baseline other than the Row 'Change Week 24 to Week 28'

Table 11.1.1.1.2 Summary of FACS Tube 1

Antisense Therapeutics Limited
Protocol: 1102-DMD-CT02
Population: All Enrolled Subjects (excludes Screen Failures) (N=9)

		-------- Change From Baseline** (Week 1, Day 1)---------									
Measure	Visit	n	Miss.	Mean	95% CI (for Mean)	Std.	Min.	Median	Max.	P-value (Paired T-Test)	P-Value (WSRT)
NK CD49d-	Visit 2 (Week 1, Day 1)										
	Visit 4 (Week 5, Day 29)	9	0	-0.01	(-0.01, 0.00)	0.01	-0.03	-0.01	0.01	0.123	0.074
	Visit 6 (Week 8, Day 53)	9	0	0.00	(-0.01, 0.02)	0.02	-0.02	-0.00	0.05	0.938	0.129
	Visit 8 (Week 12, Day 81)	9	0	-0.01	(-0.02, 0.00)	0.01	-0.04	-0.01	0.01	0.116	0.098
	Visit 14 (Week 24, Day 165)	9	0	-0.01	(-0.01, 0.00)	0.01	-0.02	-0.01	0.00	0.089	0.098
	Visit 15 (Week 28, Day 190)	9	0	-0.01	(-0.02,-0.00)	0.01	-0.03	-0.01	0.01	0.037	0.027
	Change Week 24 to Week 28	9	0	-0.00	(-0.00, 0.00)	0.00	-0.01	-0.00	0.00	0.050	0.055
Lymphocytes	Visit 2 (Week 1, Day 1)										
	Visit 4 (Week 5, Day 29)	9	0	0.19	(-0.41, 0.79)	0.78	-1.18	0.51	1.00	0.476	0.570
	Visit 6 (Week 8, Day 53)	9	0	-0.56	(-1.52, 0.40)	1.25	-2.88	-0.20	0.83	0.215	0.426
	Visit 8 (Week 12, Day 81)	9	0	-0.53	(-1.65, 0.58)	1.45	-3.04	-0.44	1.23	0.302	0.426
	Visit 14 (Week 24, Day 165)	9	0	-0.28	(-1.10, 0.55)	1.07	-2.60	-0.23	1.18	0.460	0.570
	Visit 15 (Week 28, Day 190)	9	0	0.19	(-0.44, 0.81)	0.81	-1.55	0.48	1.04	0.511	0.301
	Change Week 24 to Week 28	9	0	0.46	(-0.00, 0.93)	0.61	-0.46	0.59	1.30	0.051	0.055

Miss. = Missing (subject attends a visit but measure not recorded)
All units = x10^9/L; WSRT=Wilcoxon Sign-Rank Test
** Change from Baseline other than the Row 'Change Week 24 to Week 28'

Table 11.1.1.1.2 Summary of FACS Tube 1

Antisense Therapeutics Limited
Protocol: 1102-DMD-CT02
Population: All Enrolled Subjects (excludes Screen Failures) (N=9)

		-------- Change From Baseline** (Week 1, Day 1)---------									
Measure	Visit	n	Miss.	Mean	95% CI (for Mean)	Std.	Min.	Median	Max.	P-value (Paired T-Test)	P-Value (WSRT)
Percent Parent Lymphocytes	Visit 2 (Week 1, Day 1)										
	Visit 4 (Week 5, Day 29)	9	0	-0.37	(-5.87, 5.13)	7.15	-12.8	1.25	8.02	0.881	0.910
	Visit 6 (Week 8, Day 53)	9	0	-6.73	(-12.5,-0.91)	7.57	-22.2	-3.87	-0.01	0.028	0.004
	Visit 8 (Week 12, Day 81)	9	0	-2.95	(-9.46, 3.56)	8.47	-22.3	-1.50	6.06	0.326	0.512
	Visit 14 (Week 24, Day 165)	9	0	-3.67	(-10.5, 3.13)	8.84	-22.3	-0.06	4.94	0.248	0.496
	Visit 15 (Week 28, Day 190)	9	0	-2.25	(-7.73, 3.23)	7.13	-18.4	-0.89	5.60	0.372	0.570
	Change Week 24 to Week 28	9	0	1.43	(-3.51, 6.36)	6.42	-6.56	0.66	15.40	0.524	0.820
Percent Parent CD4+	Visit 2 (Week 1, Day 1)										
	Visit 4 (Week 5, Day 29)	9	0	0.83	(-1.90, 3.55)	3.55	-6.85	1.20	6.15	0.505	0.359
	Visit 6 (Week 8, Day 53)	9	0	0.08	(-4.10, 4.25)	5.43	-9.06	0.81	9.06	0.968	0.762
	Visit 8 (Week 12, Day 81)	9	0	-0.59	(-3.33, 2.16)	3.58	-6.91	0.09	4.20	0.636	0.711
	Visit 14 (Week 24, Day 165)	9	0	-1.45	(-4.43, 1.53)	3.88	-9.95	-1.02	2.74	0.295	0.570
	Visit 15 (Week 28, Day 190)	9	0	-0.80	(-4.33, 2.73)	4.60	-11.8	-0.25	4.33	0.615	0.910
	Change Week 24 to Week 28	9	0	0.64	(-0.97, 2.26)	2.11	-1.89	1.21	4.02	0.386	0.496

Miss. = Missing (subject attends a visit but measure not recorded)
All units = x10^9/L; WSRT=Wilcoxon Sign-Rank Test
** Change from Baseline other than the Row 'Change Week 24 to Week 28'

Table 11.1.1.1.2 Summary of FACS Tube 1

Antisense Therapeutics Limited
Protocol: 1102-DMD-CT02
Population: All Enrolled Subjects (excludes Screen Failures) (N=9)

		-------- Change From Baseline** (Week 1, Day 1)---------									
Measure	Visit	n	Miss.	Mean	95% CI (for Mean)	Std.	Min.	Median	Max.	P-value (Paired T-Test)	P-Value (WSRT)
Percent Parent CD8+	Visit 2 (Week 1, Day 1)										
	Visit 4 (Week 5, Day 29)	9	0	-0.02	(-2.43, 2.39)	3.13	-3.45	-1.11	7.38	0.985	0.496
	Visit 6 (Week 8, Day 53)	9	0	0.20	(-3.59, 3.99)	4.93	-5.82	-0.84	8.71	0.907	0.910
	Visit 8 (Week 12, Day 81)	9	0	1.38	(-1.00, 3.75)	3.09	-2.57	-0.03	6.86	0.218	0.496
	Visit 14 (Week 24, Day 165)	9	0	1.47	(-0.87, 3.82)	3.05	-1.93	0.67	7.68	0.186	0.250
	Visit 15 (Week 28, Day 190)	9	0	0.96	(-1.67, 3.59)	3.42	-1.93	-0.46	9.45	0.426	0.820
	Change Week 24 to Week 28	9	0	-0.52	(-1.83, 0.80)	1.71	-2.78	-1.29	1.77	0.391	0.301

Miss. = Missing (subject attends a visit but measure not recorded)
All units = x10^9/L; WSRT=Wilcoxon Sign-Rank Test
** Change from Baseline other than the Row 'Change Week 24 to Week 28'

Table 13.4.1.1 Summary of MRI Cross Sectional Muscle Area (mm2) - Central Reading

Antisense Therapeutics Limited
Protocol: 1102-DMD-CT02
Population: All Enrolled Subjects (excludes Screen Failures) (N=9)

		Actual Values							Change From Screening						
Muscle Group	Visit/Time	n	Miss.	Mean	Std.	Min.	Median	Max.	n	Miss.	Mean	Std.	Min.	Median	Max.
Volar Muscle	Visit 1 (Screening)	9	0	904.8	367.9	424	789.0	1548							
	Visit 8 (Week 12, Day 81)	9	0	940.6	404.0	419	754.0	1665	9	0	35.8	84.2	-50.0	7.0	225.0
	Visit 14 (Week 24, Day 165)	9	0	927.6	365.7	416	785.0	1508	9	0	22.8	70.2	-73.0	16.0	125.0
Dorsal Muscles	Visit 1 (Screening)	9	0	466.6	180.5	237	482.0	829							
	Visit 8 (Week 12, Day 81)	9	0	457.4	177.2	245	468.0	825	9	0	-9.1	19.6	-44.0	-7.0	21.0
	Visit 14 (Week 24, Day 165)	9	0	467.4	172.8	253	479.0	804	9	0	0.9	25.7	-34.0	-3.0	41.0
ECRLB-Br	Visit 1 (Screening)	9	0	99.3	65.8	14	95.0	207							
	Visit 8 (Week 12, Day 81)	9	0	97.0	70.7	8	94.0	213	9	0	-2.3	6.7	-16.0	-1.0	6.0
	Visit 14 (Week 24, Day 165)	9	0	98.0	70.1	21	91.0	227	9	0	-1.3	9.9	-15.0	-4.0	20.0
Total Area (mm2)	Visit 1 (Screening)	9	0	1471	574.0	756	1361	2550							
	Visit 8 (Week 12, Day 81)	9	0	1495	597.2	758	1432	2497	9	0	24.3	79.2	-74.0	2.0	185.0
	Visit 14 (Week 24, Day 165)	9	0	1493	547.5	760	1328	2459	9	0	22.3	76.9	-91.0	32.0	126.0

Miss. = Missing (measure not recorded for a subject)

Table 13.4.1.2 Summary of MRI Cross Sectional Muscle Area (mm2) - Distal Reading

Antisense Therapeutics Limited
Protocol: 1102-DMD-CT02
Population: All Enrolled Subjects (excludes Screen Failures) (N=9)

		Actual Values							Change From Screening						
Muscle Group	Visit/Time	n	Miss.	Mean	Std.	Min.	Median	Max.	n	Miss.	Mean	Std.	Min.	Median	Max.
Volar Muscle	Visit 1 (Screening)	9	0	422.0	137.0	250	378.0	686							
	Visit 8 (Week 12, Day 81)	8	1	426.4	121.6	286	402.5	651	8	1	-17.1	19.0	-36.0	-26.0	12.0
	Visit 14 (Week 24, Day 165)	7	2	416.3	129.8	322	365.0	696	7	2	18.0	64.7	-55.0	10.0	145.0
Dorsal Muscles	Visit 1 (Screening)	9	0	172.9	102.7	39	174.0	313							
	Visit 8 (Week 12, Day 81)	8	1	193.9	92.8	32	199.0	322	8	1	6.3	28.1	-24.0	2.5	69.0
	Visit 14 (Week 24, Day 165)	7	2	183.9	115.4	36	182.0	327	7	2	11.0	13.9	-3.0	8.0	37.0
ECRLB-Br	Visit 1 (Screening)	0	9	.	.	.	.	.							
	Visit 8 (Week 12, Day 81)	0	9	.	.	.	.	.	0	9	.	.	.	.	.
	Visit 14 (Week 24, Day 165)	0	9	.	.	.	.	.	0	9	.	.	.	.	.
Total Area (mm2)	Visit 1 (Screening)	9	0	594.9	218.6	305	591.0	999							
	Visit 8 (Week 12, Day 81)	8	1	620.3	196.6	318	593.0	973	8	1	-10.9	38.9	-53.0	-22.0	71.0
	Visit 14 (Week 24, Day 165)	7	2	600.1	210.5	401	607.0	1023	7	2	29.0	58.8	-37.0	16.0	151.0

Miss. = Missing (measure not recorded for a subject)

Table 13.4.1.3 Summary of MRI Cross Sectional Muscle Area (mm2) - Proximal Reading

Antisense Therapeutics Limited
Protocol: 1102-DMD-CT02
Population: All Enrolled Subjects (excludes Screen Failures) (N=9)

		Actual Values							Change From Screening						
Muscle Group	Visit/Time	n	Miss.	Mean	Std.	Min.	Median	Max.	n	Miss.	Mean	Std.	Min.	Median	Max.
Volar Muscle	Visit 1 (Screening)	9	0	909.0	249.2	469	923.0	1249							
	Visit 8 (Week 12, Day 81)	9	0	912.6	275.1	472	891.0	1327	9	0	3.6	78.6	-177	3.0	78.0
	Visit 14 (Week 24, Day 165)	9	0	888.1	340.1	453	777.0	1449	9	0	-20.9	188.7	-475	22.0	200.0
Dorsal Muscles	Visit 1 (Screening)	9	0	537.3	209.3	371	503.0	1031							
	Visit 8 (Week 12, Day 81)	9	0	517.0	190.4	371	473.0	926	9	0	-20.3	44.2	-105	-21.0	53.0
	Visit 14 (Week 24, Day 165)	9	0	538.2	201.5	341	436.0	868	9	0	0.9	116.5	-163	-10.0	265.0
ECRLB-Br	Visit 1 (Screening)	9	0	210.8	81.9	85	205.0	339							
	Visit 8 (Week 12, Day 81)	9	0	222.9	83.5	82	210.0	319	9	0	12.1	40.1	-23.0	0.0	111.0
	Visit 14 (Week 24, Day 165)	9	0	225.0	80.8	84	206.0	329	9	0	14.2	26.0	-10.0	1.0	74.0
Total Area (mm2)	Visit 1 (Screening)	9	0	1657	472.2	947	1631	2452							
	Visit 8 (Week 12, Day 81)	9	0	1652	495.5	956	1547	2342	9	0	-4.7	122.9	-213	9.0	216.0
	Visit 14 (Week 24, Day 165)	9	0	1651	505.1	971	1418	2310	9	0	-5.8	145.3	-213	24.0	207.0

Miss. = Missing (measure not recorded for a subject)

Table 13.5.1.1 Summary of MRI Fat Fraction (%) - Central Reading

Antisense Therapeutics Limited
Protocol: 1102-DMD-CT02
Population: All Enrolled Subjects (excludes Screen Failures) (N=9)

		Actual Values							Change From Screening						
Muscle Group	Visit/Time	n	Miss.	Mean	Std.	Min.	Median	Max.	n	Miss.	Mean	Std.	Min.	Median	Max.
Volar Muscle	Visit 1 (Screening)	9	0	34.5	23.1	9	23.3	65							
	Visit 8 (Week 12, Day 81)	9	0	33.5	26.4	7	20.1	73	9	0	-1.1	10.2	-26.3	0.5	8.5
	Visit 14 (Week 24, Day 165)	9	0	34.0	25.6	10	20.9	73	9	0	-0.6	9.4	-23.6	2.3	9.2
Dorsal Muscles	Visit 1 (Screening)	9	0	25.6	13.9	12	22.5	50							
	Visit 8 (Week 12, Day 81)	9	0	25.7	15.5	11	16.3	51	9	0	0.2	5.0	-8.3	0.2	9.4
	Visit 14 (Week 24, Day 165)	9	0	24.7	15.4	10	16.7	50	9	0	-0.9	3.3	-7.9	-0.6	4.8
ECRLB-Br	Visit 1 (Screening)	9	0	21.9	10.0	10	22.1	42							
	Visit 8 (Week 12, Day 81)	9	0	22.9	13.5	8	20.0	48	9	0	1.0	7.6	-12.8	0.4	10.4
	Visit 14 (Week 24, Day 165)	9	0	21.8	13.6	4	25.0	44	9	0	-0.1	8.2	-14.6	2.1	11.1
Average Fat Fraction (%)	Visit 1 (Screening)	9	0	27.3	14.6	12	21.3	49							
	Visit 8 (Week 12, Day 81)	9	0	27.4	17.8	9	17.3	57	9	0	0.0	7.0	-15.8	0.4	8.0
	Visit 14 (Week 24, Day 165)	9	0	26.8	17.6	8	19.2	51	9	0	-0.5	6.6	-15.3	1.4	8.3

Miss. = Missing (measure not recorded for a subject)

Table 13.5.1.2 Summary of MRI Fat Fraction (%) - Distal Reading

Antisense Therapeutics Limited
Protocol: 1102-DMD-CT02
Population: All Enrolled Subjects (excludes Screen Failures) (N=9)

		Actual Values							Change From Screening						
Muscle Group	Visit/Time	n	Miss.	Mean	Std.	Min.	Median	Max.	n	Miss.	Mean	Std.	Min.	Median	Max.
Volar Muscle	Visit 1 (Screening)	9	0	37.1	17.4	16	33.9	59							
	Visit 8 (Week 12, Day 81)	8	1	34.2	19.3	15	27.0	62	8	1	-3.3	12.2	-32.5	0.1	5.7
	Visit 14 (Week 24, Day 165)	7	2	35.6	21.4	12	32.8	62	7	2	-5.8	13.2	-26.0	1.1	5.6
Dorsal Muscles	Visit 1 (Screening)	9	0	34.0	18.8	14	27.4	69							
	Visit 8 (Week 12, Day 81)	8	1	28.7	15.0	13	26.0	53	8	1	-3.5	14.4	-37.3	-1.5	10.5
	Visit 14 (Week 24, Day 165)	7	2	30.1	16.0	13	24.8	52	7	2	-7.8	21.9	-44.2	2.9	14.0
ECRLB-Br	Visit 1 (Screening)	0	9	.	.	.	.	.							
	Visit 8 (Week 12, Day 81)	0	9	.	.	.	.	.	0	9	.	.	.	.	.
	Visit 14 (Week 24, Day 165)	0	9	.	.	.	.	.	0	9	.	.	.	.	.
Average Fat Fraction (%)	Visit 1 (Screening)	9	0	33.2	13.9	15	38.9	53							
	Visit 8 (Week 12, Day 81)	8	1	30.3	17.5	14	21.6	58	8	1	-2.0	9.3	-23.3	-0.9	7.1
	Visit 14 (Week 24, Day 165)	7	2	31.5	19.3	14	19.2	57	7	2	-5.1	14.6	-27.7	2.0	9.8

Miss. = Missing (measure not recorded for a subject)

Table 13.5.1.3 Summary of MRI Fat Fraction (%) - Proximal Reading

Antisense Therapeutics Limited
Protocol: 1102-DMD-CT02
Population: All Enrolled Subjects (excludes Screen Failures) (N=9)

		Actual Values							Change From Screening						
Muscle Group	Visit/Time	n	Miss.	Mean	Std.	Min.	Median	Max.	n	Miss.	Mean	Std.	Min.	Median	Max.
Volar Muscle	Visit 1 (Screening)	9	0	41.5	22.7	13	33.7	73							
	Visit 8 (Week 12, Day 81)	9	0	39.7	23.6	9	30.7	74	9	0	-1.8	10.2	-27.5	0.9	6.5
	Visit 14 (Week 24, Day 165)	9	0	37.3	21.8	17	29.1	71	9	0	-4.2	11.0	-28.5	-2.1	10.4
Dorsal Muscles	Visit 1 (Screening)	9	0	27.9	13.4	12	25.9	45							
	Visit 8 (Week 12, Day 81)	9	0	27.8	17.6	6	20.4	57	9	0	-0.1	9.8	-18.4	-0.3	14.3
	Visit 14 (Week 24, Day 165)	9	0	26.9	15.7	7	18.9	51	9	0	-0.9	9.2	-20.7	0.4	9.7
ECRLB-Br	Visit 1 (Screening)	9	0	29.2	20.3	11	21.0	58							
	Visit 8 (Week 12, Day 81)	9	0	26.4	21.0	5	15.0	58	9	0	-2.8	6.7	-16.4	-1.4	5.1
	Visit 14 (Week 24, Day 165)	9	0	27.9	22.0	7	17.0	65	9	0	-1.3	6.4	-14.2	-1.5	7.7
Average Fat Fraction (%)	Visit 1 (Screening)	9	0	32.9	17.7	15	23.9	57							
	Visit 8 (Week 12, Day 81)	9	0	31.3	20.5	6	21.2	63	9	0	-1.6	7.1	-17.2	0.1	5.7
	Visit 14 (Week 24, Day 165)	9	0	30.7	19.4	10	20.9	60	9	0	-2.1	7.1	-16.9	-0.4	9.3

Miss. = Missing (measure not recorded for a subject)

Table 13.6 Summary of MRI - Fat and Lean Muscle Area

Antisense Therapeutics Limited
Protocol: 1102-DMD-CT02
Population: All Enrolled Subjects (excludes Screen Failures) (N=9)

			Actual Values							Change From Screening						
Reading	Area (mm²)	Visit/Time	n	Miss.	Mean	Std.	Min.	Median	Max.	n	Miss.	Mean	Std.	Min.	Median	Max.
Central	Fat	Visit 1 (Screening)	9	0	451.9	344.1	96	385.1	1182							
		Visit 8 (Week 12, Day 81)	9	0	474.2	449.9	78	305.8	1491	9	0	22.3	145.4	-255	7.3	309.3
		Visit 14 (Week 24, Day 165)	9	0	460.3	414.7	112	234.3	1395	9	0	8.4	114.9	-229	18.0	213.4
Central	Lean	Visit 1 (Screening)	9	0	1019	474.0	371	1028	2039							
		Visit 8 (Week 12, Day 81)	9	0	1021	468.0	378	907.1	1983	9	0	2.0	96.2	-131	18.0	180.6
		Visit 14 (Week 24, Day 165)	9	0	1033	451.9	342	990.4	1930	9	0	13.9	112.5	-181	5.8	179.7
Distal	Fat	Visit 1 (Screening)	9	0	209.2	122.0	77	216.2	432							
		Visit 8 (Week 12, Day 81)	8	1	209.5	149.6	68	151.4	474	8	1	-11.9	52.4	-131	1.0	42.7
		Visit 14 (Week 24, Day 165)	7	2	222.3	180.3	55	128.6	525	7	2	-2.9	61.3	-87.6	0.6	93.6
Distal	Lean	Visit 1 (Screening)	9	0	385.7	164.7	145	405.4	621							
		Visit 8 (Week 12, Day 81)	8	1	410.8	138.9	233	442.4	585	8	1	1.0	54.4	-68.7	-11.5	87.9
		Visit 14 (Week 24, Day 165)	7	2	377.8	112.5	258	367.7	549	7	2	31.9	100.7	-69.6	-14.6	207.5

Miss. = Missing (measure not recorded for a subject)
Fat Area(mm²)=(Dorsal Area x Dorsal Fat Fraction)+(Volar Area x Volar Fat Fraction)+(ECRLB-Br Area x ECRLB-Br Fat Fraction)
Lean Muscle Area = the remaining area after subtracting the fat fraction from the total CSA, i.e. the non-fat muscle area.

Table 13.6 Summary of MRI - Fat and Lean Muscle Area

Antisense Therapeutics Limited
Protocol: 1102-DMD-CT02
Population: All Enrolled Subjects (excludes Screen Failures) (N=9)

			Actual Values							Change From Screening						
Reading	Area (mm²)	Visit/Time	n	Miss.	Mean	Std.	Min.	Median	Max.	n	Miss.	Mean	Std.	Min.	Median	Max.
Proximal	Fat Area	Visit 1 (Screening)	9	0	563.8	279.7	193	545.4	1050							
		Visit 8 (Week 12, Day 81)	9	0	559.7	371.1	90	553.2	1318	9	0	-4.1	166.8	-350	3.8	268.1
		Visit 14 (Week 24, Day 165)	9	0	545.0	370.9	165	493.7	1384	9	0	-18.8	182.2	-372	-10.9	333.8
Proximal	Lean	Visit 1 (Screening)	9	0	1093	466.1	402	1090	1846							
		Visit 8 (Week 12, Day 81)	9	0	1093	445.5	403	1135	1759	9	0	-0.6	144.4	-109	-34.8	369.7
		Visit 14 (Week 24, Day 165)	9	0	1106	453.9	472	1114	1816	9	0	13.0	163.2	-202	21.8	340.3

Miss. = Missing (measure not recorded for a subject)
Fat Area(mm²)=(Dorsal Area x Dorsal Fat Fraction)+(Volar Area x Volar Fat Fraction)+(ECRLB-Br Area x ECRLB-Br Fat Fraction)
Lean Muscle Area = the remaining area after subtracting the fat fraction from the total CSA, i.e. the non-fat muscle area.

Table 13.7.1 Summary of MRI - Lean Muscle Area (mm²)- By Muscle Group - Central Reading

Antisense Therapeutics Limited
Protocol: 1102-DMD-CT02
Population: All Enrolled Subjects (excludes Screen Failures) (N=9)

		Actual Values							Change From Screening						
Muscle Group	Visit/Time	n	Miss.	Mean	Std.	Min.	Median	Max.	n	Miss.	Mean	Std.	Min.	Median	Max.
Dorsal	Visit 1 (Screening)	9	0	349.1	146.5	119.4	358.6	642.5							
	Visit 8 (Week 12, Day 81)	9	0	338.5	143.6	135.0	338.1	630.3	9	0	-10.6	25.5	-52.2	-4.8	16.7
	Visit 14 (Week 24, Day 165)	9	0	352.9	145.7	126.5	340.4	627.9	9	0	3.9	26.3	-49.6	7.1	38.6
Volar	Visit 1 (Screening)	9	0	595.1	324.5	182.7	558.3	1263.2							
	Visit 8 (Week 12, Day 81)	9	0	614.9	330.4	178.5	602.4	1235.9	9	0	19.8	74.8	-69.4	21.6	179.5
	Visit 14 (Week 24, Day 165)	9	0	609.8	323.1	157.2	598.7	1176.2	9	0	14.6	91.6	-114.1	-16.3	166.1
ECRLB-Br	Visit 1 (Screening)	9	0	74.7	44.7	12.0	68.7	133.2							
	Visit 8 (Week 12, Day 81)	9	0	67.4	41.2	7.4	64.3	116.8	9	0	-7.2	6.3	-16.5	-4.6	2.0
	Visit 14 (Week 24, Day 165)	9	0	70.0	42.0	20.2	58.6	128.0	9	0	-4.6	8.8	-17.8	-7.5	8.2
Total Lean Muscle Area	Visit 1 (Screening)	9	0	1019	474.0	370.9	1028	2038.9							
	Visit 8 (Week 12, Day 81)	9	0	1021	468.0	377.8	907.1	1982.9	9	0	2.0	96.2	-131.4	18.0	180.6
	Visit 14 (Week 24, Day 165)	9	0	1033	451.9	342.4	990.4	1929.8	9	0	13.9	112.5	-181.4	5.8	179.7

Miss. = Missing (measure not recorded for a subject)
Lean Muscle Area = the remaining area after subtracting the fat fraction from the total CSA, i.e. the non-fat muscle area.

Table 13.7.2 Summary of MRI - Lean Muscle Area (mm²)- By Muscle Group - Distal Reading

Antisense Therapeutics Limited
Protocol: 1102-DMD-CT02
Population: All Enrolled Subjects (excludes Screen Failures) (N=9)

		Actual Values							Change From Screening						
Muscle Group	Visit/Time	n	Miss.	Mean	Std.	Min.	Median	Max.	n	Miss.	Mean	Std.	Min.	Median	Max.
Dorsal	Visit 1 (Screening)	9	0	121.4	80.5	12.1	90.2	227.2							
	Visit 8 (Week 12, Day 81)	8	1	136.9	68.6	21.9	139.3	232.1	8	1	3.8	26.8	-27.3	-0.1	58.8
	Visit 14 (Week 24, Day 165)	7	2	122.4	80.5	27.1	102.1	256.6	7	2	5.2	25.3	-35.6	11.9	40.1
Volar	Visit 1 (Screening)	9	0	264.3	103.8	132.7	295.2	424.9							
	Visit 8 (Week 12, Day 81)	8	1	273.9	89.6	155.8	297.0	403.3	8	1	-2.7	38.0	-41.4	-10.5	78.1
	Visit 14 (Week 24, Day 165)	7	2	255.4	70.3	158.4	265.7	349.6	7	2	26.7	87.5	-49.5	-2.8	184.3
ECRLB-Br	Visit 1 (Screening)	0	9												
	Visit 8 (Week 12, Day 81)	0	9						0	9					
	Visit 14 (Week 24, Day 165)	0	9						0	9					
Total Lean Muscle Area	Visit 1 (Screening)	9	0	385.7	164.7	144.8	405.4	621.4							
	Visit 8 (Week 12, Day 81)	8	1	410.8	138.9	232.6	442.4	585.2	8	1	1.0	54.4	-68.7	-11.5	87.9
	Visit 14 (Week 24, Day 165)	7	2	377.8	112.5	258.3	367.7	549.0	7	2	31.9	100.7	-69.6	-14.6	207.5

Miss. = Missing (measure not recorded for a subject)
Lean Muscle Area = the remaining area after subtracting the fat fraction from the total CSA, i.e. the non-fat muscle area.

Table 13.7.3 Summary of MRI - Lean Muscle Area (mm²)- By Muscle Group - Proximal Reading

Antisense Therapeutics Limited
Protocol: 1102-DMD-CT02
Population: All Enrolled Subjects (excludes Screen Failures) (N=9)

		Actual Values							Change From Screening						
Muscle Group	Visit/Time	n	Miss.	Mean	Std.	Min.	Median	Max.	n	Miss.	Mean	Std.	Min.	Median	Max.
Dorsal	Visit 1 (Screening)	9	0	388.4	165.6	215.8	359.8	764.0							
	Visit 8 (Week 12, Day 81)	9	0	371.8	160.6	168.8	372.1	744.5	9	0	-16.5	43.4	-71.1	-19.5	68.7
	Visit 14 (Week 24, Day 165)	9	0	391.6	164.1	197.5	357.1	703.9	9	0	3.2	64.9	-60.0	-13.0	153.1
Volar	Visit 1 (Screening)	9	0	547.4	261.6	149.6	552.7	833.9							
	Visit 8 (Week 12, Day 81)	9	0	554.2	249.2	144.4	652.6	883.8	9	0	6.8	116.2	-121.7	-15.3	285.3
	Visit 14 (Week 24, Day 165)	9	0	548.3	268.7	219.0	575.6	981.0	9	0	0.9	178.3	-341.7	22.9	287.2
ECRLB-Br	Visit 1 (Screening)	9	0	157.6	88.4	36.2	154.5	303.4							
	Visit 8 (Week 12, Day 81)	9	0	166.8	76.2	36.5	166.2	274.6	9	0	9.2	29.7	-28.8	2.0	77.8
	Visit 14 (Week 24, Day 165)	9	0	166.4	84.3	37.5	150.1	291.8	9	0	8.9	16.5	-13.5	9.7	39.5
Total Lean Muscle Area	Visit 1 (Screening)	9	0	1093	466.1	401.6	1090	1845.8							
	Visit 8 (Week 12, Day 81)	9	0	1093	445.5	402.8	1135	1759.5	9	0	-0.6	144.4	-109.4	-34.8	369.7
	Visit 14 (Week 24, Day 165)	9	0	1106	453.9	472.3	1114	1816.3	9	0	13.0	163.2	-202.1	21.8	340.3

Miss. = Missing (measure not recorded for a subject)
Lean Muscle Area = the remaining area after subtracting the fat fraction from the total CSA, i.e. the non-fat muscle area.
