# Supplementary material for: A phase 2 open-label study of the safety and efficacy of weekly dosing of ATL1102 in patients with non-ambulatory Duchenne muscular dystrophy and pharmacology in mdx mice
Source: PLoS One. 2024 Jan 25;19(1):e0294847. doi: 10.1371/journal.pone.0294847 (PMC10810432; doi:10.1371/journal.pone.0294847)
Supplement: S2 File — (PDF) [file pone.0294847.s010.pdf]

Study Title:	A Phase 2 open label study to determine the safety, efficacy and pharmacokinetic profile of weekly dosing of ATL1102 in patients with non-ambulatory Duchenne Muscular Dystrophy.
Protocol Number:	1102-DMD-CT02
Test Drug:	ATL1102 (CD49d antisense oligonucleotide)
Indication:	Duchenne Muscular Dystrophy
Study Sponsor:	Antisense Therapeutics Limited<br>6 Wallace Avenue<br>Toorak Victoria 3142<br>Australia<br>Telephone +61 3 9827 8999
Version:	FINAL, 6.0
Date:	20 Sep 2019

The investigator is obliged to provide Antisense Therapeutics Limited (ATL) with complete test results and all data derived from this study.

PROTOCOL SIGNATURES

Sponsor's Approval

The signature below constitutes Antisense Therapeutics Limited approval of this protocol.

________________________________ ____________________
Date

Investigator's Statement

The undersigned Investigator agrees:

1. To conduct the study in accordance with the study protocol, the International Council on Harmonisation Good Clinical Practice and any national and local laws and regulations.
2. That alteration of the procedures described in the study protocol, other than to protect participant safety, rights, or welfare, is not allowed without prior written approval from Antisense Therapeutics Limited.
3. The study-specific data of the participants will be kept in the participants' files and documented in the case report form in a complete and accurate manner. All requested study-related records will be made available for direct access to ATL representatives for monitoring or auditing the study.
4. To allow authorised qualified delegates of Antisense Therapeutics Limited to perform regular visits to monitor the study data.
5. To dispose of used and unused investigational product and materials as instructed by Antisense Therapeutics Limited.
6. To ensure that all persons at their site assisting with the clinical study are adequately informed and trained about the study protocol, the investigational product and their study-related duties and functions.

Investigator signature	Date
Name	Site number

1 PROTOCOL SYNOPSIS

Study Title:	A Phase 2 open label study to determine the safety, efficacy and pharmacokinetic profile of weekly dosing of ATL1102 in patients with non-ambulatory Duchenne Muscular Dystrophy.
Study Number:	1102-DMD-CT02
Study Phase:	2
Indication:	Duchenne Muscular Dystrophy (DMD)
Study Objectives:	Primary objective:<br>• To assess the safety and tolerability of 25 mg of ATL1102 administered once weekly in patients with DMD.<br><br>Secondary objectives:<br>• To investigate the lymphocyte-modulatory potential of ATL1102 in patients with DMD.<br>• To evaluate the pharmacokinetic (PK) profile of ATL1102 in patients with DMD.<br>• To evaluate the effects of ATL1102 on functional capacity in patients with DMD.<br>• To evaluate the effects of ATL1102 on respiratory function in patients with DMD.<br>• To evaluate the effects of ATL1102 on quality-of-life in patients with DMD.<br><br>Exploratory objectives:<br>• To further evaluate the pharmacodynamic (PD) effects of ATL1102 in patients with DMD.
Study Design	This is a single-centre, open-labelled study to assess the safety, efficacy, PD and PK of a once weekly dose of 25mg ATL1102 for 24 weeks in non-ambulatory patients with DMD.<br>One dosing regimen will be assessed in 9 participants as follows:<br>• 25 mg, once per week for 24 weeks, subcutaneously.<br>Safety data will be reviewed by an independent Data Safety Monitoring Board (DSMB) throughout the study.<br>Study Participants will be administered ATL1102 either at the study site or in the home environment:<br>• Study site dosing: Weeks 1, 3, 5, 7, 10, 14, 16, 18, 20 and 22<br>• Home environment dosing: Weeks 2, 4, 6, 8, 9, 11, 12, 13, 15, 17, 19, 21, 23 and 24.

	Study site assessments will include evaluations of safety, PD and PK and occur at Screening, Baseline (Week 1, Day 1), Weeks 3, 5, 7, 8, 10, 12, 14, 16, 18, 20, 22, 24, 28 and 32, with the final study visit being at Week 32.
Number of Participants:	9 participants with DMD will be enrolled.
Number of Sites:	One study site in Australia.
Duration of Study per participant:	Up to 36 weeks:<br>• Up to 28-day screening period<br>• 24-week dosing period<br>• 8-week follow-up period
Inclusion Criteria:	Participants who:<br>1. Are adolescent males, aged 10 to 18 years inclusive, at the time of providing informed consent.<br>2. Have been diagnosed with Duchenne Muscular Dystrophy and have been non-ambulatory for at least 3 months. Non-ambulatory for this study is defined as having consistently required a wheelchair to mobilise more than a few metres for at least 3 months.<br>The diagnosis of DMD is to be confirmed by at least one of the following:<br>a. Dystrophin immunofluorescence and/or immunoblot showing near complete dystrophin protein deficiency, and clinical picture consistent with typical DMD; or<br>b. Gene deletion test positive (missing one or more exons) of the dystrophin gene, where the reading frame can be predicted as 'out-of-frame', and clinical picture consistent with typical DMD; or<br>c. Complete dystrophin gene sequencing showing an alteration (point mutation, duplication, or other mutation resulting in a stop codon) that can be definitely associated with DMD, with a typical clinical picture of DMD; or<br>d. Positive family history of DMD confirmed by one of the criteria listed above in a sibling or maternal uncle, and clinical picture typical of DMD.<br>3. Have a body weight of more than 25 kg and less than or equal to 65 kg.<br>4. If currently receiving glucocorticoid therapy, have been on a stable dose of glucocorticoid therapy for at least 3 months prior to Day 1.<br>5. Are currently on stable doses of cardiac therapy (including angiotensin converting enzyme inhibitors, aldosterone receptor antagonists and/or beta blockers) for at least 3 months prior to Day 1.<br>6. Have a parent/guardian who is capable of understanding the purposes and risks of the study and able to provide written informed consent. If the participant is of sufficient maturity and has the ability to understand the nature and consequence of the study, involvement in study consent discussions are required.

7. Are able, and have a parent/guardian who are, willing and able to comply with scheduled visits, study drug administration plan, and study procedures.

Exclusion Criteria: Participants who:

1. Have been diagnosed with Duchenne Muscular Dystrophy and are still ambulatory. Ambulatory for this study is being able to complete at least 75 meters during the 6-minute walk test in the 4 weeks prior to Day 1.
2. Have the following abnormal haematology values during the Screening period or on Day 1, prior to first dose:
   a. Lymphocytes <1.2 x 10^9/L
   b. Neutrophils <1.8 x 10^9/L
   c. Platelets <150 x 10^9/L
3. Have a history of clinically significant bleeding or coagulation abnormalities.
4. Have hepatic dysfunction indicated by an abnormal total bilirubin and gamma glutamyl transferase (GGT) results at Screening.
5. Have renal impairment indicated by serum creatinine ≥ 1.5 mg/dL (132 umol/l) at Screening.
6. Have uncontrolled clinical symptoms and signs of congestive heart failure consistent with Stage C or Stage D criteria according to the American College of Cardiology/American Heart Association guidelines for cardiac dysfunction within 3 months of Day 1.
7. Have an inability to complete the cardiac, pulmonary or strength range of motion and mobility assessments at Screening.
8. Have taken nutritional, herbal, or antioxidant supplements that have a known demonstrated activity for maintaining or improving skeletal muscle strength or functional mobility within 4 weeks of Day 1. NOTE: daily multivitamin, Vitamin D or calcium supplements are permitted.
9. Are currently receiving antiplatelet or anticoagulant therapy, or have taken medication with an antiplatelet or anticoagulant effect within 4 weeks prior Day 1 (e.g., aspirin).
10. Have received any investigational product in the 2 months prior to Screening (4 months if the previous drug was a new chemical entity), whichever is longer.
11. Have severe behavioural disorder or inadequate cognitive development that would make them unable to comply with the study assessments, which, in the opinion of the investigator, makes the participant unsuitable for participation in the study.

Investigational Product, Dose, and Mode of Administration:	The Investigational Product (IP) is ATL1102.<br><br>The ATL1102 drug product formulation is a 150mg/mL sterile, aqueous solution containing the ATL1102 drug substance in Water for Injection with a small amount of either HCl or NaOH to adjust the pH of the solution to 7.4.<br><br>Dose of ATL1102 will be 25 mg, once per week for 24 weeks, subcutaneously.<br><br>The dose is to be administered as a single injection in different anatomical sites (rotating every week), with the preferred administration area being the healthy and intact skin of the abdomen:<br><br>• Injection volume 0.17ml (one injection site required).
Criteria for Evaluation:	<u>Primary Objective Endpoint:</u><br><br>Safety and tolerability will be assessed by:<br>• The frequency and intensity of adverse events (AEs)<br>• The frequency and intensity of redness and swelling at the injection site<br>• Number and percentage values of the following laboratory tests indicative of vascular and renal inflammation:<br>• C-reactive protein (CRP)<br>• Creatinine and Creatinine Clearance<br>• Number and percentage of platelet values<br>• Other Clinical laboratory tests and assessments<br>• Haematology<br>• Coagulation<br>• Biochemistry<br>• Urinalysis<br>• Vital signs<br>• Electrocardiogram (ECG)<br><br><u>Secondary Objective Endpoints:</u><br><br>*<u>Primary efficacy endpoints</u>*<br><br>Lymphocyte-modulation potential will be determined by assessing the following haematological parameters by cell surface flow cytometry:<br>• Number and percentages of lymphocytes<br>• Number and percentages of CD4+ and CD8+ T cells<br>• Number of CD4+ CD49dhi and CD8+ CD49dhi T cells<br><br><u>Pharmacokinetic endpoints</u> include concentrations of ATL1102 in plasma (apparent values where applicable):<br>• Area under the plasma concentration time curve (AUC)<br>• AUC from time point zero (baseline) extrapolated to infinity ($AUC_{0\text{-inf}}$)<br>• AUC from time point zero (baseline) to the time point of the last quantifiable concentration ($AUC_{0\text{-last}}$)

- Maximum and minimum concentration (C_{max} and C_{min}) and C_{trough} in plasma
- Time to reach maximum concentration in plasma (T_{max})
- Apparent terminal plasma half-life ($t_{½}$)

Secondary efficacy endpoints

Functional capacity will be assessed by the number and percentage (as appropriate) of change in:

- Upper limb muscle inflammation score (muscle oedema), as determined by Short Tau Inversion Recovery (STIR) on quantitative MRI
- Muscle structure score (comprised of muscle atrophy and fat infiltration) as determined by quantitative MRI utilising the 3-point Dixon sequences of upper limb muscle structure
- MyoSet score as determined by the Myo-Pinch, Myo-Grip, and MoviPlate scores
- Performance of Upper Limb (PUL), version 2
- Egen Klassifikation (EK) Scale, version 2, to evaluate functional ability in non-ambulant participants

Respiratory function will be assessed by:

- Change in maximum inspiratory pressure (MIP) and expiratory pressures (MEP)
- Change in peak expiratory flow (PEF) and cough peak flow (CPF)
- Change in forced vital capacity (FVC) and forced expiratory volume in 1 second (FEV1)
- Change in sniff nasal inspiratory pressure (SNIP)

Quality of life will be assessed by percentage of change in neuromuscular modules of the Pediatric quality of life instrument (PedsQL) score.

Exploratory PD endpoints

Blood and serum samples will be collected to:

- Investigate inflammatory and muscle injury marker changes:
  CRP and other serum markers of inflammation
  Creatine kinase (CK), Aspartate aminotransferase (AST), and Lactate dehydrogenase (LDH) for muscle injury
- Explore the effect of ATL1102 on key haematology cells and purified mononuclear cell ribonucleic acid (RNA).

Statistical Methods and Analyses:

Sample Size Justification:

A key primary efficacy variable is lymphocyte count. A clinically important, and statistically significant reduction in lymphocyte count from baseline to end of treatment will provide some evidence of efficacy of ATL1102 for DMD patients. Based on results from the clinical study 1102-CT02 (assessing ATL1102 in patients with remitting-relapsing multiple sclerosis), the ATL1102 group baseline mean lymphocyte count was 1.89 (x10^9/L) with a standard deviation of 0.428 (x10^9/L). For change from baseline to end of treatment the standard deviation of lymphocyte count was 0.477 (10^9/L). A clinically important reduction in lymphocyte count from baseline to end of treatment was judged to be 25%, which equates to a reduction of 0.47 (=0.75x1.89) (x10^9/L) in mean lymphocyte count. For the sample size calculation, the level of significance was set to 0.05 with a 2-sided paired t-test, mean difference of 0.47 (x10^9/L) from baseline to end of treatment, and standard deviation of 0.428 (x10^9/L). With these settings a sample size of 9 patients is required to achieve a power of 80%.

Nine evaluable participants are considered sufficient to investigate the safety, tolerability and PK and PD profile of ATL1102 in a rare target patient population.

Quantitative safety, efficacy, PK and PD data will be summarised by descriptive statistics (arithmetic mean, standard deviation, standard error of the mean, median, minimum, and maximum) overall and over time. Summaries will also be presented for the change from baseline, when appropriate. Qualitative variables will be summarised by frequency, percentage of patients, and Clopper-Pearson 95% CI overall, and over time.

For key efficacy variables, such as lymphocyte count, the paired t-test will be used to test the change from baseline to end of treatment. There will be no adjustment for multiple comparisons.

All participants who have received at least one dose of ATL1102 will be evaluated for safety. All AEs will be coded using the Medical Dictionary for Regulatory Activities. Incidence of AEs by the severity, relationship to treatment, and outcome will be provided. Laboratory parameters, vital signs and other safety parameters will be listed by participant and presented using descriptive summary statistics (as appropriate).

There will be no imputation for missing observations. The results will be reported as observed.

Committees: An independent Data Safety Monitoring Board (DSMB) will be established prior to study start, with an appropriate charter to review safety data at regular intervals throughout the study.

1. STUDY SCHEDULE OF ASSESSMENTS

Study Stage	Screening	Baseline	Active Treatment												Follow Up	
**Assessment Number**	**1**	**2**	**3**	**4**	**5**	**6**	**7**	**8**	**9**	**10**	**11**	**12**	**13**	**14**	**15**	**16**
**Study Week**		**Week1 [w]**	**Week 3**	**Week 5**	**Week 7**	**Week 8**	**Week 10**	**Week 12**	**Week 14**	**Week 16**	**Week 18**	**Week 20**	**Week 22**	**Week 24**	**Week 28^**	**Week 32**
**Study Day**	**Day -28 to Day -1**	**Day 1**	**Day 15**	**Day 29**	**Day 43**	**Day 53**	**Day 64**	**Day 81**	**Day 92**	**Day 106**	**Day 120**	**Day 134**	**Day 148**	**Day 165**	**Day 190**	**Day 218**
Study Visit Windows		*0*	*± 1 day*	*± 1 day*	*± 1 day*	*± 1 day*	*± 1 day*	*± 1 day*	*± 1 day*	*± 1 day*	*± 1 day*	*± 1 day*	*± 1 day*	*± 1 day*	*± 3 days*	*± 3 days*
Number of ATL1102 Doses		*1*	*3*	*5*	*7*	*8*	*10*	*12*	*14*	*16*	*18*	*20*	*22*	*24*		
Informed Consent	X															
Review of Eligibility Criteria	X	X														
Demographics & Medical History	X															
Physical Examination[a]	X	X		X		X		X		X				X	X	X
Height	X															
Body Weight	X	X		X				X						X	X	
Vital signs[b]	X	X[c]	X	X		X		X		X				X	X	X
12-lead ECG[d]	X	X								X		X			X	
MRI[e]	X							X						X		
Haematology[f]	X	X[g]	X	X	X	X	X	X	X	X[g]	X	X	X	X	X	X
Biochemistry[h] and haptoglobin*	X	X	X*	X	X*	X	X*	X	X*	X	X*	X	X*	X	X	X
Coagulation parameters[i] & fibrinogen**	X	X	X**	X	X**	X	X**	X	X**	X	X**	X	X**	X	X	
Urinalysis[j]	X	X	X	X	X	X	X	X	X	X	X	X	X	X	X	
Lymphocyte Cells Samples[k]		X		X		X		X						X	X	
Complement C3, C4, Bb samples[l]		X		X		X		X		X		X		X	X	
Immunogenicity samples[m] & MCP-1***		X	X***	X	X***	X	X***	X	X***	X	X***	X	X***	X	X	
Functional Capacity Assessments[n]		X		X		X		X						X		

**Study Stage**	**Screening**	**Baseline**	**Active Treatment**												**Follow Up**	
**Assessment Number**	**1**	**2**	**3**	**4**	**5**	**6**	**7**	**8**	**9**	**10**	**11**	**12**	**13**	**14**	**15**	**16**
**Study Week**		**Week1 [w]**	**Week 3**	**Week 5**	**Week 7**	**Week 8**	**Week 10**	**Week 12**	**Week 14**	**Week 16**	**Week 18**	**Week 20**	**Week 22**	**Week 24**	**Week 28^**	**Week 32**
**Study Day**	**Day -28 to Day -1**	**Day 1**	**Day 15**	**Day 29**	**Day 43**	**Day 53**	**Day 64**	**Day 81**	**Day 92**	**Day 106**	**Day 120**	**Day 134**	**Day 148**	**Day 165**	**Day 190**	**Day 218**
Study Visit Windows		*0*	*± 1 day*	*± 1 day*	*± 1 day*	*± 1 day*	*± 1 day*	*± 1 day*	*± 1 day*	*± 1 day*	*± 1 day*	*± 1 day*	*± 1 day*	*± 1 day*	*± 3 days*	*± 3 days*
Number of ATL1102 Doses		*1*	*3*	*5*	*7*	*8*	*10*	*12*	*14*	*16*	*18*	*20*	*22*	*24*		
Quality of Life Assessment[o]		X						X						X		
Respiratory Function assessments[p]		X		X		X		X						X	X	
Steroids/NSAID Phone reminder the day prior to each scheduled visit[q]	X	X	X	X	X	X	X	X	X	X	X	X	X	X	X	X
IP administration, Week 1 to 24[r]		X	X	X	X		X		X	X	X	X	X			
Local injection site tolerability assessment[s]		X	X	X	X	X	X	X	X	X	X	X	X	X	X	X
Provision of Diary Card to Participants[t]		X	X	X	X	X	X	X	X	X	X	X	X			
Pharmacokinetic samples[u]		X		X		X		X		X				X	X	X
Exploratory PD samples[v]		X		X		X		X		X		X		X	X	
Adverse event assessments		X	X	X	X	X	X	X	X	X	X	X	X	X	X	X
Concomitant medication	X	X	X	X	X	X	X	X	X	X	X	X	X	X	X	X

^ If a decision is made to discontinue treatment or withdrew from the study for/by a participant for any reason, the Week 28 assessments should be conducted at that time. Additionally, if possible, a follow-up MRI should be performed if the participant withdraws prior to either Week 12 or between Week 13 and Week 24. If a participant withdraws consent and does not wish to attend for any further visits, they should be encouraged to attend the Week 28 visit as a final visit as a minimum.

a. Physical examination includes, at a minimum, HEENT, heart, respiratory, abdomen, extremities, neurological assessments.

b. Vital signs include blood pressure, pulse and tympanic temperature and will be assessed within 30 mins prior to study drug administration on dosing days.

c. Vital signs on Day 1 will be assessed within 30 mins prior to study drug administration and 1, 2 and 6 hours after administration.

d. Resting 12-lead ECG are to be done in triplicate at: Screening, Week 1 (Baseline), 16 and 28. They will be taken pre- dose (within 1 hour prior to dose administration) and post-dose (at least 1 hour post ATL1102 dose administration) on Day 1 (Week 1) and Day 106 (Week 16). The participant should have rested for ≥5 minutes with readings to be taken within 2-5 mins of each other.

e. MRI will assess Functional Muscle Structure and Oedema - upper limb function. Inflammation will be assessed by Short tau Inversion Recovery (STIR) method assessing oedema, and muscle atrophy and fat measured by the 3-point Dixon T1 weighted method. MRI at Screening can be done at any time during the 28 day Screening period, and has a ± 1 week visit window for Week 12 and Week 24 assessment.
f. Haematology will be done pre-dose and include haemoglobin, haematocrit, reticulocytes, red blood cell count, mean corpuscular cell volume, mean corpuscular cell haemoglobin, platelet count, total and differential white blood cell count (neutrophil, lymphocytes, monocytes, eosinophils, basophils)
g. For participants taking a corticosteroid, a haematology sample will also be collected post ATL1102 dose and post administration of their corticosteroid medication at Day 1 (Week 1) and Day 106 (Week 16) at 4 hours post corticosteroid dose.
h. Biochemistry (includes Liver Function Tests) will be done pre-dose and include sodium, potassium, bicarbonate, phosphate, chloride, calcium, urea, creatinine, creatinine clearance (using Modified Schwartz formula), haptoglobin, total bilirubin, direct bilirubin, alkaline phosphatase, aspartate transaminase, alanine transaminase, GGT, total protein, glucose, albumin, albumin: globulin ratio (A/G ratio), lactate dehydrogenase, creatinine kinase (CK), C-reactive protein (CRP).
*On weeks 7, 10, 14, 18 and 22, days 43, 64, 92, 120, and 148 respectively, pre-dose, only the tests for haptoglobin will be conducted.
i. Coagulation is to be done pre-dose and include prothrombin time, activated partial thromboplastin time, international normalised ratio (INR), and fibrinogen.
**On Weeks 7, 10, 14, 18 and 22, days 43, 64, 92, 120 and 148 respectively, pre-dose, only the tests for fibrinogen will be conducted.
j. Urinalysis is to be done on a sample of the first morning urine, pre-dose WEEKLY throughout the dosing period and include protein, blood, glucose, blood leucocyte esterase, pH, specific gravity, ketones, urobilinogen - by dipstick analysis. Microscopy analysis to be performed if any abnormalities. Where the weekly test does not coincide with a clinic visit, the test is to be performed by the participant or parent/guardian at their home pre-dose. When performed at home, if the dipstick protein rises to 2 or above the investigator is to be immediately notified. On clinic visit days a sample of the first morning urine should be collected either at the clinic visit or at the participant's home, as required. If collected at the participant's home, the sample should be stored cold in the refrigerator (approximately 2-8°C) and transported in the urine specimen cold pack until arrival at the clinic visit. The sample should be tested within 3 hours of collection.
k. Lymphocyte cells sample will be collected pre-dose, and comprise blood sample for cell surface flow cytometry (efficacy endpoint). It is preferred that samples be collected in the morning.
l. Complement C3, C4 and Bb samples will be collected pre-dose of ATL1102. Only C3 analysed. The C4 and Bb samples will be processed as serum and plasma samples respective, and stored frozen. If CRP increases and/or albumin and A/G ratio decrease, or Immunoglobulin G (IgG) has been found to increase, the rest of assessments will be performed based on the safety monitoring guidelines.
m. Immunogenicity, samples will be collected pre-dose at weeks 1, 5, 8, 12, 16, 20, 24 and 28, and include IgG, ANA, ANCA, ADA, MCP-1 and CIC. ***On weeks 3, 7, 10, 14, 18 and 22, only monocyte chemotactic protein-1 (MCP-1) samples will be collected pre-dose. Serum/plasma samples will be prepared from both immunogenicity and MCP-1 samples and based on safety monitoring guidelines if CRP has been found to increase and/or albumin, A/G ratio decreases, immunogenicity samples will be analysed for IgG and if found to increase, the MCP-1 samples will be analysed for MCP-1. If the IgG and MCP-1 has been found to increase the immunogenicity samples will be analysed for one of more of: anti-neutrophil cytoplasmic antibodies, anti-nuclear antibodies, anti-drug antibodies, and circulating immune complexes based on safety monitoring guidelines.
n. Functional capacity assessments are to be done pre-dose and include Myoset scores (Myopinch, MyoGrip, MoviPlate (clinician), PUL version 2 (clinician), Egan Klassifikation Scale version 2 (participant/carer). These are in addition to the MRI functional assessments.
o. Quality of Life assessment will be done pre-dose and be the neuromuscular module of Pediatric Quality of Life Inventory tool.
p. Respiratory function will be assessed pre-dose and include sniff nasal inspiratory pressure, peak expiratory flow, cough peak flow, forced vital capacity, forced expiratory volume1, maximum inspiratory pressure, maximum expiratory pressure.

q. If the participant is taking a corticosteroid as part of their concomitant medication regimen, the site will contact the participant/participants family the day before each scheduled visit to provide reminder of the requirement to NOT take steroid medication within 24 hours of the scheduled visit. Steroid dose is to be taken after any pre-dose bloods samples have been collected. No dose of NSAID is permitted within 24 hours of a scheduled visit also.

r. IP to be administered subcutaneously once per week, on the same day each week for 24 weeks (with a ± 1 day window permitted for each dose). For IP administration at Week 8, Week 12, and Week 24, the dose is to be administered 3 days PRIOR to the scheduled site visits for that week (e.g. dose on Day 50 for site visit to occur three days later on Day 53). Laboratory investigations require bi-weekly monitoring of haematology (including platelets and red blood cells), haptoglobin and fibrinogen. **No dose of study drug should be given before the investigator determines whether or not the bi-weekly platelet count is in a safe range.**

s. Local tolerability will be assessed by reviewing any redness, swelling and/or pain at the injection site. On Day 1 (Week 1) and Day 106 (Week 16), assessments will occur at 1, 2 and 6 hours after the ATL1102 injection. The size of the redness and/or swelling will be captured, and de-identified photographs of injection site reactions may be taken as assessed by the Investigator or delegate.

t. The participants will complete a Diary Card to capture any site reactions (local tolerability) for up to 48 hours after each dose and if a reaction is present at the end of 48 hours, to continue to capture until resolution or end of their study participation. As applicable, an adequate number of Diary Cards are to be given to the participant to cover for any subsequent doses to be administered at home.

u. PK samples taken pre-dose and 1, 2, 3, 4 and 6 hours post-dose on Day 1 (Week 1) and Day 106 (Week 16), and pre-dose only on Day 29 (Week 5). Cmin levels will also be taken at Day 53 (Week 8), Day 81 (Week 12), Day 165 (Week 24), and trough levels on Day 190 (Week 28), and Day 218 (Week 32).

v. The following exploratory PD samples will be collected pre-dose for the potential assessment of (i) blood for cell surface flow cytometry analysis of cells, (ii) blood for ficol gradient recovery of mononuclear cells for RNA preparation for transcriptome analysis and (iii) blood for isolation of exosomes for RNA and protein analysis (iv) serum for cytokine testing by ELISA and/or serum potentially for proteomic arrays for pathway analysis.

w. The Week 1, Day 1 Baseline visit is an extended visit to accommodate the post dose PK sampling to 6 hours post dose administration and therefore, may be performed over 2 consecutive days where the pre-dose assessments for Functional Capacity and the Quality-of-Life assessment can be performed a day prior to Day 1, where all other pre-dose assessments, dose administration and post dose assessments are to be performed.

TABLE OF CONTENTS

1 PROTOCOL SYNOPSIS....3

1. STUDY SCHEDULE OF ASSESSMENTS....9

2 BACKGROUND AND RATIONALE....20

2.1 ANTISENSE THEORY AND ATL1102....20

2.2 DUCHENNE MUSCULAR DYSTROPHY....21

2.3 NONCLINICAL STUDIES WITH ATL1102....22

2.3.1 Nonclinical Safety Studies....22

2.3.2 Animal Pharmacokinetics....23

2.4 CLINICAL STUDIES WITH ATL1102....23

2.4.1 Clinical Study 1102-CT01....24

2.4.2 Clinical Study 1102 SCM-CT01....24

2.4.3 Clinical Study 1102-CT02....24

2.4.4 Adverse Events with ATL1102....25

2.5 STUDY AND DOSE RATIONALE....26

2.5.1 Study Rationale....26

2.5.2 Dose Rationale....28

3 STUDY OBJECTIVES AND ENDPOINTS....30

4 STUDY PLAN....32

4.1 OVERALL STUDY DESIGN....32

4.2 NUMBER OF STUDY SITES....33

4.3 NUMBER OF PARTICIPANTS....33

4.4 STUDY DURATION....33

5 STUDY POPULATION....33

5.1 STUDY SELECTION RECORD....33

5.2 INCLUSION CRITERIA....34

5.3 EXCLUSION CRITERIA....34

5.4 PARTICIPANT COMPLETION AND WITHDRAWAL....35

5.4.1 Completion of Participant from Study....35

5.4.2 Withdrawal of Participants from Study....35

5.4.3 Procedure for Handling Withdrawals....36

5.4.4 Premature Termination of Study ... 36
5.4.5 Replacement of Participants ... 36

6 SAFETY MANAGEMENT PROCEDURES ... 36
6.1.1 Data Safety Monitoring Board ... 36
6.1.2 Stopping Rules / Discontinuation Criteria ... 37
6.1.3 Stopping Rules for Renal Function Test Results ... 38
6.1.4 Stopping Rule for Platelet Count Results ... 39
6.1.5 Stopping rule for C-reactive protein ... 39
6.1.6 Stopping Rule for Albumin and A/G ratio Complement C3 ... 39
6.1.7 Contraception requirements ... 40
6.1.8 Concomitant Medications and Treatments ... 41

7 STUDY TREATMENT ... 41
7.1.1 Description of the Investigational Product ... 41
7.1.2 Manufacture, Packaging and Labelling ... 41
7.1.3 Investigational Product Handling and Storage ... 42
7.1.4 Dosage and Administration ... 42
7.1.5 Compliance of Investigational Product ... 42
7.1.6 Accountability of Investigational Product ... 43
7.1.7 Dose Frequency Adjustment ... 43

8 STUDY PROCEDURES ... 44
8.1 PARTICIPANT INFORMATION AND CONSENT ... 44
8.1.1 Method of assigning treatment ... 44
8.2 STUDY PROCEDURES ... 44
8.2.1 Screening Visit (Day -28 to Day -1) – Assessment 1 ... 45
8.2.2 Active Treatment Period – Assessment Numbers 2 to 14 ... 46
8.2.3 Follow Up Period – Assessments 15 and 16 ... 55

9 STUDY MEASUREMENTS ... 56
9.1 CLINICAL SAFETY PROCEDURES ... 56
9.2 EFFICACY PROCEDURES ... 58
9.3 PHARMACOKINETICS AND EXPLORATORY PD SAMPLES ... 59
9.4 BLOOD VOLUMES FOR CLINICAL LABORATORY, PK AND PD ANALYSIS ... 60

10 ADVERSE EVENTS ... 62

10.1 ADVERSE EVENT DEFINITIONS ... 62

10.2 SERIOUS ADVERSE EVENTS ... 63

10.3 RECORDING OF ADVERSE EVENTS ... 64

10.4 REPORTING SERIOUS ADVERSE EVENTS ... 64

10.5 FOLLOW-UP OF ADVERSE EVENTS AND SERIOUS ADVERSE EVENTS ... 64

10.6 PREGNANCY REPORTING ... 64

11 DATA MANAGEMENT ... 65

11.1 DATA MANAGEMENT AND QUALITY CONTROL ... 65

12 STATISTICAL ANALYSIS ... 65

12.1 ANALYSIS SETS ... 65

12.2 DEMOGRAPHIC CHARACTERISTICS AND BASELINE DATA ... 66

12.3 EFFICACY DATA ... 66

12.4 SAFETY DATA ... 67

12.4.1 Extent of exposure ... 67

12.4.2 Adverse events ... 67

12.4.3 Clinical laboratory evaluations ... 68

12.4.4 Other safety measures ... 68

12.5 PHARMACOKINETIC DATA ... 68

12.6 VITAL SIGNS ... 68

12.7 SAMPLE SIZE CONSIDERATIONS ... 68

13 STUDY MANAGEMENT ... 69

13.1 CONTACTS ... 69

13.2 MONITORING ... 69

13.3 AUDITS AND INSPECTIONS ... 69

13.4 TRAINING OF STAFF ... 69

13.5 CHANGES TO THE PROTOCOL ... 70

13.6 STUDY AGREEMENTS ... 70

13.7 ETHICS REVIEW ... 70

13.8 ETHICAL CONDUCT OF THE STUDY ... 70

13.9 INSURANCE AND LIABILITY ... 70

13.10 PARTICIPANT INFORMATION AND INFORMED CONSENT ... 71

13.11 DATA PROTECTION 71
13.12 STUDY REGISTRATION 71
13.13 ARCHIVING 71
13.14 PUBLICATION POLICY 71

14 REFERENCES 73

LIST OF ABBREVIATIONS

Abbreviation	Definition
6MWT	6-minute walk test
AE	Adverse Event
ADA	Anti-drug antibodies
ADME	Absorption, distribution, metabolism and excretion
A/G	Albumin/globulin
ALP	Alkaline phosphatase
ALT	Alanine aminotransferase
ANA	Anti-nuclear antibodies
ANCA	anti-neutrophil cytoplasmic antibodies
APTT	Activated Partial Prothrombin Time
ASO	Antisense Oligonucleotide
AST	Aspartate aminotransferase
ATL	Antisense Therapeutics Limited
AUC	Area under (concentration-time) curve to last time-point
AUC_{inf}	Area under (concentration-time) curve to infinity
CIC	circulating immune complexes
CK	Creatinine kinase
C_{max}	Maximum observed plasma concentration
C_{min}	Minimum observed plasma concentration
CRA	Clinical Research Associate
CRF	Case Report Form
CPF	Cough peak flow
CRP	C-reactive protein
DNA	Deoxyribonucleic acid
DMD	Duchenne Muscular Dystrophy
DSMB	Data Safety Monitoring Board
ECG	Electrocardiogram
eGFR	Estimated Glomerular Filtration Rate
EK	Egen Klassifikation
FEV1	Forced expiratory volume in 1 second
FVC	Forced vital capacity
GCP	Good Clinical Practice
G-CSF	granulocyte colony stimulating factor
GGT	Gamma glutamyl transferase
IB	Investigator’s Brochure
ICH	International Council for Harmonisation
ID	Identification

IEC	Independent Ethics Committee
IgM	Immunoglobulin M
INR	International Normalised Ratio
IP	Investigational product
ITT	Intent-to-Treat
IUD	Intra-uterine device
IV	Intravenous
LDH	Lactate dehydrogenase
LOAEL	Low observed adverse effect level
MCP1	Monocyte chemotactic protein-1
MedDRA	Medical Dictionary for Regulatory Activities
MEP	Maximum expiratory pressure
MIP	Maximum inspiratory pressure
MRI	Magnetic Resonance Imaging
mRNA	Messenger Ribonucleic Acid
MS	Multiple Sclerosis
NOAEL	No observed adverse effect level
PD	Pharmacodynamic
PedsQL	Pediatric quality-of-life instrument
PEF	Peak expiratory flow
PICF	Participant Information and Consent Form
PK	Pharmacokinetic
PP	Per Protocol
PT	Preferred Term
PUL	Performance of the Upper Limb
RBC	Red blood cell
RNA	Ribonucleic acid
RRMS	Relapsing remitting multiple sclerosis
SAE	Serious Adverse Event
SAP	Statistical Analysis Plan
SC	subcutaneous
SNIP	Sniff nasal inspiratory pressure
SOC	System Organ Class
STIR	Short Tau Inversion Recovery
$T_{1/2}$	Terminal elimination half-life
TEAE	Treatment emergent adverse event
T_{max}	Time to maximal concentration
ULN	Upper Limit of Normal
VLA-4	Very Late Antigen 4

WBC	White blood cell

2 BACKGROUND AND RATIONALE

Investigators should be familiar with the current ATL1102 Investigator's Brochure (IB).

2.1 Antisense Theory and ATL1102

Antisense oligonucleotides (ASOs) are short, single stranded, chemically synthetic compounds, generally 15 to 25 bases in length, designed usually to inhibit expression of a target disease causing protein by sequence-specific binding to the protein's messenger ribonucleic acid (mRNA). In another antisense approach to therapeutics, oligonucleotides are designed to skip mutated exons in RNA to increase the level of in-frame RNA and to increase expression of a key protein like dystrophin in a disease like DMD. ASO are not biological in origin.

The interaction of an ASO with the target RNA is through the inherent high selectivity of Watson-Crick base-pairing (i.e. adenine bases always pair with thymines or uracils; guanines always pair with cytosines). Hybridisation of the ATL1102 ASO with its target RNA attracts RNase H which results in degradation of the target RNA-strand of the RNA:deoxyribonucleic acid (DNA) (oligonucleotide) duplex preventing the translation of the RNA to protein.

The use of oligonucleotides to block protein synthesis from mRNA has the potential advantage of much greater specificity compared to conventional small molecule drugs. The majority of drugs currently in use modulate the activity of specific proteins by either binding directly to the protein of interest or by binding to other proteins, such as cell surface receptors, which then modulate the target protein. Because many proteins are members of closely-related families which perform the same or a similar function, or are members of activity classes (e.g., proteinases, receptor tyrosine kinases, etc.), small molecule drugs often bind to, and affect the activity of, more than one target protein. In contrast, the hybridisation of antisense oligonucleotides to mRNA is based upon the high specificity of nucleotide sequences. As a result, it is possible to design oligonucleotides capable of inhibiting the expression of a single member of a closely related set of proteins, e.g., one of several enzyme isoforms. Therefore, antisense oligonucleotides that are developed as therapeutic agents have the potential to display fewer non-specific toxic effects than other, less selective, agents.

ATL1102 is an immunomodulatory antisense drug to human CD49d RNA, the alpha 4-integrin chain of Very Late Antigen 4 (VLA-4) (alpha4, beta1) expressed on white blood cells. It is a second generation ASO drug to the 3'-untranslated region of the human CD49d mRNA, and *in vitro* down regulates VLA-4 expression on T cells, and interferes with T-cell adhesion. This has been shown to alter disease phenotype in multiple sclerosis (MS).

ATL1102, in a Phase 2a trial in 74 adult patients with relapsing remitting multiple sclerosis (RRMS), dosed three times 200mg in the first week and twice weekly 200mg for 7 weeks significantly reduced (90%) inflammatory brain lesions at 12 weeks compared to placebo (Limmroth *et al*, 2010). ATL1102 was also shown to reduce:

- Blood lymphocytes by approximately 25% after 4 weeks of dosing compared to baseline;
- CD4+ and CD8+ T lymphocytes by approximately 25% at Week 8 compared to baseline;
- B cells (by approximately 50%) and neutrophils (by approximately 40%) at Week 8 compared to baseline; and
- CD49d expression on approximately 10% of CD4+T cells at Week 12 compared to baseline.

ATL1102 was well tolerated with mild to moderate injection site reactions, mild increases in liver enzymes, and mild to moderate reductions in platelets that returned to normal after dosing was completed (Limmroth *et al*, 2014).

ATL1102 is being developed for immune-inflammatory disorders, with this study being the first in participants with Duchenne Muscular Dystrophy (DMD).

2.2 Duchenne Muscular Dystrophy

Duchenne Muscular Dystrophy is an X-linked disease that affects 1 in 3600 to 6000 live male births (Bushby *et al*, 2010). It occurs as a result of mutations in the dystrophin gene, mainly deletions, which affect the production of protein dystrophin, causing either an absence of the protein or a defect in the protein. This results in progressive muscle degeneration, affecting the ability to ambulate unaided (Bushby *et al*, 2010). The disease predominantly affects males, with approximately 10% of females who are carriers showing some milder disease manifestations such as cognitive or cardiac dysfunction.

Diagnosis normally occurs at 5 years of age when their physical abilities differ from those of their peers, leading to use of classic Gower's manoeuvre (meaning that boys need to support themselves with hands on their thighs as they get up from the floor). Ongoing deterioration in muscle strength that affects both lower limbs, leads to impaired mobility of the upper limbs, and to further loss of function and self-care. Most children require a wheelchair by their early teenage years, with respiratory, cardiac and cognitive dysfunction also emerging. With no intervention, the mean age at death is approximately 19 years (Bushby *et al*, 2010; Seferian *et al,* 2015).

The management of DMD is currently the use of "corticosteroid, with respiratory, cardiac, orthopaedic, and rehabilitative interventions improving function, quality-of-life, health, and longevity" which has resulted in an extension of life expectancy into the fourth decade (Bushby *et al*, 2010).

Diagnosis of DMD involves assessing creatine kinase (CK) levels, which will be elevated in patients with DMD, a muscle biopsy for the absence of dystrophin protein, and testing for a mutation of the *DMD* gene. The disease progresses through various stages:

Stage 1: Pre-symptomatic	Able to be diagnosed at this stage if CK raised or positive family history.<br>May show developmental delay but no gait disturbance.
Stage 2: Early Ambulatory	Gowers' sign/manoeuvre, waddling, waddling gait, might be toe walking, can climb stairs.
Stage 3: Late Ambulatory	Increasingly laboured gait, losing ability to climb stairs and rise from floor.
Stage 4: Early non-ambulatory:	May be able to self-propel for some time, able to maintain posture, may develop scoliosis.
Stage 5: Late non-ambulatory:	Upper limb function and postural maintenance is increasingly limited.<br>Management at this stage is focused on supporting neuromuscular, orthopaedic, pulmonary, cardiac, gastrointestinal/speech/swallowing/nutrition functions, and psychosocial management.

Evaluating patients with DMD normally involves a 6-minute walk test (6MWT), which measures the

distance that a patient can walk on a flat, hard surface in 6 minutes. With the loss of ambulation, other measurements are employed to assess the status of the disease. These include clinician assessments of Performance of the Upper Limb (PUL) test, which was specifically designed for DMD. The PUL assessment is a validated tool that includes 22 items that assess across three levels with an entry level item to provide a starting functional level. Four items assess high-level performance (shoulder), nine items at mid-level and eight items assessing the distal level (Mayhew *et al*, 2013). Self-reported measures such as Egen Klassifikation (EK) Scale (version 2) are also used, with the EK Scale assessment being completed by the patient or their carer, to determine the functional ability of non-ambulatory patients with DMD. It consists of a composite scale of 10 items that are clinically relevant in DMD (Seferian *et al*, 2015).

New therapies are being developed for treating DMD, and as such, tools for evaluating effects in ambulatory and non-ambulatory patients have been designed. Assessing muscle fat transformation via magnetic resonance imaging (MRI) in patients with DMD has been shown effective in monitoring disease progression in the upper limbs of patients with DMD, with validation on the association of progressive fat transformation of muscles with loss of muscle force and function (Ricotti *et al*, 2016, Diaz-Manera *et al*, 2015). Merlini and Sabatelli (2015) have suggested a surrogate biochemical outcome is also possible to support the assessment of new treatments by demonstrating *de novo* dystrophin production and its expected beneficial effect on functional recovery of muscle fibre. In non-ambulatory patients with DMD, being able to evaluate the upper limbs is key. The Myoset assessment, is a validated tool that involves assessment of the grip (MyoGrip), pinch strength (MyoPinch) and hand function (MoviPlate) and has shown correlation between distal strength and the clinical variables such as forced vital capacity, age, duration since loss of ambulation and Brooke score, with the sensitive dynamometers of the MyoSet able to capture change in all ages of non-ambulant patients with DMD (Seferian *et al*, 2015; Servais *et al*, 2013).

2.3 Nonclinical Studies with ATL1102

The nonclinical development of ATL1102 to support both the MS and DMD indications included a battery of safety pharmacology studies, pharmacokinetic (PK) and toxicology studies, and supportive absorption, distribution, metabolism and excretion (ADME) studies.

2.3.1 Nonclinical Safety Studies

The safety and toxicity profile of ATL1102 has been evaluated in studies in mice and cynomolgus monkeys up to 27 weeks in duration via the subcutaneous (SC) route. Toxicity findings were consistent with typical ASO class-related effects in both species, including transient and/or partially reversible reductions in platelet counts; complement activation; renal tubular vacuolation associated with renal tissue uptake of ATL1102; increased serum alanine aminotransferase (ALT)/ aspartate aminotransferase (AST) and reversible histologic liver changes of lymphohistiocyte infiltrate; and histiocytic cell infiltrates in organs and in lymph nodes, reflective of immune stimulation primarily at the higher doses.

In monkeys, at doses of 3, 10 and 30 mg/kg/week, 4 of 16 high dose animals were euthanised moribund after approximately 6 months of dosing, and an unexpected moribund low dose animal (1 of 12) euthanised at the interim 13-week time-point. Class-related toxicities as noted above were associated with histopathology in the moribund animals; of note, there were heightened clinical and anatomic pathologies reflective of marked immune-stimulation, including vascular and perivascular inflammation at times in multiple organs, particularly at injection sites, in all ATL1102-treated groups. The absence of any similar changes in the previous 4-week monkey study, or in mice receiving ATL1102 and its mouse pharmacologic surrogate (TV-1104) for up to 6 months, or in clinical studies, prompted a repeat 6-month study in cynomolgus monkeys at doses of 1.5 and 3 mg/kg. In contrast to the original study, 3 mg/kg/week

was well tolerated for 13 and 26 weeks in all animals (8 of 8) in the repeat study, with no clinical signs of distress or adversity. As with the original study, histologic evidence of vascular inflammation was noted at 3 mg/kg, although of lower incidence and severity (1 of 8 animals, single occurrence, mild severity). The two chronic studies suggest that individual animal sensitivity to overall tolerability and vascular inflammation would appear to be immunologically-based, with no known apparent translation to human risk or safety. The repeat study established a no-observed-adverse-effect-level (NOAEL) of 1.5 mg/kg (no vasculitis or other adverse changes) and identified potential premonitory biomarkers that may be useful in the clinic that have been incorporated into this study.

ATL1102 was determined to be non-genotoxic in two mammalian *in vitro* assays and an *in vivo* study in mice, and had no effect on fertility in mice, and was non-teratogenic in mice and rabbits.

Further details on all observations with ATL1102 can be found in the Investigator Brochure (IB) for ATL1102.

2.3.2 **Animal Pharmacokinetics**

ADME/PK studies in animals supported that distribution to tissues is responsible for plasma clearance; with whole body clearance the result of metabolism and excretion of oligonucleotide fragments of ATL1102. Urinary excretion of radiolabelled ATL1102 was the primary route of elimination of radioactivity in rats.

Based on plasma area under the curve (AUC) and tissue concentrations, there was essentially 100% bioavailability from SC injections. Peak plasma concentrations occurred between 1 and 4 hours after injection, indicating gradual absorption from the site of injection. The apparent plasma half-life following SC injection was 4.8 to 5.7 hours due to ongoing absorption; the elimination half-life may be much longer due to slow tissue release, breakdown and clearance.

2.4 Clinical Studies with ATL1102

ATL1102 has been studied in three clinical studies to date, with two early phase studies in healthy volunteers and one study in patients with relapsing-remitting multiple sclerosis (RRMS). A total of 79 study participants have received at least one dose of ATL1102 thus far.

A total of 43 healthy volunteers were dosed in the two Phase 1 studies. Thirty-six healthy volunteers received ATL1102 in a first-in-human, two-part, placebo-controlled study to evaluate the safety, tolerability, and PKs of single and multiple SC doses of ATL1102 (ranging from 0.1 to 6 mg/kg) (Study 1102-CT01). The second Phase 1 study was a randomised, open-label study to assess the safety, tolerability, PK and pharmacodynamics (PD) of SC doses of ATL1102 alone and in combination with granulocyte-colony stimulating factor (G-CSF) in 7 healthy volunteers at a dose of 400mg/day (Study 1102-SCM-CT01).

One Phase 2 study has been completed to date (Study 1102-CT02). The study was a double-blind, placebo-controlled, multi-centre, randomised study to prove the therapeutic concept and determine the PK profile of ATL1102 by SC injections in patients with RRMS. It evaluated the effect of ATL1102 on inflammatory brain lesions as well as the safety and tolerability during 8 weeks of treatment. Seventy-seven patients with RRMS were enrolled, of which 36 received ATL1102 200mg SC three times a week in the first week and then 200mg SC twice weekly for Week 2 to 7. The 41 participants who received placebo followed the same dosing regimen.

2.4.1 Clinical Study 1102-CT01

The safety, tolerability and PKs of ATL1102 was first evaluated in a double-blind, placebo-controlled Phase 1 study of increasing single doses and multiple doses in healthy volunteers. A total of 54 healthy volunteers enrolled with 36 receiving ATL1102 and 18 receiving placebo in a 2:1 randomisation.

Overall there were 95 treatment emergent adverse events (TEAE) reported, with 46 TEAE reported by 23 of the male participants and 49 TEAE reported by 12 female participants. The most frequently reported TEAE were general disorders and administration site conditions, dizziness, headache, paraesthesia and somnolence. In the 6.0mg/kg dose group, one of the three participants received 1 dose only, and one received two doses due to injection site reactions. Most of the events were considered related to study medication (82%) and the majority of the events were assessed as mild (88%) by the Investigator with no event being deemed severe in intensity. There were no serious adverse events (SAEs) and no deaths in the study.

There were no clinically significant safety laboratory evaluations, vital signs physical examination or 12-lead electrocardiogram (ECG) measurements. Overall, ATL1102 was considered safe, with doses of up to 4mg/kg in healthy volunteers well tolerated, and based on the 6mg/kg dose group experiencing injection site reactions, an indication of less tolerance at these higher doses.

Generally, the PKs of ATL1102 were generally linear over the range of doses studied, with several parameters appearing to increase greater than proportional to dose. For higher doses, the elimination half-life was long (> 2 weeks in most participants), which is consistent with non-clinical data available. As prolonged tissue exposure of the oligonucleotide would be the goal in antisense therapy and, also, in order to reduce any potential for haemodynamic changes, the SC route has been shown as the preferred option of drug delivery.

2.4.2 Clinical Study 1102 SCM-CT01

The second Phase 1 study was a randomised, open-label study to assess the safety, tolerability, PK and PD of SC doses of ATL1102 alone and in combination with G-CSF (Neupogen®). A total of 10 healthy volunteers enrolled, with 7 receiving ATL1102.

The study focused primarily on safety data to support ongoing ATL1102 development. Both ATL1102 and Neupogen® when administered alone were well tolerated. When ATL1102 and Neupogen® (at 400mg/day and 10µg/kg/day doses respectively, daily for five days) were administered in combination, only moderate tolerability occurred. Overall there were 47 TEAEs, with 28 of these assessed as mild, and 18 events assessed as moderate. One of the TEAE was of severe intensity (drug hypersensitivity) which was also deemed to be a SAE.

2.4.3 Clinical Study 1102-CT02

The first Phase 2 study was a double-blind, placebo-controlled, multi-centre, randomised study to determine the safety and PK profile of ATL1102 as well as its potential for efficacy in patients with RRMS.

The most common TEAEs in the ATL1102 group were injection site erythema (25.0% participants), increased ALT (19.4%), MS relapse (16.7%), increased AST (11.1%), headache (11.1%), decreased platelet count (11.1%), and thrombocytopenia (11.1%). MS relapse was the most frequent TEAE in the placebo group (19.5% participants). Injection site erythema, thrombocytopenia and increased ALT were more frequent in the ATL1102 group than in the placebo group (difference ≥5%). All serious TEAEs were MS relapses except for one case of thrombocytopenia in the ATL1102 group.

The most frequent and clinically relevant change in laboratory safety variables was a decrease in platelet count observed in participants receiving ATL1102, with these decreases leading to the premature discontinuation of the study medication in two participants with moderate platelet reductions. Better local tolerability was reported in the placebo group than the ATL1102 group.

The PK profile determined for ATL1102 showed no increasing peak (C_{max}) or total (AUC) plasma exposure levels but increasing trough (pre-dose) levels suggested accumulation in tissues with multiple-dose administration. ATL1102 appears to have a long terminal elimination half-life of approximately 3 weeks.

The primary endpoint for this study was met, with ATL1102 significantly reducing the cumulative number of new active inflammatory brain lesions compared to placebo, and also reducing the circulating CD4 T-cells and CD8 T-cell by approximately 25% and reducing the proportion of VLA-4+ CD4+T-cells and CD8+ T cells by approximately 10%. ATL1102 significantly reduced the primary endpoint namely, the cumulative number of new-active inflammatory GdT1 lesions at Weeks, 4, 8 and 12 by 54% (Limmroth *et al*, 2014), which is comparable to natalizumab, the most effective antibody to VLA-4 therapy in MS in a similar duration 12 week MS study (Tubridy *et al*, 1999). The cumulative T1 lesion volume was also lower in the ATL1102 group than the placebo group: 358 (1028.4) mm^3 vs. 589 (1107.6) mm^3. The difference did not reach statistical significance (p=0.0534). No differences were found for clinical outcomes (number of MS relapses, Expanded Disability Status Scale score).

ATL1102 treatment was generally well tolerated (Limmroth *et al,* 2014) with mild-to-moderate injection site erythema (25%), mild liver enzyme increases (ALT increased 19.4% and AST increased 11.1%), headache (11.1%), and mild to moderate platelet count decreased with thrombocytopenia (22.2%). The thrombocytopenia was not associated with any bleeding events or clinical sequalae with platelets returning to within the normal range after cessation of dosing (Limmroth *et al,* 2014).

More comprehensive details of each of the clinical studies is provided in the IB.

2.4.4 Adverse Events with ATL1102

The most frequently reported TEAEs identified in Phase 1 studies were general disorders and administration site conditions, dizziness, headache, paraesthesia and somnolence, most of which were considered related to study medication. The majority of the events were assessed as mild with no event being deemed severe.

In the first Phase 2 study in patients with MS, the most common TEAEs in the ATL1102 group were injection site erythema, increased ALT and AST, MS relapse, headache, decreased platelet count, and thrombocytopenia. All serious TEAEs were MS relapses except for one case of thrombocytopenia in the ATL1102 group.

Table 1 below summarises TEAEs reported in the Study of ATL1102 in patients with RRMS (Limmroth *et al*, 2014).

Table 1: Treatment emergent adverse events (TEAEs) reported in ≥10% of participants in either treatment group

Type of TEAE (MEDRA preferred term)	Number (%) of Participants			
	Placebo (N=41)		ATL1102 (N=36)	
Injection site erythema	0	-	9	(25.0)
Thrombocytopenia	0	-	8^	(22.2)
Alanine aminotransferase increased	3	(7.3)	7	(19.4)
MS relapse	8	(19.5)	6	(16.7)
Aspartate aminotransferase increased	3	(7.3)	4	(11.1)
Headache	3	(7.3)	4	(11.1)

^ Represents platelet count abnormalities reported as adverse events. There was a drop in platelets by at least one third of starting levels in 4 participants and to below the lower limit of normal (LLN) in 4 participants. Platelet decreases led to premature discontinuation of the study medication in 2 participants with platelet counts below the LLN. Platelet counts recovered after discontinuation of study medication without any additional intervention.

2.5 Study and Dose Rationale

2.5.1 Study Rationale

Duchenne Muscular Dystrophy is a genetic muscular disease caused by loss of dystrophin, with progressive muscle wasting and associated muscle injury leading to inflammation and fibrosis, which further exacerbates the disease. DMD therapies aim to increase dystrophin levels and reduce inflammation using corticosteroids such as prednisolone, but corticosteroids although delaying loss of ambulation by up to 3 years, from 10 to 13 years of age (Koeks *et al*, 2017), have insufficient effectiveness, and long term use results in serious side effects. Improved anti-inflammatory therapies are needed to safely reduce immune-mediated pathology to ameliorate DMD severity and delay disease progression.

Patients with DMD demonstrate approximately 25% greater number of circulating CD4+ and CD8+T cells, expressing high levels of CD49d (hi-CD49d+) as compared to healthy children (Pinto-Mariz *et al,* 2015). In children with DMD who are non-ambulant or walk less than 1m/sec, there is approximately a 40% greater percentage of circulating CD4+ and CD8+T cells expressing high levels of CD49d (hi CD49d+) as compared to ambulant children walking >1m/sec (Pinto-Mariz *et al*, 2015). Pinto-Mariz *et al* (2015) have reported that ambulant children with a greater percentage of circulating hi-CD49d+ T cells have more severe and more rapid progression of disease than children with a fewer percentage of circulating hi-CD49d+ T cells. This study included ambulant patients treated with steroids, suggesting that the use of steroids did not significantly reduce the hi-CD49d+ T cells (at least at the time measured).

As summarised in a recent publication by Dr Janet Woodcock, Director of the FDA Center for Drug Evaluation and Research, children with DMD have dystrophin deficient muscles that are susceptible to contraction-induced injury triggering macrophages of the innate immune system, which leads to chronic inflammation involving macrophages and neutrophils (Rosenberg et al, 2015). These subsequently activate the adaptive immune system CD4+ and CD8+ T cells, which perpetuate immune mediated damage of muscle (Rosenberg *et al*, 2015). Independent studies have identified patients with CD4+ and CD8+ T cells also express high levels of CD49d (Pinto-Mariz *et al,* 2015), including boys on corticosteroids, or those that have lost ambulation by 10 years of age, and have high levels of the CD49d ligand osteopontin has also been found in patient muscle fibres (Zanotti *et al*, 2011). These patients also have

more rapidly progressive disease, suggesting that CD49d and its ligand osteopontin are more than disease severity markers in DMD, and are potential targets for the improved treatment of DMD.

Pinto Mariz *et al* (2015), also noted the following in patients with DMD with hiCD49d+ T cells

- 100% of patients who had greater than one third highCD49dT+CD4+ and greater than half highCD49dT+CD8+ T cells lost ambulation by the age of 10 compared to the average age of 13 years for loss of ambulation.
- Patients with DMD who were unable to walk by 10 years of age had 14-33% higher levels of hiCD49d+, CD4+, and CD8+ T cell than boys who lose the ability to ambulate after 10 years of age.
- Ambulant patients treated with steroids were included in the studies, suggesting steroids do not reduce the CD49d high expressing T cells sufficiently to delay loss of ambulation.

These findings support the further exploration of ATL1102 and the potential benefits of targeting CD49d+ T cells in boys with DMD.

CD49d plays an important role in leukocyte adhesion, transmigration, survival and activation of immune cells and interactions with extracellular matrix components osteopontin and fibronectin in muscle. Osteopontin, the ligand of VLA-4, with a role in both inflammation and fibrosis (Zanotti *et al*, 2011), is up-regulated in the muscle fibres of patients with DMD. CD49d+ T cell binding to fibronectin may drive muscle damage and fibrosis (Pinto-Mariz *et al*, 2010). Antibodies to CD49d in *ex-vivo* studies block transendothelial fibronectin driven migration of T cells and block T cell adhesion to myotubes in patients with DMD (Pinto-Mariz *et al,* 2015). CD49d+ T cells and in particular hi CD49d+ T cells, which are effector and memory cells (Iannetta *et al*, 2016), may thus drive the immune mediated muscle damage and fibrosis in patients with DMD.

Based on ATL1102's mechanism of action to reduce expression of CD49d+ on immune cells, T-cell adhesion *in vitro* and distribute to tissues including lymphoid organs and reduce circulating CD4+ T-cells and CD8+ T-cells and thereby the inflammation in the brains of MS patients (Section 2.1 and 2.4.3; Limmroth *et al*, 2014), ATL1102 represents a novel therapeutic strategy for reducing disease severity and slowing DMD disease progression by decreasing the immune cell-mediated damage of muscle in patients with DMD.

This study will assess key efficacy measurements of lymphocyte counts and by MRI, assessing functional muscle structure of the upper limbs by measuring fat infiltration and atrophy with 3-point Dixon sequence and oedema/inflammation (via the short tau inversion recovery (STIR)) method.

Further efficacy assessments include the MyoPinch, the most sensitive parameter to assess patient benefit, as part of the functional capacity assessments, supported by the MyoGrip and MoviPlate.

1. The laboratory parameter C-reactive protein (CRP) will be assessed as an exploratory marker for inflammation, with CK, lactate dehydrogenase (LDH), and AST exploratory markers for assessing for muscle injury. Standard safety laboratory measures will be assessed throughout the study with albumin, albumin/globulin (A/G) ratio, and Complement C3 part of the safety assessment for vasculitis. Platelet counts will be assessed along with haematology, biochemistry, key liver enzymes and urinalysis. Further safety measures will include injection site reactions and respiratory function tests.

2.5.2 Dose Rationale

The proposed dosing for this study in patients with DMD with ATL1102 is a maximum dose of 25mg once weekly for 24 weeks. Based on the minimum weight of above 25 kg and maximum weight of below 65 kg, this equates to a 0.39 mg/kg/week to below 1 mg/kg/week dosing regimen, which is supported by the safety and efficacy outcomes of the prior clinical studies, the ATL1102 animal toxicology data and the safety margins calculated from the preclinical toxicology studies in both mice and monkeys.

Support from Prior Clinical Studies of ATL1102:

In the MS patient Phase 2a study (1102-CT02), patients with RRMS were treated with ATL1102 200 mg three times in the first week then twice weekly for the next 7 weeks. In an 80 kg patient with MS, this would equate to a total of 7.5 mg/kg in the first week and a total 5 mg/kg/week for the following 7 weeks. This dosing schedule was supported by the outcomes from a Phase 1 study of ATL1102 in normal volunteers (1102-CT01). Based on the clinical experiences with other antisense drugs of a similar class, the ATL1102 dose tested was a high dose in the MS study, and lower doses such as those to be tested in the present study could be active.

As noted in the Phase 2 study summary (see Section 2.4.3) the reduction in the circulating CD4 T-cells and CD8 T-cell by approximately 25% and reduction in the proportion of VLA-4+ CD4+T-cells and VLA-4+ CD8+ T cells by approximately 10% is important when considering ATL1102 as a potential treatment for the inflammation and immune mediated damage associated with these cells in DMD (Pinto Mariz, et al 2015).

As noted above, the dose employed in the MS study was 200 mg three times in the first week and twice weekly for the next 7 weeks. By way of comparison, the 25 mg dose is 1/8th the individual dose and at lower frequency per week. The total exposure in the MS study is 3400 mg compared to 600 mg in the proposed study. Thus, the proposed dosing strategy for the DMD study is within the clinical safety experience for ATL1102. The proposed DMD study also measures all the potential safety risks observed in the ATL1102 clinical studies.

Support from Nonclinical Studies of ATL112:

The proposed DMD study design includes important measures to mitigate perceived safety risks to patients by employing appropriate clinical monitoring and related stopping rules including those related to nonclinical studies. Vascular and renal inflammation was reported in the six-month monkey toxicology studies at ATL1102 doses of 3 mg/kg/week. It was not observed in the six month monkey studies at doses of 1.5 mg/kg/week nor in the other preclinical studies (the one-month monkey toxicology study and the one and six-month mouse toxicology studies). There was no reported evidence of vasculitis in the 36 participants treated with ATL1102 for two months in the Phase II MS study (Limmroth *et al, 2014;* see Appendix 6 of the Investigator's Brochure (IB): "*Sturgess Report*"). The proposed DMD study will monitor for this adverse event (AE) and includes stopping rule criteria for any adverse inflammatory events that may be observed. Additionally, most participants in this study will be receiving corticosteroid therapy at a dose that would be considered protective of vasculitis risk as corticosteroid therapy is a first-line treatment of choice for severe vascular inflammation (Appendix 6 of IB: Sturgess Report).

The NOAELs from the chronic monkey studies provide plasma exposure safety margins of between 2 to 6.4-fold based on Cmax and 2.1 to 7.8-fold based on AUC and are supportive of dosing at the 25 mg dose level in patients with DMD in the weight range. The low observed adverse effect levels (LOAELs) exposures from chronic monkey studies are greater than the exposures at the NOAELs and provide plasma exposure safety margins of 6.3-fold for Cmax and 5 .1-fold for AUC in the lightest patients and in excess of10-fold in heavier patients and are supportive of dosing at the 25 mg dose level in patients with DMD and allow

for safe dose in boys of all weights, with monitoring of relevant clinical pathology parameters based on the NOAELs and LOAELs. With regard to the relevance of toxicities noted in long-term studies with ATL1102, particularly in monkeys against the backdrop of the disease state, there would appear to be minimal risk to human safety given 1) the proposed aetiology of antisense oligonucleotide complement activation and C3 reduction and sequelae not considered relevant for humans, 2) the low doses tested 3) the monitoring and stopping rules, and 4) the concurrent treatment of most patients with DMD with corticosteroids attenuating the potential for drug-related inflammatory AEs.

ATL1102 Activity in DMD trial:

In the Phase 2a MS study ATL1102 significantly reduced the cumulative number of new active inflammatory brain lesions, and also reduced circulating lymphocytes by 25% at both Week 4 (3 days after the 8th dose) and Week 8 (3 days after the 16th dose), with CD4 T-cells and CD8 T-cell reduced by approximately 25% at week 8. The total exposure in the MS study at 4 weeks was 1600 mg (20 mg/kg in an 80 kg patient) compared to 600 mg in the proposed DMD study (9.2 - 24 mg/kg in a 25-65 kg patient or median 16.6 mg/kg). Lighter body weight (25kg) patients achieve a 20 mg/kg exposure by 20 weeks, and 35 kg patients achieve up to 17mg/kg by week 24. Lymphocyte numbers were not assessed earlier than 4 weeks of dosing compared to Baseline in the MS study. If the 25% lymphocyte reduction had also occurred earlier than 4 weeks, at 2 weeks (3 days after the 4th dose), the MS patients would have received a cumulative dose of approximately 800 mg (10mg/kg in an 80 kg patient). The heaviest 65kg DMD patients achieve a 9.2 mg/kg exposure by week 24.

When contemplating the potential clinical activity of ATL1102 in this DMD trial dosing for 24 weeks it is also useful to look at the pharmacometric modelling for the Phase IIb MS study of the same duration (Guzy and Bauer, 2012). Based on the data from the Phase 2a 8-week MS study) modelling indicates that less frequent ATL1102 dosing of 200mg/week, 200mg every other week and 200mg every three weeks has the potential to significantly reduce MRI brain lesions and to minimize side effects including platelet reductions (Table 2 below; Guzy and Bauer, 2012). Given the approximately 3-week tissue half-life of ATL1102, a 200mg dose every 3 weeks equates to approximately 67mg per week or 0.8mg/kg/week in an 80kg patient, which is close to the median 0.7mg/kg once weekly dose planned in this study of patients with DMD trial of 25 to 65kg.

Another phosphorothioate antisense drug, AO19 designed to skip dystrophin exon 19 and 20 mutations, has been tested in a 10 year old non-ambulant DMD patient looking at the effects on circulating lymphocyte dystrophin RNA. Dosed at 0.5mg/kg/week once weekly iv, AO19 reduced the target out of frame dystrophin, and increased the in-frame dystrophin 18-21 RNA levels approximately 50% in circulating lymphocytes by 4 weeks of dosing. AO19 had activity in lymphocytes as early as 3 weeks of dosing, with effects at 4 weeks greater than effects at 3 weeks, suggesting that longer duration dosing may have additional effects on in-frame dystrophin levels in lymphocytes (Takeshima et al, 2006).

Table 2: Pharmacometric Modelling of MRI in ATL1102 Phase 2a Study of Patients with RRMS.

**Dose**	**% Patients expected with Platelets <100/ml at 4 wks**	**% Patients expected with platelets >150/nL at 4 wks**	**Cum, T1 MRI reduction expected at 4-7 months treatment vs. Placebo.**
200mg qw	≤5%	~20%	~60%
200mg q2w	~0%	~0%	~45%
200mg q3w	~0%	~0%	~35%

Safety in DMD trial:

In the Phase 2a MS study most of the platelet reductions occurred within the first month and were closely associated with ATL1102 pharmacokinetics (Guzy and Bauer, 2012). Pharmacometric modelling suggests that at 200mg/week over 4 weeks ~20% of patients will have platelets below normal (150/nl) and <5% of patients will have platelets below 100/nl (see Table 2) at which levels there is an increased risk of bleeding. Modelling suggests at lower doses there will not be significant platelets reductions at 4 weeks. The modelling is consistent with the more recent observations that 200 mg/week of second generation antisense drugs are not associated with platelet reductions (Crooke *et al*, 2016).

It is anticipated in the DMD study at 25mg ATL1102 per week that platelet reductions, injection site erythema and liver enzyme increases should occur at lower frequencies and or with less severity than in the Phase 2 DMD study because of the lower dosing proposed compared with the Phase IIa in MS study.

Thus, clinical studies conducted to date indicate that ATL1102 has a safety (AE) and laboratory profile that is clinically manageable at the proposed dose and duration to be administered. Safety margins are supportive of the 25 mg/week dosing (up to 1 mg/kg dose for the minimum weight of 25kg) with monitoring of relevant clinical pathology parameters and Data Safety Monitoring Board (DSMB) review of the safety data throughout the study. Based on the review of the cumulative data, the potential risks and benefits identified in association with ATL1102 justify the investigation of this investigational product in patients with DMD.

3 STUDY OBJECTIVES AND ENDPOINTS

The objectives of this study are to assess the safety, efficacy, and PK profile of weekly dosing of 25mg of ATL1102 in patients with non-ambulatory DMD.

**Primary Objectives**	**Primary Endpoints**
• To assess the safety and tolerability of 25 mg of ATL1102 administered once weekly in patients with DMD.	• Frequency and intensity of adverse events (AEs)<br>• Frequency and intensity of redness, swelling and pain at the injection site<br>• Change in number and percentage values of the following laboratory tests indicative of vascular and renal inflammation<br>o CRP parameter<br>o Creatinine and Creatinine Clearance parameters<br>• Changes in platelet numbers and percentage<br>• Other Clinical laboratory test and assessments<br>o Haematology<br>o Coagulation<br>o Biochemistry and Liver function

	○ Urinalysis<br>○ Vital signs<br>○ ECG
**Secondary Objectives**	**Secondary Endpoints**
• To investigate the lymphocyte-modulatory potential of ATL1102 in patients with DMD.<br>• To evaluate the PK profile of ATL1102 in patients with DMD.<br>• To evaluate the effects of ATL1102 on functional capacity in patients with DMD.<br>• To evaluate the effects of ATL1102 on respiratory function in patients with DMD.<br>• To evaluate the effects of ATL1102 on quality-of-life in patients with DMD.	Primary efficacy:<br>• Lymphocyte-modulation potential will be determined by assessing the following haematological parameters by cell surface flow cytometry:<br>○ Number and percentages of lymphocytes<br>○ Number and percentages of CD4+ and CD8+ T cells<br>○ Number of CD4+ CD49dhi and CD8+ CD49dhi T cells<br><br>Secondary efficacy:<br>• Functional capacity will be assessed by the number and percentage of change in:<br>○ Changes in the upper limb muscle inflammation score (muscle oedema), as determined by the STIR on quantitative MRI<br>○ Muscle structure score (comprised of muscle atrophy and fat infiltration) as determined by quantitative MRI utilising the 3-point Dixon sequences of upper limb muscle structure<br>○ MyoSet score, as determined by the total of the Myo-Pinch, Myo-Grip, and MoviPlate scores.<br>○ PUL scores<br>○ EK Scale, Version 2, to evaluate strength in non-ambulant participants.<br><br>• Respiratory function will be assessed by:<br>○ Change in maximum inspiratory pressure (MIP) and expiratory pressures (MEP)<br>○ Change in peak expiratory flow (PEF) and cough peak flow (CPF)

	○ Change in forced vital capacity (FVC) and forced expiratory volume in 1 second (FEV1)<br>○ Change in sniff nasal inspiratory pressure (SNIP).<br><br>• Quality-of-life will be assessed by percentage of change in the neuromuscular module of the Pediatric quality of life instrument (PedsQL) score.<br><br>Pharmacokinetic Endpoints:<br><br>• Concentrations of ATL1102 in plasma (single and multiple dose concentration-time profile) including AUC, AUC_{inf}, $AUC_{0\text{-}last}$, C_{max}, C_{min}, C_{trough}, T_{max}, and $T_{1/2.}$
**Exploratory Objectives**	**Exploratory Endpoints**
• To further evaluate the PD effects of ATL1102 in patients with DMD.	Blood samples will be collected to:<br><br>• Investigate inflammatory and muscle injury marker changes:<br>○ CRP and other serum markers of inflammation<br>○ CK, AST, and LDH for muscle injury.<br>• Explore the effect of ATL1102 on key haematology cells, purified mononuclear cell RNA, and for proteomic evaluations.

4 STUDY PLAN

4.1 Overall Study Design

This is a single-centre, open label study to assess the safety, efficacy and PK of ATL1102 in non-ambulatory patients with DMD. A weekly ATL1102 dose of 25 mg will be administered subcutaneously for 24 weeks. Nine participants will be enrolled in the study.

A 28-day screening period will be followed by a 24-week active treatment period and an 8-week follow up period. Participants will be enrolled into the study and receive the first dose of investigational product (IP) on Day 1 (Baseline).

Study assessments, which will include evaluations of safety, efficacy and PK parameters, will occur at Screening, Day 1 (Baseline, Week 1), Weeks 3, 5, 7, 8, 10, 12, 14, 16, 18, 20, 22 and 24. Two follow up visits will occur at Week 28 and Week 32.

ATL1102 may be administered at the study site or in the participant's home environment as summarized in 7.14.

A diagram of the study schedule is shown in **Figure 1** below.

Figure 1: Study Flow Diagram

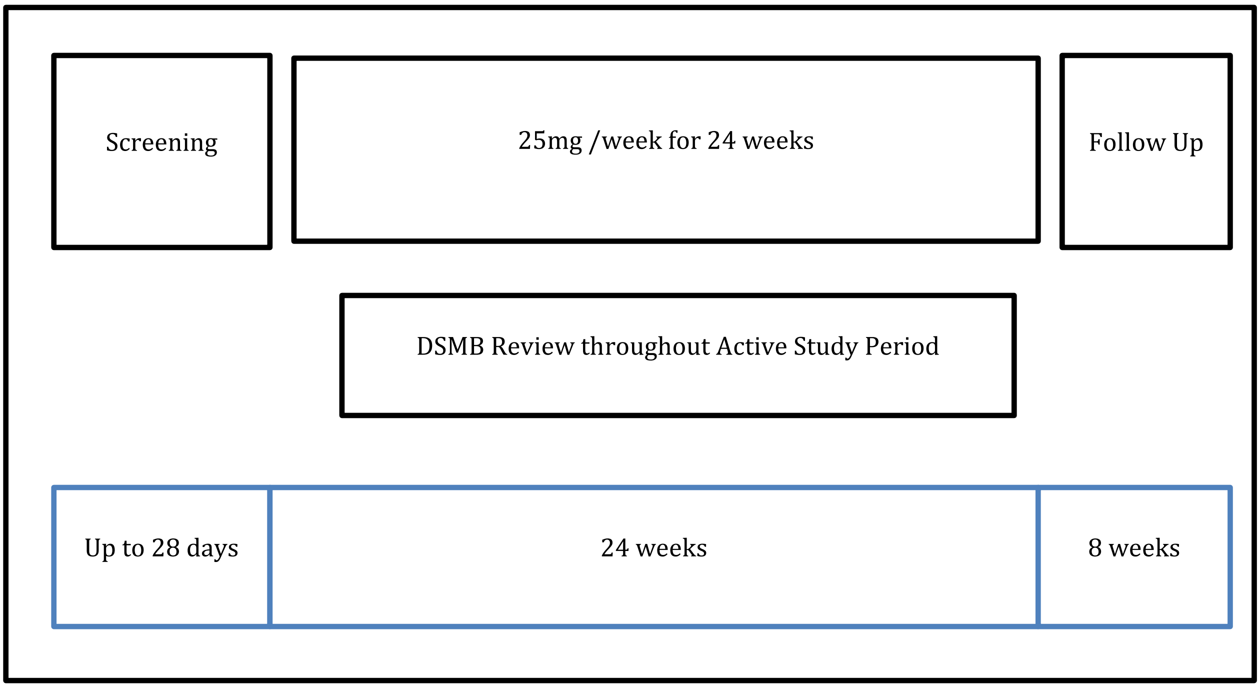

4.2 Number of Study Sites

It is planned for one study site to be involved in the conduct of this study.

4.3 Number of Participants

It is planned that up to 9 adolescence male participants, aged 10 to 18 years inclusive, with non-ambulatory DMD will be enrolled into the study.

4.4 Study Duration

The duration of the study for each participant is up to 36 weeks. This includes a screening period of up to 28 days, followed by 24 weeks of treatment and an 8-week follow up period.

5 STUDY POPULATION

Participant eligibility should be reviewed and documented by an appropriately qualified member of the Investigator's study team before participants are included in the study. All relevant medical and non-medical conditions should be taken into consideration when deciding whether this protocol is suitable for a particular participant.

5.1 Study Selection Record

Investigators must keep a record of all patients with DMD who were considered for the study but were not enrolled.

5.2 Inclusion criteria

Participants must meet all of the following inclusion criteria to be eligible to participate in this study:

1. Are adolescent males, aged 10 to 18 years inclusive, at the time of providing informed consent.
2. Have been diagnosed with Duchenne Muscular Dystrophy and have been non-ambulatory for at least 3 months. Non-ambulatory for this study is consistently requiring a chair to mobilise more than a few steps for at least 3 months.

   The diagnosis of DMD is to be confirmed by at least one of the following:

   a. Dystrophin immunofluorescence and/or immunoblot showing near complete dystrophin protein deficiency, and clinical picture consistent with typical DMD; or
   b. Gene deletions test positive (missing one or more exons) of the dystrophin gene, where reading frame can be predicted as 'out-of-frame', and clinical picture consistent with typical DMD; or
   c. Complete dystrophin gene sequencing showing an alteration (point mutation, duplication, or other mutation resulting in a stop codon) that can be definitely associated with DMD, with a typical clinical picture of DMD; or
   d. Positive family history of DMD confirmed by one of the criteria listed above in a sibling or maternal uncle, and clinical picture typical of DMD.
3. Have a body weight of more than 25 and less than or equal to 65 kg.
4. If currently receiving glucocorticoid therapy, have been on a stable dose of glucocorticoid therapy for at least 3 months prior to Day 1.
5. Are currently on stable doses of cardiac therapy (including angiotensin converting enzyme inhibitors, aldosterone receptor antagonists and/or beta blockers) for at least 3 months prior to Day 1.
6. Have a parent/guardian who is capable of understanding the purposes and risks of the study and able to provide written informed consent. If the participant is of sufficient maturity and has the ability to understand the nature and consequence of the study, involvement in study consent discussions is required.
7. Are able, and have a parent/guardian who are, willing and able to comply with scheduled visits, study drug administration plan, and study procedures.

5.3 Exclusion criteria

If one or more of the following exclusion criteria are met, the participant will be ineligible to take part in this study:

1. Have been diagnosed with Duchenne Muscular Dystrophy and are still ambulatory. Ambulatory for this study is being able to complete at least 75 meters during the 6-minute walk test in the 4 weeks prior to Day 1.
2. Have the following abnormal haematology values during the Screening period or on Day 1, prior to first dose:
   a. Lymphocytes <1.2 x 10^9/L
   b. Neutrophils <1.8 x 10^9/L
   c. Platelets <150 x 10^9/L
3. Have a history of clinically significant bleeding or coagulation abnormalities.
4. Have hepatic dysfunction indicated by an abnormal total bilirubin and gamma glutamyl transferase (GGT) results at Screening

5. Have renal impairment indicated by serum creatinine ≥2.0 mg/dL at Screening.
6. Have uncontrolled clinical symptoms and signs of congestive heart failure consist with Stage C or Stage D criteria according to the American College of Cardiology/American Heart Association guidelines for cardiac dysfunction within 3 months of Day 1.
7. Have an inability to complete the cardiac, pulmonary or strength range of motion and mobility assessments at Screening.
8. Have taken nutritional, herbal, or antioxidant supplements that have a known demonstrated activity for maintaining or improving skeletal muscle strength or functional mobility within 4 weeks of Day 1. NOTE: daily multivitamin, Vitamin D or calcium supplements are permitted.
9. Are currently receiving antiplatelet or anticoagulant therapy, or have taken medication with an antiplatelet or anticoagulant effect within 4 weeks prior Day 1 (e.g. aspirin).
10. Have received any investigational product in the 2 months prior to Screening (4 months if the previous drug was a new chemical entity), whichever is longer.
11. Have severe behavioural disorder or inadequate cognitive development that would make them unable to comply with the study assessments, which, in the opinion of the investigator, makes the participant unsuitable for participation in the study.

5.4 Participant Completion and Withdrawal

5.4.1 Completion of Participant from Study

A participant will have completed the study once all of their specific study-related procedures have been conducted and a final assessment (Week 32) has been performed (approximately 8 weeks after the participants' last dose).

Any AEs or SAEs still ongoing after the final study visit will be followed-up in accordance with Section 10.

5.4.2 Withdrawal of Participants from Study

Participants can terminate their study participation at any time and without giving a reason, without prejudice to further treatment. Participants who discontinue from the Study should always be asked about the reason(s) for their discontinuation and about the presence of any AEs. If possible, they should be seen and assessed by an investigator and have a safety follow-up visit 28 days after stopping IP.

The Investigator or the Antisense Therapeutics Limited (ATL) Medical Monitor can exclude a participant from further taking part in the Study.

Possible reasons for discontinuing a participant may include:

- Participant withdrawal of consent
- Any unacceptable AEs, in the judgement of the Investigator
- Participant's non-compliance with the protocol or dosing requirements
- New intercurrent diseases, which may influence the effect of the Study treatment
- The participant commencing a prohibited medication
- Technical reasons e.g. change of physician/Investigator, change of residence

If a decision is made to discontinue a participant from treatment, the Week 28 visit assessments should be conducted at that time, as the end of study assessment. Additionally, if possible, the follow-up MRI

should be performed if the participant withdraws prior to either Week 12, or between Week 13 and Week 24.

If a participant withdraws consent and does not wish to attend for any further visits, they should be encouraged to attend a final visit (with Week 28 procedures to be performed at this visit) for safety follow up as a minimum. If the participant also withdraws consent for disclosure of future information, no further evaluations should be performed, and no additional data should be collected. The Sponsor may retain and continue to use any data collected before such withdrawal of consent.

5.4.3 Procedure for Handling Withdrawals

In the event that the participant withdraws from the study, the Investigator should record the date of the withdrawal, the person who initiated withdrawal and the reason for withdrawal in the source documents. This information is to be entered into withdrawal page of the participant's Case Report Form (CRF).

5.4.4 Premature Termination of Study

Antisense reserves the right to discontinue the clinical study at any stage for any reason including commercial considerations.

5.4.5 Replacement of Participants

Participants who withdraw from the study prior to, or do not complete at least 12-weeks of treatment will be replaced. If a participant withdraws after Week 12, no replacement will occur.

6 SAFETY MANAGEMENT PROCEDURES

6.1.1 Data Safety Monitoring Board

An independent Data Safety Monitoring Board (DSMB) will be established prior to study initiation, with an appropriate charter to review data and monitor safety results prepared.

The DSMB will determine if further formal meetings and/or reviews/discussions of the results will be required during the study, in addition to the following pre-specified intervals throughout the study (at a minimum):

- After two participants have completed 4 weeks of treatment
- After four participants have completed 8 weeks of treatment
- After nine (all) participants have completed 12 weeks of treatment.

The core DSMB members will comprise four individuals with appropriate experience in the area of DMD, paediatric medicine, statistics, and the conduct of and safety monitoring of clinical studies. Non-core members of the DSMB, such as the Site Investigator, will only attend the open sessions to provide clarification on issues relating to the conduct of the study and will have no voting rights.

The main purpose of the DSMB will be to protect the interests of the participants included in the study. Stopping rules based on safety will be used as guidelines only and will not be the only basis on which to recommend stopping the study. Additional guidelines will be determined by the DSMB if/as required, and documented in the charter prior to study start.

The DSMB will convey to Antisense Therapeutics Limited their recommendations if the study may continue as planned or whether there are any concerns. Minutes will be kept of all meetings.

The final decision on whether the study should be stopped at any time will be the responsibility of Antisense Limited. Any decision to stop will be communicated to Investigators and ethics committee.

6.1.2 Stopping Rules / Discontinuation Criteria

Stopping Rule Guidance: If any of the stopping criteria described below is met the participant must be permanently discontinued from further treatment with IP. See Section 5.4 for requirements for completing the study.

6.1.2.1 Stopping Rules for Liver Chemistry Elevations

Due to the elevation of CK following muscle damage in patients with DMD elevations of AST and ALT levels in patients with DMD are commonly 10- to 20-fold greater than the upper limit of normal (ULN). Assessing liver chemistry for identification of drug induced liver injury utilising these parameters is impractical in this study. Due to this, assessment of hepatotoxicity will be based on assessing participants for increases in bilirubin or GGT or INR.

Based on the guidelines established by Abboud and Kaplowitz (2007), the following rules will be implemented, based on the Common Terminology Criteria for AEs, Version 4.0:

Temporary Halting of Study Drug Administration:

- Bilirubin >1.5 – 3.0 x ULN (Grade 2)
- GGT >2.5 – 5.0 x ULN (Grade 2) or
- INR >1.5-2.5X ULN (Grade 2)
- A significant increase in ALT by > 150 U/L from Baseline ALT level (Day 1 result)

With any of the above results, assessment of the participants CK levels is required. If the CK levels are stable or decreasing from the Baseline levels, further evaluation for hepatotoxicity is required, and an increase in the frequency of laboratory monitoring implemented.

Permanent Halting of Study Drug Administration:

If, at two consecutive measurements (not less than 7 days nor more than 10 days apart) the following occurs:

- Bilirubin >1.5 – 3.0 x ULN (Grade 2)
- GGT >2.5 – 5.0 x ULN (Grade 2) or
- INR >1.5-2.5 X ULN (Grade 2)

OR, if at one measurement, the following occurs

- Bilirubin >3.0 – 10.0 x ULN (Grade 3) or higher
- GGT >5.0 – 20.0 x ULN (Grade 3) or higher.
- INR > 2.5 X ULN (Grade 3) or higher

6.1.2.2 Safety Monitoring Rules for Liver Chemistry Elevations

In the event of a confirmed Grade 2 bilirubin or GGT or INR measurement **at any time during the study** (treatment or follow up period), the following should be done;

- Participants with confirmed Grade 2 bilirubin or GGT or INR levels that are continuing to rise should have their liver chemistry tests (ALT, AST, alkaline phosphatase, international normalised ratio (INR), GGT and total bilirubin) retested **at least** once weekly until levels stabilise and bilirubin and INR levels become ≤ 1.5 x ULN and GGT levels ≤ 2.5 x ULN.
- Obtain a more detailed history of symptoms and prior and concurrent diseases.
- Obtain further history for concomitant drug use (including non-prescription medications, herbal and dietary supplement preparations, alcohol use, recreational drug use), and special diets.
- Obtain a history for exposure to environmental chemical agents and travel.
- Perform serology for viral hepatitis (Hepatitis A virus immunoglobulin M (IgM), Hepatitis B surface antigen, Hepatitis C virus antibody), cytomegalovirus IgM, and Epstein-Barr virus antibody panel.
- Perform serology for autoimmune hepatitis [e.g., antinuclear antibody (ANA)].

Additional liver evaluations, including additional liver activity parameters, gastroenterology/hepatology consults, hepatic computer tomography or MRI scans may be performed at the discretion of the Investigator, in consultation with the Medical Monitor. Repetition of the above evaluations should be considered if a participant's bilirubin and/or GGT and/or INR levels reach Grade 3 levels.

Dosing is to be halted during the safety monitoring period.

6.1.3 Stopping Rules for Renal Function Test Results

Urinalysis by dipstick test will be performed weekly throughout the dosing period. The Investigator will be informed immediately if the protein rises to 2 or above.

Dosing of a participant will be postponed in the event of the following confirmed results at any time during the study:

- Serum creatinine increase ≥ 0.3 mg/dL (26.5 µmol/L) **or** ≥ 40% (whichever is greater) above baseline creatinine values. In recognition that creatinine values may be generally lower in the age-group eligible for the study and as a result of DMD, this stopping criterion will be assessed together with other evidence of deteriorating renal function, including of immunological origin (see other relevant stopping rules below). If, in the opinion of the PI and the medical monitor, a creatinine elevation is not in itself of clinical concern and there is no other evidence to support emerging renal dysfunction, dosing of a participant can be allowed to continue.
- Proteinuria, dipstick ≥ 2+ (confirmed by dipstick re-measurement and then further confirmed by a quantitative total urine protein measurement of > 1.0 g/24hr).

If dosing is postponed, the participant will return for retest the following week (7 days from the day of initial result blood draw or urine test). In the event of a persistent elevation that is observed over 2 consecutive weeks, for either of the two criteria, and which continues to warrant withholding IP dosing, the IP will be **stopped permanently** for that participant.

The follow-up schedule for any events meeting either of these stopping criteria will be determined by the Investigator in consultation with the Medical Monitor.

6.1.4 Stopping Rule for Platelet Count Results

IP dosing will be **stopped permanently** for a participant in the event of a confirmed platelet count less than 75 x 10^9/L. The follow-up schedule for any event that meets this criterion will be determined by the Investigator in consultation with the Medical Monitor.

6.1.4.1 Safety Monitoring Rules for Platelet Reductions

In the event of a confirmed platelet measurement < 100 x 10^9/L or a decrease of > 30% from Baseline at any time during the study (treatment or post-treatment period), the following should be done:

- Participants with confirmed platelet level decrease of > 30% from baseline that are continuing to decrease should have their platelet counts retested at least once weekly until levels stabilise and platelet levels remain >100 x 10^9/L.
- Obtain further history for concomitant drug use (including non-prescription medications, herbal and dietary supplement preparations, alcohol use, recreational drug use), and special diets.
- Dose reductions in response to platelet reductions can be considered on a case-by-case basis after discussion with the medical monitor.

6.1.5 Stopping rule for C-reactive protein

In the event of a confirmed CRP value of > 30 mg/L recorded at two consecutive clinic visits (one month apart) which could not be explained by other symptoms or events (e.g. infection, inflammation flare) the study drug will be stopped permanently in that participant.

Specimens for confirmatory CRP assays should be taken ideally 3 to 4 days after the last dose of study drug that the participant received, and after treatment/resolution of any symptoms or event that could explain the CRP elevation.

6.1.6 Stopping Rule for Albumin and A/G ratio Complement C3

The Albumin, A/G ratio and complement C3 stopping rule is based on identification of biomarker data from two of the ATL1102 monkey toxicology studies in which mild A/G ratio decreases preceded more notable increases in complement activation and other potential markers (e.g. CRP). In animals with vasculitis there was a reduction in both A/G ratio and complement C3.

- In the event of a confirmed decrease in albumin or A/G ratio of 25% from Baseline and confirmed decrease in complement C3 of more than 20% from Baseline at any time during the study continues at 2 consecutive visits (a minimum of 2 weeks apart), and which cannot be explained by other symptoms or events, the study drug **will be stopped permanently** in that participant.

6.1.6.1 Safety Monitoring of Albumin and A/G Ratio, IgG and C3

In the event of a confirmed decrease in Albumin, or A/G ratio of > 25% from baseline at any time during the study (during treatment or the follow up period), the following should be done:

- Participants should have their total protein, Albumin, A/G, IgG tested at least once fortnightly thereafter.
- If IgG is increased 30% from baseline, participants should have their Baseline (Day 1) and Week 12 samples analysed for IgG1, monocyte chemotactic protein 1 (MCP1), and complement C3. Participants should also have their monocyte chemotactic protein (MCP-1) and complement C3 tested at least once fortnightly thereafter.

- Obtain further history for concomitant drug use (including non-prescription medications, herbal and dietary supplement preparations, alcohol use, recreational drug use), and special diets.
- Treat on a case-by-case basis after discussion with the Medical Monitor.
- In the event of a confirmed decrease in Albumin or A/G ratio of 25% and confirmed increase in IgG of 30% and confirmed increase MCP-1 of 80% and confirmed decrease of complement C3 of more than 20%, the Baseline (Day 1) and Week 12 samples are to be analysed for anti-neutrophil cytoplasmic antibody (ANCA) and ANA.

6.1.6.2 Additional Safety Monitoring of Haematology, Haptoglobin, Fibrinogen and MCP-1

Biweekly tests for Haematology (including platelets, RBC) and haptoglobin and fibrinogen will be assessed for any clinically significant changes. Bi-weekly samples will be collected for testing MCP-1.

6.1.7 **Contraception requirements**

Studies in mice and rabbits indicate that ATL1102 does not adversely affect fertility, or have any adverse effect on embryo-foetal development at maternally non-toxic doses. Data in pregnant women, however, does not exist; therefore adequate forms of contraception are required for participants enrolling in ATL1102 clinical studies. As a precaution male participants should avoid fathering children for at least three months after receiving their last dose of ATL1102.

If the participant or their partner meets one of the following criteria, there is no requirement to use contraception:

- A female who has undergone surgical sterilisation (e.g. tubal occlusion, hysterectomy, bilateral salpingectomy, or bilateral salpingo-oophorectomy).
- A male who has been vasectomised for at least six months, with documentation of no sperm in the ejaculate.
- True abstinence in line with the preferred and usual lifestyle of the participant.

For all other participants, they must be advised to use one or more highly effective methods of contraception, if they intend to engage in sexual intercourse with a partner of the opposite sex during the study or within three months of receiving their last dose of ATL1102.

Highly effective methods of contraception are those with a failure rate of less than 1% per year and include;

- Implants
- Injectables
- Combined oral contraceptive pill
- Progesterone or copper eluting intrauterine device (IUD)

A condom is acceptable as a second (additional) form of contraception.

If a partner of a male participant does become pregnant during the periods specified above, they will be asked to inform the investigator as soon as possible.

6.1.8 Concomitant Medications and Treatments

Participants may not commence any new medications indicated for DMD during the study, including during the follow up period. If the participant needs to start another medication for DMD, they must be withdrawn from the study treatment. They should complete the Week 24 visit assessments prior to commencing the new medication, and if possible, attend the site for a follow up visit after the last dose of ATL1102 (see Section 5.4).

Concomitant medication is only permitted if it is indispensable due to intercurrent acute or chronic diseases or conditions.

- Paracetamol and Ibuprofen are permitted to treat acute conditions, such as fever or temporary pain (e.g., headache), following discussion by the participant (or their carers) with the study Investigators to confirm suitability of use. The maximum doses permitted by the manufacturers are not to be exceeded.
- The dose of concomitant medication required for a chronic disease should be kept as constant as possible throughout the study. Full details of any medication administered (trade name, dosage regimen) have to be recorded in the participant records. Any physical or other form of treatment must also be recorded.

The following medications and treatments are specifically **not permitted** for the specified periods prior to, and during, the study;

- Aspirin, at any dose
- No non-steroidal anti-inflammatory drug (NSAID) are permitted within 24 hours prior to each scheduled visit.

At the end of the follow up period the participant’s doctor will discuss appropriate future treatment for their DMD as required.

7 STUDY TREATMENT

7.1.1 Description of the Investigational Product

The ATL1102 drug product formulation is a 150 mg/mL sterile, aqueous solution containing the ATL1102 drug substance in water for injection with a small amount of either HCl or NaOH to adjust the pH of the solution to 7.4.

ATL1102 will be supplied as a sterile solution in glass vials with an approximate 0.7mL volume. The vial overage allows for the withdrawal of 0.67 mL (100 mg).

ATL1102 solution must not come into contact with amber glass or amber plastic.

7.1.2 Manufacture, Packaging and Labelling

The drug substance was manufactured by Nitto Avecia Inc., Milford, Massachusetts, USA. The drug product, ATL1102, was manufactured by PYRAMID Laboratories, Inc., Costa Mesa, California, USA, in accordance with *Good Manufacturing Practices Annex 13: Manufacture of Investigational Products* and local regulatory requirements.

ATL1102 will be labelled in accordance with all applicable regulatory requirements.

The IP will be supplied in glass vials to the study site, with an outer carton packaging, after receipt of required documents in accordance with all applicable regulatory requirements and ATL procedures.

7.1.3 Investigational Product Handling and Storage

At the study site, ATL1102 should be stored under temperature-monitored conditions (2ºC to 8ºC) in a secure area with limited access, protected from light.

The solution should be allowed to reach ambient temperature for approximately 30 minutes prior to dosing. Upon drawing into the delivery syringe, IP must be administered within 2 hours.

Only participants enrolled in the study may receive IP. Authorised study personnel will dispense the IP according to the sequential enrolment of participants, following the sites normal procedures for dispensing a pharmacy-controlled medication. All used and unused vials must be maintained until accountability has been completed.

Refer to the Pharmacy and Study Manuals for further details about the storage and preparation of ATL1102 for administration.

7.1.4 Dosage and Administration

A dose of 25 mg ATL1102 will be assessed. Based on the vial volume of 0.67 mL (containing 100 mg of ATL1102), the dose volume for each participant is 0.17mL.

All doses of ATL1102 will be administered by SC injection, into healthy and intact skin, with the abdomen being the preferred administration area.

All participants will receive IP at either the study site (S) or in the home (H) environment on Weeks 1 to Weeks 24 as follows:

Week 1 – Day 1 (S)	Week 2 – Day 8 (H)	Week 3 – Day 15 (S)	Week 4 – Day 22 (H)
Week 5 – Day 29 (S)	Week 6 – Day 36 (H)	Week 7 – Day 43 (S)	Week 8 – Day 50 (H)
Week 9 – Day 57 (H)	Week 10 – Day 64 (S)	Week 11 – Day 71 (H)	Week 12 – Day 78 (H)
Week 13 – Day 85 (H)	Week 14 – Day 92 (S)	Week 15 – Day 99 (H)	Week 16 – Day 106 (S)
Week 17 – Day 113 (H)	Week 18 – Day 120 (S)	Week 19 – Day 127 (H)	Week 20 – Day 134 (S)
Week 21 – Day 141 (H)	Week 22 – Day 148 (S)	Week 23 – Day 155 (H)	Week 24 – Day 162 (H)

If an injection is not tolerated, the utilisation of ice prior to the next injection is appropriate. Prior to subsequent IP injections the participant should apply ice (wrapped in a towel) to the proposed injection site for approximately 10 minutes. Post injection, the participant should apply ice for approximately 10 more minutes while resting. These measures may reduce the occurrence of injection site reactions.

If the injection is still not tolerated with icing, the investigator may request sponsor approval to inject into the back of the upper arm or thigh.

The participant and/or their parent/guardian must capture any injection site reactions (redness, swelling, pain), and other information including whether the injection site was treated with ice or not, and whether the injection was given in the abdomen, upper arm or thigh, in the take home diary card.

7.1.5 Compliance of Investigational Product

To ensure that participants receive the correct dosage of IP, the following procedures are required:

For IP administration at the site:

Two authorised study site personnel will prepare Study Product for administration. The first individual will prepare the dose of IP with the second individual confirming the dose and volume of the IP injection. The appropriately trained study site personnel will administer the dose of ATL1102.

For IP administration in the home environment:

The person who will administer the IP in the home environment (either a visiting nurse or the participant's parent/guardian) will be trained by the study site personnel on how to prepare the dosage correctly and how to administer the injection prior to the administration of the IP being permitted in the home environment. Following approval from the site personnel for home administration to occur, the site pharmacist will dispense the correct number of vials for the dose of IP in the home environment. The visiting nurse or the participant's parent/guardian will prepare the correct dosage of ATL1102 and administer the IP.

The dose and volume will be documented on the IP Dispensing and Accountability Form, Administration forms, Participant Diary (for all home administrations) and captured in the CRF. The actual time and location of the dose administration will also be recorded.

Participants will be considered compliant if they administer ±20% of expected doses. Lack of treatment compliance will not necessarily lead to discontinuation from the Study.

7.1.6 Accountability of Investigational Product

Investigational Product must only be used for participants enrolled in this clinical study and should not be used for any other purpose.

The Investigator or designee is responsible for IP storage, dispensing, accountability, reconciliation and record maintenance. The Investigator or designated site staff must maintain IP accountability records throughout the course of the study including records of the amount of IP received, the identification of the participant for whom the drug was dispensed, the date(s) and quantity of the drug dispensed and the amount returned by participants.

The IP vials and records must be available for inspection by a study monitor during the study. IP supplies, including unused, partially used or empty vials, will either be returned by the study site to ATL or their agent, or destroyed on site if written approval to do so is given by ATL and appropriate facilities and procedures are available. After study completion, it must be possible to reconcile delivery records with those records of used and unused IP.

Records shall be maintained by the Investigator of any disposition of the IP. These records must show the identification and quantity of IP disposed of, the method of destruction (taking into account the requirements of local law), and the person who disposed of the IP. Where IP is destroyed on-site, a record of destruction shall be issued and submitted to ATL.

At the end of the Study an overall drug accountability will be generated to check the conformity of the number of dispensed and used/lost/returned IP vials.

7.1.7 Dose Frequency Adjustment

If a participant experiences an AE requiring (in the Investigator's opinion) temporary suspension of IP, the IP should only be restarted after the AE has resolved or stabilised. For any serious or severe AE causing

suspension of dosing, written approval should be obtained from the Medical Monitor prior to resuming IP.

If a participant is not tolerating the IP that is reported as an AE, and in the Investigator's opinion may require temporary suspension of IP, the Investigator may discuss with the Medical Monitor the option of restarting the IP after the AE has resolved or stabilizes, at a reduced dose frequency of once every two weeks. Any changes to dosing frequency, or injection site location, must be agreed by the Medical Monitor prior to implementation.

8 STUDY PROCEDURES

The timing and frequency of the study assessments are described in the Schedule of Assessments. Below is a summary of the assessments required at each study assessment.

8.1 Participant Information and Consent

The Investigator / delegate will recruit participants through their normal clinic practice. Adequate information must be provided regarding the study conduct to the parent/guardian of the participant and if appropriate, for the participant to be included in this discussion. The parent/guardian will be given time to read and understand the Parent/Guardian Participant Information and Consent Form (PICF) and have any questions answered. They may wish to take the Parent/Guardian PICF and consider it further, or to discuss it with their family or their usual doctor before signing.

Written informed consent must be obtained from the parent/guardian of the participant before any tests or investigations outlined in the study protocol are carried out. The Parent/Guardian PICF must be personally signed and dated by both the Investigator and the participant's parent/guardian.

As evaluated by the Investigator, based on the participant's maturity and understanding to grasp the nature and consequence of the study, the participant will be provided with a Participant PICF to review to allow them to give their consent, in addition to the parent/guardian consent.

8.1.1 Method of assigning treatment

A participant identification (ID) number will be allocated to each participant who is consented for the study so that participants can be identified without making assumptions about their subsequent eligibility for the Study. Participants will be allocated to sequential, ascending 3-digit ID numbers (001, 002, 003 *etc.*), which in combination with the 2-digit site number will provide a unique ID for each participant. The participant will retain the same ID number for the duration of the study. If a participant fails screening and is completely re-screened (see Section 8.2.1.1), they will retain their assigned participant ID number.

If a participant fails screening and is not randomised, or discontinues from the Study, the participant ID number will not be reused.

Participant eligibility will be established before enrolment on Day 1.

8.2 Study Procedures

Study assessments, which will include evaluations of safety, efficacy and PK parameters, will be conducted during the Screening period (of up to 28 days prior to Day 1), the 24-week active treatment period and the 8-week follow up period. Participants will be enrolled into the study on Day 1.

Each study visit due date is calculated from the Day 1 visit.

8.2.1 Screening Visit (Day -28 to Day -1) – Assessment 1

The purpose of the Screening visit is to confirm participant eligibility against inclusion and exclusion criteria. If the participant is taking a corticosteroid as part of their concomitant medication regimen, the study site are to contact the participant on the day PRIOR to the screening visit to remind the participant NOT to take their steroid dose on the morning of screening. The participant is to bring the medication with them. Following the completion of blood sampling procedures, the participant will be able to take their steroid medication. This reminder is to also confirm that no NSAIDs are permitted within 24 hours prior to the screening visit.

In this study, participant eligibility must be determined within the 28 days prior to the intended date of dosing (Day 1, Baseline). Screening assessments may be conducted on different days during the Screening period if required due to timing, participant fatigue, or participant/carer availability.

All participants must have written informed consent provided for them before any study-specific assessments or procedures are performed.

The assessments to be performed at the screening visit include:

- Review of inclusion and exclusion criteria
- Demographics and Medical history, including diagnosis and history of DMD
- Concomitant medications
- Height and body weight – body surface area will be calculated from these results in the database
- Physical examination
- Vital signs (blood pressure [resting], heart rate, body temperature [tympanic measurement])
- 12-lead ECG; triplicate measurement (resting)
- Clinical laboratory assessments:
    - Haematology
    - Biochemistry
    - Urinalysis
    - Coagulation test
- MRI Upper Limbs
    - The MRI does not need to be conducted on the same day as the other screening assessments, only within the Screening period and prior to Day 1.

Participants who complete all of these assessments (with the exception of MRI) and who fulfil the eligibility criteria will be enrolled into the study. MRI assessment is required however, is only to be performed where possible.

8.2.1.1 Re-Screens

A repeat of an individual screening assessment (e.g., laboratory tests, ECG) is permitted if the result is considered to be unusual for that participant. If the participant is taking a corticosteroid as part of their concomitant medication regimen, the study site are to contact the participant on the day PRIOR to the re-screening visit to remind the participant NOT to take their steroid dose on the morning of re-screening. The participant is to bring the medication with them. Following the completion of blood sample procedures, the participant will be able to take their steroid medication. This reminder is to also confirm that no NSAIDs are permitted within 24 hours prior to the re-screening visit.

If appropriate, and with prior approval from ATL or their designee, participants who fail screening initially may be completely re-screened.

Eligible participants must be enrolled within 28 days of the first screening assessment. Approval to enrol later than 28 days must be obtained from ATL, and selected screening procedures may be required to be repeated first (e.g., laboratory assessments, ECGs, review all medical history, concomitant medications and review of entry criteria).

8.2.2 **Active Treatment Period – Assessment Numbers 2 to 14**

If the participant is taking a corticosteroid as part of their concomitant medication regimen, the study site are to contact the participant on the day PRIOR to each scheduled dosing day (for site or home administration) to remind the participant NOT to take their steroid dose on the morning of dosing. For all IP administrations occurring at the study site, the participant is to bring the medication with them. Following the completion of pre-dose blood sample collection, the participant will be able to take their steroid medication. This reminder is to also confirm that no NSAIDs are permitted within 24 hours prior to each scheduled visit.

The participant is to attend the study site a total of 10 times in the active treatment period on Day 1, Day 15, Day 29, Day 43, Day 64, Day 92, Day 106, Day 120, Day 134, and Day 148 to receive their dose of ATL1102. All fourteen other weekly doses of ATL1102 may be administered in the home environment if the participant choses.

The participant is to attend the study site up to a total of 14 times in the active treatment period on Day1, Day 15, Day 29, Day 43, **Day 53**, Day 64, **Day 81**, Day 92, Day 106, Day 120, Day 134, Day 148, and **Day 165** for efficacy and safety assessments and collection of blood samples. The Week 1, Day 1 Baseline visit is an extended visit to accommodate the post dose PK sampling to 6 hours post dose administration and therefore, may be performed over 2 consecutive days where the pre-dose assessments for Functional Capacity and the Quality-of-Life assessment can be performed a day prior to Day 1, where all other pre-dose assessments, dose administration and post dose assessments will be performed.

8.2.2.1 Assessment 2: Week 1, Day 1 - Baseline

Participants who meet all of the inclusion and none of the exclusion criteria will be scheduled to return to the site for their Day 1, Baseline visit. The Baseline visit may occur over 2 consecutive days where pre-dose assessments for Functional Capacity and the Quality-of-Life assessment can be performed a day prior to Day 1, where all other pre-dose assessments, dose administration and post dose assessments are to be performed. At Baseline, the following will be performed *PRIOR* to the administration of the ATL1102 dose:

- Review of inclusion and exclusion criteria
- Physical examination

- Body weight
- Vital signs
    - Assessment of BP, pulse and temperature will occur within 1 hr prior to ATL1102 injection
- 12-lead ECG (triplicate) to occur within 1 hour prior to ATL1102 dose
- Clinical laboratory assessments:
    - Haematology
    - Biochemistry
    - Coagulation
    - Urinalysis
- Pharmacodynamic assessments
    - Lymphocyte Cells sample
    - Complement C3 and Bb sample
    - Immunogenicity sample
    - Exploratory PD sample
- Pharmacokinetic assessment
    - A blood sample will be collected before dosing for baseline plasma PK assessment
- Functional Capacity assessment
    - PUL, version 2
    - MyoSet, including the MyoPinch, MyoGrip, MoviPlate
    - EK Scale, version 2
- Respiratory Function assessment
    - SNIP, PEF, CPF, FVC, FEV1, MIP, and MEP
- Quality-of-Life assessment
    - The PedsQL will be completed by the participant (or carer, if participant unable to complete)
- Review of concomitant medications and AEs (NOTE: If any AEs reported, these are to be recorded as pre-treatment AEs if present prior to administration of IP).

Following completion of the above assessments, the first dose of ATL1102 will be administered subcutaneously.

The participant will be reminded to take their daily dose of corticosteroids (if applicable) once pre-dose blood sample collection has been completed, per their normal regimen.

The assessments to be performed *AFTER* administration of ATL1102 are:

- Vital signs
    - Assessments of BP, pulse and temperature will occur at 1, 2 and 6 hours after ATL1102 injection.
- 12-lead ECG (triplicate) to occur at least 1 hour post ATL1102 dose
- Injection site tolerability assessment
    - Assessments of redness, swelling and pain at the injection site will occur at 1, 2 and 6 hours after the ATL1102 injection.
    - The participant and their parent/guardian are to be provided with a diary card to take home to capture any site reactions (redness, swelling, pain).
- Clinical laboratory assessments – For participants taking corticosteroids ONLY.
    - Haematology – sample to be collected approximately 4 hours after the participant has taken their corticosteroid medication.

- Pharmacokinetic assessment
  - A blood sample will be collected at 1, 2, 3, 4 and 6 hours after the ATL1102 dose has been administered for plasma PK assessment
- Review of AEs.

8.2.2.2 Assessment 3: Week 3, Day 15

The participant is to attend the study site to receive their scheduled dose of ATL1102. *PRIOR* to receiving ATL1102, the following will be performed:

- Vital signs
  - Assessment of BP, pulse and temperature will occur within 1 hour prior to ATL1102 injection
- Clinical laboratory assessment
  - Haematology
  - Biochemistry – haptoglobin only
  - Coagulation – fibrinogen only
  - Urinalysis
- Pharmacodynamic assessment
  - Immunogenicity sample – MCP-1 sample only
- Review of concomitant medications and AEs.

Following completion of the above assessments, the dose of ATL1102 will be administered subcutaneously.

The assessments to be performed *AFTER* administration of ATL1102 are:

- Injection site tolerability assessment
  - Assessments of redness, swelling and pain at the injection site will occur prior to discharge.
  - The participant and their parent/guardian are to be provided with a diary card to take home to capture any site reactions (redness, swelling, pain).
- Review of AEs.

8.2.2.3 Assessment 4: Week 5, Day 29

The participant is to attend the study site to receive their scheduled dose of ATL1102. *PRIOR* to receiving ATL1102, the following will be performed:

- Physical Examination
- Body weight
- Vital signs
  - Assessment of BP, pulse and temperature will occur within 1 hour prior to ATL1102 injection
- Clinical laboratory assessments:
  - Haematology
  - Biochemistry - including haptoglobin
  - Coagulation – including fibrinogen
  - Urinalysis
- Pharmacodynamic assessments
  - Lymphocyte Cells sample

  - Complement C3 and Bb sample
  - Immunogenicity sample
  - Exploratory PD sample
- Pharmacokinetic assessment
  - A blood sample will be collected before dosing for baseline plasma PK assessment
- Functional Capacity assessment
  - PUL, version 2
  - MyoSet, including the MyoPinch, MyoGrip, MoviPlate
  - EK Scale, version 2
- Respiratory Function assessment
  - SNIP, PEF, CPF, FVC, FEV1, MIP, and MEP
- Review of concomitant medications and AEs

Following completion of the above assessments, the dose of ATL1102 will be administered subcutaneously.

The assessments to be performed *AFTER* administration of ATL1102 are:

- Injection site tolerability assessment
  - Assessments of redness, swelling and pain at the injection site will occur prior to discharge.
  - The participant and their parent/guardian are to be provided with a diary card to take home to capture any site reactions (redness, swelling, pain).
- Review of AEs.

8.2.2.4 Assessment 5: Week 7, Day 43

The participant is to attend the study site to receive their scheduled dose of ATL1102. *PRIOR* to receiving ATL1102, the following will be performed:

- Clinical laboratory assessment
  - Haematology
  - Biochemistry – haptoglobin only
  - Coagulation – fibrinogen only
  - Urinalysis
- Pharmacodynamic assessment
  - Immunogenicity sample – MCP-1 sample only

Following completion of the above assessments, the dose of ATL1102 will be administered subcutaneously.

The assessments to be performed *AFTER* administration of ATL1102 are:

- Injection site tolerability assessment
  - Assessments of redness, swelling and pain at the injection site will occur prior to discharge.
  - The participant and their parent/guardian are to be provided with a diary card to take home to capture any site reactions (redness, swelling, pain).
- Review of AEs.

8.2.2.5 Assessment 6: Week 8, Day 53

The dose of IP for this week is to be administered 3 days PRIOR to this scheduled site visit. The following assessments will be performed:

- Physical Examination
- Vital signs
    - Assessment of BP, pulse and temperature
- Clinical laboratory assessments:
    - Haematology
    - Biochemistry
    - Coagulation
    - Urinalysis
- Pharmacodynamic assessment
    - Lymphocyte Cells sample
    - Complement C3 and Bb sample
    - Immunogenicity sample
    - Exploratory PD sample
- Pharmacokinetic assessment
    - A blood sample will be collected for plasma PK assessment
- Functional Capacity assessment
    - PUL, version 2
    - MyoSet, including the MyoGrip, MyoPinch, MoviPlate
    - EK Scale, version 2
- Respiratory Function assessment
    - SNIP, PEF, CPF, FVC, FEV1, MIP and MEP
- Injection site tolerability assessment
    - The participant and their parent/guardian are to be provided with a diary card to take home to capture any site reactions (redness, swelling, pain).
- Review of concomitant medications and AEs.

8.2.2.6 Assessment 7: Week 10, Day 64

The participant is to attend the study site to receive their scheduled dose of ATL1102. *PRIOR* to receiving ATL1102, the following will be performed:

- Clinical laboratory assessment
    - Haematology
    - Biochemistry – haptoglobin only
    - Coagulation – fibrinogen only
    - Urinalysis
- Pharmacodynamic assessment
    - Immunogenicity sample – MCP-1 sample only

Following completion of the above assessments, the dose of ATL1102 will be administered subcutaneously.

The assessments to be performed *AFTER* administration of ATL1102 are:

- Injection site tolerability assessment

- Assessments of redness, swelling and pain at the injection site will occur prior to discharge.
- The participant and their parent/guardian are to be provided with a diary card to take home to capture any site reactions (redness, swelling, pain).
- Review of AEs.

8.2.2.7 Assessment 8: Week 12, Day 81

The dose of IP for each of these weeks is to be administered 3 days PRIOR to the scheduled week's site visit. The following assessments will be performed:

- Physical examination
- Body Weight
- Vital signs
    - Assessment of BP, pulse and temperature
- Clinical laboratory assessment
    - Haematology
    - Biochemistry
    - Coagulation
    - Urinalysis
- Pharmacokinetic assessment
    - A blood sample will be collected for plasma PK assessment
- Pharmacodynamic assessment
    - Lymphocyte Cells sample
    - Complement C3 and Bb sample
    - Immunogenicity sample
    - Exploratory PD sample
- MRI Upper Limb assessment (this assessment may be performed 1 week prior to or after these scheduled site visits).
- Functional Capacity assessment
    - PUL, version 2
    - MyoSet, including the MyoGrip, MyoPinch, MoviPlate
    - EK Scale, version 2
- Respiratory Function assessment
    - SNIP, PEF, CPF, FVC, FEV1, MIP and MEP
- Quality-of-Life assessment
    - The PedsQL will be completed by the participant (or carer, if participant unable to complete)
- Injection site tolerability assessment
    - The participant and their parent/guardian are to be provided with a diary card to take home to capture any site reactions (redness, swelling, pain).
- Review of concomitant medications and AEs.

8.2.2.8 Assessment 9: Week 14, Day 92

The participant is to attend the study site to receive their scheduled dose of ATL1102. *PRIOR* to receiving ATL1102, the following will be performed:

- Clinical laboratory assessment

    - Haematology
    - Biochemistry – haptoglobin only
    - Coagulation – fibrinogen only
    - Urinalysis
- Pharmacodynamic assessment
    - Immunogenicity sample – MCP-1 sample only

Following completion of the above assessments, the dose of ATL1102 will be administered subcutaneously.

The assessments to be performed *AFTER* administration of ATL1102 are:

- Injection site tolerability assessment
    - Assessments of redness, swelling and pain at the injection site will occur prior to discharge.
    - The participant and their parent/guardian are to be provided with a diary card to take home to capture any site reactions (redness, swelling, pain).
- Review of AEs.

8.2.2.9 Assessment 10: Week 16, Day 106

The participant will be required to attend the study site to receive the dose of ATL1102. *PRIOR* to receiving the ATL112 dose, the following will be performed:

- Physical Examination
- Vital signs
    - Assessment of BP, pulse and temperature will occur within 1 hour prior to ATL1102 injection
- 12-lead ECG (triplicate) to occur within 1 hour prior to ATL1102 dose
- Clinical laboratory assessment
    - Haematology
    - Biochemistry
    - Coagulation
    - Urinalysis
- Pharmacokinetic assessment
    - A blood sample will be collected before dosing for baseline plasma PK assessment
- Pharmacodynamic assessment
    - Complement C3 and Bb sample
    - Immunogenicity sample
    - Exploratory PD sample
- Review of concomitant medications and AEs.

Following completion of the above assessments, the dose of ATL1102 will be administered subcutaneously.

- The assessments to be performed *AFTER* administration of ATL1102 are:
- Vital signs
    - Assessments of BP, pulse and temperature will occur at 1, 2 and 6 hours after ATL1102 injection.
- 12-lead ECG (triplicate) to occur at least 1 hour post ATL1102 dose

- Clinical laboratory assessments – For participants taking corticosteroids ONLY.
  - Haematology – sample to be collected approximately 4 hours after the participant has taken their corticosteroid medication.
- Pharmacokinetic assessment
- A blood sample will be collected at 1, 2, 3, 4 and 6 hours after the ATL1102 dose has been administered for plasma PK assessment
- Injection site tolerability assessment
  - Assessments of redness, swelling and pain at the injection site prior to participant's discharge from the study site.
  - The participant and their parent/guardian are to be provided with a diary card to take home to capture any site reactions (redness, swelling, pain).
- Review of AEs.

8.2.2.10 Assessment 11: Week 18, Day 120

The participant is to attend the study site to receive their scheduled dose of ATL1102. *PRIOR* to receiving ATL1102, the following will be performed:

- Clinical laboratory assessment
  - Haematology
  - Biochemistry – haptoglobin only
  - Coagulation – fibrinogen only
  - Urinalysis
- Pharmacodynamic assessment
  - Immunogenicity sample – MCP-1 sample only

Following completion of the above assessments, the dose of ATL1102 will be administered subcutaneously.

The assessments to be performed *AFTER* administration of ATL1102 are:

- Injection site tolerability assessment
  - Assessments of redness, swelling and pain at the injection site will occur prior to discharge.
  - The participant and their parent/guardian are to be provided with a diary card to take home to capture any site reactions (redness, swelling, pain).
- Review of AEs.

8.2.2.11 Assessment 12: Week 20, Day 134

The participant will be required to attend the study site to receive the dose of ATL1102. *PRIOR* to receiving the ATL112 dose, the following will be performed:

- 12-lead ECG (triplicate) to occur within 1 hour prior to ATL1102 dose
- Clinical laboratory assessment
  - Haematology
  - Biochemistry
  - Coagulation
  - Urinalysis
- Pharmacodynamic assessment
  - Complement C3 and Bb sample

    - Immunogenicity sample
    - Exploratory PD sample
- Review of concomitant medications and AEs.

Following completion of the above assessments, the dose of ATL1102 will be administered subcutaneously. The assessments to be performed *AFTER* administration of ATL1102 are:

- Injection site tolerability assessment
- Assessments of redness, swelling and pain at the injection site prior to participant's discharge from the study site
- The participant and their parent/guardian are to be provided with a diary card to take home to capture any site reactions (redness, swelling, pain).
- Review of AEs.

8.2.2.12 Assessment 13: Week 22, Day 148

The participant is to attend the study site to receive their scheduled dose of ATL1102. *PRIOR* to receiving ATL1102, the following will be performed:

- Clinical laboratory assessment
    - Haematology
    - Biochemistry –haptoglobin only
    - Coagulation – fibrinogen only
    - Urinalysis
- Pharmacodynamic assessment
    - Immunogenicity sample – MCP-1 sample only

Following completion of the above assessments, the dose of ATL1102 will be administered subcutaneously.

The assessments to be performed *AFTER* administration of ATL1102 are:

- Injection site tolerability assessment
    - Assessments of redness, swelling and pain at the injection site will occur prior to discharge.
    - The participant and their parent/guardian are to be provided with a diary card to take home to capture any site reactions (redness, swelling, pain).
- Review of AEs.

8.2.2.13 Assessment 14: Week 24, Day 165

The dose of IP for each of these weeks is to be administered 3 days PRIOR to the scheduled week's site visit. The following assessments will be performed:

- Physical examination
- Body Weight
- Vital signs
    - Assessment of BP, pulse and temperature
- Clinical laboratory assessment
    - Haematology
    - Biochemistry
    - Coagulation

    - Urinalysis
- Pharmacokinetic assessment
    - A blood sample will be collected for plasma PK assessment
- Pharmacodynamic assessment
    - Lymphocyte Cells sample
    - Complement C3 and Bb sample
    - Immunogenicity sample
    - Exploratory PD sample
- MRI Upper Limb assessment (this assessment may be performed 1 week prior to or after these scheduled site visits).
- Functional Capacity assessment
    - PUL, version 2
    - MyoSet, including the MyoGrip, MyoPinch, MoviPlate
    - EK Scale, version 2
- Respiratory Function assessment
    - SNIP, PEF, CPF, FVC, FEV1, MIP and MEP
- Quality-of-Life assessment
    - The PedsQL will be completed by the participant (or carer, if participant unable to complete)
- Injection site tolerability assessment
    - The participant and their parent/guardian are to be provided with a diary card to take home to capture any site reactions (redness, swelling, pain).
- Review of concomitant medications and AEs.

8.2.3 Follow Up Period – Assessments 15 and 16

Following completion of the active treatment period, two follow up visits will occur. The participant will be required to attend the study site for all follow up visits. If the participant is taking a corticosteroid as part of their concomitant medication regimen, the study site are to contact the participant on the day PRIOR to each follow up visit day to remind the participant NOT to take their steroid dose on the morning of dosing. The participant is to bring the medication with them to the follow up visits. Following the completion of blood sample collection, the participant will be able to take their steroid medication.

8.2.3.1 Assessment 15: Week 28, Day 190

The participant will be required to attend the study site for visit with key safety, PK and efficacy assessments occurring following the completion of the participant's last dose of IP.

The assessments required for Week 28 are also to be performed if the participant withdraws from the study early. If the participant withdraws prior to either Week 12 or between Week 13 and 24, the MRI of the Upper Limbs is also to be performed if possible.

- Physical examination (including assessment of injection site for any remaining reactions)
- Body weight
- Vital signs
    - Assessment of BP, pulse and temperature
- 12-lead ECG (triplicate)
- Clinical laboratory assessments:
    - Haematology

    - Biochemistry
    - Urinalysis
    - Coagulation test
- Pharmacodynamic assessment
    - Lymphocyte Cells sample
    - Complement C3 and Bb sample
    - Immunogenicity sample
    - Exploratory PD sample
- Pharmacokinetic assessment
    - A blood sample will be collected for trough plasma PK assessment
- Respiratory Function assessment
    - SNIP, PEF, CPF, FVC, FEV1, MIP and MEP
- Review of concomitant medications and AEs

8.2.3.2 Assessment 16: Week 32, Day 218

The Week 32 visit is the final study visit. The following assessments will be performed:

- Physical examination (including assessment of injection site for any remaining reactions)
- Vital signs
- Clinical laboratory assessments:
    - Haematology
    - Biochemistry
- Pharmacokinetic assessment
    - A blood sample will be collected for trough plasma PK assessment
- Review of concomitant medications and AEs
- Adverse event assessments

9 STUDY MEASUREMENTS

9.1 Clinical Safety Procedures

The following clinical procedures will be conducted during this study:

- A detailed review of the participant's medical history, including concomitant medication use.
- Height measurement (screening only)
- Body weight measurement
    - Body mass index will be calculated automatically in the CRF
- Physical examination
    - Head, eyes, ears, nose and throat (HEENT), heart, respiratory, abdomen, extremities and neurological assessments will be performed by the Investigator or a qualified delegate
- Vital signs
    - Blood pressure (systolic and diastolic) and heart rate will be taken after the participant has rested for at least 5 minutes
    - Body temperature will be recorded using tympanic measurement

- 12-lead ECG
  - Triplicate measurements will be taken after the participant has rested for at least 5 minutes. Each reading will be taken within 2 to 5 minutes of each other. Measurements of heart rate, PQ (PR), QRS, QT, QTc and ventricular rate (RR) should be recorded. Additional parameters will be calculated as required (e.g., QTcF).
- Local tolerability assessments
  - Assessment of redness, swelling and/or pain at the injection site will be performed. The size of the redness and/or swelling will be captured, and de-identified photographs of injection site reactions may be taken as assessed by the Investigator or delegate.
  - The participant and their parent/guardian are to be provided with a diary card to capture any site reactions (redness, swelling, pain) for up to 48 hours after each dose, and if present at the end of 48 hours, to continue to capture until resolution or end of their study participation.
- Clinical laboratory tests
  - Haematology assessments include the following:
    - Haemoglobin, haematocrit, reticulocytes, red blood cell count, mean corpuscular cell volume, mean corpuscular cell haemoglobin, platelet count, total and differential white blood cell count (neutrophils, lymphocytes, monocytes, eosinophils, basophils).
  - Biochemistry include the following:
    - Sodium, potassium, bicarbonate, phosphate, chloride, calcium, urea, creatinine, creatinine clearance (using the Modified Schwartz formula), haptoglobin, total bilirubin, direct bilirubin, alkaline phosphatase, AST, ALT, GGT, total protein, glucose, albumin, albumin:globulin ratio (A/G ratio), lactate dehydrogenase, CK, CRP.
  - Urinalysis includes the following:
    - Protein, glucose, blood, leukocyte esterase, pH, specific gravity, ketones, urobilinogen, nitrite by dipstick analysis. Note that the dipstick test will be performed weekly, either at the clinic visit or by the participant or parent/guardian at home. The participant, parent/guardian should be advised to contact the Investigator immediately if the dipstick protein rises to 2 or above.
    - The dipstick test must be performed on a sample of the participant's first morning urine. On clinic visit days a sample of the first morning urine should be collected either at the clinic visit or at the participant's home, as required. If collected at the participant's home, the sample should be stored cold in the refrigerator (approximately 2-8°C) until arrival at the clinic visit. The sample should be tested within 3 hours of collection.
    - Microscopy to be performed if abnormalities are observed and deemed necessary by the Investigator / delegate, in particular when blood, protein, nitrite and leukocyte esterase is positive or strong positive.
  - Coagulation test includes the following:
    - Prothrombin time (PT), activated partial thromboplastin time (APTT)

- INR
- Fibrinogen
- Complement C3, C4 and Bb samples

  Complement C3, C4 and Bb samples will be collected pre-dose. The samples will be processed as plasma and serum samples and only C3 analysed. If CRP increases and/or albumin and A/G ratio decrease, or Immunoglobulin G (IgG) has been found to increase, the rest of assessments will be performed based on the safety monitoring guidelines.

- Immunogenicity assessments will include IgG and one or more of the following:
  - Anti-neutrophil cytoplasmic antibody (ANCA)
  - Anti-nuclear antibody (ANA)
  - Anti-drug antibodies (ADA)
  - Circulatory immune complexes (CIC)
  - Monocyte chemotactic protein-1 (MCP1)

  Immunogenicity and MCP-1 samples will be collected pre-dose. Samples will be prepared and analysed for IgG if CRP increases and/or albumin, A/G ratio decreases. If IgG increases, the immunogenicity and MCP-1 samples will be analysed for IgG, MCP-1 and the C3 sample also analysed as per the monitoring rules. If IgG and MCP-1 increase, and C3 decrease the immunogenicity samples are to be analysed as per the monitoring rules.

The timing and frequency of the above procedures are described in the Schedule of Assessments and Sections 8.2.

Safety will be assessed by via the above clinical procedures. Any new or worsening clinically significant abnormalities on these parameters will be reported as an AE, and the participant will be reviewed regularly for AEs. AE assessments will be conducted at every site visit and participants should be instructed to record AEs on the diary card throughout the study. The participant should be asked to report any changes in their health from the prior visit and will be assessed by the appropriate site personnel by questioning and performing the safety procedures required at that visit for expected or unexpected adverse events. The current known safety profile of ATL1102 is detailed in the Investigator Brochure.

9.2 Efficacy Procedures

- Lymphocyte Cells

  A blood sample will be collected to assess, via flow cytometry, leukocytes and lymphocytes including the following cells:

  - CD4+ and CD8+ lymphocytes
  - CD49d (VLA-4 expression) on CD4+ and CD8+ lymphocytes,
    - assessing high expressing CD4+ and CD8+ cells
    - i.e CD4+CD49dhi and CD8+CD49dhi
  - CD45 leucocytes
  - CD3+ T Lymphocytes and CD16+ NK lymphocytes
  - Th1, Th2, and Th17 T helper cells
  - CD19+ B-cells

  - CD14+ monocytes and
  - HLA-DR activated T and B cells and monocytes

- MRI of the Upper Limbs

  Functional muscle structure will be assessed by MRI, using a 3-point Dixon and STIR sequence:

  - 3-point Dixon = fat infiltration and atrophy measurements
  - STIR = oedema measurement, inflammation score

  The MRI scans will be performed at the study site, and the images reviewed and reported by qualified personnel at the study site. MRI assessments are required however, are only to be performed where possible.

- Functional Capacity Assessments:
  - The PUL, version 2 includes 22 items, with an entry item to define the starting functional level. The Investigator or qualified delegate will perform the assessment at the study site. The shoulder level, middle level and distal level scores will be captured along with the total score (calculated by adding the three level scores together, with a maximum global score possible of 74).
  - The Myoset assessments will evaluate strength and fatigability of the upper limb and include MyoPinch, MyoGrip and MoviPlate. This measure will be performed by the Investigator or qualified delegate at the study site.
  - Egen Klassifikation Scale, version 2, is an interview type questionnaire to determine the performance of tasks of daily living with a total score of 51 possible. The Investigator or delegate will oversee the participant or carer complete this questionnaire.
- Quality of Life Assessment via the neuromuscular module of Paediatric Quality of life Inventory tool
- Respiratory Function Assessments:
  - The suite of respiratory function assessments will be performed at the study site by qualified personnel. The assessments include:
  - SNIP
  - PEF and CPF
  - FVC and FEV1
  - MIP and MEP

9.3 Pharmacokinetics and Exploratory PD Samples

Blood samples for plasma PK will be taken on Week 1, Day 1 and Week 16, Day 106, with samples collected pre ATL1102 dose and at 1, 2, 3, 4 and 6 hours post-dose. Further samples will be collected at Week 5/Day 29 (pre-dose), Week 8, Day 53, Week 12/Day 81, Week 24/Day 165, Week 28/Day 190 and Week 32/Day 218 visits.

Blood samples will be collected to allow for investigation of exploratory PD endpoints that may further explore the effect of ATL1102 on key haematology cells, purified mononuclear cell RNA, and for proteomic evaluations.

These samples will be processed on site and either analysed by the sites local laboratory or shipped to a central laboratory for analysis. They will be stored frozen and analysed in batches.

9.4 Blood Volumes for Clinical Laboratory, PK and PD Analysis

A number of blood samples will be collected throughout the study. The assessment, sample volume and the sampling frequency is summarised below (Table 3 and 4).

Table 3: Blood Collection Volume

Assessment	Volume (mL) per sample	Number of Samples	Total Volume
Biochemistry	6.5	10	65.0
haptoglobin/fibrinogen	2.0	6	12.0
Haematology	1.0	18	18.0
Coagulation	2.5	8	22.5
Pharmacokinetic	2.0	18	36.0
Lymphocyte Cells	1.0	6	6.0
Complement C3, C4, Bb			
Immunogenicity	17	8	136
Exploratory PD			
MCP-1	0.5	6	3
**TOTAL**			**298.5 mL**

Table 4: Blood Sampling Collection Volumes Per Visit

Study Day	Assessments	Total Blood Volume Collected (mLs)
Screening	Haematology, Biochemistry, Coagulation	10.0
Day 1	Haematology (x2), Biochemistry, Coagulation Lymphocyte Cells, Complements, Immunogenicity, PK (x6), PD	40.0
Day 15	Haematology, haptoglobin, fibrinogen,MCP-1	3.5
Day 29	Haematology, Biochemistry, Coagulation, Lymphocyte Cells, Complements, Immunogenicity, PK, PD	30.0
Day 43	Haematology, haptoglobin, fibrinogen, MCP-1	3.5
Day 53	Haematology, Biochemistry, Coagulation, Lymphocyte Cells, Complements, Immunogenicity, PK, PD	30.0
Day 64	Haematology, haptoglobin, fibrinogen, MCP-1	3.5
Day 81	Haematology, Biochemistry, Coagulation, Lymphocyte Cells, Complements, Immunogenicity, PK, PD	30.0
Day 92	Haematology, haptoglobin, fibrinogen, MCP-1	3.5
Day 106	Haematology *(x2)*, Biochemistry, Coagulation, Complements, Immunogenicity, PK *(x6)*, PD	40.0
Day 120	Haematology, haptoglobin, fibrinogen, MCP-1	3.5
Day 134	Haematology, Biochemistry, Coagulation, Complements, Immunogenicity, PD	28.0
Day 148	Haematology, haptoglobin, fibrinogen, MCP-1	3.5
Day 165	Haematology, Biochemistry, Coagulation, Lymphocyte Cells, Complements, Immunogenicity, PK, PD	30.0
Day 190	Haematology, Biochemistry, Coagulation, Lymphocyte Cells, Complements, Immunogenicity, PK, PD	30.0
Day 218	Haematology, Biochemistry, PK	9.5
	**TOTAL**	**298.5 mL**

A total of 298.5 mL of blood will be sampled from each participant. The maximum amount withdrawn during a 24-hour period is 40 mL. Based on the estimated circulating blood volume for the youngest

possible participant being approximately 2.5 litres, the collection of 40 mls of blood within a one-day period equates to approximately 2% of the total circulating volume, which remains within the WHO 2011 guidelines on blood sample volume limits of 1-5% of total blood volume collected within a 24 hour period. The overall volume of blood collected throughout the study is also within the WHO guidelines for the amount of 10% total blood volume permitted to be collected over an 8-week period.

The collection, handling and processing of all blood samples will be in accordance with the relevant written procedures outlined in the Study Manual and the details on collection, storage, handling and transportation of specimens.

10 ADVERSE EVENTS

The definitions of AEs and SAEs are given below. It is extremely important that all staff involved in the Study are familiar with the content of this section. The Investigator is responsible for ensuring this.

10.1 Adverse Event Definitions

An AE is defined as any untoward medical occurrence in a participant or clinical investigation participant administered a pharmaceutical product and which does not necessarily have a causal relationship with this treatment. An AE can therefore be any unfavourable and unintended sign (including an abnormal laboratory finding), symptom, or disease temporally associated with the use of a medicinal (investigational) product whether or not related to the medicinal (investigational) product.

Laboratory reference ranges are defined by upper or lower limits of parameters of the respective laboratory. The Investigator should ensure that each parameter out of the normal range is assessed for clinical significance and potential for being an AE. It is at the discretion of the Investigator to document any change in laboratory result as an AE if he considers the change to be clinically significant, even if the absolute value is within the alert limit or reference range.

The participant must be instructed to inform the Investigator about all AEs and these must be documented in the participant records and CRF together with their intensity;

- Severe are those AEs which make normal daily routine impossible.
- Moderate AEs impact the normal daily routine.
- Mild AEs do not impact normal daily routine.

An AE that is assessed as severe should not be confused with a SAE. Severity is a category utilised for rating the intensity of an event, and both AEs and SAEs can be assessed as severe. An event is defined as ‘serious’ when it meets one of the pre-defined outcomes as described in Section 10.2.

The Investigator must assign causality to each adverse event in relation to ATL1102 based on the following scale:

- **Not related:** AE for which there is evidence of another explanation, e.g. the adverse event is obviously explained by the participant’s disease(s), is in accordance with the known effect of a concomitant medication, or has occurred prior to first administration of ATL1102.
- **Unlikely related:** AE with a time to ATL1102 administration that makes a relationship improbable (but not impossible), and disease or other drugs provide plausible explanations.

- **Possibly related:** AE with a reasonable time relationship to ATL1102 administration, but which could also be explained by disease or other drugs. Information on ATL1102 withdrawal may be lacking or unclear.
- **Probably related:** AE with reasonable time relationship to ATL1102 administration that is unlikely to be attributed to disease or other drugs. Response to ATL1102 withdrawal is clinically reasonable. Rechallenge is not required.
- **Definitely related:** AE with plausible time relationship to ATL1102 administration which cannot be explained by disease or other drugs. Response to ATL1102 withdrawal is plausible (pharmacologically, pathologically), and event is definitive pharmacologically or phenomenologically (i.e. an objective and specific medical disorder or a recognised pharmacological phenomenon). Rechallenge, if performed/necessary, is satisfactory.

All AEs must be documented by the Investigator, regardless of causality.

Expected AEs are defined as all AEs stated in the IB. If an AE has not been previously reported (including type, degree, or frequency) in the IB, it is an unexpected adverse event. ATL or designee is responsible for determining the expectedness of an AE.

If an AE leads to premature discontinuation of the study, the appropriate pages of the CRF must be completed.

10.2 Serious Adverse Events

An AE shall be classified as serious if it:

- Results in death.
- Is life-threatening.

  Life threatening in the definition of serious refers to an event in which the participant was at risk of death at the time of the event, it does not refer to an event which hypothetically might have caused death if it were more severe.
- Requires in-patient hospitalisation or prolongation of existing hospitalisation.

  Hospitalisation is defined as in-patient admission or care regardless of duration.

  Out-participant treatment in an emergency room is not in itself an SAE, although the reasons for it may be (e.g. bronchospasm, laryngeal oedema). Elective surgery, hospitalisation for social reasons (with no causing AE), or hospital admissions and/or surgical operations planned before or during this study are not considered AEs if the illness or disease existed before the participant was enrolled in the study, provided that it did not deteriorate in an unexpected way during the study.
- Results in persistent or significant disability/incapacity.
- Is a congenital anomaly/birth defect.
- Is an important medical event.

  This includes events that may not be immediately life-threatening or result in death or hospitalisation but may jeopardise the participant or may require intervention to prevent one of the other outcomes listed above.

10.3 Recording of Adverse Events

AEs will be captured from the time of informed consent until the final study visit (which is 8 weeks after last dose of IP. AEs occurring prior to the first dose of IP will be recorded as pre-treatment AEs. SAEs possibly related to ATL1102 occurring to a study participant after the SAE reporting period will be reported to the Sponsor if the Investigator becomes aware of them.

It is preferable that AEs are reported as diagnoses if one is able to be made, rather than individual signs and symptoms. The AE description, start and stop dates, intensity, causality and outcome must be recorded, as well as any actions taken.

Unless a diagnosis is made, or signs and symptoms are present, laboratory values or vital signs abnormalities should only be reported as AEs if they cause the participant to discontinue from the Study, the investigator feels it is clinically significant, or they meet a criterion for a SAE. Clinically significant laboratory values should be reported to the sponsor within 24 hours of awareness, using the SAE reporting details in Section 10.4.

10.4 Reporting Serious Adverse Events

Investigators and other site personnel must report SAEs on a SAE form to the study safety group using the contact details below within 24 hours of becoming aware of the SAE, regardless of causality.

REFER TO THE SAFETY REPORTING PLAN FOR SERIOUS ADVERSE EVENTS REPORTING CONTACTS AND DETAILS.

Follow-up information on SAEs must also be reported by the investigational site within the same time frame. If a non-serious AE becomes serious, this and other relevant follow-up information must also be provided within 24 hours.

All SAEs will be recorded in the participant records and the CRF. ATL or designee is responsible for informing the regulatory authorities of the SAE as appropriate.

If required, the Investigator must notify their Independent Ethics Committee (IEC) of any SAEs occurring at their site, within the time period specific by the IEC.

In addition to SAEs, non-serious AEs that are assessed as severe must be reported to the sponsor within 24 hours of site awareness. This may be done via telephone or by scanning or faxing a copy of the CRF AE page to the addresses noted above.

10.5 Follow-up of Adverse Events and Serious Adverse Events

All AEs and all SAEs must be followed by the Investigator for at least 4 months after the last dose of study medication and until resolution, until the AE stabilises or is recognised as a permanent condition by the Investigator, or until the participant is lost to follow up, whichever comes first. Follow-up investigations may be necessary according to the Investigator’s medical judgement.

10.6 Pregnancy Reporting

If a participant’s partner becomes pregnant they will be asked to consent to follow up of the pregnancy. Pregnancy of the participant’s partner will be recorded on a pregnancy notification form.

ATL or designee will follow-up on the outcome of the pregnancy and the health status of the neonate, including after study completion.

11 DATA MANAGEMENT

11.1 Data Management and Quality Control

Data collection and entry into the CRF will be completed by authorised study site personnel designated by the Investigator. Appropriate training will be completed with the Investigator and all authorised study site personnel prior to the study being initiated and any data being entered into the CRF for any study participants.

All data must be entered in English. The CRFs should always reflect the latest observations on the participants participating in the Study; therefore, the CRFs are to be completed as soon as possible after the participant's visit. To avoid inter-observer variability, every effort should be made to ensure that the same individual who made the initial Baseline determinations completes all efficacy and safety evaluations. The Investigator must verify that all data entries in the CRFs are accurate and correct. If some assessments are not done, or if certain information is not available or not applicable or unknown, this should be indicated in the CRF. The Investigator (or designee) will be required to sign off on the CRF data.

The study monitor/Clinical Research Associate (CRA) will review the CRFs and evaluate them for completeness and consistency, and compare them to the source documents to ensure that there are no discrepancies between critical data. All entries, corrections and alterations are to be made by the responsible Investigator or designee. The CRA cannot enter data into the CRFs. If corrections are needed, the CRA or Data Manager will query the site.

The CRF is essentially a data entry form and should not constitute the original, or source document, unless otherwise specified. Source documents are all documents used by the Investigator or hospital that relate to the participant's medical history, that verify the existence of the participant, the inclusion and exclusion criteria and all records covering the participant's participation in the study. They include, but are not limited to, laboratory reports, ECG results, pharmacy dispensing records, hospital records, participant files, etc. In this study, the Diary Cards are also considered source documents.

The Investigator is responsible for maintaining source documents. These are to be made available for inspection by the CRA at each monitoring visit.

12 STATISTICAL ANALYSIS

The statistical analysis principles described below will be supplemented by a statistical analysis plan (SAP), which will be finalised before the database is locked. Any changes to the statistical plans will be described and justified in the final report.

12.1 Analysis Sets

The following sets will be used for the statistical analyses:

- **Full analysis set:** All participants in the study.
- **Safety set:** All participants who received any study medication and who have at least one post-dose observation.
- **Intention-to-treat (ITT) set:** All participants who received any study medication and who have reached at least one post efficacy observation.
- **Per-protocol (PP) set:** All participants from the ITT population who essentially completed the study in compliance with the protocol and who reported no major violation of the study protocol.

The final decision to exclude a participant from the per-protocol population will be taken during the study data review meeting.

- **Pharmacokinetic set**: All randomised participants who were administered at least one dose of study medication and have at least one post dose PK result.

12.2 Demographic Characteristics and Baseline Data

Demographic and Baseline variables will be described by statistical characteristics. Categorical data will be described by frequency and percentage, continuous data by mean, standard deviation, minimum, 1st quartile, median, 3rd quartile and maximum. Also the number of missing and non-missing values will be given.

Descriptive analyses will be presented for the ITT. Descriptive analysis for the PP population will be presented if substantially different population from the ITT.

12.3 Efficacy Data

Primary Efficacy Analyses

Quantitative efficacy data, such as,

- Reduction in the number and percentage of lymphocytes at Weeks 5, 8, 12 and 24 compared to Baseline
- Reduction in the number and percentage of CD4+ and CD8+ T cells at Weeks 5, 8, 12 and 24 compared to Baseline
- Reduction in the number of CD4+ CD49dhi T cells and CD8+ CD49dhi T cells at Weeks 5, 8, 12 and 24 compared to Baseline

will be summarised by descriptive statistics (arithmetic mean, standard deviation, standard error of the mean, median, minimum, and maximum) for both cohorts, overall, and over time. Summaries will also be presented for the change from Baseline, when appropriate. Qualitative variables will be summarised by frequency, percentage of participants, and Clopper-Pearson 95% CI overall, and over time.

For key efficacy variables, such as lymphocyte count, the paired t-test will be used to test the change from Baseline to end of treatment. The assumptions of the paired t-test, such as Normality and constant variance, will be assessed visually with residual versus fitted plots, and Normal probability plots. If necessary rectifying transformations will be applied.

There will be no adjustment for multiple comparisons.

There will be no imputations for missing data, so data will be analysed as observed.

Secondary Efficacy Analyses

The secondary functional capacity variables, such as:

- MRI Muscle structure and oedema score
- MyoSet score
- Performance of Upper Limb, and
- Egen Klassifikation score

And respiratory function variables:

- Change in MIP and MEP
- Change in PEF and CPF
- Change in FVC and FEV1, and
- Change in SNIP

And quality-of-life will be assessed by percentage of change in neuromuscular module PedsQL score.

12.4 Safety Data

All participants who have received at least one dose of ATL1102 and who have at least one post-dose observation (Safety data set) will be evaluated for safety. All AEs will be coded using the Medical Dictionary for Regulatory Activities (MedDRA). Incidence of AEs by the severity, relationship to treatment, and outcome will be provided. Laboratory parameters, vital signs and other safety parameters will be listed by participant and presented using descriptive summary statistics (as appropriate).

Additionally, number and percentage values of the following laboratory tests indicative of vascular and renal inflammation (CRP, Creatinine and Creatinine Clearance parameters) and CK, AST, and LDH for muscle injury changes will be analysed.

12.4.1 Extent of exposure

The duration of exposure and number of participants exposed to study treatment will be summarised.

12.4.2 Adverse events

AEs will be coded using the MedDRA and summarised by system organ classes and preferred term.

A summary of the number and percentage of participants with the following AEs will be displayed by:

- All AEs
- Drug-related AEs
- SAEs
- AEs leading to permanent discontinuation of IP.

Treatment emergent AEs (TEAEs) will be defined as AEs with an onset date on or after the date of administration of study drug. If the onset date is missing, the AE will be considered to be treatment emergent.

Treatment-emergent adverse events will be summarised using the latest version of MedDRA by System Organ Class (SOC) and Preferred Term (PT). The incidence and percentage of participants with at least 1 occurrence of a PT will be included, according to the most severe grade using a 3-point scale (mild, moderate, severe). The number of events per PT will also be summarised. Causality (relationship to study treatment) will be summarised separately.

The incidence and frequency of TEAEs, SAEs, related TEAEs, related SAEs, and TEAEs leading to treatment interruption/delay, or discontinuation will be summarised according to SOC and PT. Adverse events and SAEs will also be listed. The duration of TEAEs will be determined and included in listings, along with the action taken and outcome.

Duration of Adverse Events in days will be calculated as the AE stop date minus the AE onset date + 1. Duration will not be calculated for participants who do not have both a full AE onset date and a full AE stop date.

12.4.3 Clinical laboratory evaluations

Summary statistics will be presented by overall for each laboratory value and change from Baseline in each laboratory value at every assessment.

Each laboratory value will be flagged to show whether it is a value within, below, or above the normal range.

Laboratory abnormalities will be reported by the Investigator as AEs if the abnormality is considered clinically significant or if the abnormality results in clinical sequelae. Laboratory results and change from Baseline will be summarised overall, and at each scheduled time point using descriptive statistics. If repeat laboratory tests for the same participant are taken at the same scheduled time point the latest result will be used in the summary tables for that time point. The incidence of laboratory abnormalities will be summarised. Any laboratory results that were analysed but not planned in the protocol will be displayed in the summary tables and listings as unplanned laboratory tests.

12.4.4 Other safety measures

Continuous variables will be summarised along with the change from Baseline at each time point. Other variables will be summarised as appropriate to the data, for example ECG and urinalysis.

12.5 Pharmacokinetic Data

Single and multiple dose PK parameters including concentration-time profile of ATL1102 will be obtained. PK determinations will include the following: AUC, AUC_{inf}, $AUC_{0\text{-}last}$, C_{max}, C_{min}, C_{trough}, T_{max}, and $T_{1/2}$. The PK determinations will be summarised as number of observations, mean, standard deviation, minimum, median, and maximum for each cohort, overall, and over time.

12.6 Vital Signs

Vital Signs (systolic blood pressure, diastolic blood pressure, pulse rate, and body temperature) will be summarised as number of observations, mean, standard deviation, minimum, median, and maximum overall, and by time. If appropriate, vital signs will be reported as change from Baseline and summarised overall, and over time. Vital signs will be listed by participant and time.

12.7 Sample Size Considerations

A key primary efficacy variable is lymphocyte count. A clinically important, and statistically significant reduction in lymphocyte count from baseline to end of treatment will provide some evidence of efficacy of ATL1102 for DMD patients. From the Clinical Study 1102-CT02 (ATL1102 in patients with RRMS), the ATL1102 group baseline mean lymphocyte count was 1.89 ($x10^9$/L) with a standard deviation of 0.428 ($x10^9$/L). For change from baseline to end of treatment the standard deviation of lymphocyte count was 0.477 (10^9/L). A clinically important reduction in lymphocyte count from baseline to end of treatment was judged to be 25%, which equates to a reduction of 0.47 (=0.75x1.89) ($x10^9$/L) in mean lymphocyte count. For the sample size calculation the level of significance was set to 0.05 with a 2-sided paired t-test, mean difference of 0.47 ($x10^9$/L) from baseline to end of treatment, and standard deviation of 0.428 ($x10^9$/L). With these settings a sample size of 9 patients is required to achieve a power of 80%.

Nine evaluable participants are considered sufficient to investigate the safety, tolerability and PK and PD profile of ATL1102 in a rare target patient population.

13 STUDY MANAGEMENT

13.1 Contacts

Details of Investigators, study site, Medical Monitor, contract research organisation (or similar), CRA, ATL and designee contacts can be found in the Study Manual.

13.2 Monitoring

Study monitoring will be performed in accordance with applicable regulations, International Council on Harmonisation (ICH) Good Clinical Practice (GCP), and ATL and designee Standard Operating Procedures (SOPs).

Before the start of the Study, a representative of ATL or designee will contact the investigational site to ensure facilities are adequate and discuss responsibilities with the site staff with regards to following the protocol and regulatory and ethical requirements.

During the Study, a CRA from ATL or its designee will regularly visit the site to monitor and confirm protocol, regulatory and ethical adherence, confirm data accuracy and provide information and support as needed.

The Investigator agrees to allow the CRA direct access to all relevant documents, including electronic medical records, and to allocate his time and the time of his staff to the CRA to discuss findings and any relevant issues.

Site staff will be provided with CRA and back up contact details in the event they have queries or require assistance.

13.3 Audits and Inspections

An audit is a systematic and independent examination of Study related activities and documents to determine whether the evaluated Study related activities were conducted, and the data were recorded, analysed and accurately reported according to the protocol, ATL Standard Operating Procedures and those of ATL designees, GCP, and the applicable regulatory requirements.

Authorised representatives of ATL, its designee, a regulatory authority, or the IEC may visit the centre to perform audits or inspections. The investigator should contact ATL or designee immediately if they are contacted by a regulatory agency about an inspection at their centre. If an audit or inspection occurs, the Investigator and institution agree to allow the auditor/inspector direct access to all relevant documents and allocate their time and the time of their staff to the auditor/inspector to discuss findings and any relevant issues.

13.4 Training of Staff

Each individual involved in conducting a Study should be qualified by education, training, and experience to perform his or her respective tasks.

Site staff may be trained for this study at investigator meetings and initiation visits by ATL, or their designees.

The Investigator will maintain records of all individuals involved in the Study at their site. The Investigator will ensure that appropriate training relevant to the Study is given to all these staff, and that they will receive any new information relevant to the performance of this Study in a timely manner.

13.5 Changes to the Protocol

Study procedures will not be changed without the agreement of ATL.

If it is necessary for the Study protocol to be amended, the amendment or a new version of the Study protocol must be notified to or approved by each site's IEC before implementation at that site, unless the safety of participants is involved. Local requirements must be followed.

If a protocol amendment requires a change to a particular centre's PICF, approval of the revised PICF by ATL or their designee and by the IEC is required before the revised form can be used.

ATL or their designee will distribute amendments and new versions of the protocol to each PI and to the appropriate regulatory authorities as required. The PI will be responsible for submitting to their IEC.

13.6 Study Agreements

The site investigator at each centre must comply with all the terms, conditions and obligations of the Study agreement for this Study. In the event of any inconsistency between this protocol and the Study agreement, the Study agreement shall prevail.

13.7 Ethics Review

The protocol and the PICFs will be submitted for approval to the appropriate IEC, and must be approved or given a favourable opinion in writing as appropriate. In addition, the IEC must approve all advertising used to recruit participants for the Study. The investigator must submit written IEC approval to ATL or designee before they can enrol any participant into the Study.

Any amendment to the protocol will be sent to the IEC. No deviations from or changes to the protocol will be implemented without documented approval/favourable opinion from the IEC of an amendment, except where necessary to eliminate an immediate hazard(s) to Study participant, or when the change(s) involves only logistical or administrative aspects of the Study.

The deviations from or changes to the protocol which were implemented to eliminate an immediate hazard to the Study participant and the proposed amendment, if appropriate, should be submitted to the IEC for review and approval as soon as possible.

The protocol may have to be re-approved by the IEC annually, as local regulations require.

The Investigator must submit progress reports to the IEC according to local regulations and guidelines. The Investigator must also provide the IEC with any reports of SAEs from the Study site in accordance with their IEC's requirements and timelines.

13.8 Ethical Conduct of the Study

The Study will be performed in accordance with the ethical principles in the Guidelines of the World Medical Association's Declaration of Helsinki in its current revised edition, ICH GCP (Committee Proprietary Medicinal Product/ICH/135/95) and applicable regulatory requirements.

13.9 Insurance and Liability

ATL has appropriate liability insurance cover in accordance with all local legal requirements. Further details of this and financial arrangements are specified in the agreements with the Study sites.

13.10 Participant Information and Informed Consent

The Investigator will ensure that the parent/guardian of the participant is given full and adequate oral and written information about the nature, purpose, possible risks and potential benefits of the Study. The participant is to be permitted to be involved in these discussions is appropriate. Parents/guardians of the participant must also be notified that they are free to discontinue from the Study at any time, and they should be given the opportunity to ask questions and should be allowed time to consider the information provided.

The Parent/guardians signed and dated informed consent must be obtained before conducting any procedure specifically for the Study. As appropriate, the participant may sign and date a Participant PICF in addition to the Parent/Guardian PICF.

The Investigator must store the original, signed PICF. A copy of the signed and dated PICF(s) must be given to the parent/guardian of the participant.

13.11 Data Protection

The PICF will explain that Study data will be stored in a computer database, maintaining confidentiality in accordance with national data legislation. Participants in this database will be identified by participant ID number only. The PICF will also explain that for data verification purposes, authorised representatives of ATL, regulatory authorities, IECs or sites may require direct access to parts of the hospital or practice records relevant to the Study, including participant medical history.

13.12 Study Registration

Prior to commencing recruitment, a description of this clinical Study will be available on www.anzctr.org.au. This website will not include information that can identify participants.

13.13 Archiving

The Investigator is responsible for the archiving of the Study records for their site. Study records include the participant files as well as the source data, the Investigator Site File, pharmacy records, and other study documents. To meet sponsor country requirements Study records must be archived for at least 15 years (or at least 2 years since the formal discontinuation of clinical development of the IP).

These documents may need to be retained for a longer period if required by the applicable local regulatory requirements or by an agreement with ATL. It is the responsibility of ATL to inform the PI/institution as to when these documents no longer need to be retained. Records may not be destroyed without prior written consent from ATL.

If the Investigator leaves an investigational site for whatever reason, the responsibility for all study related records must be transferred to another person at site.

13.14 Publication Policy

By signing the study protocol, the Investigator agrees with the use of results of the study for the purposes of national and international registration, publication and information for medical and pharmaceutical professionals. If necessary, the authorities will be notified of the Investigator's name, address, qualifications and extent of involvement.

An Investigator shall not publish any data related to this study (poster, abstract, paper, slide presentation, etc.) without having consulted with ATL in advance. The objectives, the content and the results of the

present clinical Study should be considered confidential. All data and results are the exclusive property of ATL.

Except for legal reasons, the Investigators will not reveal the result of the study to a third party without a mutual agreement about the analysis and interpretation of the data with ATL.

14 REFERENCES

Abboud G, Kaplowitz N. Drug-induced liver injury. *Drug Saf* 2007, 30: 277-94.

Bushby K, Finkel R, Birnkrant DJ, *et al*. Diagnosis and management of Duchenne muscular dystrophy part 1: diagnosis and pharmacological and psychosocial management. *Lancet Neurol* 2010, 9: 77-93.

Common Terminology Criteria for Adverse Events (CTCAE), Version 4.0. Published May 28 2009. US Department of Health and Human Services, National Institute of Health/National Cancer Institute.

Crooke, S.T., Baker, B.F., Kwoh, T.J., *et al*. Integrated safety assessment of 2'-O-Methoxyethyl chimeric antisense oligonucleotides in nonhuman primates and healthy human volunteers. *Molecular Therapy* 2016. 24:1771-82.

Diaz-Manera J, Llauger J, Gallardo E and Illa I. Muscle MRI in muscular dystrophies. *Acta Mylo* 2015; 34(2-3): 95-108.

Guzy S and Bauer. Pharmacometrics in drug development. In: *Statistical Methods in Healthcare,* 2012. Pp 56-77.

Iannetta M, Zingaropoli MA, Bellizzi A, *et al*. Natalizumab Affects T-Cell Phenotype in Multiple Sclerosis: Implications for JCV Reactivation. *PLoS ONE* 2016; 11:1-15.

Koeks Zaida *et al. Clinical outcomes in Duchenne Muscular Dystrophy: A study of 5345 Patients from the TREAT-NMD DMD Global Database. J. Neuromuscular Diseases* 4 (2017(293-306.

Limmroth V, Barkhof F, Desem N, *et al*. CD49d antisense drug ATL1102 reduces disease activity in patients with relapsing-remitting MS. *Neurology* 2014; 83:1-9

Mayhew A, Mazzone ES, Eagle M, *et al*. Development of the Performance of the Upper Limb module for Duchenne muscular dystrophy. DMCN 2013, 55:1038-45.

Merlini L and Sabatelli P. Improving clinical trial design for Duchenne muscular dystrophy. *BMC Neurology* 2015, 15:153.

Pinto-Mariz F, Carvalho LR, Araújo AQC, *et al*. CD49d is a disease progression biomarker and a potential target for immunotherapy in Duchenne muscular dystrophy. *Skeletal Muscle* 2015, 5: 45-55.

Pinto-Mariz F, Carvalho LR, de Mello W, *et al*. Differential integrin expression in T lymphocytes: Potential role in DMD muscle damage. *J Neuroimmunol* 2010, 223: 128-130.

Ricotti V, Evans MR, Sinclair CD, *et al*. Upper Limb Evaluation in Duchenne Muscular Dystrophy: Fat-Water Quantification by MRI, Muscle Force and Function Define Endpoints for Clinical Trials. *PLoS ONE* 2016: 11(9):e0162542.

Rosenberg AS, Puig M, Nagaraju K, *et al*. Immune-mediated pathology in Duchenne muscular dystrophy. *Sci Transl Med* 2015, 7: 299rv4.

Seferian A, Moraux A, Annoussamy M, *et al*. Upper Limb Strength and Function Changes during a One-Year Follow-Up in Non-Ambulant Patients with Duchenne Muscular Dystrophy: An Observational Multicenter Trial. *PLos ONE* 2015:10(2):e0113999.

Servais L, Deconinck N, Moraux A, *et al*. Innovative methods to assess upper limb strength and function in non-ambulant Duchenne patients. *Neuromuscul Disord* 2013, 23: 139-48.

Takeshima Y, Yagi M, Matsuo M, et al Intravenous infusion of an Antisense Oligonucleotide Results in Exon Skipping in Muscle Dystrophin mRNA of Duchenne Muscular Dystrophy. Pediatric Research 2006:59 (5) 690-694

Tubridy N., Behan P.O., Capildeo, R. *et al*. The effect of anti-[alpha]4 integrin antibody on brain lesion activity in MS. *Neurology* 1999. 53:466-472.

WHO – Blood volumes - Bulletin of the World Health Organization 2011; 89:46-53)

Zanotti S, Gibertini S, Di Blasi C, *et al*. Osteopontin is highly expressed in severely dystrophic muscle and seems to play a role in muscle regeneration and fibrosis. *Histopathol* 2011, 59: 1215-28.
