# Supplementary material for: A phase 2 open-label study of the safety and efficacy of weekly dosing of ATL1102 in patients with non-ambulatory Duchenne muscular dystrophy and pharmacology in mdx mice
Source: PLoS One. 2024 Jan 25;19(1):e0294847. doi: 10.1371/journal.pone.0294847 (PMC10810432; doi:10.1371/journal.pone.0294847)
Supplement: S3 File — (PDF) [file pone.0294847.s011.pdf]

McCloud Consulting Group
International Biostatistics & Data Management Specialists

STATISTICAL ANALYSIS PLAN

(Part 1)

**PROTOCOL NUMBER:**	1102-DMD-CT02
**TITLE:**	A Phase 2 open label study to determine the safety, efficacy and pharmacokinetic profile of weekly dosing of ATL1102 in patients with non-ambulatory Duchenne Muscular Dystrophy (DMD).
**STUDY DRUG:**	ATL1102 (CD49d antisense oligonucleotide)
**AUTHOR:**	Peter Button<br>Project Statistician<br>McCloud Consulting Group<br>13/23 Narabang Way<br>Belrose NSW 2095<br>Australia
**SPONSOR:**	Antisense Therapeutics Limited<br>6 Wallace Avenue<br>Toorak Victoria 3142<br>Australia
**DATE DRAFT:**	30th March 2020
**DOCUMENT VERSION:**	Final V1.0

SIGNATURES

STATISTICAL ANALYSIS PLAN (Part 1 V1.0)

TITLE: A Phase 2 open label study to determine the safety, efficacy and pharmacokinetic profile of weekly dosing of ATL1102 in patients with non-ambulatory Duchenne Muscular Dystrophy (DMD).

AUTHORS: MCG PERSONNEL:

**NAME (TITLE):**	**DATE**	**SIGNATURE**
Peter Button<br>(MCG Project Statistician)	30 March 2020	[signature]

REVIEWERS: MCG and SPONSOR PERSONNEL:

**NAME (TITLE):**	**DATE**	**SIGNATURE**
Philip McCloud<br>(MCG Director and Principal Statistician)		

**NAME (TITLE):**	**DATE**	**SIGNATURE**
Nuket Desem<br>(Sponsor Representative)<br>Director, Clinical and Regulatory Affairs<br>Antisense Therapeutics Limited	30 MAR 2020	[signature]

SIGNATURES

STATISTICAL ANALYSIS PLAN (Part 1 V1.0)

TITLE: A Phase 2 open label study to determine the safety, efficacy and pharmacokinetic profile of weekly dosing of ATL1102 in patients with non-ambulatory Duchenne Muscular Dystrophy (DMD).

AUTHORS: MCG PERSONNEL:

NAME (TITLE):	DATE	SIGNATURE
Peter Button<br>(MCG Project Statistician)		

REVIEWERS: MCG and SPONSOR PERSONNEL:

NAME (TITLE):	DATE	SIGNATURE
Philip McCloud<br>(MCG Director and Principal Statistician)	31 MAR 2020	Philip McCloud

NAME (TITLE):	DATE	SIGNATURE
Nuket Desem<br>(Sponsor Representative)<br>Director, Clinical and Regulatory Affairs<br>Antisense Therapeutics Limited		

VERSION HISTORY

Document	Summary of Change	Date of Issue
Draft Version 0.1		15th October 2018
Draft Version 0.2	Minor edits	29th October 2019
Draft Version 0.3	Consistent version number with Part 2	24th November 2019
Draft Version 0.4	Consistent version number with Part 2 (which now includes output templates for MRI data)	19th February 2020
Draft Version 0.5	Consistent version number with Part 2 (which now includes output templates for PedsQL Parent and Teens data)	20th February 2020
Draft Version 0.6	Include Section 6.4.2. to specify convention for handling repeat assessment data, and update Section 10.2 to specify which efficacy analyses will be repeated with the Per-Protocol population.	25th March 2020
Final Version 1.0	Include a clarification in Exploratory Analysis section, a reference to Shift table for reporting Laboratory data, and clarification of 'Related' definitions in the Adverse Event reporting section	30th March 2020

LIST OF ABBREVIATIONS

Abbreviation	Definition
6MWT	6-minute walk test
AE	Adverse Event
ADA	Anti-drug antibodies
ADME	Absorption, distribution, metabolism and excretion
A/G	Albumin/globulin
ALP	Alkaline phosphatase
ALT	Alanine aminotransferase
ANA	Anti-nuclear antibodies
ANCA	anti-neutrophil cytoplasmic antibodies
APTT	Activated Partial Prothrombin Time
ASO	Antisense Oligonucleotide
AST	Aspartate aminotransferase
ATL	Antisense Therapeutics Limited
AUC	Area under (concentration-time) curve to last time-point
AUC_{inf}	Area under (concentration-time) curve to infinity
CIC	circulating immune complexes
CK	Creatinine kinase
C_{max}	Maximum observed plasma concentration
C_{min}	Minimum observed plasma concentration
CRA	Clinical Research Associate
CRF	Case Report Form
CPF	Cough peak flow
CRP	C-reactive protein
DNA	Deoxyribonucleic acid
DMD	Duchenne Muscular Dystrophy
DSMB	Data Safety Monitoring Board
ECG	Electrocardiogram
EK	Egen Klassifikation
FEV1	Forced expiratory volume in 1 second

FVC	Forced vital capacity
GCP	Good Clinical Practice
G-CSF	granulocyte colony stimulating factor
GGT	Gamma glutamyl transferase
IB	Investigator’s Brochure
ICH	International Council for Harmonisation
ID	Identification
IEC	Independent Ethics Committee
IgM	Immunoglobulin M
INR	International Normalised Ratio
IP	Investigational product
ITT	Intent-to-Treat
IUD	Intra-uterine device
IV	Intravenous
LDH	Lactate dehydrogenase
LOAEL	Low observed adverse effect level
MCP1	Monocyte chemotactic protein-1
MedDRA	Medical Dictionary for Regulatory Activities
MEP	Maximum expiratory pressure
MIP	Maximum inspiratory pressure
MRI	Magnetic Resonance Imaging
mRNA	Messenger Ribonucleic Acid
MS	Multiple Sclerosis
NOAEL	No observed adverse effect level
PD	Pharmacodynamic
PedsQL	Paediatric quality-of-life instrument
PEF	Peak expiratory flow
PICF	Patient Information and Consent Form
PK	Pharmacokinetic
PP	Per Protocol
PT	Preferred Term
PUL	Performance of the Upper Limb

RBC	Red blood cell
RNA	Ribonucleic acid
RRMS	Relapsing remitting multiple sclerosis
SAE	Serious Adverse Event
SAP	Statistical Analysis Plan
SC	subcutaneous
SNIP	Sniff nasal inspiratory pressure
SOC	System Organ Class
STIR	Short Tau Inversion Recovery
$T_{1/2}$	Terminal elimination half-life
TEAE	Treatment emergent adverse event
T_{max}	Time to maximal concentration
ULN	Upper Limit of Normal
VLA-4	Very Late Antigen 4
WBC	White blood cell

CONTENTS

Version History 3

List of Abbreviations 4

Contents 7

1. INTRODUCTION 10

2. Study Objectives 10
   - 2.1 Primary Objective 10
   - 2.2 Secondary Objectives 11
   - 2.3 Exploratory Objectives 12

3. Study Design 12
   - 3.1 General Description 12
   - 3.2 Length of Study 13
   - 3.3 Schedule of Events 13
   - 3.4 Treatment Assignment 13
   - 3.5 Determination of Sample Size 13

4. Planned Analyses 14
   - 4.1 Independent Safety Review Committee 14
   - 4.2 Interim Analysis 14
   - 4.3 Final Analysis 14

5. Analysis Populations 14
   - 5.1 Full Analysis Set 14
   - 5.2 ITT Population 14
   - 5.3 Safety Population 14
   - 5.4 Per-Protocol Population 15
   - 5.5 Pharmacokinetic Population 15
   - 5.6 Description of the Populations 15

6. Data Handling Conventions 15

6.1 Treatment Group 15
6.2 Data Presentation 15
6.3 Premature Withdrawal and Missing Data 16
6.4 Common Derivations 16
6.4.1 Baseline 16
6.4.2 Repeat Assessments 16
6.5 Visit Window Conventions 16
6.6 Software 16

7. Statistical Considerations 17
7.1 Statistical Tests 17
7.2 Multiplicity Adjustments 17
7.3 Multi-centre Studies 17
7.4 Other Strata and Covariates 17
7.5 Sub-Group Analyses 17

8. Output Shells 17

9. Study Population 17
9.1 Patient Disposition 17
9.2 Protocol Deviations 17
9.3 Demographics and Baseline Characteristics 18

10. Efficacy Analysis 18
10.1 Primary Efficacy Endpoints 18
10.2 Primary Efficacy Analysis 18
10.3 Secondary Efficacy Endpoints 19
10.4 Secondary Efficacy Analysis 20

11. Safety Analysis 20
11.1 Exposure 20
11.2 Adverse Events (AEs) 20
11.3 Injection Site Tolerability 22
11.4 Clinical Laboratory Evaluations 22

11.4.1 Liver Chemistry Elevations 22
11.4.2 Renal Function Tests 23
11.4.3 Platelet Count 23
11.4.4 C-reactive protein 23
11.4.5 Albumin, A/G Ratio and Complement C3 24
11.5 Pharmacokinetic Data 24
11.6 Vital Signs 24
11.7 12-lead ECG 25
11.8 Physical Examination 25
11.9 Concomitant Medications/Treatments 25
12. References 25

1. INTRODUCTION

This document describes the planned statistical analyses for Protocol 1102-DMD-CT02, a Phase 2 open label study to determine the safety, efficacy and pharmacokinetic profile of weekly dosing of ATL1102 in patients with non-ambulatory Duchenne Muscular Dystrophy (DMD).

The planned statistical analyses of Protocol 1102-DMD-CT02 shall be described in this statistical analysis plan (SAP).

This SAP supplements the study Protocol 1102-DMD-CT02 Final Version 6.0 dated 20th September 2019. If the protocol is amended, this SAP will be revised as required. The plan will be finalized before the study database is locked.

2. STUDY OBJECTIVES

The objectives of this study are to assess the safety, efficacy, and PK profile of weekly dosing of 25mg of ATL1102 in patients with non-ambulatory DMD.

2.1 Primary Objective

The primary objective is to assess the safety and tolerability of 25 mg of ATL1102 administered once weekly in patients with DMD – and this will be done by reporting on the following measures:

- The frequency and intensity of adverse events (AEs)
- The frequency and intensity of redness, swelling and pain at the injection site
- The change in number and percentage values of the following laboratory tests indicative of vascular and renal inflammation
  - C-reactive protein (CRP) parameter
  - Creatinine and Creatinine Clearance parameters
- The changes in platelet numbers and percentage
- Other Clinical laboratory test and assessments
  - Haematology
  - Coagulation
  - Biochemistry
  - Urinalysis

- Vital Signs
- Electrocardiogram (ECG)

2.2 Secondary Objectives

The secondary objectives of the study include the following:

- To investigate the lymphocyte-modulatory potential of ATL1102 in patients with DMD.
- To evaluate the PK profile of ATL1102 in patients with DMD.
- To evaluate the effects of ATL1102 on functional capacity in patients with DMD.
- To evaluate the effects of ATL1102 on respiratory function in patients with DMD.
- To evaluate the effects of ATL1102 on quality-of-life in patients with DMD.

And these objectives will be assessed by reporting the following measures:

For primary efficacy:

- Lymphocyte-modulation potential will be determined by assessing the following haematological parameters by cell surface flow cytometry:
    - Number and percentages of lymphocytes
    - Number and percentages of CD4+ and CD8+ T cells
    - Number of CD4+ CD49dhi and CD8+ CD49dhi T cells

And for secondary efficacy:

- Quantitative MRI measures of Cross Sectional Muscle Area (mm^2), and Fat Fraction (%)
- Muscle Structure Fatty Infiltration (3 point Dixon – Grade 1, 2 and 3)
- Muscle Oedema Score (by STIR responses of Normal Appearance, Mild, Moderate, or Severe Involvement)
- Muscle Structure Atrophy (3 point Dixon: Normal muscle, No atrophy; Minimal to Mild atrophy; Severe atrophy; Completely atrophic)
- MyoSet score as determined by the Myo-Pinch, Myo-Grip, and MoviPlate scores
- Performance of Upper Limb (PUL), version 2
- Egen Klassifikation (EK) Scale, version 2, to evaluate functional ability in non-ambulant patients

Respiratory function will be assessed by:

- Change in maximum inspiratory pressure (MIP) and expiratory pressures (MEP)
- Change in peak expiratory flow (PEF) and cough peak flow (CPF)
- Change in forced vital capacity (FVC) and forced expiratory volume in 1 second (FEV1)

The protocol indicated that the change in sniff nasal inspiratory pressure (SNIP) would be recorded – but this was not done and can therefore not be reported.

Quality-of-life will be assessed by percentage of change in the neuromuscular module of the Paediatric quality of life instrument (PedsQL) score.

And the pharmacokinetic endpoints include the following:

- Concentrations of ATL1102 in plasma (single and multiple dose concentration-time profile) including AUC, AUC_{inf}, $AUC_{0\text{-}last}$, C_{max}, C_{min}, C_{trough}, T_{max}, and $T_{1/2.}$

2.3 Exploratory Objectives

Further exploratory objectives include the evaluation of the PD effects of ATL1102 in patients with DMD – and these include:

- Investigating the inflammatory and muscle injury marker changes:
  - CRP and other serum markers of inflammation
  - CK, AST, and LDH for muscle injury.
- Exploring the effect of ATL1102 on key haematology cells, purified mononuclear cell RNA, and for proteomic evaluations. Where some of these analyses will be performed at a later stage and the results presented in a separate report.

3. STUDY DESIGN

3.1 General Description

This is a single-centre, open label study to assess the safety, efficacy and PK of ATL1102 in non-ambulatory patients with DMD. A weekly ATL1102 dose of 25 mg will be administered subcutaneously for 24 weeks. Nine patients will be enrolled in the study.

A 28-day screening period will be followed by a 24-week active treatment period and an 8-week follow up period. Patients will be enrolled into the study and receive the first dose of investigational product (IP) on Day 1 (Baseline).

Study assessments, which will include evaluations of safety, efficacy and PK parameters, will occur at Screening, Day 1 (Baseline, Week 1), and Weeks 3, 5, 7, 8, 10, 12, 14, 16, 18, 20, 22 and 24. Two follow up visits will occur at Week 28 and Week 32.

3.2 Length of Study

The duration of the study for each patient is up to 36 weeks. This includes a screening period of up to 28 days, followed by 24 weeks of treatment and an 8-week follow up period.

3.3 Schedule of Events

The schedule of assessments can be found in Schedule of Assessments section of the final protocol.

3.4 Treatment Assignment

This is a single arm, open-labelled study and one dosing regimen of 25 mg, once per week for 24 weeks, subcutaneously will be assessed in 9 patients.

3.5 Determination of Sample Size

Sample size estimation is based on the following. A key primary efficacy variable is lymphocyte count. A clinically important, and statistically significant reduction in lymphocyte count from baseline to end of treatment will provide some evidence of efficacy of ATL1102 for DMD patients. Based on results from the clinical study 1102-CT02 (assessing ATL1102 in patients with remitting-relapsing multiple sclerosis), the ATL1102 group baseline mean lymphocyte count was 1.89 (x10^9/L) with a standard deviation of 0.428 (x10^9/L). For change from baseline to end of treatment the standard deviation of lymphocyte count was 0.477 (10^9/L). A clinically important reduction in lymphocyte count from baseline to end of treatment was judged to be 25%, which equates to a reduction of 0.47 (=0.75x1.89) (x10^9/L) in mean lymphocyte count. For the sample size calculation, the level of significance was set to 0.05 with a 2-sided paired t-test, mean difference of 0.47 (x10^9/L) from baseline to end of treatment, and standard deviation of 0.428 (x10^9/L). With these settings a sample size of 9 patients is required to achieve a power of 80%. Nine

evaluable patients are considered sufficient to investigate the safety, tolerability and PK and PD profile of ATL1102 in a rare target patient population.

4. PLANNED ANALYSES

4.1 Independent Safety Review Committee

An independent Data Safety Monitoring Board (DSMB) will be established prior to study start, with an appropriate charter to review safety data at regular intervals throughout the study. Details of the remit of the DSMB and schedule of the proposed meetings is described in the DSMB Charter document.

4.2 Interim Analysis

There are no planned formal interim analyses, however the open label safety and efficacy data were reviewed on an ongoing basis during the conduct of the study.

4.3 Final Analysis

The final analysis will be conducted once all enrolled patients have completed the study and the database lock has occurred.

5. ANALYSIS POPULATIONS

5.1 Full Analysis Set

The Full Analysis Set (FAS) will include all patients who provided informed consent.

5.2 ITT Population

The Intent-to-treat (ITT) population will comprise all patients who received any study medication and who have reached at least one post-dose efficacy observation.

5.3 Safety Population

The Safety (SAF) population will comprise all patients who received any study medication and who have at least one post-dose observation.

5.4 Per-Protocol Population

The Per Protocol (PP) population will comprise all patients from the ITT population who essentially completed the study in compliance with the protocol and who reported no major violation of the study protocol. The final decision to exclude a patient from the per-protocol population will be taken during the study data review meeting, and documented prior to database lock.

5.5 Pharmacokinetic Population

The Pharmacokinetic (PK) population will comprise all enrolled patients who were administered at least one dose of study medication and have at least one post dose PK result.

5.6 Description of the Populations

A summary table with the description of the number of patients in each analysis set, the number of patients who completed the study, and the number of patients who discontinued classified by reason of withdrawal, will be prepared. Corresponding individual listings will be prepared.

Listings with end of study status and visit dates will also be prepared.

6. DATA HANDLING CONVENTIONS

6.1 Treatment Group

All patients will be allocated to the same treatment regimen - weekly ATL1102 dose of 25 mg will be administered subcutaneously for 24 weeks.

The treatment group label on the outputs will be: ATL1102 25 mg.

6.2 Data Presentation

Descriptive analyses will be completed for this study. For continuous parameters, these will include the number of observations, mean, standard deviation, median, minimum and maximum. The minimum and maximum values will be presented to the same number of decimal places as recorded in the database. The other summary statistics will be presented to 1 more decimal place than the raw data. Change from Baseline values will be presented with the above summary statistics plus the 95% CI for the mean change. Categorical

parameters will be summarised as frequency counts and percentages. Percentages will be rounded to 1 decimal place, with the denominator being the number of patients in the relevant population, unless otherwise stated. All confidence intervals reported will be two-sided 95% confidence levels, unless specified otherwise.

The baseline assessment at Day 1 will be used as a reference point when summarising the change from baseline.

In addition, graphs of the individual patient values may be plotted over time for parameters of interest.

6.3 Premature Withdrawal and Missing Data

All observed data for patients who withdraw early will be used for the analysis up to the point of their discontinuation. There will be no imputation for missing values.

6.4 Common Derivations

6.4.1 Baseline

For all parameters presented by visit in the summary tables, baseline will be defined as the last, valid, non-missing assessment prior to the first study drug administration.

6.4.2 Repeat Assessments

For assessments that are repeated (on the same day) the data from the latter (later timepoint) will be used in the summary tables (all data will be included in the listings).

6.5 Visit Window Conventions

Summary tables that present results over time will use the Visit (Weeks) as recorded on the eCRF. Data recorded at Unscheduled visits will be presented in the Listings.

6.6 Software

All analyses will be performed using SAS version 9.4 or later.

7. STATISTICAL CONSIDERATIONS

7.1 Statistical Tests

Formal statistical testing for change from baseline will be conducted for the primary efficacy variables referenced in Section 10.1. Statistical testing of additional variables may be conducted as required.

7.2 Multiplicity Adjustments

There will be no adjustment for multiplicity.

7.3 Multi-centre Studies

Since this is a single-centre there will be no adjustment for centre.

7.4 Other Strata and Covariates

Adjustments for factors or covariates will be undertaken if required.

7.5 Sub-Group Analyses

Subgroup analyses will be undertaken if required

8. OUTPUT SHELLS

Template shells for the tables, listings, and figures are presented in SAP Part II.

9. STUDY POPULATION

9.1 Patient Disposition

The number and percentage of patients who were included in each analysis population, completed the study, and completed each visit will be presented. The reason for early withdrawal will also be summarized and listed.

9.2 Protocol Deviations

A protocol deviation is defined as any intentional or unintentional change to, or noncompliance with, the approved protocol, procedures, or requirements. Deviations may result from the action or inaction of the patients, investigator, or site staff. All deviations will be tracked and should be reported to Institutional Review Boards (IRBs) in accordance with their reporting policy.

Possible protocol deviations will be documented in the source documents and tracked by the Sponsor.

A final list of protocol deviations will be approved by the Sponsor, the Medical Monitor, and others as appropriate, and after database lock provided to McCloud Consulting Group. The final list of protocol deviations will include date of deviation, the deviation, the reason for deviation, the action taken, and comments, and will be included in the final study report.

9.3 Demographics and Baseline Characteristics

Descriptive analyses will be presented for the ITT. Descriptive analysis for the PP population will be presented if substantially different population from the ITT. This information will include the following:

- Demographics including Age, Gender, Weight, Height, BMI, Race, and Hispanic/Latino status
- Medical History (past and current) and DMD History

10. EFFICACY ANALYSIS

10.1 Primary Efficacy Endpoints

The primary efficacy endpoints were the following:

- The reduction in the number and percentage of lymphocytes at Weeks 5, 8, 12 and 24 compared to Baseline,
- The reduction in the number and percentage of CD4+ and CD8+ T cells at Weeks 5, 8, 12 and 24 compared to Baseline, and
- The reduction in the number of CD4+ CD49dhi T cells and CD8+ CD49dhi T cells at Weeks 5, 8, 12 and 24 compared to Baseline.

10.2 Primary Efficacy Analysis

The primary efficacy endpoints will be summarised by descriptive statistics – actual values and change from baseline values by arithmetic mean, standard deviation, standard error of the mean, median, minimum, and maximum, at each of the visits where data was recorded.

For these endpoints the paired t-test will be used to test the change from Baseline to end of treatment (Week 24). The null hypothesis (H_0) is that the change from baseline is zero, the

alternative (H_A) is that the change is not zero. The assumptions of the paired t-test, such as Normality and constant variance, will be assessed visually with residual versus fitted plots, and Normal probability plots. If necessary rectifying transformations will be applied, or a non-parametric test, such as the Wilcoxon sign-rank test, will be used.

There will be no adjustment for multiple comparisons. There will be no imputations for missing data, so data will be analysed as observed.

The primary efficacy analyses described above will be repeated with the Per-Protocol population.

10.3 Secondary Efficacy Endpoints

The secondary efficacy endpoints included the following quantitative measures:

- Quantitative MRI measures of Cross Sectional Muscle Area (mm^2), and Fat Fraction (%)
- MyoSet score as determined by the Myo-Pinch, Myo-Grip, and MoviPlate scores
- Performance of Upper Limb (PUL), version 2
- Egen Klassifikation (EK) Scale, version 2, to evaluate functional ability in non-ambulant participants.

The respiratory function measures included the following:

- Change in maximum inspiratory pressure (MIP) and expiratory pressures (MEP)
- Change in peak expiratory flow (PEF) and cough peak flow (CPF)
- Change in forced vital capacity (FVC) and forced expiratory volume in 1 second (FEV1)

The Quality of life measures included the percentage of change in neuromuscular modules of the Paediatric quality of life instrument (PedsQL) score.

And the following qualitative measures:

- Muscle Structure Fatty Infiltration (3 point Dixon – Grade 1, 2 and 3)
- Muscle Oedema Score (by STIR responses of Normal Appearance, Mild, Moderate, or Severe Involvement)

- Muscle Structure Atrophy (3 point Dixon: Normal muscle, No atrophy; Minimal to Mild atrophy; Severe atrophy; Completely atrophic)

10.4 Secondary Efficacy Analysis

There is no formal statistical testing proposed for the secondary efficacy endpoints, but may be performed as required.

Quantitative measures included in Section 10.3 will be summarised with descriptive statistics for actual values, and change from baseline (and percent change if required), at each visit that the endpoint measure is recorded.

Qualitative measures will be summarised using contingency tables (with number and percent) showing the change in responses at each visit.

11. SAFETY ANALYSIS

All participants who have received at least one dose of ATL1102 and who have at least one post-dose observation (Safety data set) will be evaluated for safety. All AEs will be coded using the Medical Dictionary for Regulatory Activities (MedDRA). Incidence of AEs by the severity, relationship to treatment, and outcome will be provided. Laboratory parameters, vital signs and other safety parameters will be listed by participant and presented using descriptive summary statistics (as appropriate).

Additionally, number and percentage values of the following laboratory tests indicative of vascular and renal inflammation (CRP, Creatinine and Creatinine Clearance parameters) and CK, AST, and LDH for muscle injury changes will be analysed.

11.1 Exposure

The duration of exposure (first to last treatment dates) and number of patients exposed to study treatment will be summarised. A listing will display the date study medication was consumed, medication interruptions, duration of study drug and percentage treatment compliance.

11.2 Adverse Events (AEs)

The AEs will be coded using the MedDRA and summarised by system organ classes and preferred term.

A summary of the number and percentage of participants with the following AEs will be displayed by:

- All AEs
- Drug-related AEs
- SAEs
- AEs leading to permanent discontinuation of IP.

Adverse Events that are classified as being ‘Related’ (to study medication) includes those recorded as ‘Possible’, ‘Probably’, ‘Definitely’, or ‘Unlikely’, and ‘Not Related’ includes those recorded as ‘Not Related’

Treatment emergent AEs (TEAEs) will be defined as AEs with an onset date on or after the date of administration of study drug. If the onset date is missing, the AE will be considered to be treatment emergent.

Treatment-emergent adverse events will be summarised using the latest version of MedDRA by System Organ Class (SOC) and Preferred Term (PT). The incidence and percentage of participants with at least 1 occurrence of a PT will be included, according to the most severe grade using a 3-point scale (mild, moderate, severe). The number of events per PT will also be summarised. Causality (relationship to study treatment) will be summarised separately.

The incidence and frequency of TEAEs, SAEs, related TEAEs, related SAEs, and TEAEs leading to treatment interruption/delay, or discontinuation will be summarised according to SOC and PT. Adverse events and SAEs will also be listed. The duration of TEAEs will be determined and included in listings, along with the action taken and outcome.

Duration of Adverse Events in days will be calculated as the AE stop date minus the AE onset date + 1. Duration will not be calculated for participants who do not have both a full AE onset date and a full AE stop date.

In addition, an overall summary table of AEs will be produced showing the number of patients (and the number of events) with AEs, Related AEs, Moderate/Severe AEs, AEs resulting in Withdrawal, Death.

11.3 Injection Site Tolerability

At 1, 2 and 6 hours after the ATL1102 administration the injection site tolerability symptoms – including assessments of redness, swelling and pain – were recorded. These symptoms including size of redness and swelling areas, and pain score, will be presented in Listings.

11.4 Clinical Laboratory Evaluations

Summary statistics will be presented by overall for each laboratory value and change from Baseline in each laboratory value at every assessment.

Each laboratory value will be flagged to show whether it is a value within, below, or above the normal range, and Shift tables will be produced showing Baseline categories (Low, Normal, High, Not Done) against categories at subsequent visits

Laboratory abnormalities will be reported by the Investigator as AEs if the abnormality is considered clinically significant or if the abnormality results in clinical sequelae.

Laboratory results and change from Baseline will be summarised overall, and at each scheduled time point using descriptive statistics. If repeat laboratory tests for the same participant are taken at the same scheduled time point the latest result will be used in the summary tables for that time point. The incidence of laboratory abnormalities will be summarised. Any laboratory results that were analysed but not planned in the protocol will be displayed in the summary tables and listings as unplanned laboratory tests.

11.4.1 Liver Chemistry Elevations

Liver chemistry elevations which may lead to temporary or permanent halting of study drug administration will be determined for each patient in the study and presented in Listings – specifically a listing showing, for each patient at each assessment where data is available:

- Bilirubin value and grade (Grade 2 : >1.5 – 3.0 x ULN; Grade 3 : >3.0 – 10.0 x ULN; or higher)
- GGT value and grade (Grade 2 : >2.5 – 5.0 x ULN; Grade 3 : GGT >5.0 – 20.0 x ULN; or higher)
- INR value and grade (Grade 2 : >1.5-2.5X ULN; Grade 3 : INR > 2.5 X ULN; or higher)
- ALT value and change from baseline values (specifically identifying any increase by > 150 U/L from Baseline ALT level (Day 1 result))

- CK levels (if available).

11.4.2 Renal Function Tests

Renal function test results which may lead to temporary or permanent halting of study drug administration will be determined for each patient in the study and presented in Listings – specifically a listing showing, for each patient at each assessment where data is available:

- Serum creatinine values and change from baseline values - showing any increase from baseline creatinine values - (specifically identifying serum creatinine increase >= 0.3 mg/dL (26.5 µmol/L) OR >=40% (whichever is greater) above baseline creatinine values)
- Proteinuria dipstick results (specifically identifying dipstick >=2+ (confirmed by dipstick re-measurement and then further confirmed by a quantitative total urine protein measurement of >1.0h/24hr)).

11.4.3 Platelet Count

Platelet count test results which may lead to temporary or permanent halting of study drug administration will be determined for each patient in the study and presented in Listings – specifically a listing showing, for each patient at each assessment where data is available:

- Platelet Count values and change from baseline values specifically identifying any platelet count less than 75 x 10^9/L and/or any platelet measurement < 100 x 10^9/L or a decrease of > 30% from Baseline at any time during the study (treatment or post-treatment period).

11.4.4 C-reactive protein

C-reactive protein test results which may lead to temporary or permanent halting of study drug administration will be determined for each patient in the study and presented in Listings – specifically a listing showing, for each patient at each assessment where data is available:

- CRP values and change from baseline values specifically identifying any change of > 30 mg/L recorded at two consecutive clinic visits (one month apart) which could not be explained by other symptoms or events (e.g. infection, inflammation flare).

11.4.5 Albumin, A/G Ratio and Complement C3

Albumin, A/G ratio and complement C3 test results which may lead to temporary or permanent halting of study drug administration will be determined for each patient in the study and presented in Listings – specifically a listing showing, for each patient at each assessment where data is available:

- Albumin values and change from baseline values (specifically any decrease of 25% from Baseline)
- A/G ratio values and change from baseline values (specifically any decrease of 25% from Baseline)
- Complement C3 values and change from baseline values (specifically any decrease of 20% from Baseline)
- IgG values and change from baseline values (specifically any increase of 30% from Baseline).

Note: A/G Ratio is calculated as:

A/G Ratio = (Albumin Level)/(Total Protein – Albumin Level).

11.5 Pharmacokinetic Data

Single and multiple dose PK parameters including concentration-time profile of ATL1102 will be obtained. PK determinations will include the following: AUC, AUC_{inf}, $AUC_{0\text{-}last}$, C_{max}, C_{min}, C_{trough}, T_{max}, and $T_{1/2}$. The PK determinations will be summarised as number of observations, mean, standard deviation, minimum, median, and maximum for each cohort, overall, and over time.

11.6 Vital Signs

Vital Signs (systolic blood pressure, diastolic blood pressure, pulse rate, and body temperature) will be summarised as number of observations, mean, standard deviation, minimum, median, and maximum overall, and by time. If appropriate, vital signs will be reported as change from Baseline and summarised overall, and over time. Vital signs will be listed by patient and time. In addition, Vital Sign assessments recorded as Normal, Abnormal NCS, and Abnormal CS will be presented at each visit.

11.7 12-lead ECG

Triplicate 12-lead ECG measurements were taken after the participant has rested for at least 5 minutes. These measurements, including heart rate, PQ (PR), QRS, QT, QTc and ventricular rate (RR), were taken and recoded within 2 to 5 minutes of each other. Additional parameters were calculated as required (e.g., QTcF). Where three ECG measurements are recorded at an assessment, then the average of those three measurements will be used as the measure at that visit in the summary table. The observed ECG results, and change from Baseline, will be summarized at each scheduled visit and listed.

In addition, ECG assessments recorded as Normal, Abnormal NCS, and Abnormal CS will be presented at each visit.

11.8 Physical Examination

The results of the Physical examination (including those for head, eyes, ears, nose and throat (HEENT), heart, respiratory, abdomen, extremities, and neurological) at the visits where this data was recorded, will be presented in Listings.

11.9 Concomitant Medications/Treatments

Concomitant medications will by coded using the WHO drug dictionary (latest release). The start and stop dates will be used to identify when a concomitant medication was taken during the study and the details included in listings.

12. REFERENCES

1. SAS/STAT Software. 9.4 ed. Cary, NC: SAS Institute
