# Supplementary material for: A phase 2 open-label study of the safety and efficacy of weekly dosing of ATL1102 in patients with non-ambulatory Duchenne muscular dystrophy and pharmacology in mdx mice
Source: PLoS One. 2024 Jan 25;19(1):e0294847. doi: 10.1371/journal.pone.0294847 (PMC10810432; doi:10.1371/journal.pone.0294847)
Supplement: S4 File — (PDF) [file pone.0294847.s012.pdf]

STATISTICAL ANALYSIS PLAN

(Part 2)

PROTOCOL NUMBER: 1102-DMD-CT02

TITLE: A Phase 2 open label study to determine the safety, efficacy and pharmacokinetic profile of weekly dosing of ATL1102 in patients with non-ambulatory Duchenne Muscular Dystrophy (DMD).

STUDY DRUG: ATL1102 (CD49d antisense oligonucleotide)

AUTHOR:
Peter Button
Project Statistician
McCloud Consulting Group
13/23 Narabang Way
Belrose NSW 2095
Australia

SPONSOR:
Antisense Therapeutics Limited
6 Wallace Avenue
Toorak Victoria 3142
Australia

DATE DRAFT: 30th March 2020

DOCUMENT VERSION: Final V1.0

SIGNATURES

STATISTICAL ANALYSIS PLAN (Part 2 V1.0)

**TITLE:**	A Phase 2 open label study to determine the safety, efficacy and pharmacokinetic profile of weekly dosing of ATL1102 in patients with non-ambulatory Duchenne Muscular Dystrophy (DMD).

AUTHORS: MCG PERSONNEL:

**NAME (TITLE):**	**DATE**	**SIGNATURE**
Peter Button<br>(MCG Project Statistician)	31 March 2020	

REVIEWERS: MCG and SPONSOR PERSONNEL:

**NAME (TITLE):**	**DATE**	**SIGNATURE**
Philip McCloud<br>(MCG Director and Principal Statistician)	31MAR2020	Philip McCloud

**NAME (TITLE):**	**DATE**	**SIGNATURE**
Nuket Desem<br>(Sponsor Representative)<br>Director, Clinical and Regulatory Affairs<br>Antisense Therapeutics Limited	31 March 2020	

VERSION HISTORY

Document	Summary of Change	Date of Issue
Draft Version 0.1		15th October 2018
Draft Version 0.2	Update VAD Specs	29th October 2019
Draft Version 0.3	Include Output Templates in separate document	24th November 2019
Draft Version 0.4	Update to include MRI data Tables and Listings	19th February 2020
Draft Version 0.5	Update to include output templates for PedsQL Parent and Teens data	20th February 2020
Draft Version 0.6	Include Section 6.4.2. to specify convention for handling repeat assessment data, and update Section 10.2 to specify which efficacy analyses will be repeated with the Per-Protocol population.	27th February 2020
Final V1.0	Consistent with Part 1 V1.0 date	30th March 2020

LIST OF ABBREVIATIONS

Abbreviation	Definition
6MWT	6-minute walk test
AE	Adverse Event
ADA	Anti-drug antibodies
ADME	Absorption, distribution, metabolism and excretion
A/G	Albumin/globulin
ALP	Alkaline phosphatase
ALT	Alanine aminotransferase
ANA	Anti-nuclear antibodies
ANCA	anti-neutrophil cytoplasmic antibodies
APTT	Activated Partial Prothrombin Time
ASO	Antisense Oligonucleotide
AST	Aspartate aminotransferase
ATL	Antisense Therapeutics Limited
AUC	Area under (concentration-time) curve to last time-point
AUC_{inf}	Area under (concentration-time) curve to infinity
CIC	circulating immune complexes
CK	Creatinine kinase
C_{max}	Maximum observed plasma concentration
C_{min}	Minimum observed plasma concentration
CRA	Clinical Research Associate
CRF	Case Report Form
CPF	Cough peak flow
CRP	C-reactive protein
DNA	Deoxyribonucleic acid
DMD	Duchenne Muscular Dystrophy
DSMB	Data Safety Monitoring Board
ECG	Electrocardiogram
EK	Egen Klassifikation
FEV1	Forced expiratory volume in 1 second

FVC	Forced vital capacity
GCP	Good Clinical Practice
G-CSF	granulocyte colony stimulating factor
GGT	Gamma glutamyl transferase
IB	Investigator’s Brochure
ICH	International Council for Harmonisation
ID	Identification
IEC	Independent Ethics Committee
IgM	Immunoglobulin M
INR	International Normalised Ratio
IP	Investigational product
ITT	Intent-to-Treat
IUD	Intra-uterine device
IV	Intravenous
LDH	Lactate dehydrogenase
LOAEL	Low observed adverse effect level
MCP1	Monocyte chemotactic protein-1
MedDRA	Medical Dictionary for Regulatory Activities
MEP	Maximum expiratory pressure
MIP	Maximum inspiratory pressure
MRI	Magnetic Resonance Imaging
mRNA	Messenger Ribonucleic Acid
MS	Multiple Sclerosis
NOAEL	No observed adverse effect level
PD	Pharmacodynamic
PedsQL	Pediatric quality-of-life instrument
PEF	Peak expiratory flow
PICF	Patient Information and Consent Form
PK	Pharmacokinetic
PP	Per Protocol
PT	Preferred Term
PUL	Performance of the Upper Limb

RBC	Red blood cell
RNA	Ribonucleic acid
RRMS	Relapsing remitting multiple sclerosis
SAE	Serious Adverse Event
SAP	Statistical Analysis Plan
SC	subcutaneous
SNIP	Sniff nasal inspiratory pressure
SOC	System Organ Class
STIR	Short Tau Inversion Recovery
$T_{1/2}$	Terminal elimination half-life
TEAE	Treatment emergent adverse event
T_{max}	Time to maximal concentration
ULN	Upper Limit of Normal
VLA-4	Very Late Antigen 4
WBC	White blood cell

CONTENTS

Version History 3

List of Abbreviations 4

Contents 7

1. INTRODUCTION 8

2. StANDARD HEADER AND FOOTER 8

3. PROGRAMMING NOTES 8

4. Value added datasets specifications 9

5. References 11

6. List of outputs 11

7. Output Templates 15

1. INTRODUCTION

This document describes the data displays to be produced planned statistical analyses for the safety, efficacy and pharmacokinetic endpoints for the Protocol 1102-DMD-CT02. This includes underlying populations and templates. The templates will provide an overview and guidance of available information and layout schemes, but do not necessarily need to be an exact match with outputs produced.

This SAP supplements the study Protocol 1102-DMD-CT02 Final Version 6.0 dated 20th September 2019. If the protocol is amended, this SAP will be revised as required. The plan will be finalized before the study database is locked.

2. STANDARD HEADER AND FOOTER

The following standard header and footer will be included on all tables and listings:

<u>Header</u>
<Table, Listing and Figure Title>
Antisense Therapeutics Limited
Protocol: 1102-DMD-CT02
<Subtitle, if needed>
Population: <   >

<u>Footer</u>
Program: < filename of the program> Report: < filename of the report> Run Date: <Date of production DDMMMYYYY>
Footnote requirements to explain specifics for the output

3. PROGRAMMING NOTES

Notes for programmers' will be stated as "Notes" below the table templates or written directly within a table as annotations. Notes for programming should not necessarily appear in any outputs.

4. VALUE ADDED DATASETS SPECIFICATIONS

Laboratory Data VAD

Laboratory Ranges dataset [has Age specific normal ranges – need Age from Demo]

Notes:

- Set lower/upper limits for CRP to 0, 7.99
- If value (lborresc) is <5 set value to 4.99
- If value (lborresc) is <10 set value to 9.99
- Determine AG Ratio as AG_Ratio=(albumin/(total_protein-albumin));
- Determine H, L flags after merging ranges

FACS Tube VADs

Approach used to determine FACS parameters:

Using the lymphocyte counts from the Haematology lab results (FBC) calculate the cell numbers (as 10^9/L) utilising the cell subset percentages determined by the FACS analysis as these cell subset percentages will be accurate. This applies to the T lymphocytes (CD3+) and the subsets of T lymphocytes (CD4+, CD8+), B lymphocytes (CD19+), the NK Cells and the Th1, Th2 and Th17 cells. The same is also applicable for the Monocytes. where the monocyte counts from the FBC can be used to calculated the cell numbers for the cell subsets including the M1 cell subsets based on the cell subset percentages determined by the FACS analysis.

MYOSET Data VAD

Include Grip, Pinch and Moviplate Total Scores – and for each Score the Dominant/Non-Dominant Side and the Right/Left Hand Side

And need to determine Percent Predicted Values for Grip and Pinch Scores:

For Average (Left and Right Hand), Right Hand and Left Hand

Percent Predicted Pinch

If Age < 17 then

- Percent Predicted = (Pinch Score x 100)/(1.956 x (Exp(0.088 x Age)))
- Percent Predicted =(Grip Score x 100)/(3.3996 x (Exp(0.1527 x Age)))

If Age ≥ 17 then

- Percent Predicted = (Pinch Score x 100)/(8.206 x (Exp(-0.001 x Age)))
- Percent Predicted =(Grip Score x 100)/(45.662 x (Exp(-0.001 x Age)))

Age should be based on patient's Age at a particular visit (so formula used for a patient may change during the study (if they Age < 17 to Age ≥ 17

PUL2.0 Measure VAD

Three Dimension and one Total Score

High Level Shoulder Dimension Score: Sum responses to Items 1-6
Mid Level Elbow Dimension Score: Sum responses to Items 7-15
Distal Wrist & Hand Dimension Score: Sum responses to Items 16-22
Total Score: Sum responses to Items 1-22

PedsQL VAD for Teens and Parent

Description of the Duchenne Muscular Dystrophy Module:

Dimensions	Number of Items	Cluster of Items	Reversed Scoring	Direction of Dimensions
Daily Activities	5	1-5	1-5	Higher scores indicate lower problems
Treatment	4	1-4	1-4	
Worry	6	1-6	1-6	
Communication	3	1-3	1-3	

Scoring of Dimensions:

Item Scaling	5-point Likert scale from 0 (Never) to 4 (Almost always)
Weighting of Items	No
Extension of the Scoring Scale	Scores are transformed on a scale from 0 to 100.

Scoring Procedure	**<u>Step 1: Transform Score</u>**<br><br>Items are reversed scored and linearly transformed to a 0-100 scale as follows:<br><br>0=100, 1=75, 2=50, 3=35, 4=0<br><br>**<u>Step 2: Calculate Scores by Dimensions</u>**<br><br>If more than 50% of the items in the scale are missing, the scale scores should not be computed<br><br>Mean score= Sum of the items over the number of items answered
Interpretation and Analysis of Missing Data	No Total Score:<br><br>If more than 50% of the items in the scale are missing, the Scale Scores should not be computed.<br><br>If 50% or more items are completed: Impute the mean of the completed items in a scale.

5. REFERENCES

1. SAS/STAT Software. 9.4 ed. Cary, NC: SAS Institute

6. LIST OF OUTPUTS

<u>Listings</u>

Listing L1.1	Subject Visit Days
Listing L1.2	Subject Demographics
Listing L1.3	Protocol Deviations
Listing L1.4	Subject Study Populations
Listing L1.5	Subject Comments
Listing L2.1.1	Subject Duchenne Muscular Dystrophy History
Listing L2.1.2	Subject Duchenne Muscular Dystrophy History - Durations
Listing L2.2	Subject Medical and Surgical History
Listing L3.1	Subject Prior Duchenne Muscular Dystrophy Medication
Listing L3.2	Subject Prior Duchenne Muscular Dystrophy Medication - Durations

Listing L4	Subject Prior Non-DMD Medication
Listing L5	Subject Concomitant Medication
Listing L6	Subject Disposition Completion Withdrawal
Listing L7	Subject Treatment Administration
Listing L8	Injection Site Tolerability
Listing L9	Vital Signs
Listing L10	ECG
Listing L11	Adverse Events
Listing L12	Serious Adverse Events

Listing L13.1.1-5	Biochemistry
Listing L13.1.6	Biochemistry
Listing L13.2.1-2	Coagulation
Listing L13.2.3	Coagulation
Listing L13.3.1-3	Haematology
Listing L13.3.4	Haematology
Listing L13.4.1-2	Urinalysis
Listing L13.4.3	Urinalysis

Listing 14.1	Liver Chemistry Elevations Stopping Rule
Listing 14.2	Renal Function Test Result Stopping Rule
Listing 14.3	Platelet Count Result Stopping Rule and C-reactive Protein Stopping Rule
Listing 14.4	Albumin and A_G ratio Complement C3 Test Result Stopping Rule

Listing L15.1	FACS Tube 1 Data
Listing L15.2	FACS Tube 2 Data
Listing L15.3	FACS Tube 3 Data

Listing 16.1	Listing of MRI Muscle Structure Fatty Infiltration (3 point Dixon)
Listing 16.2	Listing of MRI Muscle Oedema Score (by STIR)
Listing 16.3	Listing of MRI Muscle Structure Atrophy (3 point Dixon)
Listing 16.4	Listing of MRI Cross Sectional Muscle Area (mm2)
Listing 16.5	Listing of MRI Fat Fraction (%)

Listing L17.1-3	Respiratory Function Assessment (SNIP, PEF, PEF(%Pred), CPF, FVC, FVC(%Pred), FEV1, FEV1(%Pred) MIP, MEP
Listing L17.4	Respiratory Function Assessment

[Listing 21 in output templates]

Listing L18	Egen Klassification Scale Version (EK2)

[Listing 22 in output templates]

Listing 19.1 MyoSet – MyoGrip
Listing 19.2 MyoSet – MyoPinch
[Listing 23 in output templates]
Listing 19.3 MyoSet – MoviePlate

Listing L20.1.1 MyoSet - Combined Dominant
Listing L20.1.2 MyoSet - Combined Non-Dominant
Listing L20.2.1 MyoSet - Combined - Percent Predicted Average
Listing L20.2.2 MyoSet - Combined - Percent Predicted Dominant
Listing L20.2.3 MyoSet - Combined - Percent Predicted Non-Dominant
[Listing 24 in output templates]

Listing L21 PedsQL Parent Report
[Listing 25 in output templates]

Listing L22 PedsQL Teen Report
[Listing 26 in output templates]

Listing L23 Functional Capacity Assessment – PUL 2.0 (PUL for DMD) Performance of the Upper Limb Module (PUL) – Responses to questions related to Dimensions for High Level Shoulder, Mid-Level Elbow, Distal wrist and hand
[Listing 27 in output templates]

Listing 24 Pharmacodynamic Assessment Immunogenicity
Listing 25 Pharmacodynamic Blood Sample

Figures

Figure 1.1-1.4 PUL 2.0 (PUL for DMD)
Figure 2 - EK2
Figure 3 - PedsQL Parent
Figure 4 - PedsQL Teen
Figure 5 - Lymphocytes
Figure 5_2 Eosinophils
Figure 6.1-6.6 MyoSet
Figure 6.7 MyoSet – Percent Predicted
Figure 7.1-7.11 FACS Tube 1
Figure 8.1-8.6 FACS Tube 2
Figure 9.1a/b-9.6a/b Respiratory
Figure 10.1-10.10 FACS Tube 1 – group means
Figure 10.2.1-10.2.10 FACS Tube 1 – change group means
Figure 11.1.1-11.1.6 FACS Tube 2 – group means

Figure 11.2.1-11.2.6 FACS Tube 2 – change group means

Tables

Table T1 Demographics
Table T2.1-4 Vital Signs
Table T2.5 Vital Signs Assessment
Table T3.1 Adverse Events
Table T3.2 Overall Summary of Adverse Events
Table T4 Treatment Exposure
Table T5.1 Biochemistry
Table T5.2 Coagulation
Table T5.3 Haematology
Table T6.1 Biochemistry (Shift Tables)
Table T6.2 Coagulation (Shift Tables)
Table T6.3 Haematology (Shift Tables)
Table T7.1-7.4 PUL2.0 Total Score (Overall and By Entry Score)
Table T8 Egen Klassification Scale Version 2(EK2)
Table T9 Respiratory Measures
Table T10 MYOSET Measures

Table T11.1.1.1 FACS Tube 1 Change (FAS)
Table T11.1.1.1.1FACS Tube 1 Change PValues (FAS)
Table T11.1.1.1.2FACS Tube 1 Change PValues ExtraWk24_28 (FAS)

Table T11.1.1.2 FACS Tube 1 Percent Change (FAS)
Table T11.1.1.2.1FACS Tube 1 Percent Change PValues (FAS)
Table T11.1.1.2.2FACS Tube 1 Percent Change PValues ExtraWk24_28 (FAS)

Tables T11.1.1 Repeated for Per Protocol Population

Table T11.1.2.1 FACS Tube 1 Change (PP)
Table T11.1.2.1.1FACS Tube 1 Change PValues (PP)
Table T11.1.2.1.2FACS Tube 1 Change PValues ExtraWk24_28 (PP)

Table T11.1.2.2 FACS Tube 1 Percent Change (PP)
Table T11.1.2.2.1FACS Tube 1 Percent Change PValues (PP)
Table T11.1.2.2.2FACS Tube 1 Percent Change PValues ExtraWk24_28 (PP)

Table T11.2.1 FACS Tube 2 Change
Table T11.2.2 FACS Tube 2 Percent Change
Table T11.3.1 FACS Tube 3 Change
Table T11.3.2 FACS Tube 3 Percent Change

Table T12.1-5	ECG Data
Table T12.6	ECG Data Assessment

Table 13.1.1-.3	Summary of MRI Muscle Structure Fatty Infiltration (3 point Dixon) – Central Reading (and Distal, Proximal Readings)
Table 13.2.1-.3	Summary of MRI Muscle Oedema Score (by STIR) – Central Reading (and Distal, Proximal Readings)
Table 13.3.1-.3	Summary of MRI Muscle Structure Atrophy (3 point Dixon) – Central Reading (and Distal, Proximal Readings
Table 13.4.1-.3	Summary of MRI Cross Sectional Muscle Area (mm2) – Central Reading (and Distal, Proximal Readings
Table 13.5.1-.3	Summary of MRI Fat Fraction (%) – Central Reading (and Distal, Proximal Readings

[Table 16 in output templates]

Table 14	Summary of PedsQL Parent Report
Table 15	Summary of PedsQL Teen Report

7. OUTPUT TEMPLATES

In Document SAP Part 2 V1.0 LTF Templates
