# Supplementary material for: A phase 2 open-label study of the safety and efficacy of weekly dosing of ATL1102 in patients with non-ambulatory Duchenne muscular dystrophy and pharmacology in mdx mice
Source: PLoS One. 2024 Jan 25;19(1):e0294847. doi: 10.1371/journal.pone.0294847 (PMC10810432; doi:10.1371/journal.pone.0294847)
Supplement: Attachment — Submitted filename: Response to Reviewers June 2023.docx [file pone.0294847.s013.docx]

Thursday, 29 June 2023

To the Editors, PLOS One Journal

Dear Editors,

Thank you for considering the revision of the below manuscript for consideration of publication in PLOS One Journal. The revised manuscript is attached and the authors’ response to the reviewers comments are below.

**PONE-D-21-29128: “A Phase 2 open-label study to determine the safety and efficacy of weekly dosing of ATL1102 in patients with non-ambulatory Duchenne muscular dystrophy”**

1) Before we can proceed, please report in the Methods section the date range in which participants were recruited for research purposes.

***The manuscript has been updated as requested to include: “Participants were recruited from a single site in Melbourne, Australia with the study running from August 2018 until January 2020.”***

2) Please submit the CONSORT flow diagram of the study design as the manuscript's ""Fig 1"".

***CONSORT flow diagram has been included and referenced in the manuscript as “Fig 1”.***

Journal Requirements:

When submitting your revision, we need you to address these additional requirements.

1. Please ensure that your manuscript meets PLOS ONE's style requirements, including those for file naming.

***The authors have checked the formatting against the guidelines and have made changes accordingly.***

2. You indicated that you had ethical approval for your study. In your Methods section, please ensure you have also stated whether you obtained consent from parents or guardians of the minors included in the study or whether the research ethics committee or IRB specifically waived the need for their consent.

***Consent was obtained from parents/guardians. Manuscript updated.***

3. Thank you for stating the following in the Competing Interests:

“This study was sponsored by Antisense Therapeutics Ltd. Authors GT and ND are paid employees of Antisense Therapeutics Ltd.

The other authors have declared that no competing interests exist.”

We note that one or more of the authors have an affiliation to the commercial funders of this research study: Antisense Therapeutics Ltd

a. Please provide an amended Funding Statement declaring this commercial affiliation, as well as a statement regarding the Role of Funders in your study. If the funding organization did not play a role in the study design, data collection and analysis, decision to publish, or preparation of the manuscript and only provided financial support in the form of authors' salaries and/or research materials, please review your statements relating to the author contributions, and ensure you have specifically and accurately indicated the role(s) that these authors had in your study. You can update author roles in the Author Contributions section of the online submission form.

Please also include the following statement within your amended Funding Statement.

“The funder provided support in the form of salaries for authors [Dr Tachas and Ms Desem], but did not have any additional role in the study design, data collection and analysis, decision to publish, or preparation of the manuscript. The specific roles of these authors are articulated in the ‘author contributions’ section.”

If your commercial affiliation did play a role in your study, please state and explain this role within your updated Funding Statement.

b. Please also provide an updated Competing Interests Statement declaring this commercial affiliation along with any other relevant declarations relating to employment, consultancy, patents, products in development, or marketed products, etc.

Within your Competing Interests Statement, please confirm that this commercial affiliation does not alter your adherence to all PLOS ONE policies on sharing data and materials by including the following statement: "This does not alter our adherence to  PLOS ONE policies on sharing data and materials.” (as detailed online in our guide for authors <http://journals.plos.org/plosone/s/competing-interests>). If this adherence statement is not accurate and there are restrictions on sharing of data and/or materials, please state these. Please note that we cannot proceed with consideration of your article until this information has been declared.

Please include both an updated Funding Statement and Competing Interests Statement in your cover letter. We will change the online submission form on your behalf.

***Funding Statement:***

***The ATL1102 in DMD clinical trial was funded in its entirety by the sponsor Antisense Therapeutics Ltd. Authors Ms Desem and Dr Tachas are employees of the sponsor and so received payment for services from the sponsor as employees. Ms. Desem and Dr Tachas hold an equity interest in the sponsor. Dr Tachas and Ms Desem along with other sponsor employees and sub-contracted specialists were involved in the study design and data analysis. Authors Dr Woodcock and Dr Ryan were at the time of the trial employees of the Royal Children’s Hospital and Murdoch Children’s Research Institute and are not affiliated with the sponsor in any way and have not received any direct personal payment or honoraria from the sponsors, nor do they or their family members hold a financial interest or stock in the sponsor company. Dr Woodcock is still an employee of the above institutions, but Dr Ryan has since left the employment to take up public office. Dr Woodcock and Dr Ryan were involved in the trial design as unpaid consultants. As this was a clinical trial, publication was always planned from trial inception. No employees of the sponsor were involved in the data collection, although Ms Desem did liaise closely with the MCRI/RCH site staff and Clinical Trial Organisation throughout the trial.***

***Author Dr Button was paid for services as the study statistician.***

***None of the other authors received any payment from the sponsor to conduct this study. All other authors had input into writing or revising this manuscript.***

***Competing Interests Statement:***

***The authors confirm that the involvement of employees of the sponsor Antisense Therapeutics Ltd in the trial design, data analysis and decision to publish this data does not alter our adherence to PLOS ONE policies on sharing data and materials.***

4. We note that you have indicated that data from this study are available upon request. PLOS only allows data to be available upon request if there are legal or ethical restrictions on sharing data publicly. For information on unacceptable data access restrictions, please see <http://journals.plos.org/plosone/s/data-availability#loc-unacceptable-data-access-restrictions>.

In your revised cover letter, please address the following prompts:

a) If there are ethical or legal restrictions on sharing a de-identified data set, please explain them in detail (e.g., data contain potentially identifying or sensitive patient information) and who has imposed them (e.g., an ethics committee). Please also provide contact information for a data access committee, ethics committee, or other institutional body to which data requests may be sent.

***No legal restrictions exist to prevent full dissemination of the data. Ethically, as this deals with patients who have a rare disease, single patient level data could be used to inadvertently identify subjects. As such, it is not ethical to publish freely and completely such data. The Royal Children’s Hospital HREC, MCRI and the sponsor Antisense Therapeutics Ltd would be willing to share such data to appropriately qualified researchers but the authors cannot make it freely available to all.***

***Requests for complete data sets should be sent to the corresponding author*** [***ian.woodcock@rch.org.au***](mailto:ian.woodcock@rch.org.au)***. The HREC can be contacted by email*** [***rch.ethics@rch.org.au***](mailto:rch.ethics@rch.org.au)***.***

b) If there are no restrictions, please upload the minimal anonymized data set necessary to replicate your study findings as either Supporting Information files or to a stable, public repository and provide us with the relevant URLs, DOIs, or accession numbers. Please see <http://www.bmj.com/content/340/bmj.c181.long> for guidelines on how to de-identify and prepare clinical data for publication. For a list of acceptable repositories, please see <http://journals.plos.org/plosone/s/data-availability#loc-recommended-repositories>.

We will update your Data Availability statement on your behalf to reflect the information you provide

5. Please include a separate caption for each figure in your manuscript.

***Manuscript changed to include an Appendix with figure captions included.***

6. Please include captions for your Supporting Information files at the end of your manuscript, and update any in-text citations to match accordingly. Please see our Supporting Information guidelines for more information: <http://journals.plos.org/plosone/s/supporting-information>.

***Manuscript changed to include an Appendix with figure captions included.***

[Note: HTML markup is below. Please do not edit.]

Reviewers' comments:

Reviewer's Responses to Questions

**Comments to the Author**

1. Is the manuscript technically sound, and do the data support the conclusions?

The manuscript must describe a technically sound piece of scientific research with data that supports the conclusions. Experiments must have been conducted rigorously, with appropriate controls, replication, and sample sizes. The conclusions must be drawn appropriately based on the data presented.

Reviewer #1: Yes

Reviewer #2: Partly

2. Has the statistical analysis been performed appropriately and rigorously?

Reviewer #1: No

Reviewer #2: I Don't Know

3. Have the authors made all data underlying the findings in their manuscript fully available?

The [PLOS Data policy](http://www.plosone.org/static/policies.action#sharing) requires authors to make all data underlying the findings described in their manuscript fully available without restriction, with rare exception (please refer to the Data Availability Statement in the manuscript PDF file). The data should be provided as part of the manuscript or its supporting information, or deposited to a public repository. For example, in addition to summary statistics, the data points behind means, medians and variance measures should be available. If there are restrictions on publicly sharing data—e.g. participant privacy or use of data from a third party—those must be specified.

Reviewer #1: Yes

Reviewer #2: Yes

4. Is the manuscript presented in an intelligible fashion and written in standard English?

PLOS ONE does not copyedit accepted manuscripts, so the language in submitted articles must be clear, correct, and unambiguous. Any typographical or grammatical errors should be corrected at revision, so please note any specific errors here.

Reviewer #1: Yes

Reviewer #2: Yes

**5. Review Comments to the Author**

Please use the space provided to explain your answers to the questions above. You may also include additional comments for the author, including concerns about dual publication, research ethics, or publication ethics. (Please upload your review as an attachment if it exceeds 20,000 characters)

Reviewer #1: Here are some comments:
The sample size calculation is for lymphocyte modulation which according to the protocol is a key secondary efficacy outcome. Since the primary endpoint was safety, I would like you to add something along what is written in the protocol to the methods after the sample size calculation “Nine evaluable participants are considered sufficient to investigate the safety, tolerability and PK and PD profile of ATL1102 in a rare target patient population.”

***Manuscript updated accordingly.***

Results: Lymphocyte count references to table 3 for 8,12, 24w results but table 3 only reports baseline, 24w and 28w.

***Table 3 changed to include results for weeks 8 and 12.***

***Table 3 week 8 and week 12 lymphocyte and T lymphocyte data can now be read together with baseline end of dosing week 24 data, and the week 28, four-week post- dosing data. This data supports the statements on line 260 that there was a consistent trend toward declines in the mean number of lymphocytes, and CD49d+ T lymphocytes measured 3 days post-dose at week 8, 12 and 24. The Lymphocyte and T lymphocytes were measured during treatment 3 days after the last dose on these 3 occasions starting at week 8 based upon data from a previous study of ATL1102 in patients with Relapsing-Remitting Multiple Sclerosis (RRMS) analyzing blood was indicated appropriate (lines 190 to 192).***

The protocol and the statistical analysis plan only mention comparisons baseline with time points up to week 24. Nothing is written about w24 vs w28. Why is focus on w24 vs w28?

***The SAP part 1 page 13 mentions study assessment evaluations of various parameters at screening, baseline, various weeks to week 24 and the follow up visit Week 28. We analyzed comparisons baseline with time points to week 24 (all 3 day post dose data now in table 3) and over time to week 28. The week 28 lymphocyte data is four weeks after the end of the dosing week 24 sample data. Based on the previous study of ATL1102 in patients with RRMS, 3 days after the last dose at week 8 treatment effects at week 12, four weeks past the last dose had returned to normal range. This supported the rationale for assessing w24 to w28 data in the DMD study. With subjects in the trial on ATL1102 treatment for 24 weeks in sync when only 4 weeks apart from week 28, a trend rebound of lymphocytes was observed at week 28 compared to week 24 ((p= 0.051 paired T test) and mean number of CD3+CD49d+T lymphocytes (CD4+CD49d+ and CD8+CD49d+ T lymphocytes) at week 24 is statistically significantly lower vs week 28 (p=0.030 paired T test)***

Why did the authors choose to use paired t-tests?

***The sample size statement in the protocol was based on the t-test being used to analyze the data. A paired t-test was used as we were interested in the difference in lymphocyte numbers for the same subject at two times and estimated with a normal distribution based on our calculations, we had 80% powering to see lymphocyte reduction of 0.47 (=0.75x1.89) (x109/L) in mean lymphocyte count based on the RRMS data. However, because the sample size was small, and we could not necessarily establish that the data were normally distributed, we also used the non-parametric Wilcoxon Sign–Rank test on the same paired observations and in all cases the results were consistent with those from the T-test.***

Overall test of time trend seems more appropriate with post hoc comparisons (i.e. contrasts) between time points (General least squares model or mixed effects model adjusting for the basline value i.e. timeXvalue = baslineValue + time).

***Repeated measures analysis of lymphocyte, CD3+ CD49d + T cells and CD3-CD49d+ NK lymphocytes was run in a post hoc analysis comparing baseline to a linear combination of values measured three days post-dose at weeks 8, 12 and 24. We only saw the NK lymphocyte numbers decreased significantly as stated. Repeated measures analysis is not mentioned in the statistical analysis plan and we have now captured this as a Post Hoc statistical method.***

I would suggest more figures. Why not show lymphocyte results as spaghetti-plots per white blood cell type (or at least for the lymphocytes)? (i.e. one line per patient over baseline, 8w,12w, 24w and 28w results)

***The below figure has been added.***

***“The Table 3 lymphocyte baseline, week 8, 12 and 24 week data 3 days post each dose and the week 28 data, four week past end of dosing data is shown as a bee swarm plot with mean expression values of lymphocytes.”***

“Repeated measures analysis of CD3-CD49d+ NK lymphocytes in a post hoc analysis comparing baseline to a linear combination of values measured three days post-dose at weeks 8, 12 and 24 was significantly lower compared to baseline (p=0.018), with comparable NK lymphocyte numbers at week 28.” Here repeated measures analysis is written but this is not mentioned in the statistical analysis plan nor the methods. (This is also written in the discussion.)

***This analysis was performed post-hoc and was not part of the original analysis plan. The manuscript has been updated accordingly.***

The method for correlation is not mentioned under the statistical analysis section.

***Associations between variables was tested using a Pearson correlation test. The manuscript has been updated to state this.***

Please report the p-value for the correlation estimates.

***As this was a Pearson correlation the p-value has little relevance given the small sample size. However, the p-value was <0.0001. We have not included this in the manuscript.***

Figure S2 and S3 should be labelled as scatter plots not “correlation”, also, the blue line needs to be described (linear regression line?)
***We have now relabelled supplementary Figures S2 and S3.***

***Figure S2: Scatter Plot showing the association between the Moviplate scores and the PUL 2.0 distal dimension scores over the 24 Week ATL1102 treatment period.***

**Figure S3: Scatter plot showing the association of the Grip Strength scores and the MRI data of the forearm lean muscle area (non-fat) over the 24-week ATL1102 treatment period: The MRI is the determined lean muscle mass compartment across the mid (central) dominant forearm. The linear regression line is also plotted.**

Regarding the mdx analyses: Nothing is written about the methods of these analyses under the statistical analysis section.

***The statistical analysis section has now been updated to include the preclinical (mouse) analyses used.***

Mdx analyses: Why adjust for multiple comparisons in one analysis (Tukey) and not in the next (Fishers LSD test)?

***This has now been corrected – both analyses have been adjusted for multiple comparisons (Tukey)***

Mdx analyses: Please report effect estimates with estimate, 95% confidence interval and p-value
***All data is now represented in the figures as mean with 95% confidence interval and associated p-value, when statistical significance is reached (P<0.05).***

Mdx analyses: Please report overall Anova p-value before reporting between group comparisons.

***ANOVA summary p-values have been included in the text (line 218 and 226). The below tables show the details for this section.***

***CD49d mRNA (Figure B)***

| ***ANOVA summary*** |  |
| --- | --- |
| ***F*** | ***4.912*** |
| ***P value*** | ***0.0072*** |
| ***P value summary*** | ******** |
| ***Significant diff. among means (P < 0.05)?*** | ***Yes*** |
| ***R squared*** | ***0.3448*** |

***Eccentric muscle damage (Figure E and F)***

| ***ANOVA summary*** |  |
| --- | --- |
| ***F*** | ***20.61*** |
| ***P value*** | ***<0.0001*** |
| ***P value summary*** | ********** |
| ***Significant diff. among means (P < 0.05)?*** | ***Yes*** |
| ***R squared*** | ***0.6789*** |

Regarding limitations, it is important to write in the discussion that due to the large number of statistical tests there is a high risk of spurious significant results at the 5% significance level.

***Whilst we agree conducting more tests can increase the probability of finding an effect by chance the authors feel that the fairly consistent data trends due not suggest results that appear by chance. There is a consistency of lymphocyte and subset reductions as now shown in table 3 and the few statistical tests looking at the difference at two times: during and after treatment. Moreover, there was consistency of results in the multiple functional, strength and MRI parameters, and good correlations across these assessments.***

Reviewer #2: Page 14, lines 322-332. But according to the results, the PUL2.0 score remained stable with no significant change between baseline and week twenty-four.

***The authors feel that the manuscript does not state that the findings were significant, merely that there was a positive change in PUL compared to published natural history data. By clearly displaying the confidence intervals we can allow the reader to make a judgement on the reliability of the results.***

Page 15, line 327. This study is not appropriate to support the findings of the study.

***The previously published data from the Pane et al study suggested that over a twelve-month period a mean decrease in PUL2.0 score of 2.17 can occur, in older age patients who it should be noted have less starting PUL2.0 function to lose, compared to those in the phase II trial. We agree that this study is not comparable to our population and so we have moved the sentence comparing the Phase II Trial cohort with the Pane cohort to make it clearer it is not directly relevant to support the findings of the study. We have not completely removed the reference however, as we feel it is illustrative of the natural history decline in patients with DMD who are no longer ambulant. Whilst there is a lack of published data to compare our dataset to, we need to use any previously published data available as a cautious comparator.***

Page 15, line 331. It should be Table 4 instead of Table 5.

***Manuscript altered.***

Page 16, line 363. This statement contradicts the results; no significant change was apparent between baseline and week twenty-four for the mean Mercuri semi-quantitative fatty infiltration score.

***The authors have provided the corresponding comparison in the dorsal muscle group across the central slice which comprise more than 50% of the cross-sectional area which suggests a trend towards decrease in forearm fat fraction over the six months. The stated confidence intervals in the dorsal vs volar muscles in table 6 allow the reader to draw their own conclusions as to the importance of this result.***

6. PLOS authors have the option to publish the peer review history of their article ([what does this mean?](https://journals.plos.org/plosone/s/editorial-and-peer-review-process#loc-peer-review-history)). If published, this will include your full peer review and any attached files.


If you choose “no”, your identity will remain anonymous but your review may still be made public.


**Do you want your identity to be public for this peer review?** For information about this choice, including consent withdrawal, please see our [Privacy Policy](https://www.plos.org/privacy-policy).

Reviewer #1: No

Reviewer #2: **Yes:** MARICELA RODRIGUEZ

 Thank you for considering our re-submission.

Yours faithfully,

**Dr Ian R Woodcock MBBS MSc MRCPCH(UK) FRACP**

**Paediatric Neurologist, The Royal Children's Hospital, Melbourne**

**PhD Student, The University of Melbourne**

**Honorary Fellow, The Murdoch Childrens Research Institute, Melbourne**

**Melbourne Children’s Campus
50 Flemington Road, Parkville, Victoria, 3052
T: 03 9345 5661**

**E:** [**Ian.Woodcock@rch.org.au**](about:blank)
