# Supplementary material for: A phase 2 open-label study of the safety and efficacy of weekly dosing of ATL1102 in patients with non-ambulatory Duchenne muscular dystrophy and pharmacology in mdx mice
Source: PLoS One. 2024 Jan 25;19(1):e0294847. doi: 10.1371/journal.pone.0294847 (PMC10810432; doi:10.1371/journal.pone.0294847)
Supplement: Attachment — Submitted filename: Response to Reviewers October 2023.docx [file pone.0294847.s014.docx]

Thursday, 5 October 2023

From: Dr Ian R Woodcock

Paediatric Neurologist

The Royal Children’s Hospital and The Murdoch Children’s Research Institute

50 Flemington Road, Parkville, VIC 3052

To: The Editors

PLOS One

Dear Editors,

Thank you for considering the revision of the below manuscript for consideration of publication in PLOS One Journal. The revised manuscript is attached and the authors’ response to the reviewers’ comments are below.

**PLOS ONE**

**PONE-D-23-13519**

**“A Phase 2 open-label study of the safety and efficacy of weekly dosing of ATL1102 in patients with non-ambulatory Duchenne muscular dystrophy”**

**In response to reviewer 1 comments, title to be changed to: ““A Phase 2 open-label study of the safety and efficacy of weekly dosing of ATL1102 in patients with non-ambulatory Duchenne muscular dystrophy and pharmacology in mdx mice”**

**Journal Requirements**

When submitting your revision, we need you to address these additional requirements.

1. Please ensure that your manuscript meets PLOS ONE's style requirements, including those for file naming. The PLOS ONE style templates can be found at

[https://journals.plos.org/plosone/s/file?id=wjVg/PLOSOne_formatting_sample_main_body.pdf](https://urldefense.com/v3/__https:/journals.plos.org/plosone/s/file?id=wjVg*PLOSOne_formatting_sample_main_body.pdf__;Lw!!Jgbw!G3H3Z-2kwqIpE4XNB0ptiJw4WseJsz65z6clK7O3V0UdJ8c0usOqjsBtfBW1MWbePZ8aTEdhwy6S-9hzUuQ0$) and

[https://journals.plos.org/plosone/s/file?id=ba62/PLOSOne_formatting_sample_title_authors_affiliations.pdf](https://urldefense.com/v3/__https:/journals.plos.org/plosone/s/file?id=ba62*PLOSOne_formatting_sample_title_authors_affiliations.pdf__;Lw!!Jgbw!G3H3Z-2kwqIpE4XNB0ptiJw4WseJsz65z6clK7O3V0UdJ8c0usOqjsBtfBW1MWbePZ8aTEdhwy6S-7xc3ZXk$)

***All style requirements checked and changed where required to meet with journal style requirements.***

2. Thank you for stating the following in the Competing Interests/Financial Disclosure * (delete as necessary) section:

"The ATL1102 in DMD clinical trial was funded in its entirety by the sponsor Antisense Therapeutics Ltd. Authors Ms Desem and Dr Tachas are employees of the sponsor and so received payment for services from the sponsor as employees. Ms. Desem and Dr Tachas hold an equity interest in the sponsor. Dr Tachas and Ms Desem along with other sponsor employees and sub-contracted specialists were involved in the study design and data analysis. Authors Dr Woodcock and Dr Ryan were at the time of the trial employees of the Royal Children’s Hospital and Murdoch Children’s Research Institute and are not affiliated with the sponsor in any way and have not received any direct personal payment or honoraria from the sponsors, nor do they or their family members hold a financial interest or stock in the sponsor company. Dr Woodcock is still an employee of the above institutions, but Dr Ryan has since left the employment to take up public office. Dr Woodcock and Dr Ryan were involved in the trial design as unpaid consultants. As this was a clinical trial, publication was always planned from trial inception. No employees of the sponsor were involved in the data collection, although Ms Desem did liaise closely with the MCRI/RCH site staff and Clinical Trial Organisation throughout the trial.

Author Dr Button was paid for services as the study statistician.

None of the other authors received any payment from the sponsor to conduct this study. All other authors had input into writing or revising this manuscript.

We note that you received funding from a commercial source: "

Antisense Therapeutics Ltd"

Please provide an amended Competing Interests Statement that explicitly states this commercial funder, along with any other relevant declarations relating to employment, consultancy, patents, products in development, marketed products, etc.

Within this Competing Interests Statement, please confirm that this does not alter your adherence to all PLOS ONE policies on sharing data and materials by including the following statement: "This does not alter our adherence to PLOS ONE policies on sharing data and materials.” (as detailed online in our guide for authors [http://journals.plos.org/plosone/s/competing-interests).](https://urldefense.com/v3/__http:/journals.plos.org/plosone/s/competing-interests).__;!!Jgbw!G3H3Z-2kwqIpE4XNB0ptiJw4WseJsz65z6clK7O3V0UdJ8c0usOqjsBtfBW1MWbePZ8aTEdhwy6S-1eh6Foy$)  If there are restrictions on sharing of data and/or materials, please state these. Please note that we cannot proceed with consideration of your article until this information has been declared.

Please include your amended Competing Interests Statement within your cover letter. We will change the online submission form on your behalf.

***This information has been amended as below:***

***Competing Interests/Financial Disclosure Statement:***

***The ATL1102 in DMD clinical trial was funded in its entirety by the commercial sponsor Antisense Therapeutics Ltd. Antisense Therapeutics Ltd is a publicly traded company, listed on the Australian ASX.***

***At the time of the trial, authors Ms Desem and Dr Tachas were employees of the sponsor and so received payment for services from the sponsor as employees. Ms. Desem and Dr Tachas hold an equity interest in the sponsor. Dr Tachas and Ms Desem along with other sponsor employees and sub-contracted specialists were involved in the study design and data analysis. Ms Desem has since left the company and no longer is employed by the sponsor.***

***Authors Dr Woodcock and Dr Ryan were at the time of the trial employees of the Royal Children’s Hospital and Murdoch Children’s Research Institute and are not affiliated with the sponsor in any way and have not received any direct personal payment or honoraria from the sponsors, nor do they or their family members hold a financial interest or stock in the sponsor company. Dr Woodcock is still an employee of the above institutions, but Dr Ryan has since left the employment to take up public office as a Member of the Australian Parliament. Dr Woodcock and Dr Ryan were involved in the trial design as unpaid consultants.***

***Dr Woodcock has received honoraria for work performed including educational activities and attendance at advisory board meetings from pharmaceutical companies Biogen, Novartis, Roche and Avidity and an educational travel bursary to attend an international conference in 2016 from Biogen. Dr Woodcock has received grants for research work from FSHD Global Research Foundation, FSHD Society and Fulcrum Therapeutics. Dr Ryan has been principal investigator on a number of industry-sponsored clinical trials. None of these disclosures affected the work Dr Woodcock performed on this clinical trial.***

***Dr Ryan has received honoraria for work performed including educational activities and attendance at advisory board meetings from pharmaceutical companies Biogen, Novartis, Roche. Dr Ryan has been principal investigator on a number of industry-sponsored clinical trials. None of these disclosures affected the work Dr Ryan performed on this clinical trial.***

***Dr Yiu has received advisory board honoraria from Biogen and Roche, and has received research support from Biogen, Roche, Pfizer and PTC therapeutics unrelated to the content of this manuscript. Dr Yiu has been principal investigator on a number of industry-sponsored clinical trials. None of these disclosures affected the work Dr Yiu performed on this clinical trial.***

***Prof. Delatycki has received grant awards from NHMRC and is principal investigator in industry sponsored clinical trials including trials sponsored by Rearta and PTC.***

***As this was a clinical trial, publication was always planned from trial inception. No employees of the sponsor were involved in the data collection, although Ms Desem did liaise closely with the MCRI/RCH site staff and Clinical Trial Organisation throughout the trial.***

***As the study statistician, author Dr Button was paid a consultancy fee for his services from the trial sponsor commercial company Antisense Therapeutics Ltd.***

***Authors Dr Houweling, Dr Coles and Dr Tiong were recipients of a grant to perform the MDX studies. This grant was paid by the sponsor Antisense Therapeutics Ltd.***

***None of the other authors received any payment from the sponsor to conduct this study. All other authors had input into writing or revising this manuscript.***

***The authors confirm that the involvement of employees of the sponsor Antisense Therapeutics Ltd in the trial design, data analysis and decision to publish this data does not alter our adherence to PLOS ONE policies on sharing data and materials.***

3. Thank you for stating the following in the Competing Interests section:

"The authors confirm that the involvement of employees of the sponsor Antisense Therapeutics Ltd in the trial design, data analysis and decision to publish this data does not alter our adherence to PLOS ONE policies on sharing data and materials."

Please confirm that this does not alter your adherence to all PLOS ONE policies on sharing data and materials, by including the following statement: "This does not alter our adherence to  PLOS ONE policies on sharing data and materials.” (as detailed online in our guide for authors [http://journals.plos.org/plosone/s/competing-interests](https://urldefense.com/v3/__http:/journals.plos.org/plosone/s/competing-interests__;!!Jgbw!G3H3Z-2kwqIpE4XNB0ptiJw4WseJsz65z6clK7O3V0UdJ8c0usOqjsBtfBW1MWbePZ8aTEdhwy6S-6VubjEl$)).  If there are restrictions on sharing of data and/or materials, please state these. Please note that we cannot proceed with consideration of your article until this information has been declared.

Please include your updated Competing Interests statement in your cover letter; we will change the online submission form on your behalf.

***Competing Interests Statement:***

***The authors confirm that the involvement of employees of the sponsor Antisense Therapeutics Ltd in the trial design, data analysis and decision to publish this data does not alter our adherence to PLOS ONE policies on sharing data and materials.***

4. We note that the original protocol that you have uploaded as a Supporting Information file contains an institutional logo. As this logo is likely copyrighted, we ask that you please remove it from this file and upload an updated version upon resubmission.

***This will be amended and logo removed from the protocol attached to this re-submission.***

5. In your Data Availability statement, you have not specified where the minimal data set underlying the results described in your manuscript can be found. PLOS defines a study's minimal data set as the underlying data used to reach the conclusions drawn in the manuscript and any additional data required to replicate the reported study findings in their entirety. All PLOS journals require that the minimal data set be made fully available. For more information about our data policy, please see [http://journals.plos.org/plosone/s/data-availability](https://urldefense.com/v3/__http:/journals.plos.org/plosone/s/data-availability__;!!Jgbw!G3H3Z-2kwqIpE4XNB0ptiJw4WseJsz65z6clK7O3V0UdJ8c0usOqjsBtfBW1MWbePZ8aTEdhwy6S-8VdtQsC$).

Upon re-submitting your revised manuscript, please upload your study’s minimal underlying data set as either Supporting Information files or to a stable, public repository and include the relevant URLs, DOIs, or accession numbers within your revised cover letter. For a list of acceptable repositories, please see [http://journals.plos.org/plosone/s/data-availability#loc-recommended-repositories](https://urldefense.com/v3/__http:/journals.plos.org/plosone/s/data-availability*loc-recommended-repositories__;Iw!!Jgbw!G3H3Z-2kwqIpE4XNB0ptiJw4WseJsz65z6clK7O3V0UdJ8c0usOqjsBtfBW1MWbePZ8aTEdhwy6S---O4hyH$). Any potentially identifying patient information must be fully anonymized.

Important: If there are ethical or legal restrictions to sharing your data publicly, please explain these restrictions in detail. Please see our guidelines for more information on what we consider unacceptable restrictions to publicly sharing data: [http://journals.plos.org/plosone/s/data-availability#loc-unacceptable-data-access-restrictions](https://urldefense.com/v3/__http:/journals.plos.org/plosone/s/data-availability*loc-unacceptable-data-access-restrictions__;Iw!!Jgbw!G3H3Z-2kwqIpE4XNB0ptiJw4WseJsz65z6clK7O3V0UdJ8c0usOqjsBtfBW1MWbePZ8aTEdhwy6S-4Ft2J57$).  Note that it is not acceptable for the authors to be the sole named individuals responsible for ensuring data access.

We will update your Data Availability statement to reflect the information you provide in your cover letter.

***This has been submitted as supporting information***

6. Please review your reference list to ensure that it is complete and correct. If you have cited papers that have been retracted, please include the rationale for doing so in the manuscript text, or remove these references and replace them with relevant current references. Any changes to the reference list should be mentioned in the rebuttal letter that accompanies your revised manuscript. If you need to cite a retracted article, indicate the article’s retracted status in the References list and also include a citation and full reference for the retraction notice.

***References checked and correct***


**Reviewers' comments:**

Reviewer's Responses to Questions

**Comments to the Author**

1. Is the manuscript technically sound, and do the data support the conclusions?

The manuscript must describe a technically sound piece of scientific research with data that supports the conclusions. Experiments must have been conducted rigorously, with appropriate controls, replication, and sample sizes. The conclusions must be drawn appropriately based on the data presented.

Reviewer #1: Yes

Reviewer #2: Yes

2. Has the statistical analysis been performed appropriately and rigorously?

Reviewer #1: I Don't Know

Reviewer #2: Yes

3. Have the authors made all data underlying the findings in their manuscript fully available?

Reviewer #1: Yes

Reviewer #2: Yes

4. Is the manuscript presented in an intelligible fashion and written in standard English?
Reviewer #1: Yes

Reviewer #2: Yes

5. Review Comments to the Author

Reviewer #1: Thank you for this interesting paper. I do accept this paper, although following the title I found it confusing to discover that two studies were presented where one included mice. Given the study design and small cohort I would highlight this 2 front study in the title.

***In response to reviewer 1 comments, title to be changed to: “A Phase 2 open-label study of the safety and efficacy of weekly dosing of ATL1102 in patients with non-ambulatory Duchenne muscular dystrophy and pharmacology in mdx mice”***

Reviewer #2: Important note: This review pertains only to ‘statistical aspects’ of the study and so ‘clinical aspects’ [like medical importance, relevance of the study, ‘clinical significance and implication(s)’ of the whole study, etc.] are to be evaluated [should be assessed] separately/independently. Further please note that any ‘statistical review’ is generally done under the assumption that (such) study specific methodological [as well as execution] issues are perfectly taken care of by the investigator(s). This review is not an exception to that and so does not cover clinical aspects {however, seldom comments are made only if those issues are intimately / scientifically related & intermingle with ‘statistical aspects’ of the study}. Agreed that ‘statistical methods’ are used as just tools here, however, they are vital part of methodology [and so should be given due importance]. I look at the manuscript in/with statistical view point, other reviewer(s) look(s) at it with different angle so that in totality the review is very comprehensive. However, there should be efforts from authors side to improve (may be by taking clues from reviewer’s comments). Therefore, please do not limit the revision only (with respect) to comments made here.

COMMENTS: Although this manuscript is well drafted [and the study is excellent with respect to most of the aspects], I have few very minor observations/concerns (different opinion) which are given below:

In section ‘Pre-clinical studies in mice’ (lines 112) it is stated that “Male mdx (n = 48) and age matched C57Bl10/J wild-type (WT, n = 12) controls” which I have not understood (clearly). According to my information, sample size of matched samples generally is same. If it is not a standard ‘pair matching’, what type of matching it is? If it is 4 x 1 [4cases, 1control] matching {which is very much valid}, why not make that clear? I think, even for ‘Pre-clinical studies’ the methodology should be rigorously followed.

***There were a total of 48 mdx mice and 12 controls purchased from JAX laboratories and these were then split into 4 groups of n = 12 mdx.***

***Wording in the manuscript clarified.***

Since Consort (line 138) is a short form of the term {group of words} [‘CONsolidated Standard Of Reporting Trials (CONSORT)], it should be represented in ‘CAPITAL’ letters, in my opinion.

***Manuscript amended***

Is the statement made in lines 196-7 “A sample size of 9 participants was calculated as required to achieve a power of 80%” is correct? Consideration of “power” in Phase-II trials is questionable / rarely seen, in my experience. Most of the known formulas and sample size estimation software are available for Phase-III trials, in my knowledge. Which software or formula is used need to be mentioned.

***The manuscript has been amended to include the software used: “Using nQuery (Version 8.5.2.0, Table MOT1-1 Paired t-test for differences in means, Statistical Solutions Ltd.), a sample size of 9 participants was calculated as required to achieve a power of 80%”.***

Except these minor points, the article is acceptable. However, mind you that as pointed out in ‘important note’ above “This review pertains only to ‘statistical aspects’ of the study and so ‘clinical aspects’ should be assessed separately/independently. ‘Minor Revision’ is recommended.

6. PLOS authors have the option to publish the peer review history of their article ([what does this mean?](https://urldefense.com/v3/__https:/journals.plos.org/plosone/s/editorial-and-peer-review-process*loc-peer-review-history__;Iw!!Jgbw!G3H3Z-2kwqIpE4XNB0ptiJw4WseJsz65z6clK7O3V0UdJ8c0usOqjsBtfBW1MWbePZ8aTEdhwy6S-5lzAbXT$)). If published, this will include your full peer review and any attached files.

**Do you want your identity to be public for this peer review?** For information about this choice, including consent withdrawal, please see our [Privacy Policy](https://urldefense.com/v3/__https:/www.plos.org/privacy-policy__;!!Jgbw!G3H3Z-2kwqIpE4XNB0ptiJw4WseJsz65z6clK7O3V0UdJ8c0usOqjsBtfBW1MWbePZ8aTEdhwy6S-6Oqk7oi$).

Reviewer #1: No

Reviewer #2: No

 Thank you for considering our re-submission.

Yours faithfully,

**Dr Ian R Woodcock MBBS MSc MRCPCH(UK) FRACP**

**Paediatric Neurologist, The Royal Children's Hospital, Melbourne**

**PhD Student, The University of Melbourne**

**Honorary Fellow, The Murdoch Childrens Research Institute, Melbourne**

**Melbourne Children’s Campus
50 Flemington Road, Parkville, Victoria, 3052
T: 03 9345 5661**

**E:** [**Ian.Woodcock@rch.org.au**](about:blank)
